# Phospho­rescent mono- and diiridium(III) complexes cyclo­metalated by fluorenyl- or phenyl-pyridino ligands with bulky substituents, as prospective OLED dopants

**DOI:** 10.1107/S2056989020001784

**Published:** 2020-02-18

**Authors:** Ahmed M’hamedi, Andrei S. Batsanov

**Affiliations:** a Department of Chemistry, College of Sciences and Humanities in Al-Kharj, Prince Sattam bin Abdulaziz University, Al Kharj 11942, Saudi Arabia; b Laboratory of Structure Determination, Development, and Application of Molecular Material, Department of Chemistry, University of Abdelhamid Ibn Badis BP 227, Mostaganem 27000, Algeria; c Department of Chemistry, University of Durham, South Road, Durham DH1 3LE, United Kingdom

**Keywords:** crystal structure, OLED, optoelectronic, phospho­rescence

## Abstract

The crystal structures of tris­[9,9-dihexyl-2-(5-meth­oxy­pyridin-2-yl-κ*N*)-9*H*-fluoren-3-yl-κ*C* ^3^]iridium pentane monosolvate, [Ir(C_31_H_38_NO)_3_]·C_5_H_12_, (I), di-μ_2_-chlorido-bis­{bis­[2-(5-fluoro­pyridin-2-yl)-9,9-dihexyl-9*H*-fluoren-3-yl]iridium} pentane 0.3-solvate, [Ir_2_(C_30_H_35_FN)_4_Cl_2_]·0.3C_5_H_12_, (II), di-μ_2_-cyanato-bis­{bis­[9,9-dihexyl-2-(5-meth­oxy­pyridin-2-yl)-9*H*-fluoren-1-yl]iridium} pentane monosolvate, [Ir_2_(C_31_H_38_NO)_4_(NCO)_2_(NCO)_2_]·C_5_H_12_, (III), and {μ-*N*,*N*′-bis­[3,5-bis­(tri­fluoro­meth­yl)phen­yl]oxamidato}bis(bis{2-[4-(2,4,6-trimethylphenyl)pyridin-2-yl]phenyl-κ^2^ *C* ^1^,*N*′}iridium)–chloro­benzene–pentane (1/2.3/0.4), [Ir_2_(C_20_H_19_N)_4_(C_18_H_6_F_12_N_2_O_2_)]·2.3C_6_H_5_Cl·0.4C_5_H_12_, (IV), synthesized in the quest for organic light-emitting devices, were determined. The bis-μ_2_-chloro and bis-μ_2_-cyanato complexes have ΔΔ and ΛΛ configurations of the distorted octa­hedral Ir centres in racemic crystals, whereas the oxamido complex has a centrosymmetric (*meso*) structure with the ΔΛ configuration. The bridging oxamido moiety has a nearly planar *anti* geometry. All structures show substantial disorder of both host mol­ecules and solvents of crystallization.

## Abstract 2

The structures of four phospho­rescent monoiridium and chloro-, cyanato- or oxamidato-bridged diiridium complexes with a potential for OLED applications are described.

## Chemical context   

Over the two decades since the pioneering work of Baldo *et al.* (1998 ▸), cyclo­metalated Ir^III^ complexes have been developed as emitters (phospho­rescent dopants) for organic light-emitting diodes (OLEDs) or light-emitting electrochemical cells (LECs) (Li *et al.*, 2018 ▸; Adeloye, 2019 ▸). These complexes are structurally and synthetically versatile. A large contribution of the Ir orbitals to the excited state results in efficient spin-orbit coupling, allowing in principle the harvesting of all the electro-generated excitons and a 100% inter­nal quantum efficiency of electro-phospho­rescence. Because the luminescent properties are metal-ligand based, colour fine-tuning can be achieved by choosing the ligands (of which phenyl­pyridine, ppy, is by far the most important) and substituents to systematically vary the electronic and steric properties, or by incorporation of ancillary ligands. Originally the research focused on mono-iridium species, because the di-iridium complexes then known (mostly bis-μ-chloro bridged) proved generally poor emitters because of their electron-withdrawing bridges; for example [Ir(ppy)_2_Cl]_2_ has a quantum yield 80 times lower than *fac*-Ir(ppy)_3_ (King *et al.*, 1985 ▸).
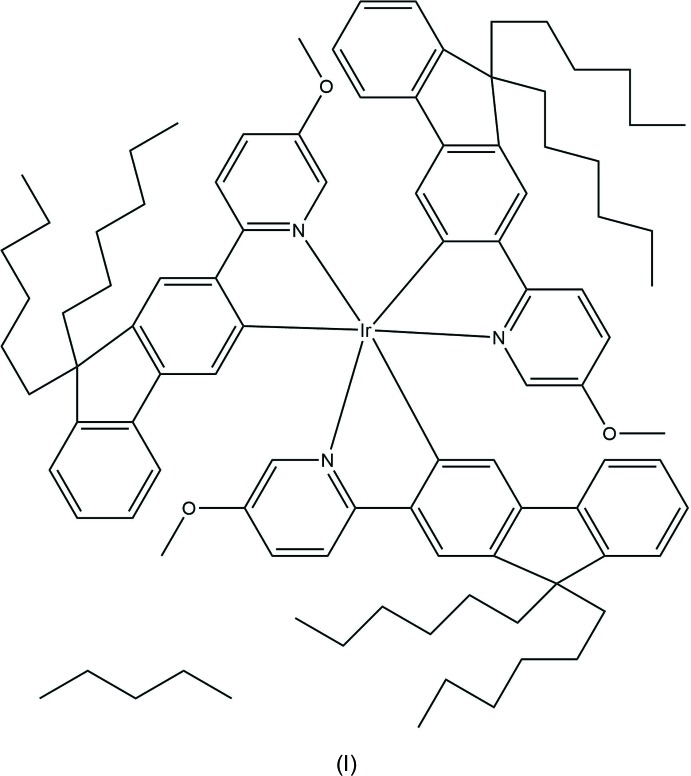
 
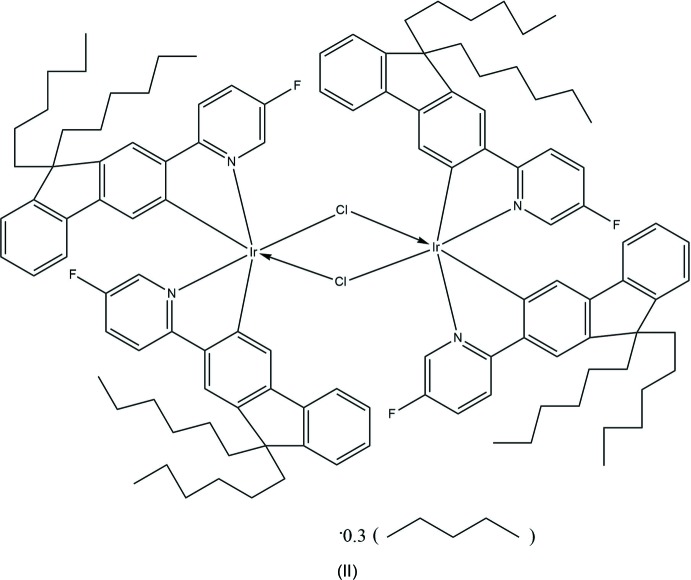
 
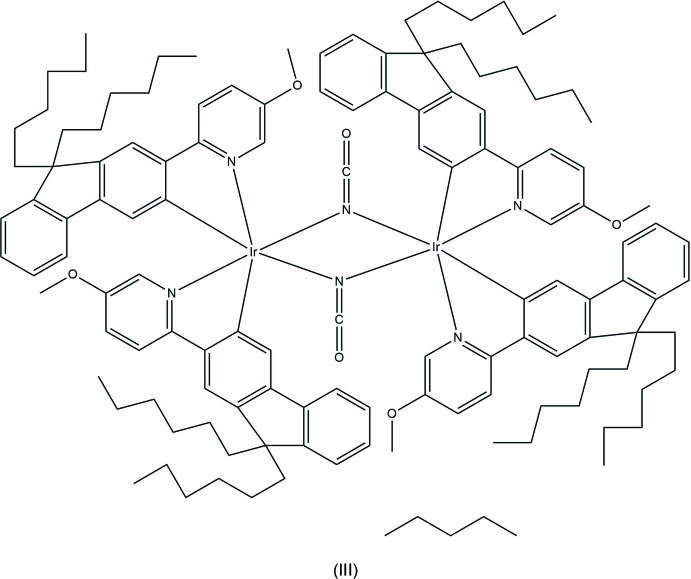
 
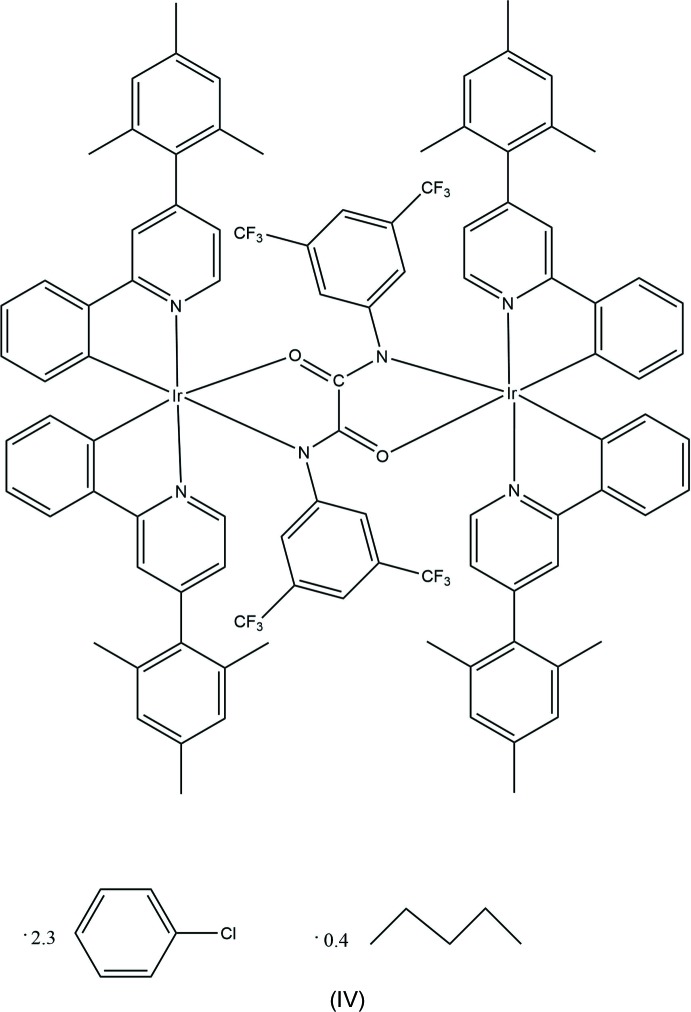


The mono-iridium complex (I), has shown external quantum efficiency (EQE) in devices of 4.8% and current efficiency of 14 cd A^−1^, compared to 0.9% and 3 cd A^−1^ for complex (II), which is nevertheless exceptionally good for its class (M’hamedi *et al.*, 2012 ▸). Better results (2.6% and 8 cd A^−1^) were obtained with complex (III), although NCO ligands are also electron-withdrawing. Later some bridges were found to be propitious for high-yield phospho­rescence, particularly di­aryl­hydrazido (Congrave *et al.*, 2017 ▸), oxamido and di­aryl­oxamido ligands (Graf *et al.*, 2013 ▸; M’hamedi *et al.*, 2017 ▸). Here we report the X-ray crystal structures of complexes (I)–(IV), in relation to their optoelectronic properties.

It is noteworthy that the optoelectronic properties of such complexes depend strongly on the position of the substituents in the pyridine ring. Whereas both electron-donating (OMe) or electron-withdrawing (F) substituents are known to lower the device efficiency of mono-iridium complexes – probably by perturbing the electron and hole mobilities and hence reducing the exiton formation (Al-Attar *et al.*, 2011 ▸) – a substituent *para* to the N atom lowers the device efficiencies drastically (M’hamedi *et al.*, 2012 ▸).

## Structural commentary   

**Figure 1:**
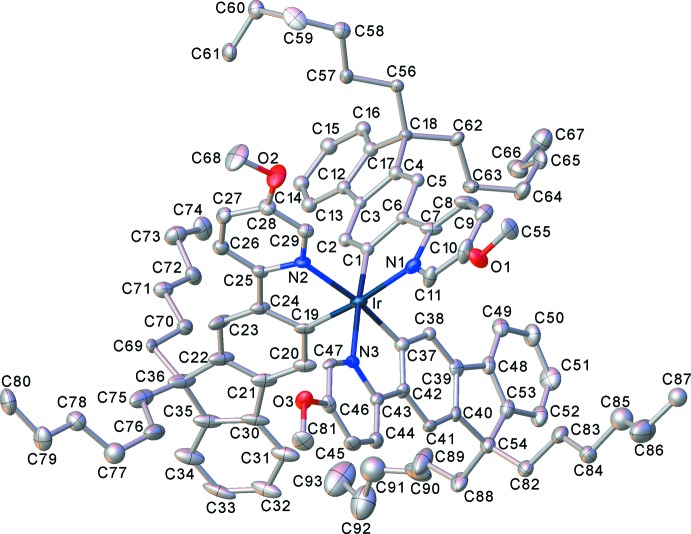
The mol­ecular structure of (I), showing 50% probability displacement ellipsoids for non-H atoms. The disorder and all H atoms are omitted for clarity.

**Figure 2:**
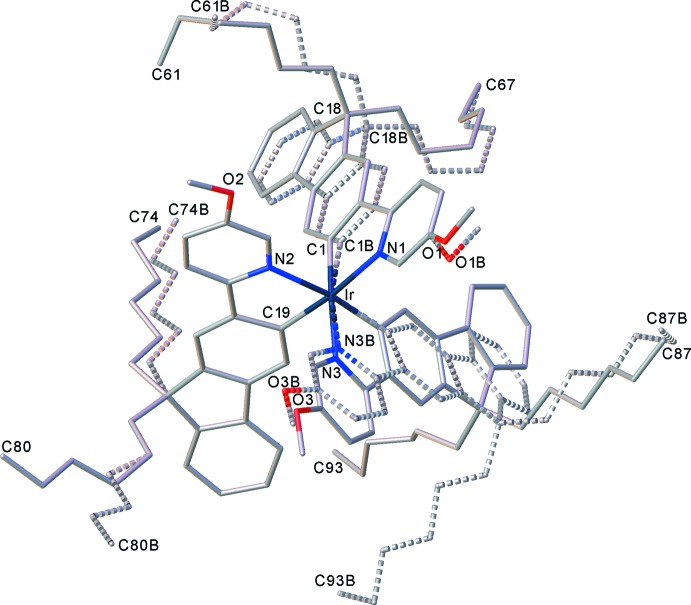
Disorder in the structure of (I); H atoms are omitted. All disordered fragments have 50% occupancies

The mononuclear complex (I) (Fig. 1 ▸) crystallized as a pentane monosolvate, like its isomer (I*a*), which had the meth­oxy substituent in the 4- rather than 5-position of the pyridine ring (M’hamedi *et al.*, 2012 ▸), and shows a rather similar mol­ecular geometry. Both structures show extensive disorder; however, while in (I*a*) it is confined to the *n*-hexyl chains and the solvent, in (I) the disorder (between two half-occupied positions) involves one ligand entirely, most of another one (except the pyridine ring) and both *n*-hexyl chains of the third ligand, as well as the pentane mol­ecule (Fig. 2 ▸). The Ir coordination in (I) is distorted *fac*-octa­hedral, with each Ir—N bond in a *trans* orientation to an Ir—C bond, confirming the earlier assessment from NMR spectra (M’hamedi *et al.*, 2012 ▸). The mean distances Ir—N = 2.13 (1) and Ir—C = 2.02 (1) Å are slightly longer than those in (I*a*) (2.119 and 2.006 Å, respectively) and similar to those in the unsubstituted Ir(ppy)_3_, both in its trigonal (Breu *et al.*, 2005 ▸) and tetra­gonal polymorphs (Berger *et al.*, 2010 ▸; Takayasu *et al.*, 2013 ▸; Wang *et al.*, 2013 ▸).

**Figure 3:**
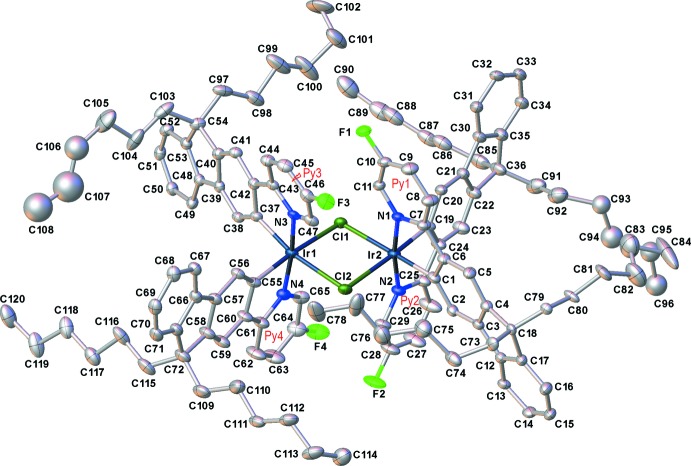
The mol­ecular structure of (II), showing 30% probability displacement ellipsoids and the notation of pyridyl rings. The disorder and all H atoms are omitted.

**Figure 4:**
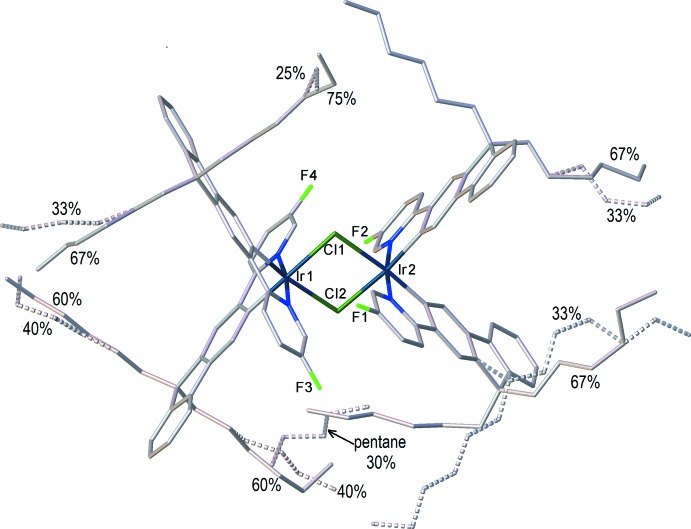
Disorder in the structure of (II), showing occupancies. H atoms are omitted.

Complex (II) crystallized from chloro­benzene/pentane as a non-stoichiometric pentane solvate, in a triclinic structure with one independent mol­ecule in a general position. In contrast, its analogues with non-substituted (II*a*) and 4-fluorinated (II*b*) pyridyl rings (M’hamedi *et al.*, 2012 ▸), which crystallized from the *same* solvents as the chloro­benzene solvates, as well a CH_2_Cl_2_/water solvate of II*a* (II*a*′) (Bettington *et al.*, 2006 ▸), all form isomorphous ortho­rhom­bic crystals (space group *Fddd*) with the crystallographic mol­ecular symmetry 222. Notably, the analogue of (II) with 4-meth­oxy-substituted pyridine rings (II*c*) also crystallized in a triclinic structure, with the lattice parameters and host mol­ecules’ packing broadly similar to those of (II), albeit with more solvent of crystallization (two chloro­benzene and three pentane mol­ecules per formula unit) and correspondingly expanded unit-cell volume (M’hamedi *et al.*, 2012 ▸). The mol­ecular geometry of (II) (Fig. 3 ▸), as that of (II*c*), only approximately conforms to *C*_2_ symmetry: the Ir_2_Cl_2_ system is not planar, as in (II*a*) and (II*b*), but folded by 7.7 (1)° (II) or 8.26 (3)° (II*c*) along the Cl⋯Cl vector. In (II), the iridium atoms have distorted octa­hedral coordination with the N atoms *trans* to one another and both C atoms *trans* to bridging chloro ligands. The mean bond lengths Ir—Cl = 2.507 (2), Ir—N = 2.036 (5) and Ir—C = 1.998 (6) Å are not substanti­ally different from those in (II*a*) [2.498 (1), 2.038 (5) and 1.993 (6) Å, respectively] or (II*b*) [2.498 (2), 2.016 (7) and 1.977 (8) Å, respectively], although the Ir1⋯Ir2 distance in (II) [3.740 (1) Å] is appreciably longer than in (II*a*) or (II*b*) (3.675 or 3.674 Å, respectively). Both Ir centres in the mol­ecule of (II) have the same configuration: either ΔΔ or ΛΛ, the crystal being racemic. The *n*-hexyl chains of the ligands show complicated disorder (Fig. 4 ▸), being superimposed upon a pentane mol­ecule of crystallization with a partial (*ca* 30%) occupancy.

Near-parallel alignment of pyridyl rings coordinated to different Ir atoms [inter­planar angles Py1/Py = 13.4 (2)°, Py2/Py4 = 6.3 (3)°, see Fig. 3 ▸], may seem propitious to intra­molecular π–π stacking, which is important for optoelectronic properties in these phases. However, with the parallel slip distance of 2.73 Å between rings Py1 and Py3, and of 3.62 Å between Py2 and Py4, the π-systems of the former pair overlap only on the fringes and the latter not at all. The closest contacts are C10⋯C46 = 3.35 (1) and C11⋯C47 = 3.10 (1) Å in the Py1/Py3 pair and C28⋯C64 = 3.51 (1) and C29⋯C65 = 3.21 (1) Å for Py2/Py4. Other di­chloro-bridged complexes show a similar pattern: in (II*a*) and (II*b*), the Py/Py angles are 9.4 (3) and 6.3 (4)°, with parallel slips of 2.94 and 2.88 Å, respectively. In (II*c*), the two Py/Py pairs are even more unequal than in (II): one showing a fringe overlap [Py/Py angle = 5.1 (1)°, slip = 3.0 Å, shortest C⋯C contacts of 3.36 (1) and 3.42 (1) Å] and the other a more substantial overlap [Py/Py angle = 7.6 (1)°, slip of only 2.03 Å] and the mean inter­planar separation being 3.21 Å.

**Figure 5:**
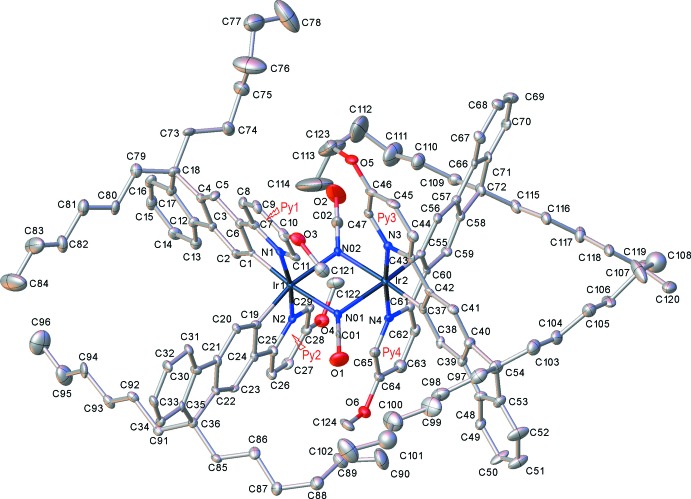
The mol­ecular structure of (III), showing 30% probability displacement ellipsoids for non-H atoms. The disorder and all H atoms are omitted for clarity.

**Figure 6:**
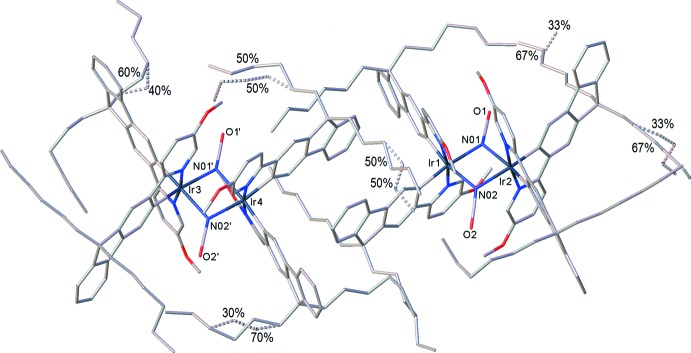
Disorder in the structure of (III), showing occupancies. H atoms are omitted.

Complex (III) (Fig. 5 ▸) crystallized as a pentane monosolvate in a centrosymmetric monoclinic structure with two mol­ecules per asymmetric unit. Like (II), (III) crystallized as a racemate; the mol­ecule has no crystallographic symmetry but an approximate local twofold symmetry relating the iridium centres, which have the same configurations. Thus, mol­ecules with ΔΔ or ΛΛ configurations are equally present, whereas the previously reported analogues of (III) with non-substituted (III*a*) or 4-fluorinated (III*b*) pyridyl rings (M’hamedi *et al.*, 2012 ▸) gave non-solvated chiral crystals (space group *P*2_1_), which were isomorphous and contained one independent mol­ecule each. The precision of structure (III) is limited, due to massive disorder, generally weak diffraction intensities and, possibly, incommensurate modulation along the a* direction, as indicated by ‘streaky’ reflection peaks. The intra­molecular Ir⋯Ir distances in (III), 3.410 (1) and 3.432 (1) Å, are similar to those in (III*a*) [3.402 (1) Å] and III*b* [3.425 (1) Å] and *ca* 0.3 Å shorter than in chloro-bridged complexes. Similarly to (II) and especially to (II*c*), in each independent mol­ecule one pair of pyridyl rings shows substantial π–π stacking [inter­planar Py/Py angles of 11.4 (3) and 10.7 (4)°, slips of 1.90 Å, mean inter­planar separations of 3.25 Å], the other only a fringe overlap [Py/Py angles = 13.9 (3) and 19.5 (4)°, slips of 2.88 and 2.83 Å]. In this structure, the *n*-hexyl chains also show extensive disorder (Fig. 6 ▸), which could be only imperfectly resolved. These chains surround well-defined voids containing disordered solvent, which was masked. The electron density maps, and the shape and size of the voids suggest the solvent to be pentane rather than chloro­benzene.

**Figure 7:**
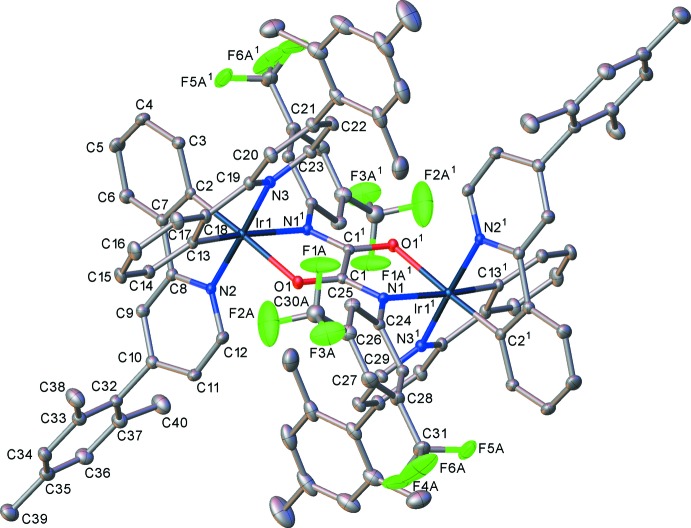
The mol­ecular structure of (IV), showing 50% probability displacement ellipsoids for non-H atoms. Symmetry code (^1^): 1 − *x*, 1 − *y*, 1 − *z*.

**Figure 8:**
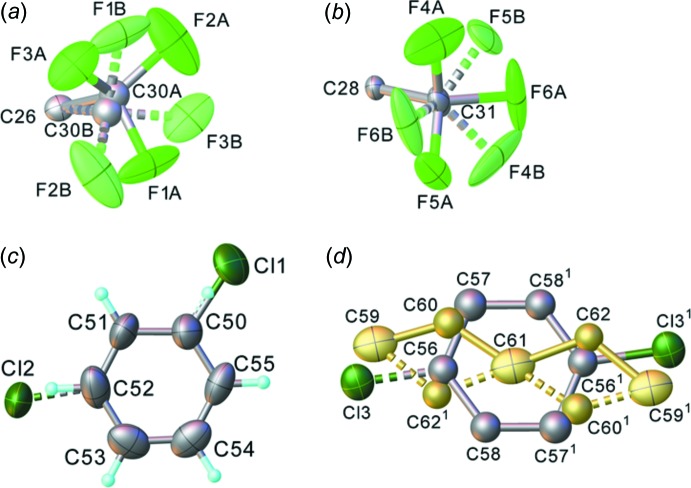
The disorder of the CF_3_ groups (*a*), (*b*), chloro­benzene of crystallization (*c*) and chloro­benzene/pentane site-sharing (*d*) [symmetry code (^1^): −*x*, −*y*, −*z*] in the structure of (IV).

In fact, for such diiridium complexes with *mono*atomic bridges, both metal centres have to adopt the same chirality: the *meso* diastereomer with opposite (ΔΛ) configurations of the two iridium centres would have sterically impossible short intra­molecular contacts between the cyclo­metalating ligands. With longer bridges, however, both the ΔΔ/ΛΛ and *meso* isomers are sterically possible – and in some cases have been isolated and structurally characterized (Congrave *et al.*, 2017 ▸). Thus, the mol­ecule of (IV) (Fig. 7 ▸) is crystallographically centrosymmetric, i.e. *meso.* As in complexes (II) and (III), the Ir atoms in (IV) have a distorted octa­hedral coordination with the pyridine N atoms in *trans* positions to each other. The bridging oxamido ligand adopts the *anti* geometry, its central C_2_N_2_O_2_ moiety is almost planar, the 3,5-tri­fluoro­methyl­ated arene ring is inclined to its plane by 47.3 (1)° and the Ir atom deviates from it by 0.334 (4) Å, which amounts to an 11.2 (1)° fold of the chelate ring along the O1⋯N1^1^ vector [symmetry code: (i) 1 − *x*, 1 − *y*, 1 − *z*]. In both independent cyclo­metalating ligands, the mesityl substituent is almost perpendicular to the pyridine ring [dihedral angles 85.9 (1) and 81.5 (1)°]. The Ir1—N1^i^ bond of 2.182 (2) Å is substanti­ally longer than in the analogue with a 4-*tert*-butyl­phenyl substituent at this N atom [2.147 (3) Å, *see above*]. The CF_3_ groups and the solvent of crystallization are disordered (Fig. 8 ▸).

Recently, we prepared and characterized two close analogues of (IV), which proved efficient as dopants in phospho­r­escent organic light-emitting devices (M’hamedi *et al.*, 2017 ▸), *viz*. tetra­kis­[2-phenyl-4-(mesit­yl)pyridine-*C* ^2^,*N*′]-bis­(μ-oxa­mid­ato)diiridium (IV*a*) with an unsubstituted oxamidato bridge, and its derivative with an *N*,*N*′-bis­(4-*tert*-butyl­phen­yl)-substituted bridge (IV*b*). As in (IV), both have an *anti*-configuration of the bridge: (IV*b*) certainly, (IV*a*) most probably, although in the latter the bridge is disordered, as in (μ_2_-oxamidato)tetra­kis­(5-methyl-2-(pyridin-2-yl)phen­yl)diiridium (V), reported by Graf *et al.* (2013 ▸). It is noteworthy that both (IV*b*) and (V), like (IV), are *meso* (ΔΛ) diastereomers where the Ir centres are related by a crystallographic inversion centre (IV*b*) or mirror plane (V), whereas mol­ecule (IV*a*) lies on a crystallographic twofold axis in the centrosymmetric space group *Pbcm*, *i.e*. the crystal is a racemate of ΔΔ and ΛΛ diastereomers.

## Supra­molecular features   

The packing of (I)–(III) is defined by the flexible and extensively disordered *n*-hexyl substituents, with the remaining gaps filled by pentane solvent mol­ecules. There are few inter­molecular contacts between aromatic moieties, and those of the edge-to-edge kind; no π–π stacking or inter­calation exists. This promises better solubility and compatibility with the host polymer and, indeed, can explain the relatively high efficiency of these complexes in optoelectronic devices (M’hamedi *et al.*, 2012 ▸). The structure of (IV) is also devoid of any π–π stacking; the contacts between all aromatic systems, whether ppy moieties, mesityl substituents or chloro­benzene mol­ecules of crystallization, are of edge-to-face (‘π–σ’) type.

## Database survey   

The Cambridge Crystallographic Database (CSD, version 5.40, including updates up to August 2019; Groom *et al.*, 2016 ▸) was searched for bis-chloro-bridged diiridium complexes with cyclo­metalating fluorenyl-pyridino ligands. No structures were found besides ACISUB (II*a*′), NIFKEU (II*a*), NIFKAQ (II*b*) and NIFJUJ (II*c*), discussed above. A search for similar bis-cyanato-bridged complexes returned structures NIFJOD (III*a*) and NIFJIX (III*b*), also discussed above. No Ir(μ_2_-*X*)Ir analogues with other *X* ligands were found. A search for *anti*-oxamido-bridged diiridium complexes with two chelating phenyl­pyridyl moieties (with any substituents) at each Ir atom, returned three structures discussed above, *viz*. SAWDIG (IV*a*), SAWDEC (IV*b*) and GIRMIF (V). The only mono-iridium complexes cyclo­metalated by three fluorenyl-pyridino derivatives, are NIFJET (I*a*, see above) and tris­(2-(pyridin-2-yl)spiro­[fluorene-9,9′-xanthen]-3-yl)-iridium, SAWRIU (Ren *et al.*, 2017 ▸).

Of the four structures of *fac*-Ir(ppy)_3_ present in the CSD, KAVJOH is the trigonal polymorph (Breu *et al.*, 2005 ▸), KAVJOH01 (Berger *et al.*, 2010 ▸) and KAVJOH03 (Takayasu *et al.*, 2013 ▸) refer to the same tetra­gonal form (space group 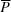
42_1_ *c*) at 100 and 193 K, respectively, whereas KAVJOH02 (Wang *et al.*, 2013 ▸) is probably the latter structure solved in a wrong space group (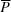
4).

## Synthesis and crystallization   

Complexes (I), (II) and (III) were synthesized as described elsewhere (M’hamedi *et al.*, 2012 ▸), *viz*. (I) and (II) by the reaction of the corresponding fluorenyl­pyridine with Ir(acac)_3_ or IrCl_3_, respectively, (III) by reaction of the meth­oxy-substituted analogue of (II) (*i.e*. di-chloro-bridged complex) with Bu_4_N^+^NCO^−^. All three complexes were recrystallized from the mixed solvents chloro­benzene/pentane.

Complex (IV) was prepared following the previous procedure for the synthesis of diiridium(III) complexes with bridging oxamidato ligands (M’hamedi *et al.*, 2017 ▸): a mixture of sodium methoxide (20.8 mg, 3.0 eq.) in methanol (2 ml), and *N,N′*-bis­(3,5-bis­(tri­fluoro­methyl­phen­yl)oxamide (98.6 mg, 1.5 eq.) in methanol (6 ml) and the corresponding di­chloro-bridged dimer (Rota Martir *et al.*, 2016 ▸) (200 mg, 0.128 mmol) in THF (15 ml) gave a product that was purified by column chromatography over silica gel, eluting with di­chloro­methane (saturated with K_2_CO_3_ and 2% Et_3_N) to give (IV) as a yellow–orange coloured solid (141 mg, 58% yield). Crystals were grown from a chloro­benzene/pentane solution. MS (MALDI–TOF) *m/z* 1984.2 ([*M* + H, ^191^Ir, ^193^Ir], 100%). Calculated for C_98_H_78_F_12_Ir_2_N_6_O_2_ *m*/*z* 1984.53.

## Refinement   

Crystal data, data collection and structure refinement details are summarized in Table 1 ▸.

In structure (I), the entire cyclo­metalating ligand N3^^^C37, ligand N1^^^C1 except the pyridyl ring and the methyl atom C67, and *n*-hexyl substituents (except atoms C75 and C76) in the ligand N2^^^C19 are disordered equally between two positions (one of them labelled *B*). In the pentane solvent mol­ecule, all atoms except C(1*S*) are also disordered between two positions (alternative carbon positions are primed).

In structure (II), atom C18 of the fluorene ring and both *n*-hexyl chains attached to it (except the C82 atom), chains C93—C94—C95—C96 and C105—C106—C107—C108 (with their respective H atoms) are disordered over two sets of sites with occupancies 2/3 and 1/3. The butyl chain at C110 is disordered between positions C111—C112—C113—C114 and C211—C212—C213—C214, the butyl chain at C116 is disordered between positions C117—C118—C119—C120 and C217—C218—C219—C220 (with respective H atoms); both in a 0.6:0.4 ratio, respectively. The ethyl group at C100 is disordered between positions C101—C102 and C201—C202 in a 0.75:0.25 ratio. All minor positions are labelled *B*. The pentane mol­ecule of crystallization, C1*S*—C2*S*—C3*S*—C4*S*—C5*S* (with respective H atoms) shares the site with disordered *n*-hexyl groups and was refined with a 0.3 occupancy.

In structure (III), the ethyl group at C100 is disordered between positions C101—C102 and C125—C126 and the butyl group at C104 between positions C105—C106—C107—C108 and C10*A*—C10*B*—C10*C*—C10*D*, with occupancies 2/3 and 1/3, respectively, in both cases. A methyl­ene group is disordered between positions C80′ and C80*B* with occupancies 0.6 and 0.4 and a di­methyl­ene fragment is disordered between positions C204—C205 and C20*B*—C20*C* with occupancies 0.7 and 0.3, respectively. The propyl chain at C211 is disordered between positions C212—C213—C214 and C21*B*—C21*C*—C21*D*, and the butyl chain at C216 is disordered between positions C217—C218—C219—C220 and C21*E*—C21*F*—C21*G*—C21*H* with equal (0.5) occupancies in both cases. The structure contains enclosed solvent-accessible voids of 374 Å^3^ (four per unit cell), occupied by disordered solvent, which could not be refined at atomic resolution and was masked using the *OLEX2* SMTBX solvent-masking procedure based on Rees *et al.* (2005 ▸). The diffuse electron density in the voids being too low for chloro­benzene, the integral of 70 e per void can be inter­preted as two pentane mol­ecules (42 e each).

In structures (I)–(III), all H atoms were permitted to ride in geometrically idealized positions with C—H = 0.95, 0.99 and 0.98 Å for aromatic, methyl­ene and methyl C atoms, respectively.

In structure (IV), aromatic H atoms were permitted to ride in geometrically idealized positions with C—H = 0.95 Å. Methyl groups were ascribed idealized geometry (C—H = 0.98 Å) and were permitted to rotate around the C—C bonds (with a common refined *U*_iso_ for all H atoms of each group), except the C48H_3_ group, which was treated as ideally disordered between two opposite orientations. For the latter and aromatic H atoms, *U*_iso_(H) = 1.2*U*_eq_(C). The C31F_3_ group is disordered (by rotation about the C—C bond) between orientations *A* and *B* with occupancies of 0.774 (5) and 0.226 (5), respectively, while the C30F_3_ group is disordered by a similar rotation *and* tilt of the C—C bond, between orientations *A* and *B* with occupancies of 0.586 (15) and 0.414 (15), respectively. The chloro­benzene mol­ecule in a general position has the chlorine atom disordered equally between positions Cl1 and Cl2. The void around the inversion centre (0, 0, 0) with the solvent-accessible volume of 204 Å^3^ is shared by *ca* 0.15 of a chloro­benzene and 0.20 of a pentane mol­ecule, refined at atomic resolution; the occupancies are in agreement with the integral electron density of 33.4 e per void, as estimated by *OLEX2* SMTBX. The benzene ring has crystallographic inversion symmetry: the Cl atom is disordered between two positions related by this inversion. The central pentane atom C61 lies at the inversion centre, the adjacent atom is disordered equally between two positions with the terminal atom ordered. The pentane H atoms were not located. A strong peak of residual electron density (4.2 e Å^−3^) near the Ir1 atom can be inter­preted as an alternative position, Ir1, of this atom, due to a whole-mol­ecule disordered by a 12° rotation around its (crystallographic) inversion centre. Refinement of the occupancies of Ir1 and Ir2 (assuming equal ADPs) converged at 0.9817 (6) and 0.0183 (6), respectively, with a Ir1⋯Ir2 distance of 0.62 Å.

## Supplementary Material

Crystal structure: contains datablock(s) I, II, III, IV, global. DOI: 10.1107/S2056989020001784/hb7885sup1.cif

Structure factors: contains datablock(s) I. DOI: 10.1107/S2056989020001784/hb7885Isup2.hkl

Supporting information file. DOI: 10.1107/S2056989020001784/hb7885Isup6.cdx

Structure factors: contains datablock(s) II. DOI: 10.1107/S2056989020001784/hb7885IIsup3.hkl

Supporting information file. DOI: 10.1107/S2056989020001784/hb7885IIsup7.cdx

Structure factors: contains datablock(s) III. DOI: 10.1107/S2056989020001784/hb7885IIIsup4.hkl

Supporting information file. DOI: 10.1107/S2056989020001784/hb7885IIIsup8.cdx

Structure factors: contains datablock(s) IV. DOI: 10.1107/S2056989020001784/hb7885IVsup5.hkl

Supporting information file. DOI: 10.1107/S2056989020001784/hb7885IVsup9.cdx

CCDC references: 1982629, 1982628, 1982627, 1982626

Additional supporting information:  crystallographic information; 3D view; checkCIF report

## Figures and Tables

**Table 1:** Experimental details

	(I)	(II)	(III)	(IV)
Crystal data				
Chemical formula	[Ir(C_31_H_38_NO)_3_]·C_5_H_12_	[Ir_2_(C_30_H_35_FN)_4_Cl_2_]·0.3C_5_H_12_	[Ir_2_(C_31_H_38_NO)_4_(NCO)_2_]·C_5_H_12_	[Ir_2_(C_20_H_19_N)_4_(C_18_H_6_F_12_N_2_O_2_)]·2.3C_6_H_5_Cl·0.4C_5_H_12_
*M*_r_	1586.21	2191.30	2303.07	2271.78
Crystal system, space group	Triclinic, *P* 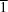	Triclinic, *P* 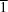	Monoclinic, *P*2_1_/*c*	Triclinic, *P* 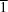
Temperature (K)	120	120	120	120
*a*, *b*, *c* (Å)	16.4880 (5), 17.0003 (5), 17.0288 (5)	12.2744 (7), 17.6132 (10), 25.3966 (15)	38.976 (6), 21.615 (3), 28.759 (4)	11.8734 (5), 14.2267 (6), 16.6076 (7)
α, β, γ (°)	91.9252 (12), 97.8137 (12), 117.3489 (11)	105.119 (2), 93.787 (2), 90.779 (2)	90, 108.920 (3), 90	110.386 (2), 106.524 (2), 96.303 (2)
*V* (Å^3^)	4175.2 (3)	5286.2 (5)	22920 (6)	2452.00 (18)
*Z*	2	2	8	1
Radiation type	Mo *K*α	Mo *K*α	Mo *K*α	Mo *K*α
μ (mm^−1^)	1.65	2.62	2.38	2.85
Crystal size (mm)	0.11 × 0.07 × 0.04	0.14 × 0.1 × 0.04	0.2 × 0.1 × 0.01	0.12 × 0.08 × 0.04

Data collection				
Diffractometer	Bruker SMART CCD 6000	Bruker SMART CCD 6000	Bruker SMART CCD 6000	Bruker D8 Venture
Absorption correction	Multi-scan (*SADABS*; Krause *et al.*, 2015 ▸)	Integration (*SADABS*; Krause *et al.*, 2015 ▸)	Multi-scan (*SADABS*; Krause *et al.*, 2015 ▸)	Integration (*SADABS*; Krause *et al.*, 2015 ▸)
*T*_min_, *T*_max_	0.803, 0.850	0.735, 0.927	0.809, 1.000	0.770, 0.913
No. of measured, independent and observed [*I* > 2σ(*I*)] reflections	46917, 19128, 13949	48264, 18623, 12096	95765, 40676, 19348	54411, 14405, 12696
*R*_int_	0.071	0.065	0.143	0.032
(sin θ/λ)_max_ (Å^−1^)	0.650	0.595	0.601	0.705

Refinement				
*R*[*F* ^2^ > 2σ(*F* ^2^)], *wR*(*F* ^2^), *S*	0.042, 0.076, 0.91	0.041, 0.103, 0.95	0.077, 0.185, 0.97	0.028, 0.073, 1.06
No. of reflections	19128	18623	40676	14405
No. of parameters	1358	1342	2479	702
No. of restraints	1114	2905	2600	593
H-atom treatment	H-atom parameters constrained	H-atom parameters constrained	H-atom parameters constrained	H atoms treated by a mixture of independent and constrained refinement
Δρ_max_, Δρ_min_ (e Å^−3^)	1.73, −0.86	1.39, −0.76	2.80, −2.20	0.96, −1.01

Computer programs: *SMART* (Bruker, 2001 ▸), *SAINT* (Bruker, 2001 ▸, 2008 ▸, 2016 ▸, 2017 ▸), *APEX3* (Bruker, 2016 ▸), *SHELXS* (Sheldrick, 2008 ▸), *SHELXT2018* (Sheldrick, 2015*a* ▸), *SHELXL2018* (Sheldrick, 2015*b* ▸) and *OLEX2* (Dolomanov *et al.*, 2009 ▸).

## supplementary crystallographic information

### \ Tris[9,9-dihexyl-2-(5-methoxypyridin-2-yl-κN)-9H-fluoren-3-\ yl-κC3]iridium pentane monosolvate (I) Crystal data

**Table:**

[Ir(C_31_H_38_NO)_3_]·C_5_H_12_	*Z* = 2
*M**_r_* = 1586.21	*F*(000) = 1672
Triclinic, *P*1	*D*_x_ = 1.262 Mg m^−^^3^
*a* = 16.4880 (5) Å	Mo *K*α radiation, λ = 0.71073 Å
*b* = 17.0003 (5) Å	Cell parameters from 9920 reflections
*c* = 17.0288 (5) Å	θ = 2.4–26.4°
α = 91.9252 (12)°	µ = 1.65 mm^−^^1^
β = 97.8137 (12)°	*T* = 120 K
γ = 117.3489 (11)°	Block, yellow
*V* = 4175.2 (3) Å^3^	0.11 × 0.07 × 0.04 mm

### \ Tris[9,9-dihexyl-2-(5-methoxypyridin-2-yl-κN)-9H-fluoren-3-\ yl-κC3]iridium pentane monosolvate (I) Data collection

**Table:**

Bruker SMART CCD 6000 diffractometer	19128 independent reflections
Radiation source: sealed X-ray tube	13949 reflections with *I* > 2σ(*I*)
Parallel,graphite monochromator	*R*_int_ = 0.071
Detector resolution: 5.6 pixels mm^-1^	θ_max_ = 27.5°, θ_min_ = 1.2°
ω scans	*h* = −21→21
Absorption correction: multi-scan (SADABS; Krause *et al.*, 2015)	*k* = −22→22
*T*_min_ = 0.803, *T*_max_ = 0.850	*l* = −22→22
46917 measured reflections	

### \ Tris[9,9-dihexyl-2-(5-methoxypyridin-2-yl-κN)-9H-fluoren-3-\ yl-κC3]iridium pentane monosolvate (I) Refinement

**Table:**

Refinement on *F*^2^	1114 restraints
Least-squares matrix: full	Hydrogen site location: mixed
*R*[*F*^2^ > 2σ(*F*^2^)] = 0.042	H-atom parameters constrained
*wR*(*F*^2^) = 0.076	*w* = 1/[σ^2^(*F*_o_^2^) + (0.026*P*)^2^] where *P* = (*F*_o_^2^ + 2*F*_c_^2^)/3
*S* = 0.91	(Δ/σ)_max_ = 0.004
19128 reflections	Δρ_max_ = 1.73 e Å^−^^3^
1358 parameters	Δρ_min_ = −0.85 e Å^−^^3^

### \ Tris[9,9-dihexyl-2-(5-methoxypyridin-2-yl-κN)-9H-fluoren-3-\ yl-κC3]iridium pentane monosolvate (I) Special details

**Table:**

Experimental. The data collection nominally covered full sphere of reciprocal space, by a combination of 4 runs of narrow-frame ω-scans (scan width 0.3° ω, 60s exposure, 2 x 600 + 552 +50 scans), every run at a different φ and/or 2θ angle. Crystal to detector distance 4.84 cm.
Geometry. All esds (except the esd in the dihedral angle between two l.s. planes) are estimated using the full covariance matrix. The cell esds are taken into account individually in the estimation of esds in distances, angles and torsion angles; correlations between esds in cell parameters are only used when they are defined by crystal symmetry. An approximate (isotropic) treatment of cell esds is used for estimating esds involving l.s. planes.
Refinement. extensive disorder

### \ Tris[9,9-dihexyl-2-(5-methoxypyridin-2-yl-κN)-9H-fluoren-3-\ yl-κC3]iridium pentane monosolvate (I) Fractional atomic coordinates and isotropic or equivalent isotropic displacement parameters (Å^2^)

**Table:**

	*x*	*y*	*z*	*U*_iso_*/*U*_eq_	Occ. (<1)
Ir	0.31386 (2)	0.66708 (2)	0.41146 (2)	0.01857 (4)	
O2	0.13331 (19)	0.60982 (18)	0.66372 (15)	0.0417 (7)	
N1	0.36370 (19)	0.7927 (2)	0.47826 (16)	0.0199 (7)	
N2	0.21147 (18)	0.59240 (19)	0.48141 (16)	0.0171 (6)	
C7	0.3206 (2)	0.8412 (3)	0.4527 (2)	0.0247 (8)	
C8	0.3470 (2)	0.9236 (3)	0.4933 (2)	0.0332 (10)	
H8	0.315976	0.956674	0.475663	0.040*	
C9	0.4172 (2)	0.9587 (3)	0.5587 (2)	0.0279 (9)	
H9	0.435109	1.015300	0.586099	0.033*	
C10	0.4604 (2)	0.9088 (3)	0.5834 (2)	0.0314 (9)	
C11	0.4323 (2)	0.8266 (2)	0.5417 (2)	0.0274 (9)	
H11	0.462678	0.792825	0.558575	0.033*	
C19	0.2515 (2)	0.5464 (2)	0.3469 (2)	0.0215 (8)	
C20	0.2749 (2)	0.5202 (3)	0.2775 (2)	0.0264 (8)	
H20	0.325118	0.562731	0.255432	0.032*	
C21	0.2255 (3)	0.4334 (3)	0.2407 (2)	0.0308 (9)	
C22	0.1493 (3)	0.3698 (3)	0.2695 (2)	0.0381 (10)	
C23	0.1240 (2)	0.3930 (3)	0.3370 (2)	0.0361 (10)	
H23	0.072209	0.350170	0.357125	0.043*	
C24	0.1752 (2)	0.4793 (2)	0.3762 (2)	0.0237 (8)	
C25	0.1543 (2)	0.5059 (2)	0.4501 (2)	0.0193 (7)	
C26	0.0853 (2)	0.4518 (2)	0.4915 (2)	0.0241 (8)	
H26	0.044333	0.392169	0.469197	0.029*	
C27	0.0751 (2)	0.4820 (2)	0.5630 (2)	0.0238 (8)	
H27	0.028585	0.443697	0.591056	0.029*	
C28	0.1344 (3)	0.5694 (2)	0.5937 (2)	0.0235 (8)	
C29	0.2009 (2)	0.6224 (2)	0.55021 (19)	0.0203 (8)	
H29	0.240932	0.682756	0.570954	0.024*	
C30	0.2415 (3)	0.3895 (3)	0.1730 (2)	0.0333 (9)	
C31	0.3118 (3)	0.4221 (3)	0.1271 (2)	0.0375 (10)	
H31	0.357650	0.482873	0.135077	0.045*	
C32	0.3126 (3)	0.3625 (3)	0.0698 (2)	0.0446 (12)	
H32	0.359715	0.383465	0.037724	0.054*	
C33	0.2469 (3)	0.2742 (3)	0.0580 (2)	0.0477 (12)	
H33	0.250002	0.234826	0.018991	0.057*	
C34	0.1762 (3)	0.2424 (3)	0.1028 (2)	0.0430 (11)	
H34	0.130227	0.181637	0.093934	0.052*	
C35	0.1742 (3)	0.3005 (3)	0.1605 (2)	0.0377 (10)	
C36	0.1066 (3)	0.2798 (3)	0.2189 (3)	0.0441 (11)	
C67	0.0931 (3)	1.0219 (3)	−0.0702 (3)	0.0469 (12)	
H67A	0.059846	0.981690	−0.119432	0.070*	0.5
H67B	0.144946	1.076060	−0.082482	0.070*	0.5
H67C	0.050716	1.037630	−0.046762	0.070*	0.5
H67D	0.045886	0.985100	−0.116102	0.070*	0.5
H67E	0.139036	1.076500	−0.087852	0.070*	0.5
H67F	0.063606	1.037290	−0.030382	0.070*	0.5
C68	0.0762 (3)	0.5530 (3)	0.7158 (2)	0.0499 (13)	
H68A	0.010813	0.529120	0.691859	0.075*	
H68B	0.088056	0.587280	0.767255	0.075*	
H68C	0.090669	0.503698	0.723869	0.075*	
C75	0.1016 (3)	0.2037 (3)	0.2673 (3)	0.0473 (12)	
H75A	0.076496	0.148649	0.230131	0.057*	
H75B	0.056836	0.193699	0.303961	0.057*	
C76	0.1918 (3)	0.2166 (3)	0.3164 (3)	0.0440 (12)	
H76A	0.237510	0.227415	0.280549	0.053*	
H76B	0.216390	0.270365	0.355089	0.053*	
O1	0.5212 (7)	0.9382 (8)	0.6548 (6)	0.030 (2)	0.5
C1	0.2209 (6)	0.7022 (5)	0.3565 (5)	0.0169 (11)*	0.5
C2	0.1481 (5)	0.6589 (6)	0.2929 (5)	0.0171 (11)*	0.5
H2	0.132963	0.600682	0.271090	0.020*	0.5
C3	0.0975 (5)	0.7001 (5)	0.2611 (4)	0.0143 (18)*	0.5
C4	0.1153 (5)	0.7841 (5)	0.2928 (4)	0.0179 (16)*	0.5
C5	0.1847 (5)	0.8282 (5)	0.3568 (4)	0.0170 (16)*	0.5
H5	0.197133	0.885457	0.379083	0.020*	0.5
C6	0.2368 (5)	0.7866 (5)	0.3899 (4)	0.0146 (19)*	0.5
C12	0.0208 (5)	0.6692 (5)	0.1928 (4)	0.0177 (19)*	0.5
C13	−0.0233 (6)	0.5908 (6)	0.1417 (5)	0.0215 (12)*	0.5
H13	−0.005164	0.545589	0.148270	0.026*	0.5
C14	−0.0937 (6)	0.5791 (6)	0.0814 (5)	0.0220 (12)*	0.5
H14	−0.126014	0.524478	0.047708	0.026*	0.5
C15	−0.1180 (6)	0.6469 (5)	0.0693 (5)	0.025 (2)*	0.5
H15	−0.170229	0.635552	0.030328	0.030*	0.5
C16	−0.0749 (5)	0.7244 (5)	0.1199 (4)	0.0204 (17)*	0.5
H16	−0.092159	0.770173	0.112675	0.025*	0.5
C17	−0.0054 (5)	0.7359 (5)	0.1821 (4)	0.0196 (16)*	0.5
C18	0.0503 (5)	0.8155 (5)	0.2457 (5)	0.0155 (16)	0.5
C55	0.5425 (8)	1.0169 (9)	0.7041 (7)	0.029 (3)	0.5
H55A	0.569555	1.068714	0.674356	0.043*	0.5
H55B	0.586821	1.023926	0.751723	0.043*	0.5
H55C	0.485599	1.012279	0.719937	0.043*	0.5
C56	−0.0105 (6)	0.8302 (6)	0.2995 (5)	0.0164 (18)	0.5
H56A	−0.046493	0.855852	0.268975	0.020*	0.5
H56B	0.030757	0.874492	0.345076	0.020*	0.5
C57	−0.0772 (5)	0.7467 (5)	0.3321 (5)	0.0207 (16)	0.5
H57A	−0.125993	0.706548	0.287690	0.025*	0.5
H57B	−0.043343	0.714948	0.354620	0.025*	0.5
C58	−0.1226 (5)	0.7682 (5)	0.3964 (4)	0.0243 (16)	0.5
H58A	−0.072599	0.813072	0.437373	0.029*	0.5
H58B	−0.159139	0.796432	0.371733	0.029*	0.5
C59	−0.1837 (8)	0.6938 (7)	0.4382 (6)	0.033 (2)	0.5
H59A	−0.148403	0.664144	0.462273	0.039*	0.5
H59B	−0.202253	0.718134	0.482033	0.039*	0.5
C60	−0.2756 (6)	0.6198 (5)	0.3798 (4)	0.0272 (17)	0.5
H60A	−0.294383	0.650373	0.338183	0.033*	0.5
H60B	−0.326133	0.594073	0.411543	0.033*	0.5
C61	−0.2663 (5)	0.5447 (5)	0.3399 (4)	0.0271 (17)	0.5
H61A	−0.253114	0.510742	0.380440	0.041*	0.5
H61B	−0.215474	0.569153	0.309228	0.041*	0.5
H61C	−0.324225	0.505468	0.303913	0.041*	0.5
C62	0.1071 (5)	0.9027 (6)	0.2087 (5)	0.0211 (17)	0.5
H62A	0.144851	0.950717	0.252725	0.025*	0.5
H62B	0.063441	0.919887	0.177735	0.025*	0.5
C63	0.1717 (6)	0.8980 (6)	0.1551 (6)	0.026 (2)	0.5
H63A	0.210984	0.874685	0.184062	0.031*	0.5
H63B	0.133554	0.854905	0.107832	0.031*	0.5
C64	0.2333 (5)	0.9862 (5)	0.1264 (5)	0.0309 (18)	0.5
H64A	0.269725	1.029477	0.174036	0.037*	0.5
H64B	0.277655	0.977667	0.097966	0.037*	0.5
C65	0.1846 (6)	1.0276 (6)	0.0722 (5)	0.031 (2)	0.5
H65A	0.231777	1.086562	0.061309	0.037*	0.5
H65B	0.141057	1.037312	0.100839	0.037*	0.5
C66	0.1328 (13)	0.9721 (13)	−0.0070 (7)	0.037 (3)	0.5
H66A	0.173953	0.954937	−0.032029	0.044*	0.5
H66B	0.079943	0.916637	0.003621	0.044*	0.5
C69	−0.0006 (6)	0.2390 (6)	0.1885 (6)	0.0221 (16)	0.5
H69A	−0.033745	0.235853	0.233445	0.026*	0.5
H69B	−0.025685	0.178243	0.160815	0.026*	0.5
C70	−0.0110 (8)	0.3013 (7)	0.1311 (7)	0.026 (2)	0.5
H70A	0.019842	0.362440	0.159028	0.031*	0.5
H70B	0.021482	0.301800	0.086078	0.031*	0.5
C71	−0.1113 (5)	0.2761 (6)	0.0978 (5)	0.0272 (18)	0.5
H71A	−0.144306	0.212378	0.076447	0.033*	0.5
H71B	−0.141306	0.283668	0.141947	0.033*	0.5
C72	−0.1224 (9)	0.3302 (7)	0.0331 (9)	0.030 (3)	0.5
H72A	−0.084753	0.394031	0.053396	0.036*	0.5
H72B	−0.096263	0.318871	−0.012414	0.036*	0.5
C73	−0.2200 (5)	0.3136 (6)	0.0028 (5)	0.036 (2)	0.5
H73A	−0.260510	0.248566	−0.008842	0.044*	0.5
H73B	−0.242470	0.335676	0.045218	0.044*	0.5
C74	−0.2285 (11)	0.3576 (9)	−0.0708 (8)	0.038 (3)	0.5
H74A	−0.185485	0.421548	−0.060777	0.057*	0.5
H74B	−0.292076	0.348574	−0.084530	0.057*	0.5
H74C	−0.213449	0.331304	−0.115111	0.057*	0.5
O1B	0.5424 (7)	0.9298 (8)	0.6356 (6)	0.025 (2)	0.5
C1B	0.2381 (6)	0.7177 (5)	0.3512 (5)	0.0169 (11)*	0.5
C2B	0.1680 (5)	0.6738 (6)	0.2846 (5)	0.0171 (11)*	0.5
H2B	0.152520	0.614587	0.265781	0.020*	0.5
C3B	0.1205 (5)	0.7151 (5)	0.2456 (5)	0.020 (2)*	0.5
C4B	0.1434 (5)	0.8031 (5)	0.2701 (4)	0.0184 (16)*	0.5
C5B	0.2109 (5)	0.8491 (5)	0.3350 (4)	0.0177 (16)*	0.5
H5B	0.226039	0.908583	0.352689	0.021*	0.5
C6B	0.2578 (5)	0.8070 (5)	0.3752 (4)	0.0160 (19)*	0.5
C12B	0.0443 (5)	0.6823 (5)	0.1773 (4)	0.0175 (19)*	0.5
C13B	−0.0040 (6)	0.5995 (6)	0.1330 (5)	0.0215 (12)*	0.5
H13B	0.010432	0.552751	0.145305	0.026*	0.5
C14B	−0.0738 (6)	0.5861 (6)	0.0706 (5)	0.0220 (12)*	0.5
H14B	−0.107655	0.529471	0.040006	0.026*	0.5
C15B	−0.0950 (6)	0.6540 (5)	0.0521 (5)	0.025 (2)*	0.5
H15B	−0.136999	0.645873	0.004675	0.030*	0.5
C16B	−0.0460 (5)	0.7378 (5)	0.0956 (4)	0.0230 (18)*	0.5
H16B	−0.060562	0.784464	0.083121	0.028*	0.5
C17B	0.0236 (5)	0.7511 (5)	0.1579 (4)	0.0205 (17)*	0.5
C18B	0.0841 (6)	0.8351 (6)	0.2160 (5)	0.0178 (16)	0.5
C55B	0.5691 (9)	1.0092 (10)	0.6860 (8)	0.033 (3)	0.5
H55D	0.578707	1.058122	0.653395	0.050*	0.5
H55E	0.626794	1.023909	0.722320	0.050*	0.5
H55F	0.520208	1.000070	0.717056	0.050*	0.5
C56B	0.0236 (5)	0.8604 (5)	0.2619 (5)	0.0220 (16)	0.5
H56C	−0.007242	0.886308	0.225153	0.026*	0.5
H56D	0.064988	0.907698	0.305463	0.026*	0.5
C57B	−0.0505 (7)	0.7856 (7)	0.2983 (6)	0.027 (2)	0.5
H57C	−0.019962	0.762480	0.338476	0.033*	0.5
H57D	−0.089972	0.736280	0.255766	0.033*	0.5
C58B	−0.1111 (5)	0.8154 (6)	0.3369 (5)	0.0355 (19)	0.5
H58C	−0.076800	0.847879	0.389922	0.043*	0.5
H58D	−0.122650	0.857489	0.304392	0.043*	0.5
C59B	−0.2033 (6)	0.7399 (6)	0.3489 (6)	0.047 (2)	0.5
H59C	−0.237676	0.708910	0.295467	0.057*	0.5
H59D	−0.238896	0.766730	0.370417	0.057*	0.5
C60B	−0.2043 (8)	0.6693 (8)	0.4012 (6)	0.041 (2)	0.5
H60C	−0.160963	0.648931	0.385238	0.049*	0.5
H60D	−0.267022	0.617431	0.391178	0.049*	0.5
C61B	−0.1804 (9)	0.6953 (9)	0.4853 (6)	0.073 (4)	0.5
H61D	−0.117047	0.744537	0.497883	0.110*	0.5
H61E	−0.184227	0.644638	0.513323	0.110*	0.5
H61F	−0.223434	0.714699	0.502326	0.110*	0.5
C62B	0.1432 (6)	0.9140 (6)	0.1734 (5)	0.0202 (18)	0.5
H62C	0.102303	0.935198	0.144108	0.024*	0.5
H62D	0.188153	0.962828	0.214268	0.024*	0.5
C63B	0.1968 (6)	0.8964 (5)	0.1156 (5)	0.0254 (18)	0.5
H63C	0.234716	0.871229	0.143561	0.030*	0.5
H63D	0.151956	0.851099	0.071991	0.030*	0.5
C64B	0.2598 (5)	0.9790 (5)	0.0780 (5)	0.0308 (18)	0.5
H64C	0.289624	0.961161	0.039218	0.037*	0.5
H64D	0.309514	1.021191	0.120848	0.037*	0.5
C65B	0.2108 (6)	1.0274 (6)	0.0359 (5)	0.0289 (19)	0.5
H65C	0.257721	1.082126	0.017999	0.035*	0.5
H65D	0.181690	1.045795	0.074859	0.035*	0.5
C66B	0.1374 (11)	0.9732 (12)	−0.0360 (6)	0.033 (3)	0.5
H66C	0.166512	0.956997	−0.076448	0.040*	0.5
H66D	0.090461	0.917377	−0.019475	0.040*	0.5
C69B	0.0147 (7)	0.2678 (6)	0.1625 (6)	0.0221 (16)	0.5
H69C	−0.034115	0.248401	0.196286	0.026*	0.5
H69D	−0.003135	0.216261	0.122856	0.026*	0.5
C70B	0.0043 (8)	0.3373 (7)	0.1153 (7)	0.025 (2)	0.5
H70C	0.002530	0.382285	0.152440	0.030*	0.5
H70D	0.058910	0.367905	0.088820	0.030*	0.5
C71B	−0.0838 (6)	0.2970 (6)	0.0528 (6)	0.034 (2)	0.5
H71C	−0.135504	0.253261	0.076851	0.041*	0.5
H71D	−0.074584	0.264181	0.008681	0.041*	0.5
C72B	−0.1113 (9)	0.3634 (7)	0.0184 (8)	0.029 (3)	0.5
H72C	−0.129388	0.390013	0.060897	0.035*	0.5
H72D	−0.056838	0.411873	0.001107	0.035*	0.5
C73B	−0.1905 (6)	0.3241 (6)	−0.0518 (6)	0.040 (2)	0.5
H73C	−0.173227	0.295604	−0.093581	0.048*	0.5
H73D	−0.245497	0.276924	−0.033991	0.048*	0.5
C74B	−0.2172 (11)	0.3911 (10)	−0.0890 (10)	0.051 (4)	0.5
H74D	−0.163553	0.437699	−0.107678	0.077*	0.5
H74E	−0.237310	0.418021	−0.049181	0.077*	0.5
H74F	−0.267920	0.360532	−0.134245	0.077*	0.5
C77B	0.1690 (6)	0.1365 (6)	0.3585 (6)	0.0401 (15)*	0.5
H77A	0.123784	0.129850	0.393728	0.048*	0.5
H77B	0.142844	0.082120	0.320328	0.048*	0.5
C78B	0.2593 (5)	0.1520 (5)	0.4077 (5)	0.0308 (18)	0.5
H78A	0.300010	0.143052	0.374782	0.037*	0.5
H78B	0.291960	0.214492	0.432702	0.037*	0.5
C79B	0.2398 (5)	0.0900 (5)	0.4717 (4)	0.0275 (17)	0.5
H79A	0.211373	0.027844	0.446620	0.033*	0.5
H79B	0.194793	0.095774	0.501250	0.033*	0.5
C80B	0.3265 (5)	0.1095 (5)	0.5296 (4)	0.0283 (17)	0.5
H80A	0.310832	0.067892	0.570280	0.042*	0.5
H80B	0.354182	0.170702	0.555290	0.042*	0.5
H80C	0.370772	0.102762	0.500630	0.042*	0.5
O3B	0.4563 (4)	0.5453 (5)	0.6547 (3)	0.0266 (14)	0.5
N3B	0.4196 (6)	0.6481 (7)	0.4829 (4)	0.0153 (17)	0.5
C37B	0.4226 (4)	0.7280 (5)	0.3517 (4)	0.0203 (12)*	0.5
C38B	0.4236 (5)	0.7644 (5)	0.2778 (4)	0.0161 (11)*	0.5
H38B	0.368830	0.764924	0.252550	0.019*	0.5
C39B	0.5013 (6)	0.7967 (5)	0.2408 (4)	0.0154 (16)	0.5
C40B	0.5830 (5)	0.7975 (5)	0.2773 (4)	0.0216 (15)	0.5
C41B	0.5878 (4)	0.7664 (5)	0.3501 (4)	0.0213 (15)	0.5
H41B	0.643577	0.767955	0.375566	0.026*	0.5
C42B	0.5086 (4)	0.7313 (5)	0.3860 (4)	0.0178 (15)	0.5
C43B	0.5044 (4)	0.6867 (5)	0.4584 (4)	0.0186 (15)	0.5
C44B	0.5790 (5)	0.6809 (5)	0.5045 (4)	0.0242 (16)	0.5
H44B	0.638532	0.710291	0.489759	0.029*	0.5
C45B	0.5672 (5)	0.6343 (5)	0.5697 (4)	0.0242 (16)	0.5
H45B	0.617500	0.629227	0.599087	0.029*	0.5
C46B	0.4804 (5)	0.5942 (5)	0.5919 (5)	0.0195 (17)	0.5
C47B	0.4092 (9)	0.6039 (11)	0.5468 (6)	0.015 (2)	0.5
H47B	0.350230	0.578082	0.563000	0.018*	0.5
C48B	0.5136 (5)	0.8269 (5)	0.1618 (4)	0.0202 (15)	0.5
C49B	0.4543 (5)	0.8379 (5)	0.1032 (5)	0.0270 (18)	0.5
H49B	0.395309	0.828774	0.112526	0.032*	0.5
C50B	0.4822 (6)	0.8619 (5)	0.0297 (5)	0.0315 (18)	0.5
H50B	0.440859	0.867579	−0.011559	0.038*	0.5
C51B	0.5689 (6)	0.8771 (6)	0.0171 (5)	0.029 (2)	0.5
H51B	0.586879	0.893595	−0.033006	0.034*	0.5
C52B	0.6305 (5)	0.8698 (5)	0.0765 (4)	0.0278 (16)	0.5
H52B	0.690364	0.881281	0.067503	0.033*	0.5
C53B	0.6020 (6)	0.8443 (5)	0.1496 (5)	0.0208 (17)	0.5
C54B	0.6573 (5)	0.8318 (6)	0.2229 (4)	0.0251 (17)	0.5
C81B	0.5305 (5)	0.5406 (5)	0.7067 (5)	0.0302 (18)	0.5
H81A	0.561443	0.516516	0.675463	0.045*	0.5
H81B	0.505113	0.501641	0.747820	0.045*	0.5
H81C	0.575410	0.600399	0.732060	0.045*	0.5
C82B	0.7418 (5)	0.9207 (6)	0.2596 (5)	0.0337 (19)	0.5
H82A	0.789131	0.935908	0.224666	0.040*	0.5
H82B	0.768811	0.911908	0.312006	0.040*	0.5
C83B	0.7246 (6)	1.0012 (6)	0.2705 (7)	0.060 (3)	0.5
H83A	0.719013	1.024677	0.218771	0.072*	0.5
H83B	0.665563	0.982337	0.290781	0.072*	0.5
C84B	0.8105 (8)	1.0763 (6)	0.3214 (6)	0.0469 (17)*	0.5
H84A	0.826644	1.050521	0.368550	0.056*	0.5
H84B	0.861453	1.093154	0.290279	0.056*	0.5
C85B	0.8124 (6)	1.1531 (6)	0.3490 (6)	0.062 (2)	0.5
H85A	0.759439	1.137729	0.377472	0.075*	0.5
H85B	0.803096	1.183469	0.302657	0.075*	0.5
C86B	0.9014 (6)	1.2189 (7)	0.4050 (6)	0.065 (4)	0.5
H86A	0.952000	1.206470	0.393914	0.077*	0.5
H86B	0.917007	1.279617	0.390707	0.077*	0.5
C87B	0.9015 (7)	1.2201 (7)	0.4931 (6)	0.051 (3)*	0.5
H87A	0.962267	1.264918	0.521503	0.077*	0.5
H87B	0.853601	1.234770	0.506076	0.077*	0.5
H87C	0.888757	1.161284	0.509298	0.077*	0.5
C88B	0.6922 (4)	0.7651 (4)	0.2036 (4)	0.0244 (15)	0.5
H88A	0.732655	0.788386	0.162970	0.029*	0.5
H88B	0.731412	0.763530	0.252368	0.029*	0.5
C89B	0.6200 (4)	0.6700 (4)	0.1734 (4)	0.0250 (16)	0.5
H89A	0.584336	0.642911	0.216253	0.030*	0.5
H89B	0.576196	0.670753	0.127859	0.030*	0.5
C90B	0.6619 (5)	0.6128 (4)	0.1472 (4)	0.0266 (16)	0.5
H90A	0.704821	0.611408	0.193171	0.032*	0.5
H90B	0.698829	0.640972	0.105372	0.032*	0.5
C91B	0.5911 (6)	0.5174 (5)	0.1149 (5)	0.0317 (19)	0.5
H91A	0.546491	0.519429	0.070865	0.038*	0.5
H91B	0.555992	0.489037	0.157768	0.038*	0.5
C92B	0.6295 (6)	0.4570 (6)	0.0840 (7)	0.036 (3)*	0.5
H92A	0.640372	0.421610	0.125230	0.043*	0.5
H92B	0.686523	0.490678	0.061281	0.043*	0.5
C93B	0.5381 (6)	0.3960 (7)	0.0148 (6)	0.051 (3)*	0.5
H93A	0.548316	0.351049	−0.013533	0.076*	0.5
H93B	0.483131	0.366264	0.040056	0.076*	0.5
H93C	0.528639	0.434370	−0.023004	0.076*	0.5
O3	0.4887 (4)	0.5455 (5)	0.6272 (4)	0.0284 (15)	0.5
N3	0.4178 (6)	0.6324 (7)	0.4594 (4)	0.0153 (17)	0.5
C37	0.3989 (5)	0.7153 (5)	0.3322 (4)	0.0203 (12)*	0.5
C38	0.3904 (5)	0.7632 (5)	0.2700 (4)	0.0161 (11)*	0.5
H38	0.341517	0.778631	0.263000	0.019*	0.5
C39	0.4543 (6)	0.7897 (5)	0.2188 (4)	0.0209 (17)	0.5
C40	0.5260 (5)	0.7670 (5)	0.2275 (4)	0.0204 (16)	0.5
C41	0.5384 (4)	0.7209 (5)	0.2894 (4)	0.0208 (15)	0.5
H41	0.587333	0.705502	0.295040	0.025*	0.5
C42	0.4747 (5)	0.6960 (5)	0.3431 (4)	0.0173 (14)	0.5
C43	0.4854 (5)	0.6533 (5)	0.4129 (4)	0.0181 (14)	0.5
C44	0.5582 (5)	0.6344 (4)	0.4404 (4)	0.0239 (16)	0.5
H44	0.605667	0.647729	0.409205	0.029*	0.5
C45	0.5635 (4)	0.5983 (5)	0.5103 (4)	0.0241 (16)	0.5
H45	0.613042	0.585522	0.527217	0.029*	0.5
C46	0.4947 (5)	0.5799 (5)	0.5561 (5)	0.0218 (17)	0.5
C47	0.4214 (8)	0.5972 (11)	0.5268 (6)	0.015 (2)	0.5
H47	0.372680	0.583088	0.556641	0.018*	0.5
C48	0.4614 (6)	0.8414 (5)	0.1507 (5)	0.0233 (16)	0.5
C49	0.4061 (5)	0.8780 (5)	0.1178 (4)	0.0266 (16)	0.5
H49	0.353932	0.871422	0.140212	0.032*	0.5
C50	0.4281 (5)	0.9235 (5)	0.0512 (4)	0.0342 (19)	0.5
H50	0.391705	0.949851	0.028882	0.041*	0.5
C51	0.5024 (6)	0.9311 (6)	0.0174 (5)	0.039 (2)	0.5
H51	0.514559	0.960452	−0.029407	0.047*	0.5
C52	0.5591 (7)	0.8965 (6)	0.0502 (5)	0.033 (2)	0.5
H52	0.611670	0.904167	0.027881	0.039*	0.5
C53	0.5369 (5)	0.8498 (5)	0.1175 (4)	0.0264 (16)	0.5
C54	0.5846 (6)	0.8017 (5)	0.1604 (5)	0.0263 (19)	0.5
C77	0.1921 (7)	0.1391 (6)	0.3640 (6)	0.0401 (15)*	0.5
H77C	0.253618	0.162721	0.398219	0.048*	0.5
H77D	0.187368	0.092651	0.324339	0.048*	0.5
C78	0.1176 (5)	0.0914 (5)	0.4170 (4)	0.0337 (19)	0.5
H78C	0.115246	0.138561	0.450828	0.040*	0.5
H78D	0.055446	0.057902	0.383688	0.040*	0.5
C79	0.1253 (6)	0.0283 (5)	0.4738 (5)	0.038 (2)	0.5
H79C	0.126868	−0.021226	0.443002	0.045*	0.5
H79D	0.184598	0.059944	0.511462	0.045*	0.5
C80	0.0469 (6)	−0.0102 (5)	0.5218 (5)	0.044 (2)	0.5
H80D	0.056426	−0.050667	0.557161	0.067*	0.5
H80E	−0.012194	−0.043207	0.485261	0.067*	0.5
H80F	0.045656	0.038103	0.553821	0.067*	0.5
C81	0.5628 (5)	0.5273 (6)	0.6592 (5)	0.0313 (19)	0.5
H81D	0.571389	0.490655	0.618836	0.047*	0.5
H81E	0.547522	0.495383	0.706319	0.047*	0.5
H81F	0.620121	0.583582	0.674322	0.047*	0.5
C82	0.6861 (6)	0.8635 (5)	0.1949 (5)	0.0267 (18)	0.5
H82C	0.721792	0.880255	0.150456	0.032*	0.5
H82D	0.710102	0.830445	0.229276	0.032*	0.5
C83	0.7042 (6)	0.9493 (6)	0.2439 (5)	0.0306 (19)	0.5
H83C	0.655252	0.933809	0.277212	0.037*	0.5
H83D	0.697772	0.990339	0.206622	0.037*	0.5
C84	0.7963 (5)	0.9979 (5)	0.2971 (5)	0.0322 (18)	0.5
H84C	0.845698	1.009560	0.265094	0.039*	0.5
H84D	0.800868	0.959530	0.338174	0.039*	0.5
C85	0.8107 (8)	1.0832 (7)	0.3467 (6)	0.0469 (17)*	0.5
H85C	0.817687	1.128705	0.309655	0.056*	0.5
H85D	0.753272	1.068576	0.368507	0.056*	0.5
C86	0.8878 (6)	1.1235 (7)	0.4122 (6)	0.062 (2)	0.5
H86C	0.947447	1.133222	0.397195	0.075*	0.5
H86D	0.873237	1.080411	0.452375	0.075*	0.5
C87	0.8989 (7)	1.2109 (6)	0.4505 (6)	0.033 (2)*	0.5
H87D	0.948489	1.232209	0.497038	0.050*	0.5
H87E	0.841098	1.202179	0.467408	0.050*	0.5
H87F	0.915488	1.255119	0.412038	0.050*	0.5
C88	0.5749 (5)	0.7239 (5)	0.1035 (5)	0.0364 (19)	0.5
H88C	0.597295	0.687537	0.134619	0.044*	0.5
H88D	0.615643	0.749069	0.063407	0.044*	0.5
C89	0.4748 (6)	0.6618 (6)	0.0588 (5)	0.059 (3)	0.5
H89C	0.435865	0.686623	0.077068	0.071*	0.5
H89D	0.455698	0.604103	0.081208	0.071*	0.5
C90	0.4392 (7)	0.6358 (6)	−0.0397 (5)	0.053 (3)	0.5
H90C	0.421884	0.680460	−0.061158	0.064*	0.5
H90D	0.491393	0.639404	−0.064770	0.064*	0.5
C91	0.3645 (8)	0.5518 (8)	−0.0610 (7)	0.059 (4)*	0.5
H91C	0.306475	0.556601	−0.065533	0.071*	0.5
H91D	0.364286	0.513718	−0.018242	0.071*	0.5
C92	0.3659 (8)	0.5083 (8)	−0.1374 (6)	0.075 (4)	0.5
H92C	0.382291	0.460083	−0.126286	0.090*	0.5
H92D	0.414164	0.552478	−0.164243	0.090*	0.5
C93	0.2739 (7)	0.4699 (8)	−0.1920 (6)	0.088 (4)	0.5
H93D	0.277605	0.441860	−0.241621	0.132*	0.5
H93E	0.226155	0.425312	−0.165960	0.132*	0.5
H93F	0.258014	0.517666	−0.203900	0.132*	0.5
C1S	0.5936 (4)	0.7728 (5)	0.7767 (4)	0.109 (3)	
H1SA	0.614669	0.740856	0.742590	0.164*	0.5
H1SB	0.627082	0.836747	0.772014	0.164*	0.5
H1SC	0.605520	0.762116	0.832268	0.164*	0.5
H1SD	0.648018	0.821263	0.810209	0.164*	0.5
H1SE	0.585660	0.715442	0.793472	0.164*	0.5
H1SF	0.602180	0.775334	0.720862	0.164*	0.5
C2S	0.4895 (6)	0.7393 (8)	0.7505 (7)	0.082 (3)*	0.5
H2SA	0.455162	0.674596	0.754765	0.098*	0.5
H2SB	0.476792	0.749459	0.694323	0.098*	0.5
C3S	0.4571 (8)	0.7912 (8)	0.8059 (7)	0.085 (3)*	0.5
H3SA	0.481237	0.790797	0.862303	0.102*	0.5
H3SB	0.483162	0.853996	0.794146	0.102*	0.5
C4S	0.3505 (8)	0.7492 (8)	0.7937 (9)	0.091 (3)*	0.5
H4SA	0.323615	0.710164	0.835075	0.109*	0.5
H4SB	0.320263	0.716914	0.739857	0.109*	0.5
C5S	0.3494 (10)	0.8403 (8)	0.8045 (9)	0.113 (5)*	0.5
H5SA	0.285182	0.829826	0.798957	0.170*	0.5
H5SB	0.382827	0.870760	0.857631	0.170*	0.5
H5SC	0.379525	0.877409	0.763835	0.170*	0.5
C2S'	0.5081 (6)	0.7825 (8)	0.7854 (8)	0.082 (3)*	0.5
H2SC	0.508701	0.784907	0.843616	0.098*	0.5
H2SD	0.524942	0.843787	0.772231	0.098*	0.5
C3S'	0.4089 (7)	0.7323 (8)	0.7516 (7)	0.085 (3)*	0.5
H3SC	0.383172	0.674290	0.773778	0.102*	0.5
H3SD	0.403005	0.719763	0.693299	0.102*	0.5
C4S'	0.3490 (9)	0.7767 (9)	0.7658 (7)	0.091 (3)*	0.5
H4SC	0.296460	0.752759	0.720705	0.109*	0.5
H4SD	0.386565	0.840719	0.760183	0.109*	0.5
C5S'	0.3070 (11)	0.7726 (12)	0.8421 (8)	0.131 (6)*	0.5
H5SD	0.271446	0.805677	0.837885	0.196*	0.5
H5SE	0.265961	0.710282	0.848592	0.196*	0.5
H5SF	0.356891	0.799047	0.888432	0.196*	0.5

### \ Tris[9,9-dihexyl-2-(5-methoxypyridin-2-yl-κN)-9H-fluoren-3-\ yl-κC3]iridium pentane monosolvate (I) Atomic displacement parameters (Å^2^)

**Table:**

	*U*^11^	*U*^22^	*U*^33^	*U*^12^	*U*^13^	*U*^23^
Ir	0.01876 (7)	0.02774 (8)	0.01489 (7)	0.01543 (6)	0.00330 (5)	0.00433 (5)
O2	0.0472 (18)	0.0385 (17)	0.0263 (15)	0.0056 (15)	0.0214 (13)	−0.0042 (13)
N1	0.0156 (15)	0.0277 (18)	0.0157 (15)	0.0092 (14)	0.0029 (12)	0.0063 (13)
N2	0.0195 (15)	0.0244 (16)	0.0123 (14)	0.0139 (14)	0.0049 (12)	0.0003 (12)
C7	0.0248 (19)	0.039 (2)	0.0167 (18)	0.0224 (19)	−0.0030 (15)	−0.0062 (16)
C8	0.028 (2)	0.045 (3)	0.033 (2)	0.026 (2)	−0.0075 (17)	−0.0136 (19)
C9	0.023 (2)	0.032 (2)	0.0201 (19)	0.0065 (18)	0.0027 (15)	−0.0057 (16)
C10	0.020 (2)	0.030 (2)	0.024 (2)	−0.0027 (18)	−0.0071 (15)	0.0046 (16)
C11	0.0175 (19)	0.026 (2)	0.028 (2)	0.0024 (17)	−0.0027 (15)	0.0098 (16)
C19	0.0201 (18)	0.035 (2)	0.0184 (18)	0.0224 (18)	−0.0021 (14)	−0.0003 (16)
C20	0.028 (2)	0.046 (2)	0.0166 (18)	0.0281 (19)	−0.0011 (15)	0.0010 (17)
C21	0.025 (2)	0.055 (3)	0.0185 (19)	0.029 (2)	−0.0100 (15)	−0.0135 (18)
C22	0.020 (2)	0.053 (3)	0.037 (2)	0.020 (2)	−0.0084 (17)	−0.026 (2)
C23	0.0130 (18)	0.045 (3)	0.041 (2)	0.0080 (19)	0.0016 (17)	−0.020 (2)
C24	0.0152 (17)	0.034 (2)	0.0220 (19)	0.0139 (17)	−0.0037 (14)	−0.0100 (16)
C25	0.0172 (17)	0.0212 (19)	0.0207 (18)	0.0117 (16)	−0.0020 (14)	−0.0039 (14)
C26	0.0211 (19)	0.023 (2)	0.028 (2)	0.0114 (17)	0.0007 (15)	−0.0008 (16)
C27	0.024 (2)	0.024 (2)	0.0231 (19)	0.0107 (17)	0.0070 (16)	0.0076 (16)
C28	0.029 (2)	0.027 (2)	0.0169 (18)	0.0143 (18)	0.0054 (15)	0.0028 (15)
C29	0.0207 (18)	0.022 (2)	0.0182 (18)	0.0115 (16)	−0.0001 (14)	−0.0012 (15)
C30	0.038 (2)	0.062 (3)	0.0181 (19)	0.043 (2)	−0.0084 (16)	−0.0099 (18)
C31	0.057 (3)	0.062 (3)	0.019 (2)	0.050 (2)	0.0031 (18)	0.0022 (19)
C32	0.084 (3)	0.069 (3)	0.020 (2)	0.066 (3)	0.016 (2)	0.013 (2)
C33	0.088 (4)	0.074 (3)	0.021 (2)	0.074 (3)	0.003 (2)	−0.001 (2)
C34	0.054 (3)	0.064 (3)	0.025 (2)	0.046 (3)	−0.0112 (19)	−0.017 (2)
C35	0.036 (2)	0.064 (3)	0.024 (2)	0.038 (2)	−0.0114 (17)	−0.0190 (19)
C36	0.023 (2)	0.058 (3)	0.047 (3)	0.022 (2)	−0.0090 (18)	−0.035 (2)
C67	0.038 (3)	0.051 (3)	0.045 (3)	0.015 (2)	0.008 (2)	0.012 (2)
C68	0.051 (3)	0.060 (3)	0.025 (2)	0.009 (3)	0.023 (2)	0.002 (2)
C75	0.022 (2)	0.050 (3)	0.053 (3)	0.007 (2)	0.0010 (19)	−0.032 (2)
C76	0.025 (2)	0.035 (3)	0.066 (3)	0.011 (2)	0.008 (2)	−0.019 (2)
O1	0.032 (5)	0.027 (4)	0.028 (5)	0.016 (4)	−0.009 (3)	−0.011 (3)
C18	0.013 (4)	0.015 (4)	0.019 (4)	0.008 (3)	0.000 (3)	−0.001 (3)
C55	0.029 (6)	0.022 (5)	0.023 (6)	0.007 (5)	−0.012 (4)	−0.011 (4)
C56	0.015 (5)	0.016 (5)	0.020 (4)	0.009 (4)	0.002 (3)	0.004 (4)
C57	0.015 (4)	0.017 (4)	0.028 (4)	0.006 (3)	0.002 (3)	0.004 (3)
C58	0.026 (4)	0.026 (4)	0.019 (4)	0.012 (3)	−0.001 (3)	−0.004 (3)
C59	0.055 (6)	0.040 (5)	0.009 (5)	0.030 (4)	−0.002 (5)	−0.002 (4)
C60	0.030 (4)	0.025 (4)	0.024 (4)	0.011 (4)	0.005 (3)	0.004 (3)
C61	0.028 (4)	0.022 (4)	0.031 (4)	0.010 (4)	0.006 (3)	0.008 (3)
C62	0.015 (4)	0.024 (5)	0.026 (4)	0.009 (4)	0.007 (3)	0.009 (4)
C63	0.027 (5)	0.026 (5)	0.036 (6)	0.017 (4)	0.016 (4)	0.009 (4)
C64	0.025 (4)	0.032 (5)	0.037 (5)	0.011 (4)	0.011 (4)	0.014 (4)
C65	0.037 (5)	0.023 (5)	0.029 (5)	0.010 (4)	0.012 (4)	0.014 (4)
C66	0.045 (7)	0.037 (6)	0.035 (6)	0.025 (6)	0.012 (6)	0.005 (6)
C69	0.015 (3)	0.007 (5)	0.036 (5)	0.000 (3)	0.002 (3)	−0.006 (3)
C70	0.020 (5)	0.023 (7)	0.031 (6)	0.008 (5)	0.000 (4)	−0.002 (4)
C71	0.024 (4)	0.031 (5)	0.025 (4)	0.013 (4)	−0.001 (3)	−0.007 (4)
C72	0.023 (5)	0.020 (7)	0.041 (6)	0.009 (5)	−0.004 (4)	−0.003 (5)
C73	0.027 (4)	0.048 (6)	0.034 (5)	0.019 (4)	0.000 (4)	0.000 (4)
C74	0.033 (6)	0.032 (8)	0.033 (6)	0.008 (6)	−0.015 (5)	−0.005 (5)
O1B	0.026 (4)	0.024 (4)	0.021 (4)	0.011 (3)	−0.009 (3)	−0.008 (3)
C18B	0.017 (4)	0.018 (5)	0.020 (4)	0.010 (4)	−0.001 (3)	0.003 (3)
C55B	0.033 (7)	0.026 (6)	0.033 (7)	0.009 (5)	−0.003 (5)	−0.008 (4)
C56B	0.028 (4)	0.029 (5)	0.015 (4)	0.019 (4)	−0.001 (3)	−0.001 (3)
C57B	0.025 (6)	0.027 (6)	0.033 (5)	0.015 (5)	0.006 (4)	0.002 (5)
C58B	0.030 (4)	0.045 (5)	0.041 (5)	0.025 (4)	0.007 (4)	−0.002 (4)
C59B	0.055 (5)	0.055 (6)	0.060 (6)	0.041 (5)	0.038 (5)	0.027 (5)
C60B	0.046 (6)	0.049 (7)	0.038 (6)	0.032 (5)	0.008 (5)	0.010 (5)
C61B	0.112 (10)	0.115 (11)	0.037 (6)	0.094 (9)	0.003 (7)	0.006 (7)
C62B	0.021 (5)	0.020 (4)	0.022 (5)	0.012 (4)	−0.001 (3)	0.002 (4)
C63B	0.026 (4)	0.026 (4)	0.031 (5)	0.017 (4)	0.005 (3)	0.008 (4)
C64B	0.023 (4)	0.028 (4)	0.037 (5)	0.008 (4)	0.006 (3)	0.009 (4)
C65B	0.036 (5)	0.021 (5)	0.031 (5)	0.014 (4)	0.010 (4)	0.009 (4)
C66B	0.032 (5)	0.025 (6)	0.032 (7)	0.005 (4)	0.009 (5)	−0.004 (6)
C69B	0.015 (3)	0.007 (5)	0.036 (5)	0.000 (3)	0.002 (3)	−0.006 (3)
C70B	0.025 (5)	0.026 (7)	0.028 (5)	0.018 (5)	−0.001 (4)	−0.006 (4)
C71B	0.027 (5)	0.023 (5)	0.045 (6)	0.011 (4)	−0.012 (4)	−0.006 (4)
C72B	0.026 (5)	0.020 (7)	0.037 (6)	0.012 (5)	−0.008 (4)	−0.005 (5)
C73B	0.034 (5)	0.036 (5)	0.045 (6)	0.017 (5)	−0.012 (4)	−0.007 (4)
C74B	0.046 (8)	0.068 (12)	0.050 (9)	0.042 (9)	−0.008 (6)	−0.001 (6)
C78B	0.034 (5)	0.026 (4)	0.038 (5)	0.019 (4)	0.007 (4)	0.012 (3)
C79B	0.027 (4)	0.023 (4)	0.031 (4)	0.011 (4)	0.004 (3)	0.006 (3)
C80B	0.032 (4)	0.024 (4)	0.027 (4)	0.013 (4)	0.003 (3)	0.003 (3)
O3B	0.026 (4)	0.037 (4)	0.021 (3)	0.018 (3)	0.002 (2)	0.009 (3)
N3B	0.0166 (16)	0.016 (4)	0.014 (4)	0.009 (2)	0.001 (2)	−0.002 (3)
C39B	0.016 (4)	0.009 (4)	0.017 (4)	0.003 (4)	0.002 (3)	−0.004 (3)
C40B	0.015 (3)	0.023 (4)	0.021 (3)	0.005 (3)	0.004 (3)	0.000 (3)
C41B	0.013 (3)	0.028 (4)	0.022 (3)	0.010 (3)	−0.002 (3)	0.000 (3)
C42B	0.011 (3)	0.023 (4)	0.015 (3)	0.006 (3)	−0.001 (3)	−0.002 (3)
C43B	0.014 (3)	0.027 (4)	0.019 (4)	0.012 (3)	0.004 (3)	0.001 (3)
C44B	0.016 (3)	0.037 (5)	0.021 (4)	0.014 (3)	0.003 (3)	0.004 (3)
C45B	0.021 (4)	0.026 (4)	0.024 (4)	0.012 (4)	−0.006 (3)	0.000 (3)
C46B	0.023 (4)	0.019 (4)	0.015 (4)	0.010 (3)	−0.002 (3)	−0.003 (3)
C47B	0.017 (4)	0.016 (5)	0.012 (5)	0.009 (4)	0.000 (3)	−0.003 (4)
C48B	0.020 (4)	0.011 (4)	0.022 (4)	0.000 (3)	0.005 (3)	0.000 (3)
C49B	0.024 (4)	0.016 (4)	0.033 (4)	0.002 (4)	0.004 (4)	0.010 (4)
C50B	0.034 (5)	0.027 (5)	0.031 (4)	0.013 (4)	0.003 (4)	0.012 (4)
C51B	0.035 (4)	0.020 (5)	0.025 (5)	0.007 (4)	0.006 (4)	0.003 (4)
C52B	0.028 (4)	0.021 (4)	0.030 (4)	0.006 (4)	0.011 (3)	0.008 (3)
C53B	0.021 (4)	0.013 (4)	0.022 (3)	0.002 (4)	0.004 (3)	0.001 (3)
C54B	0.025 (4)	0.032 (5)	0.019 (4)	0.012 (4)	0.007 (3)	0.006 (3)
C81B	0.028 (4)	0.034 (5)	0.026 (4)	0.014 (4)	−0.004 (3)	0.006 (4)
C82B	0.014 (4)	0.040 (5)	0.035 (5)	0.003 (4)	0.008 (3)	−0.007 (4)
C83B	0.026 (5)	0.035 (6)	0.096 (9)	−0.002 (5)	0.009 (5)	−0.031 (6)
C85B	0.038 (4)	0.052 (5)	0.080 (5)	0.010 (4)	0.006 (3)	−0.021 (4)
C86B	0.030 (5)	0.062 (7)	0.063 (7)	−0.012 (5)	0.022 (5)	−0.039 (6)
C88B	0.017 (4)	0.035 (4)	0.020 (4)	0.011 (3)	0.004 (3)	0.005 (3)
C89B	0.017 (4)	0.030 (4)	0.022 (4)	0.007 (3)	0.000 (3)	0.003 (3)
C90B	0.026 (4)	0.032 (4)	0.024 (4)	0.014 (3)	0.009 (3)	0.007 (3)
C91B	0.035 (5)	0.039 (5)	0.027 (5)	0.022 (4)	0.005 (4)	0.008 (4)
O3	0.024 (4)	0.032 (4)	0.030 (4)	0.015 (3)	0.001 (3)	0.013 (3)
N3	0.0166 (16)	0.016 (4)	0.014 (4)	0.009 (2)	0.001 (2)	−0.002 (3)
C39	0.021 (4)	0.017 (4)	0.016 (4)	0.004 (4)	−0.004 (3)	−0.006 (3)
C40	0.017 (4)	0.012 (4)	0.026 (4)	0.000 (3)	0.007 (3)	0.001 (3)
C41	0.014 (3)	0.020 (4)	0.029 (4)	0.008 (3)	0.005 (3)	0.001 (3)
C42	0.012 (4)	0.018 (4)	0.017 (4)	0.004 (3)	−0.001 (3)	0.000 (3)
C43	0.017 (4)	0.016 (4)	0.019 (4)	0.006 (3)	0.004 (3)	−0.001 (3)
C44	0.020 (4)	0.019 (4)	0.030 (4)	0.007 (3)	0.002 (3)	0.002 (3)
C45	0.016 (4)	0.025 (4)	0.032 (4)	0.013 (3)	−0.005 (3)	0.001 (3)
C46	0.024 (4)	0.017 (4)	0.019 (4)	0.008 (4)	−0.007 (3)	−0.001 (3)
C47	0.012 (4)	0.017 (5)	0.013 (5)	0.005 (4)	0.002 (3)	−0.007 (4)
C48	0.026 (4)	0.017 (4)	0.021 (4)	0.005 (3)	0.006 (4)	0.002 (3)
C49	0.033 (4)	0.024 (4)	0.020 (4)	0.011 (4)	0.008 (3)	−0.001 (3)
C50	0.042 (5)	0.029 (5)	0.029 (4)	0.013 (4)	0.009 (4)	0.013 (3)
C51	0.052 (5)	0.044 (6)	0.022 (4)	0.021 (5)	0.013 (4)	0.011 (4)
C52	0.038 (5)	0.028 (6)	0.027 (5)	0.009 (4)	0.013 (4)	0.006 (4)
C53	0.026 (4)	0.023 (4)	0.024 (4)	0.006 (4)	0.005 (3)	0.003 (3)
C54	0.027 (4)	0.024 (5)	0.028 (4)	0.010 (4)	0.011 (3)	0.003 (3)
C78	0.037 (5)	0.033 (5)	0.032 (4)	0.017 (4)	0.004 (4)	0.003 (3)
C79	0.040 (5)	0.032 (5)	0.037 (5)	0.015 (4)	−0.003 (4)	0.012 (4)
C80	0.054 (6)	0.024 (5)	0.049 (6)	0.013 (4)	0.007 (4)	0.011 (4)
C81	0.029 (4)	0.041 (5)	0.028 (4)	0.023 (4)	−0.009 (4)	0.005 (4)
C82	0.026 (4)	0.024 (5)	0.031 (5)	0.011 (4)	0.011 (3)	0.005 (3)
C83	0.024 (4)	0.029 (5)	0.037 (5)	0.008 (4)	0.012 (4)	0.003 (4)
C84	0.029 (4)	0.027 (5)	0.042 (5)	0.014 (4)	0.007 (4)	0.004 (4)
C86	0.038 (4)	0.052 (5)	0.080 (5)	0.010 (4)	0.006 (3)	−0.021 (4)
C88	0.037 (4)	0.031 (4)	0.036 (4)	0.008 (4)	0.022 (4)	0.000 (3)
C89	0.045 (5)	0.047 (6)	0.055 (5)	−0.007 (5)	0.022 (4)	−0.018 (5)
C90	0.066 (7)	0.057 (7)	0.064 (6)	0.049 (6)	0.016 (5)	0.013 (5)
C92	0.072 (8)	0.070 (9)	0.053 (7)	0.008 (7)	0.011 (6)	0.006 (7)
C93	0.073 (7)	0.100 (10)	0.052 (7)	0.010 (7)	0.012 (5)	−0.024 (7)
C1S	0.091 (5)	0.133 (6)	0.151 (7)	0.078 (5)	0.064 (5)	0.041 (5)

### \ Tris[9,9-dihexyl-2-(5-methoxypyridin-2-yl-κN)-9H-fluoren-3-\ yl-κC3]iridium pentane monosolvate (I) Geometric parameters (Å, º)

**Table:**

Ir—N1	2.121 (3)	C70B—C71B	1.526 (13)
Ir—N2	2.135 (3)	C71B—H71C	0.9900
Ir—C19	2.020 (4)	C71B—H71D	0.9901
Ir—C1	2.017 (7)	C71B—C72B	1.505 (11)
Ir—C1B	2.013 (7)	C72B—H72C	0.9895
Ir—N3B	2.142 (7)	C72B—H72D	0.9902
Ir—C37B	2.045 (6)	C72B—C73B	1.516 (15)
Ir—N3	2.128 (7)	C73B—H73C	0.9908
Ir—C37	1.998 (6)	C73B—H73D	0.9899
O2—C28	1.362 (4)	C73B—C74B	1.525 (13)
O2—C68	1.434 (4)	C74B—H74D	0.9800
N1—C7	1.362 (4)	C74B—H74E	0.9800
N1—C11	1.343 (4)	C74B—H74F	0.9800
N2—C25	1.369 (4)	C77B—H77A	0.9888
N2—C29	1.327 (4)	C77B—H77B	0.9899
C7—C8	1.386 (5)	C77B—C78B	1.508 (8)
C7—C6	1.505 (7)	C78B—H78A	0.9898
C7—C6B	1.474 (7)	C78B—H78B	0.9901
C8—H8	0.9499	C78B—C79B	1.504 (10)
C8—C9	1.380 (5)	C79B—H79A	0.9898
C9—H9	0.9502	C79B—H79B	0.9899
C9—C10	1.379 (5)	C79B—C80B	1.511 (10)
C10—C11	1.386 (5)	C80B—H80A	0.9805
C10—O1	1.378 (11)	C80B—H80B	0.9802
C10—O1B	1.397 (11)	C80B—H80C	0.9802
C11—H11	0.9495	O3B—C46B	1.366 (9)
C19—C20	1.411 (5)	O3B—C81B	1.445 (9)
C19—C24	1.421 (5)	N3B—C43B	1.376 (9)
C20—H20	0.9496	N3B—C47B	1.330 (9)
C20—C21	1.389 (5)	C37B—C38B	1.420 (8)
C21—C22	1.394 (6)	C37B—C42B	1.434 (8)
C21—C30	1.470 (5)	C38B—H38B	0.9497
C22—C23	1.379 (5)	C38B—C39B	1.392 (9)
C22—C36	1.529 (5)	C39B—C40B	1.399 (10)
C23—H23	0.9499	C39B—C48B	1.462 (9)
C23—C24	1.398 (5)	C40B—C41B	1.371 (9)
C24—C25	1.460 (5)	C40B—C54B	1.544 (9)
C25—C26	1.392 (5)	C41B—H41B	0.9504
C26—H26	0.9502	C41B—C42B	1.399 (8)
C26—C27	1.367 (5)	C42B—C43B	1.463 (9)
C27—H27	0.9497	C43B—C44B	1.411 (8)
C27—C28	1.382 (5)	C44B—H44B	0.9499
C28—C29	1.385 (5)	C44B—C45B	1.368 (9)
C29—H29	0.9506	C45B—H45B	0.9499
C30—C31	1.395 (5)	C45B—C46B	1.387 (10)
C30—C35	1.391 (6)	C46B—C47B	1.390 (10)
C31—H31	0.9502	C47B—H47B	0.9503
C31—C32	1.388 (5)	C48B—C49B	1.380 (10)
C32—H32	0.9500	C48B—C53B	1.395 (10)
C32—C33	1.377 (6)	C49B—H49B	0.9497
C33—H33	0.9497	C49B—C50B	1.399 (10)
C33—C34	1.386 (6)	C50B—H50B	0.9499
C34—H34	0.9501	C50B—C51B	1.382 (11)
C34—C35	1.385 (5)	C51B—H51B	0.9497
C35—C36	1.524 (6)	C51B—C52B	1.383 (11)
C36—C75	1.534 (6)	C52B—H52B	0.9500
C36—C69	1.573 (10)	C52B—C53B	1.402 (9)
C36—C69B	1.603 (11)	C53B—C54B	1.524 (11)
C67—H67A	0.9805	C54B—C82B	1.542 (11)
C67—H67B	0.9804	C54B—C88B	1.534 (9)
C67—H67C	0.9797	C81B—H81A	0.9800
C67—H67D	0.9803	C81B—H81B	0.9800
C67—H67E	0.9801	C81B—H81C	0.9800
C67—H67F	0.9798	C82B—H82A	0.9899
C67—C66	1.639 (16)	C82B—H82B	0.9900
C67—C66B	1.425 (17)	C82B—C83B	1.531 (13)
C68—H68A	0.9800	C83B—H83A	0.9897
C68—H68B	0.9800	C83B—H83B	0.9898
C68—H68C	0.9800	C83B—C84B	1.522 (11)
C75—H75A	0.9900	C84B—H84A	0.9900
C75—H75B	0.9902	C84B—H84B	0.9900
C75—C76	1.519 (5)	C84B—C85B	1.357 (11)
C76—H76A	0.9899	C85B—H85A	0.9900
C76—H76B	0.9899	C85B—H85B	0.9900
C76—C77B	1.476 (7)	C85B—C86B	1.535 (12)
C76—C77	1.572 (7)	C86B—H86A	0.9900
O1—C55	1.426 (12)	C86B—H86B	0.9900
C1—C2	1.392 (9)	C86B—C87B	1.499 (13)
C1—C6	1.418 (9)	C87B—H87A	0.9800
C2—H2	0.9500	C87B—H87B	0.9800
C2—C3	1.385 (9)	C87B—H87C	0.9800
C3—C4	1.392 (8)	C88B—H88A	0.9900
C3—C12	1.474 (8)	C88B—H88B	0.9900
C4—C5	1.370 (8)	C88B—C89B	1.519 (8)
C4—C18	1.550 (8)	C89B—H89A	0.9900
C5—H5	0.9500	C89B—H89B	0.9900
C5—C6	1.418 (8)	C89B—C90B	1.515 (8)
C12—C13	1.386 (9)	C90B—H90A	0.9900
C12—C17	1.396 (8)	C90B—H90B	0.9900
C13—H13	0.9500	C90B—C91B	1.524 (9)
C13—C14	1.375 (9)	C91B—H91A	0.9900
C14—H14	0.9500	C91B—H91B	0.9900
C14—C15	1.395 (9)	C91B—C92B	1.544 (10)
C15—H15	0.9502	C92B—H92A	0.9900
C15—C16	1.372 (9)	C92B—H92B	0.9900
C16—H16	0.9498	C92B—C93B	1.654 (10)
C16—C17	1.387 (8)	C93B—H93A	0.9800
C17—C18	1.529 (9)	C93B—H93B	0.9800
C18—C56	1.539 (11)	C93B—H93C	0.9800
C18—C62	1.555 (10)	O3—C46	1.359 (9)
C55—H55A	0.9800	O3—C81	1.437 (8)
C55—H55B	0.9800	N3—C43	1.375 (9)
C55—H55C	0.9800	N3—C47	1.320 (9)
C56—H56A	0.9903	C37—C38	1.388 (8)
C56—H56B	0.9899	C37—C42	1.422 (9)
C56—C57	1.522 (10)	C38—H38	0.9503
C57—H57A	0.9898	C38—C39	1.383 (9)
C57—H57B	0.9907	C39—C40	1.394 (10)
C57—C58	1.531 (10)	C39—C48	1.463 (9)
C58—H58A	0.9898	C40—C41	1.383 (9)
C58—H58B	0.9896	C40—C54	1.550 (10)
C58—C59	1.486 (13)	C41—H41	0.9505
C59—H59A	0.9897	C41—C42	1.413 (8)
C59—H59B	0.9899	C42—C43	1.447 (9)
C59—C60	1.619 (12)	C43—C44	1.405 (8)
C60—H60A	0.9901	C44—H44	0.9502
C60—H60B	0.9903	C44—C45	1.369 (9)
C60—C61	1.505 (10)	C45—H45	0.9495
C61—H61A	0.9800	C45—C46	1.386 (10)
C61—H61B	0.9800	C46—C47	1.403 (10)
C61—H61C	0.9800	C47—H47	0.9500
C62—H62A	0.9898	C48—C49	1.393 (10)
C62—H62B	0.9906	C48—C53	1.386 (10)
C62—C63	1.522 (10)	C49—H49	0.9500
C63—H63A	0.9901	C49—C50	1.389 (9)
C63—H63B	0.9900	C50—H50	0.9497
C63—C64	1.517 (11)	C50—C51	1.378 (10)
C64—H64A	0.9905	C51—H51	0.9501
C64—H64B	0.9900	C51—C52	1.385 (12)
C64—C65	1.535 (11)	C52—H52	0.9501
C65—H65A	0.9900	C52—C53	1.410 (10)
C65—H65B	0.9899	C53—C54	1.514 (11)
C65—C66	1.516 (15)	C54—C82	1.525 (12)
C66—H66A	0.9895	C54—C88	1.547 (9)
C66—H66B	0.9901	C77—H77C	0.9897
C69—H69A	0.9895	C77—H77D	0.9900
C69—H69B	0.9900	C77—C78	1.554 (8)
C69—C70	1.516 (11)	C78—H78C	0.9899
C70—H70A	0.9898	C78—H78D	0.9904
C70—H70B	0.9912	C78—C79	1.504 (10)
C70—C71	1.522 (13)	C79—H79C	0.9899
C71—H71A	0.9904	C79—H79D	0.9901
C71—H71B	0.9895	C79—C80	1.522 (11)
C71—C72	1.504 (13)	C80—H80D	0.9799
C72—H72A	0.9903	C80—H80E	0.9802
C72—H72B	0.9897	C80—H80F	0.9802
C72—C73	1.511 (14)	C81—H81D	0.9800
C73—H73A	0.9898	C81—H81E	0.9800
C73—H73B	0.9907	C81—H81F	0.9800
C73—C74	1.503 (13)	C82—H82C	0.9902
C74—H74A	0.9800	C82—H82D	0.9899
C74—H74B	0.9800	C82—C83	1.538 (12)
C74—H74C	0.9800	C83—H83C	0.9905
O1B—C55B	1.422 (13)	C83—H83D	0.9900
C1B—C2B	1.405 (9)	C83—C84	1.494 (11)
C1B—C6B	1.431 (9)	C84—H84C	0.9905
C2B—H2B	0.9500	C84—H84D	0.9902
C2B—C3B	1.394 (9)	C84—C85	1.556 (10)
C3B—C4B	1.396 (9)	C85—H85C	0.9900
C3B—C12B	1.468 (9)	C85—H85D	0.9900
C4B—C5B	1.372 (8)	C85—C86	1.447 (11)
C4B—C18B	1.543 (9)	C86—H86C	0.9898
C5B—H5B	0.9497	C86—H86D	0.9897
C5B—C6B	1.405 (8)	C86—C87	1.524 (13)
C12B—C13B	1.385 (9)	C87—H87D	0.9796
C12B—C17B	1.399 (8)	C87—H87E	0.9798
C13B—H13B	0.9500	C87—H87F	0.9806
C13B—C14B	1.384 (9)	C88—H88C	0.9900
C14B—H14B	0.9500	C88—H88D	0.9900
C14B—C15B	1.387 (9)	C88—C89	1.556 (9)
C15B—H15B	0.9497	C89—H89C	0.9900
C15B—C16B	1.392 (9)	C89—H89D	0.9900
C16B—H16B	0.9508	C89—C90	1.673 (10)
C16B—C17B	1.382 (8)	C90—H90C	0.9900
C17B—C18B	1.529 (9)	C90—H90D	0.9900
C18B—C56B	1.541 (10)	C90—C91	1.384 (10)
C18B—C62B	1.526 (12)	C91—H91C	0.9900
C55B—H55D	0.9800	C91—H91D	0.9900
C55B—H55E	0.9800	C91—C92	1.482 (11)
C55B—H55F	0.9800	C92—H92C	0.9900
C56B—H56C	0.9904	C92—H92D	0.9900
C56B—H56D	0.9900	C92—C93	1.502 (11)
C56B—C57B	1.526 (11)	C93—H93D	0.9800
C57B—H57C	0.9904	C93—H93E	0.9800
C57B—H57D	0.9900	C93—H93F	0.9800
C57B—C58B	1.520 (11)	C1S—H1SA	0.9800
C58B—H58C	0.9901	C1S—H1SB	0.9800
C58B—H58D	0.9895	C1S—H1SC	0.9800
C58B—C59B	1.519 (11)	C1S—H1SD	0.9800
C59B—H59C	0.9897	C1S—H1SE	0.9800
C59B—H59D	0.9899	C1S—H1SF	0.9800
C59B—C60B	1.514 (13)	C1S—C2S	1.531 (6)
C60B—H60C	0.9902	C1S—C2S'	1.518 (8)
C60B—H60D	0.9898	C2S—H2SA	0.9900
C60B—C61B	1.430 (13)	C2S—H2SB	0.9900
C61B—H61D	0.9800	C2S—C3S	1.570 (9)
C61B—H61E	0.9800	C3S—H3SA	0.9900
C61B—H61F	0.9800	C3S—H3SB	0.9900
C62B—H62C	0.9892	C3S—C4S	1.543 (9)
C62B—H62D	0.9892	C4S—H4SA	0.9900
C62B—C63B	1.516 (11)	C4S—H4SB	0.9900
C63B—H63C	0.9901	C4S—C5S	1.561 (7)
C63B—H63D	0.9902	C5S—H5SA	0.9800
C63B—C64B	1.537 (11)	C5S—H5SB	0.9800
C64B—H64C	0.9908	C5S—H5SC	0.9800
C64B—H64D	0.9899	C2S'—H2SC	0.9900
C64B—C65B	1.528 (11)	C2S'—H2SD	0.9900
C65B—H65C	0.9893	C2S'—C3S'	1.470 (9)
C65B—H65D	0.9902	C3S'—H3SC	0.9900
C65B—C66B	1.520 (15)	C3S'—H3SD	0.9900
C66B—H66C	0.9900	C3S'—C4S'	1.530 (7)
C66B—H66D	0.9900	C4S'—H4SC	0.9900
C69B—H69C	0.9910	C4S'—H4SD	0.9900
C69B—H69D	0.9899	C4S'—C5S'	1.543 (7)
C69B—C70B	1.510 (11)	C5S'—H5SD	0.9800
C70B—H70C	0.9901	C5S'—H5SE	0.9800
C70B—H70D	0.9900	C5S'—H5SF	0.9800
			
N1—Ir—N2	96.55 (10)	C70B—C69B—H69D	105.4
N1—Ir—N3B	89.3 (3)	C69B—C70B—H70C	108.8
N1—Ir—N3	99.9 (3)	C69B—C70B—H70D	109.4
N2—Ir—N3B	93.2 (2)	C69B—C70B—C71B	112.2 (8)
C19—Ir—N1	173.31 (12)	H70C—C70B—H70D	107.9
C19—Ir—N2	79.44 (13)	C71B—C70B—H70C	108.9
C19—Ir—N3B	96.3 (3)	C71B—C70B—H70D	109.5
C19—Ir—C37B	98.0 (3)	C70B—C71B—H71C	108.8
C19—Ir—N3	86.1 (3)	C70B—C71B—H71D	108.2
C1—Ir—N1	81.7 (2)	H71C—C71B—H71D	107.5
C1—Ir—N2	86.6 (3)	C72B—C71B—C70B	114.9 (8)
C1—Ir—C19	92.6 (2)	C72B—C71B—H71C	108.8
C1—Ir—N3	174.5 (3)	C72B—C71B—H71D	108.4
C1B—Ir—N1	77.9 (2)	C71B—C72B—H72C	108.5
C1B—Ir—N2	95.1 (3)	C71B—C72B—H72D	108.9
C1B—Ir—C19	97.0 (2)	C71B—C72B—C73B	114.1 (9)
C1B—Ir—N3B	165.4 (4)	H72C—C72B—H72D	107.6
C1B—Ir—C37B	93.8 (3)	C73B—C72B—H72C	108.8
C37B—Ir—N1	86.7 (2)	C73B—C72B—H72D	108.7
C37B—Ir—N2	171.0 (2)	C72B—C73B—H73C	108.8
C37B—Ir—N3B	78.4 (3)	C72B—C73B—H73D	108.7
N3—Ir—N2	98.4 (2)	C72B—C73B—C74B	114.8 (10)
C37—Ir—N1	94.3 (2)	H73C—C73B—H73D	107.6
C37—Ir—N2	169.1 (2)	C74B—C73B—H73C	108.2
C37—Ir—C19	89.6 (3)	C74B—C73B—H73D	108.6
C37—Ir—C1	93.6 (3)	C73B—C74B—H74D	109.5
C37—Ir—N3	81.0 (3)	C73B—C74B—H74E	109.5
C28—O2—C68	116.5 (3)	C73B—C74B—H74F	109.5
C7—N1—Ir	115.1 (2)	H74D—C74B—H74E	109.5
C11—N1—Ir	125.8 (2)	H74D—C74B—H74F	109.5
C11—N1—C7	119.0 (3)	H74E—C74B—H74F	109.5
C25—N2—Ir	114.2 (2)	C76—C77B—H77A	110.6
C29—N2—Ir	126.0 (2)	C76—C77B—H77B	111.2
C29—N2—C25	119.8 (3)	C76—C77B—C78B	105.3 (6)
N1—C7—C8	119.5 (3)	H77A—C77B—H77B	108.9
N1—C7—C6	110.9 (4)	C78B—C77B—H77A	109.9
N1—C7—C6B	115.8 (4)	C78B—C77B—H77B	110.9
C8—C7—C6	128.6 (4)	C77B—C78B—H78A	112.0
C8—C7—C6B	124.1 (4)	C77B—C78B—H78B	108.2
C7—C8—H8	119.1	H78A—C78B—H78B	108.1
C9—C8—C7	121.7 (4)	C79B—C78B—C77B	109.4 (6)
C9—C8—H8	119.3	C79B—C78B—H78A	109.6
C8—C9—H9	121.2	C79B—C78B—H78B	109.4
C10—C9—C8	118.0 (4)	C78B—C79B—H79A	109.3
C10—C9—H9	120.9	C78B—C79B—H79B	109.0
C9—C10—C11	119.0 (3)	C78B—C79B—C80B	112.3 (6)
C9—C10—O1B	132.5 (6)	H79A—C79B—H79B	107.9
C11—C10—O1B	107.4 (5)	C80B—C79B—H79A	109.1
O1—C10—C9	117.7 (5)	C80B—C79B—H79B	109.1
O1—C10—C11	122.8 (6)	C79B—C80B—H80A	109.7
N1—C11—C10	122.8 (3)	C79B—C80B—H80B	109.4
N1—C11—H11	118.5	C79B—C80B—H80C	109.3
C10—C11—H11	118.7	H80A—C80B—H80B	109.5
C20—C19—Ir	128.2 (3)	H80A—C80B—H80C	109.5
C20—C19—C24	116.3 (3)	H80B—C80B—H80C	109.5
C24—C19—Ir	115.5 (2)	C46B—O3B—C81B	116.4 (6)
C19—C20—H20	119.5	C43B—N3B—Ir	114.8 (5)
C21—C20—C19	121.1 (4)	C47B—N3B—Ir	125.6 (7)
C21—C20—H20	119.4	C47B—N3B—C43B	119.5 (8)
C20—C21—C22	121.2 (3)	C38B—C37B—Ir	128.5 (5)
C20—C21—C30	130.7 (4)	C38B—C37B—C42B	114.6 (5)
C22—C21—C30	108.0 (4)	C42B—C37B—Ir	116.8 (4)
C21—C22—C36	111.4 (3)	C37B—C38B—H38B	118.5
C23—C22—C21	119.3 (4)	C39B—C38B—C37B	121.9 (6)
C23—C22—C36	129.2 (4)	C39B—C38B—H38B	119.6
C22—C23—H23	120.0	C38B—C39B—C40B	120.6 (6)
C22—C23—C24	119.9 (4)	C38B—C39B—C48B	130.6 (8)
C24—C23—H23	120.1	C40B—C39B—C48B	108.7 (6)
C19—C24—C25	115.9 (3)	C39B—C40B—C54B	110.9 (6)
C23—C24—C19	122.1 (3)	C41B—C40B—C39B	120.5 (6)
C23—C24—C25	122.0 (3)	C41B—C40B—C54B	128.6 (6)
N2—C25—C24	114.9 (3)	C40B—C41B—H41B	120.7
N2—C25—C26	118.5 (3)	C40B—C41B—C42B	118.8 (6)
C26—C25—C24	126.6 (3)	C42B—C41B—H41B	120.5
C25—C26—H26	118.7	C37B—C42B—C43B	114.0 (5)
C27—C26—C25	121.9 (3)	C41B—C42B—C37B	123.6 (6)
C27—C26—H26	119.4	C41B—C42B—C43B	122.1 (6)
C26—C27—H27	120.7	N3B—C43B—C42B	115.9 (6)
C26—C27—C28	118.4 (3)	N3B—C43B—C44B	118.3 (6)
C28—C27—H27	120.9	C44B—C43B—C42B	125.8 (6)
O2—C28—C27	125.8 (3)	C43B—C44B—H44B	118.9
O2—C28—C29	115.8 (3)	C45B—C44B—C43B	121.5 (6)
C27—C28—C29	118.4 (3)	C45B—C44B—H44B	119.6
N2—C29—C28	122.9 (3)	C44B—C45B—H45B	120.6
N2—C29—H29	118.7	C44B—C45B—C46B	118.9 (7)
C28—C29—H29	118.3	C46B—C45B—H45B	120.5
C31—C30—C21	130.6 (4)	O3B—C46B—C45B	126.7 (7)
C35—C30—C21	108.7 (4)	O3B—C46B—C47B	115.2 (7)
C35—C30—C31	120.7 (4)	C45B—C46B—C47B	118.1 (8)
C30—C31—H31	121.1	N3B—C47B—C46B	123.5 (10)
C32—C31—C30	117.7 (4)	N3B—C47B—H47B	118.3
C32—C31—H31	121.3	C46B—C47B—H47B	118.2
C31—C32—H32	119.0	C49B—C48B—C39B	131.2 (8)
C33—C32—C31	121.8 (4)	C49B—C48B—C53B	120.3 (7)
C33—C32—H32	119.2	C53B—C48B—C39B	108.5 (7)
C32—C33—H33	119.8	C48B—C49B—H49B	120.2
C32—C33—C34	120.3 (4)	C48B—C49B—C50B	119.1 (8)
C34—C33—H33	119.8	C50B—C49B—H49B	120.7
C33—C34—H34	120.3	C49B—C50B—H50B	119.5
C35—C34—C33	118.8 (4)	C51B—C50B—C49B	120.4 (8)
C35—C34—H34	120.9	C51B—C50B—H50B	120.0
C30—C35—C36	111.3 (3)	C50B—C51B—H51B	119.4
C34—C35—C30	120.7 (4)	C50B—C51B—C52B	121.1 (8)
C34—C35—C36	128.0 (4)	C52B—C51B—H51B	119.5
C22—C36—C75	113.3 (4)	C51B—C52B—H52B	120.5
C22—C36—C69	114.1 (5)	C51B—C52B—C53B	118.5 (7)
C22—C36—C69B	107.8 (5)	C53B—C52B—H52B	121.0
C35—C36—C22	100.5 (3)	C48B—C53B—C52B	120.5 (8)
C35—C36—C75	112.2 (3)	C48B—C53B—C54B	111.8 (6)
C35—C36—C69	121.0 (5)	C52B—C53B—C54B	127.8 (7)
C35—C36—C69B	102.1 (4)	C53B—C54B—C40B	100.1 (6)
C75—C36—C69	96.5 (4)	C53B—C54B—C82B	111.5 (7)
C75—C36—C69B	118.9 (4)	C53B—C54B—C88B	111.9 (6)
H67A—C67—H67B	109.4	C82B—C54B—C40B	112.0 (6)
H67A—C67—H67C	109.5	C88B—C54B—C40B	113.1 (6)
H67B—C67—H67C	109.5	C88B—C54B—C82B	108.2 (6)
H67D—C67—H67E	109.4	O3B—C81B—H81A	109.5
H67D—C67—H67F	109.5	O3B—C81B—H81B	109.5
H67E—C67—H67F	109.5	O3B—C81B—H81C	109.5
C66—C67—H67A	109.4	H81A—C81B—H81B	109.5
C66—C67—H67B	109.4	H81A—C81B—H81C	109.5
C66—C67—H67C	109.6	H81B—C81B—H81C	109.5
C66B—C67—H67D	109.3	C54B—C82B—H82A	108.0
C66B—C67—H67E	109.7	C54B—C82B—H82B	108.7
C66B—C67—H67F	109.4	H82A—C82B—H82B	107.3
O2—C68—H68A	109.5	C83B—C82B—C54B	116.7 (8)
O2—C68—H68B	109.5	C83B—C82B—H82A	107.4
O2—C68—H68C	109.5	C83B—C82B—H82B	108.5
H68A—C68—H68B	109.5	C82B—C83B—H83A	110.2
H68A—C68—H68C	109.5	C82B—C83B—H83B	109.1
H68B—C68—H68C	109.5	H83A—C83B—H83B	108.1
C36—C75—H75A	108.4	C84B—C83B—C82B	108.3 (9)
C36—C75—H75B	108.1	C84B—C83B—H83A	104.7
H75A—C75—H75B	107.4	C84B—C83B—H83B	116.3
C76—C75—C36	116.7 (3)	C83B—C84B—H84A	106.8
C76—C75—H75A	108.0	C83B—C84B—H84B	106.8
C76—C75—H75B	108.0	H84A—C84B—H84B	106.7
C75—C76—H76A	108.9	C85B—C84B—C83B	122.1 (10)
C75—C76—H76B	108.6	C85B—C84B—H84A	106.8
C75—C76—C77	119.1 (5)	C85B—C84B—H84B	106.8
H76A—C76—H76B	107.7	C84B—C85B—H85A	108.4
C77B—C76—C75	106.6 (5)	C84B—C85B—H85B	108.4
C77B—C76—H76A	114.4	C84B—C85B—C86B	115.7 (9)
C77B—C76—H76B	110.5	H85A—C85B—H85B	107.4
C77—C76—H76A	104.6	C86B—C85B—H85A	108.4
C77—C76—H76B	107.4	C86B—C85B—H85B	108.4
C10—O1—C55	124.1 (9)	C85B—C86B—H86A	107.8
C2—C1—Ir	131.1 (6)	C85B—C86B—H86B	107.8
C2—C1—C6	117.4 (7)	H86A—C86B—H86B	107.1
C6—C1—Ir	111.4 (5)	C87B—C86B—C85B	118.2 (8)
C1—C2—H2	119.8	C87B—C86B—H86A	107.8
C3—C2—C1	120.5 (8)	C87B—C86B—H86B	107.8
C3—C2—H2	119.8	C86B—C87B—H87A	109.5
C2—C3—C4	121.6 (7)	C86B—C87B—H87B	109.5
C2—C3—C12	130.0 (7)	C86B—C87B—H87C	109.5
C4—C3—C12	108.4 (6)	H87A—C87B—H87B	109.5
C3—C4—C18	111.3 (6)	H87A—C87B—H87C	109.5
C5—C4—C3	120.0 (6)	H87B—C87B—H87C	109.5
C5—C4—C18	128.7 (6)	C54B—C88B—H88A	107.9
C4—C5—H5	120.5	C54B—C88B—H88B	107.9
C4—C5—C6	118.9 (6)	H88A—C88B—H88B	107.2
C6—C5—H5	120.6	C89B—C88B—C54B	117.5 (6)
C1—C6—C7	120.0 (6)	C89B—C88B—H88A	107.9
C1—C6—C5	121.5 (6)	C89B—C88B—H88B	107.9
C5—C6—C7	117.7 (6)	C88B—C89B—H89A	109.0
C13—C12—C3	131.4 (7)	C88B—C89B—H89B	109.0
C13—C12—C17	119.9 (7)	H89A—C89B—H89B	107.8
C17—C12—C3	108.6 (6)	C90B—C89B—C88B	112.9 (5)
C12—C13—H13	120.3	C90B—C89B—H89A	109.0
C14—C13—C12	119.5 (8)	C90B—C89B—H89B	109.0
C14—C13—H13	120.3	C89B—C90B—H90A	108.7
C13—C14—H14	119.7	C89B—C90B—H90B	108.7
C13—C14—C15	120.5 (9)	C89B—C90B—C91B	114.2 (6)
C15—C14—H14	119.7	H90A—C90B—H90B	107.6
C14—C15—H15	119.1	C91B—C90B—H90A	108.7
C16—C15—C14	120.3 (8)	C91B—C90B—H90B	108.7
C16—C15—H15	120.0	C90B—C91B—H91A	108.2
C15—C16—H16	120.7	C90B—C91B—H91B	108.2
C15—C16—C17	119.5 (7)	C90B—C91B—C92B	116.5 (7)
C17—C16—H16	119.8	H91A—C91B—H91B	107.3
C12—C17—C18	111.6 (6)	C92B—C91B—H91A	108.2
C16—C17—C12	120.2 (6)	C92B—C91B—H91B	108.2
C16—C17—C18	128.2 (6)	C91B—C92B—H92A	112.6
C4—C18—C62	110.8 (6)	C91B—C92B—H92B	112.6
C17—C18—C4	100.1 (5)	C91B—C92B—C93B	95.7 (7)
C17—C18—C56	112.8 (6)	H92A—C92B—H92B	110.1
C17—C18—C62	112.1 (7)	C93B—C92B—H92A	112.6
C56—C18—C4	111.0 (6)	C93B—C92B—H92B	112.6
C56—C18—C62	109.8 (6)	C92B—C93B—H93A	109.5
O1—C55—H55A	109.5	C92B—C93B—H93B	109.5
O1—C55—H55B	109.5	C92B—C93B—H93C	109.5
O1—C55—H55C	109.5	H93A—C93B—H93B	109.5
H55A—C55—H55B	109.5	H93A—C93B—H93C	109.5
H55A—C55—H55C	109.5	H93B—C93B—H93C	109.5
H55B—C55—H55C	109.5	C46—O3—C81	116.2 (6)
C18—C56—H56A	108.8	C43—N3—Ir	112.4 (5)
C18—C56—H56B	108.3	C47—N3—Ir	125.9 (6)
H56A—C56—H56B	107.6	C47—N3—C43	121.7 (7)
C57—C56—C18	114.7 (7)	C38—C37—Ir	127.1 (5)
C57—C56—H56A	108.9	C38—C37—C42	119.1 (6)
C57—C56—H56B	108.3	C42—C37—Ir	113.9 (5)
C56—C57—H57A	108.9	C37—C38—H38	120.3
C56—C57—H57B	109.4	C39—C38—C37	119.8 (6)
C56—C57—C58	112.0 (6)	C39—C38—H38	119.9
H57A—C57—H57B	107.9	C38—C39—C40	120.9 (7)
C58—C57—H57A	109.1	C38—C39—C48	130.2 (8)
C58—C57—H57B	109.4	C40—C39—C48	108.9 (6)
C57—C58—H58A	107.7	C39—C40—C54	110.1 (6)
C57—C58—H58B	108.0	C41—C40—C39	121.4 (6)
H58A—C58—H58B	107.2	C41—C40—C54	128.4 (7)
C59—C58—C57	118.0 (7)	C40—C41—H41	121.2
C59—C58—H58A	107.4	C40—C41—C42	117.6 (6)
C59—C58—H58B	108.1	C42—C41—H41	121.2
C58—C59—H59A	108.9	C37—C42—C43	117.0 (5)
C58—C59—H59B	109.2	C41—C42—C37	121.1 (6)
C58—C59—C60	113.1 (7)	C41—C42—C43	121.9 (6)
H59A—C59—H59B	107.6	N3—C43—C42	115.4 (6)
C60—C59—H59A	108.5	N3—C43—C44	116.7 (6)
C60—C59—H59B	109.3	C44—C43—C42	127.8 (6)
C59—C60—H60A	108.3	C43—C44—H44	118.5
C59—C60—H60B	108.0	C45—C44—C43	122.4 (6)
H60A—C60—H60B	107.4	C45—C44—H44	119.1
C61—C60—C59	115.7 (7)	C44—C45—H45	120.8
C61—C60—H60A	108.7	C44—C45—C46	119.0 (6)
C61—C60—H60B	108.4	C46—C45—H45	120.3
C60—C61—H61A	109.5	O3—C46—C45	127.5 (7)
C60—C61—H61B	109.5	O3—C46—C47	114.7 (8)
C60—C61—H61C	109.5	C45—C46—C47	117.8 (7)
H61A—C61—H61B	109.5	N3—C47—C46	122.3 (8)
H61A—C61—H61C	109.5	N3—C47—H47	118.8
H61B—C61—H61C	109.5	C46—C47—H47	118.8
C18—C62—H62A	108.3	C49—C48—C39	130.7 (7)
C18—C62—H62B	108.7	C53—C48—C39	108.5 (7)
H62A—C62—H62B	107.5	C53—C48—C49	120.8 (7)
C63—C62—C18	115.3 (7)	C48—C49—H49	120.6
C63—C62—H62A	108.0	C50—C49—C48	118.8 (7)
C63—C62—H62B	108.7	C50—C49—H49	120.6
C62—C63—H63A	108.9	C49—C50—H50	119.6
C62—C63—H63B	108.3	C51—C50—C49	120.7 (7)
H63A—C63—H63B	107.6	C51—C50—H50	119.7
C64—C63—C62	114.3 (8)	C50—C51—H51	119.2
C64—C63—H63A	109.2	C50—C51—C52	121.2 (8)
C64—C63—H63B	108.3	C52—C51—H51	119.6
C63—C64—H64A	107.8	C51—C52—H52	120.7
C63—C64—H64B	108.0	C51—C52—C53	118.4 (8)
C63—C64—C65	116.8 (7)	C53—C52—H52	120.8
H64A—C64—H64B	107.4	C48—C53—C52	120.1 (8)
C65—C64—H64A	107.9	C48—C53—C54	111.9 (7)
C65—C64—H64B	108.6	C52—C53—C54	128.0 (8)
C64—C65—H65A	108.6	C53—C54—C40	100.5 (6)
C64—C65—H65B	108.9	C53—C54—C82	112.8 (7)
H65A—C65—H65B	107.6	C53—C54—C88	109.7 (7)
C66—C65—C64	113.9 (9)	C82—C54—C40	111.3 (7)
C66—C65—H65A	108.2	C82—C54—C88	110.8 (6)
C66—C65—H65B	109.4	C88—C54—C40	111.4 (6)
C67—C66—H66A	108.7	C76—C77—H77C	107.3
C67—C66—H66B	108.6	C76—C77—H77D	106.7
C65—C66—C67	114.3 (12)	H77C—C77—H77D	106.8
C65—C66—H66A	109.4	C78—C77—C76	120.8 (7)
C65—C66—H66B	108.1	C78—C77—H77C	107.6
H66A—C66—H66B	107.5	C78—C77—H77D	106.9
C36—C69—H69A	110.7	C77—C78—H78C	106.9
C36—C69—H69B	110.7	C77—C78—H78D	110.4
H69A—C69—H69B	108.8	H78C—C78—H78D	106.1
C70—C69—C36	105.3 (7)	C79—C78—C77	121.9 (7)
C70—C69—H69A	110.8	C79—C78—H78C	104.9
C70—C69—H69B	110.5	C79—C78—H78D	105.5
C69—C70—H70A	108.7	C78—C79—H79C	108.6
C69—C70—H70B	108.9	C78—C79—H79D	109.0
C69—C70—C71	113.7 (8)	C78—C79—C80	114.2 (7)
H70A—C70—H70B	107.6	H79C—C79—H79D	107.6
C71—C70—H70A	109.1	C80—C79—H79C	108.8
C71—C70—H70B	108.7	C80—C79—H79D	108.4
C70—C71—H71A	109.0	C79—C80—H80D	109.6
C70—C71—H71B	108.6	C79—C80—H80E	109.2
H71A—C71—H71B	107.7	C79—C80—H80F	109.7
C72—C71—C70	114.1 (8)	H80D—C80—H80E	109.5
C72—C71—H71A	108.6	H80D—C80—H80F	109.5
C72—C71—H71B	108.6	H80E—C80—H80F	109.4
C71—C72—H72A	108.1	O3—C81—H81D	109.5
C71—C72—H72B	108.1	O3—C81—H81E	109.5
C71—C72—C73	116.5 (9)	O3—C81—H81F	109.5
H72A—C72—H72B	107.4	H81D—C81—H81E	109.5
C73—C72—H72A	107.9	H81D—C81—H81F	109.5
C73—C72—H72B	108.6	H81E—C81—H81F	109.5
C72—C73—H73A	108.4	C54—C82—H82C	108.7
C72—C73—H73B	109.2	C54—C82—H82D	108.6
H73A—C73—H73B	107.7	C54—C82—C83	114.8 (7)
C74—C73—C72	113.7 (10)	H82C—C82—H82D	107.6
C74—C73—H73A	109.1	C83—C82—H82C	108.4
C74—C73—H73B	108.6	C83—C82—H82D	108.5
C73—C74—H74A	109.5	C82—C83—H83C	108.1
C73—C74—H74B	109.5	C82—C83—H83D	108.4
C73—C74—H74C	109.5	H83C—C83—H83D	107.4
H74A—C74—H74B	109.5	C84—C83—C82	116.0 (8)
H74A—C74—H74C	109.5	C84—C83—H83C	108.3
H74B—C74—H74C	109.5	C84—C83—H83D	108.4
C10—O1B—C55B	109.7 (8)	C83—C84—H84C	109.0
C2B—C1B—Ir	125.6 (6)	C83—C84—H84D	109.1
C2B—C1B—C6B	115.7 (7)	C83—C84—C85	114.1 (8)
C6B—C1B—Ir	118.6 (5)	H84C—C84—H84D	107.8
C1B—C2B—H2B	119.2	C85—C84—H84C	112.9
C3B—C2B—C1B	121.6 (8)	C85—C84—H84D	103.7
C3B—C2B—H2B	119.2	C84—C85—H85C	107.8
C2B—C3B—C4B	120.7 (7)	C84—C85—H85D	107.8
C2B—C3B—C12B	131.1 (7)	H85C—C85—H85D	107.1
C4B—C3B—C12B	108.1 (6)	C86—C85—C84	118.0 (9)
C3B—C4B—C18B	111.2 (6)	C86—C85—H85C	107.8
C5B—C4B—C3B	120.2 (6)	C86—C85—H85D	107.8
C5B—C4B—C18B	128.5 (6)	C85—C86—H86C	113.4
C4B—C5B—H5B	120.7	C85—C86—H86D	105.8
C4B—C5B—C6B	119.0 (6)	C85—C86—C87	112.4 (9)
C6B—C5B—H5B	120.3	H86C—C86—H86D	107.4
C1B—C6B—C7	111.1 (6)	C87—C86—H86C	109.3
C5B—C6B—C7	125.5 (6)	C87—C86—H86D	108.3
C5B—C6B—C1B	122.7 (6)	C86—C87—H87D	108.6
C13B—C12B—C3B	130.6 (7)	C86—C87—H87E	110.5
C13B—C12B—C17B	120.2 (7)	C86—C87—H87F	109.4
C17B—C12B—C3B	109.2 (6)	H87D—C87—H87E	109.5
C12B—C13B—H13B	120.6	H87D—C87—H87F	109.5
C14B—C13B—C12B	118.7 (8)	H87E—C87—H87F	109.4
C14B—C13B—H13B	120.6	C54—C88—H88C	108.5
C13B—C14B—H14B	119.5	C54—C88—H88D	108.5
C13B—C14B—C15B	121.0 (9)	C54—C88—C89	115.0 (7)
C15B—C14B—H14B	119.5	H88C—C88—H88D	107.5
C14B—C15B—H15B	119.2	C89—C88—H88C	108.5
C14B—C15B—C16B	120.7 (8)	C89—C88—H88D	108.5
C16B—C15B—H15B	119.4	C88—C89—H89C	105.4
C15B—C16B—H16B	120.9	C88—C89—H89D	105.4
C17B—C16B—C15B	118.3 (7)	C88—C89—C90	127.4 (7)
C17B—C16B—H16B	120.8	H89C—C89—H89D	106.0
C12B—C17B—C18B	110.9 (6)	C90—C89—H89C	105.4
C16B—C17B—C12B	121.0 (6)	C90—C89—H89D	105.4
C16B—C17B—C18B	128.0 (6)	C89—C90—H90C	108.8
C17B—C18B—C4B	100.6 (6)	C89—C90—H90D	108.8
C17B—C18B—C56B	110.6 (7)	H90C—C90—H90D	107.7
C56B—C18B—C4B	112.5 (7)	C91—C90—C89	113.7 (9)
C62B—C18B—C4B	112.0 (6)	C91—C90—H90C	108.8
C62B—C18B—C17B	112.0 (7)	C91—C90—H90D	108.8
C62B—C18B—C56B	109.0 (7)	C90—C91—H91C	108.9
O1B—C55B—H55D	109.5	C90—C91—H91D	108.9
O1B—C55B—H55E	109.5	C90—C91—C92	113.2 (10)
O1B—C55B—H55F	109.5	H91C—C91—H91D	107.7
H55D—C55B—H55E	109.5	C92—C91—H91C	108.9
H55D—C55B—H55F	109.5	C92—C91—H91D	108.9
H55E—C55B—H55F	109.5	C91—C92—H92C	109.2
C18B—C56B—H56C	108.2	C91—C92—H92D	109.2
C18B—C56B—H56D	108.2	C91—C92—C93	111.9 (10)
H56C—C56B—H56D	107.4	H92C—C92—H92D	107.9
C57B—C56B—C18B	116.6 (7)	C93—C92—H92C	109.2
C57B—C56B—H56C	108.3	C93—C92—H92D	109.2
C57B—C56B—H56D	107.9	C92—C93—H93D	109.5
C56B—C57B—H57C	109.0	C92—C93—H93E	109.5
C56B—C57B—H57D	108.7	C92—C93—H93F	109.5
H57C—C57B—H57D	107.7	H93D—C93—H93E	109.5
C58B—C57B—C56B	112.7 (8)	H93D—C93—H93F	109.5
C58B—C57B—H57C	109.6	H93E—C93—H93F	109.5
C58B—C57B—H57D	109.1	H1SA—C1S—H1SB	109.5
C57B—C58B—H58C	108.4	H1SA—C1S—H1SC	109.5
C57B—C58B—H58D	109.3	H1SB—C1S—H1SC	109.5
H58C—C58B—H58D	107.5	H1SD—C1S—H1SE	109.5
C59B—C58B—C57B	114.5 (7)	H1SD—C1S—H1SF	109.5
C59B—C58B—H58C	107.8	H1SE—C1S—H1SF	109.5
C59B—C58B—H58D	109.1	C2S—C1S—H1SA	109.5
C58B—C59B—H59C	107.1	C2S—C1S—H1SB	109.5
C58B—C59B—H59D	107.8	C2S—C1S—H1SC	109.5
H59C—C59B—H59D	107.1	C2S'—C1S—H1SD	109.5
C60B—C59B—C58B	119.4 (8)	C2S'—C1S—H1SE	109.5
C60B—C59B—H59C	107.1	C2S'—C1S—H1SF	109.5
C60B—C59B—H59D	107.7	C1S—C2S—H2SA	110.0
C59B—C60B—H60C	108.0	C1S—C2S—H2SB	110.0
C59B—C60B—H60D	109.1	C1S—C2S—C3S	108.7 (8)
H60C—C60B—H60D	107.4	H2SA—C2S—H2SB	108.3
C61B—C60B—C59B	116.3 (11)	C3S—C2S—H2SA	110.0
C61B—C60B—H60C	108.4	C3S—C2S—H2SB	110.0
C61B—C60B—H60D	107.2	C2S—C3S—H3SA	109.3
C60B—C61B—H61D	109.5	C2S—C3S—H3SB	109.3
C60B—C61B—H61E	109.5	H3SA—C3S—H3SB	108.0
C60B—C61B—H61F	109.5	C4S—C3S—C2S	111.5 (9)
H61D—C61B—H61E	109.5	C4S—C3S—H3SA	109.3
H61D—C61B—H61F	109.5	C4S—C3S—H3SB	109.3
H61E—C61B—H61F	109.5	C3S—C4S—H4SA	112.8
C18B—C62B—H62C	108.3	C3S—C4S—H4SB	112.8
C18B—C62B—H62D	108.3	C3S—C4S—C5S	94.5 (9)
H62C—C62B—H62D	107.4	H4SA—C4S—H4SB	110.3
C63B—C62B—C18B	116.1 (7)	C5S—C4S—H4SA	112.8
C63B—C62B—H62C	108.4	C5S—C4S—H4SB	112.8
C63B—C62B—H62D	108.0	C4S—C5S—H5SA	109.5
C62B—C63B—H63C	109.0	C4S—C5S—H5SB	109.5
C62B—C63B—H63D	108.5	C4S—C5S—H5SC	109.5
C62B—C63B—C64B	114.3 (7)	H5SA—C5S—H5SB	109.5
H63C—C63B—H63D	107.7	H5SA—C5S—H5SC	109.5
C64B—C63B—H63C	109.2	H5SB—C5S—H5SC	109.5
C64B—C63B—H63D	108.0	C1S—C2S'—H2SC	103.4
C63B—C64B—H64C	109.0	C1S—C2S'—H2SD	103.4
C63B—C64B—H64D	108.0	H2SC—C2S'—H2SD	105.2
H64C—C64B—H64D	107.6	C3S'—C2S'—C1S	135.1 (10)
C65B—C64B—C63B	114.8 (7)	C3S'—C2S'—H2SC	103.4
C65B—C64B—H64C	108.7	C3S'—C2S'—H2SD	103.4
C65B—C64B—H64D	108.6	C2S'—C3S'—H3SC	108.1
C64B—C65B—H65C	108.7	C2S'—C3S'—H3SD	108.1
C64B—C65B—H65D	108.6	C2S'—C3S'—C4S'	116.7 (10)
H65C—C65B—H65D	107.5	H3SC—C3S'—H3SD	107.3
C66B—C65B—C64B	114.8 (9)	C4S'—C3S'—H3SC	108.1
C66B—C65B—H65C	108.2	C4S'—C3S'—H3SD	108.1
C66B—C65B—H65D	108.8	C3S'—C4S'—H4SC	106.3
C67—C66B—C65B	111.3 (12)	C3S'—C4S'—H4SD	106.3
C67—C66B—H66C	109.4	C3S'—C4S'—C5S'	123.9 (12)
C67—C66B—H66D	109.4	H4SC—C4S'—H4SD	106.4
C65B—C66B—H66C	109.4	C5S'—C4S'—H4SC	106.3
C65B—C66B—H66D	109.4	C5S'—C4S'—H4SD	106.3
H66C—C66B—H66D	108.0	C4S'—C5S'—H5SD	109.5
C36—C69B—H69C	105.9	C4S'—C5S'—H5SE	109.5
C36—C69B—H69D	105.5	C4S'—C5S'—H5SF	109.5
H69C—C69B—H69D	106.1	H5SD—C5S'—H5SE	109.5
C70B—C69B—C36	126.6 (7)	H5SD—C5S'—H5SF	109.5
C70B—C69B—H69C	105.9	H5SE—C5S'—H5SF	109.5
			
Ir—N1—C7—C8	178.8 (3)	C3B—C4B—C18B—C17B	0.6 (9)
Ir—N1—C7—C6	8.9 (5)	C3B—C4B—C18B—C56B	118.3 (8)
Ir—N1—C7—C6B	−10.1 (5)	C3B—C4B—C18B—C62B	−118.5 (8)
Ir—N1—C11—C10	−179.0 (3)	C3B—C12B—C13B—C14B	−179.9 (8)
Ir—N2—C25—C24	1.9 (3)	C3B—C12B—C17B—C16B	−179.8 (7)
Ir—N2—C25—C26	179.9 (2)	C3B—C12B—C17B—C18B	−2.3 (10)
Ir—N2—C29—C28	−178.1 (2)	C4B—C3B—C12B—C13B	−178.4 (9)
Ir—C19—C20—C21	178.9 (3)	C4B—C3B—C12B—C17B	2.6 (10)
Ir—C19—C24—C23	178.6 (3)	C4B—C5B—C6B—C7	−169.5 (7)
Ir—C19—C24—C25	−2.0 (4)	C4B—C5B—C6B—C1B	0.2 (12)
Ir—C1—C2—C3	−174.8 (7)	C4B—C18B—C56B—C57B	−63.7 (9)
Ir—C1—C6—C7	5.5 (9)	C4B—C18B—C62B—C63B	63.4 (9)
Ir—C1—C6—C5	174.9 (6)	C5B—C4B—C18B—C17B	−179.2 (8)
Ir—C1B—C2B—C3B	−178.6 (6)	C5B—C4B—C18B—C56B	−61.5 (11)
Ir—C1B—C6B—C7	−11.4 (9)	C5B—C4B—C18B—C62B	61.7 (11)
Ir—C1B—C6B—C5B	177.6 (6)	C6B—C7—C8—C9	−169.2 (5)
Ir—N3B—C43B—C42B	−2.4 (10)	C6B—C1B—C2B—C3B	−0.6 (12)
Ir—N3B—C43B—C44B	177.0 (6)	C12B—C3B—C4B—C5B	177.8 (7)
Ir—N3B—C47B—C46B	−179.2 (9)	C12B—C3B—C4B—C18B	−2.0 (10)
Ir—C37B—C38B—C39B	−174.0 (6)	C12B—C13B—C14B—C15B	0.3 (13)
Ir—C37B—C42B—C41B	176.4 (6)	C12B—C17B—C18B—C4B	1.0 (9)
Ir—C37B—C42B—C43B	2.3 (9)	C12B—C17B—C18B—C56B	−118.0 (7)
Ir—N3—C43—C42	1.6 (10)	C12B—C17B—C18B—C62B	120.2 (8)
Ir—N3—C43—C44	178.8 (5)	C13B—C12B—C17B—C16B	1.1 (13)
Ir—N3—C47—C46	−177.1 (9)	C13B—C12B—C17B—C18B	178.7 (8)
Ir—C37—C38—C39	−178.1 (6)	C13B—C14B—C15B—C16B	0.4 (13)
Ir—C37—C42—C41	176.3 (6)	C14B—C15B—C16B—C17B	−0.4 (13)
Ir—C37—C42—C43	−6.0 (9)	C15B—C16B—C17B—C12B	−0.4 (12)
O2—C28—C29—N2	179.1 (3)	C15B—C16B—C17B—C18B	−177.4 (8)
N1—C7—C8—C9	1.1 (6)	C16B—C17B—C18B—C4B	178.4 (8)
N1—C7—C6—C1	−9.8 (9)	C16B—C17B—C18B—C56B	59.3 (11)
N1—C7—C6—C5	−179.6 (6)	C16B—C17B—C18B—C62B	−62.5 (11)
N1—C7—C6B—C1B	13.7 (8)	C17B—C12B—C13B—C14B	−1.1 (13)
N1—C7—C6B—C5B	−175.6 (6)	C17B—C18B—C56B—C57B	47.8 (9)
N2—C25—C26—C27	−1.7 (5)	C17B—C18B—C62B—C63B	−48.7 (9)
C7—N1—C11—C10	1.1 (5)	C18B—C4B—C5B—C6B	−178.9 (8)
C7—C8—C9—C10	−0.7 (6)	C18B—C56B—C57B—C58B	−175.7 (7)
C8—C7—C6—C1	−178.5 (6)	C18B—C62B—C63B—C64B	−176.5 (6)
C8—C7—C6—C5	11.7 (10)	C56B—C18B—C62B—C63B	−171.4 (7)
C8—C7—C6B—C1B	−175.7 (6)	C56B—C57B—C58B—C59B	160.5 (8)
C8—C7—C6B—C5B	−5.0 (10)	C57B—C58B—C59B—C60B	60.9 (12)
C8—C9—C10—C11	0.5 (6)	C58B—C59B—C60B—C61B	74.1 (13)
C8—C9—C10—O1	−171.1 (6)	C62B—C18B—C56B—C57B	171.4 (7)
C8—C9—C10—O1B	166.1 (6)	C62B—C63B—C64B—C65B	−55.0 (10)
C9—C10—C11—N1	−0.7 (6)	C63B—C64B—C65B—C66B	−63.7 (11)
C9—C10—O1—C55	−0.5 (12)	C64B—C65B—C66B—C67	178.1 (8)
C9—C10—O1B—C55B	19.3 (12)	C69B—C36—C75—C76	176.9 (5)
C11—N1—C7—C8	−1.2 (5)	C69B—C70B—C71B—C72B	165.1 (10)
C11—N1—C7—C6	−171.1 (4)	C70B—C71B—C72B—C73B	171.6 (10)
C11—N1—C7—C6B	169.9 (4)	C71B—C72B—C73B—C74B	−178.2 (11)
C11—C10—O1—C55	−171.7 (8)	C77B—C78B—C79B—C80B	−173.0 (7)
C11—C10—O1B—C55B	−173.8 (8)	O3B—C46B—C47B—N3B	−178.9 (11)
C19—C20—C21—C22	2.2 (5)	N3B—C43B—C44B—C45B	3.5 (12)
C19—C20—C21—C30	−174.2 (3)	C37B—C38B—C39B—C40B	−2.5 (12)
C19—C24—C25—N2	−0.1 (4)	C37B—C38B—C39B—C48B	172.1 (8)
C19—C24—C25—C26	−177.8 (3)	C37B—C42B—C43B—N3B	0.2 (11)
C20—C19—C24—C23	−2.1 (5)	C37B—C42B—C43B—C44B	−179.1 (7)
C20—C19—C24—C25	177.3 (3)	C38B—C37B—C42B—C41B	−0.7 (12)
C20—C21—C22—C23	−1.8 (6)	C38B—C37B—C42B—C43B	−174.8 (7)
C20—C21—C22—C36	179.8 (3)	C38B—C39B—C40B—C41B	0.0 (12)
C20—C21—C30—C31	1.1 (7)	C38B—C39B—C40B—C54B	177.1 (7)
C20—C21—C30—C35	177.9 (4)	C38B—C39B—C48B—C49B	5.0 (15)
C21—C22—C23—C24	−0.6 (6)	C38B—C39B—C48B—C53B	−173.9 (8)
C21—C22—C36—C35	3.6 (4)	C39B—C40B—C41B—C42B	1.9 (11)
C21—C22—C36—C75	123.4 (4)	C39B—C40B—C54B—C53B	−3.2 (8)
C21—C22—C36—C69	−127.5 (5)	C39B—C40B—C54B—C82B	115.0 (8)
C21—C22—C36—C69B	−102.8 (5)	C39B—C40B—C54B—C88B	−122.4 (7)
C21—C30—C31—C32	175.4 (4)	C39B—C48B—C49B—C50B	−175.3 (8)
C21—C30—C35—C34	−176.4 (3)	C39B—C48B—C53B—C52B	176.6 (7)
C21—C30—C35—C36	1.3 (4)	C39B—C48B—C53B—C54B	−3.5 (9)
C22—C21—C30—C31	−175.7 (4)	C40B—C39B—C48B—C49B	−179.9 (8)
C22—C21—C30—C35	1.1 (4)	C40B—C39B—C48B—C53B	1.2 (9)
C22—C23—C24—C19	2.6 (6)	C40B—C41B—C42B—C37B	−1.6 (12)
C22—C23—C24—C25	−176.8 (3)	C40B—C41B—C42B—C43B	172.1 (7)
C22—C36—C75—C76	−54.9 (5)	C40B—C54B—C82B—C83B	−64.5 (10)
C22—C36—C69—C70	66.2 (9)	C40B—C54B—C88B—C89B	50.1 (9)
C22—C36—C69B—C70B	44.6 (11)	C41B—C40B—C54B—C53B	173.6 (8)
C23—C22—C36—C35	−174.6 (4)	C41B—C40B—C54B—C82B	−68.2 (10)
C23—C22—C36—C75	−54.8 (5)	C41B—C40B—C54B—C88B	54.4 (10)
C23—C22—C36—C69	54.3 (7)	C41B—C42B—C43B—N3B	−174.1 (8)
C23—C22—C36—C69B	79.0 (6)	C41B—C42B—C43B—C44B	6.6 (12)
C23—C24—C25—N2	179.3 (3)	C42B—C37B—C38B—C39B	2.7 (11)
C23—C24—C25—C26	1.6 (5)	C42B—C43B—C44B—C45B	−177.2 (7)
C24—C19—C20—C21	−0.3 (5)	C43B—N3B—C47B—C46B	0.4 (19)
C24—C25—C26—C27	175.9 (3)	C43B—C44B—C45B—C46B	−2.0 (12)
C25—N2—C29—C28	1.0 (5)	C44B—C45B—C46B—O3B	179.8 (7)
C25—C26—C27—C28	1.1 (5)	C44B—C45B—C46B—C47B	−0.3 (13)
C26—C27—C28—O2	179.8 (3)	C45B—C46B—C47B—N3B	1.2 (18)
C26—C27—C28—C29	0.5 (5)	C47B—N3B—C43B—C42B	178.0 (10)
C27—C28—C29—N2	−1.6 (5)	C47B—N3B—C43B—C44B	−2.7 (14)
C29—N2—C25—C24	−177.3 (3)	C48B—C39B—C40B—C41B	−175.6 (7)
C29—N2—C25—C26	0.7 (5)	C48B—C39B—C40B—C54B	1.4 (9)
C30—C21—C22—C23	175.4 (3)	C48B—C49B—C50B—C51B	−2.0 (12)
C30—C21—C22—C36	−3.0 (4)	C48B—C53B—C54B—C40B	4.0 (8)
C30—C31—C32—C33	0.1 (6)	C48B—C53B—C54B—C82B	−114.6 (7)
C30—C35—C36—C22	−2.9 (4)	C48B—C53B—C54B—C88B	124.1 (7)
C30—C35—C36—C75	−123.5 (4)	C49B—C48B—C53B—C52B	−2.4 (12)
C30—C35—C36—C69	123.7 (5)	C49B—C48B—C53B—C54B	177.5 (7)
C30—C35—C36—C69B	108.0 (5)	C49B—C50B—C51B—C52B	−0.7 (13)
C31—C30—C35—C34	0.8 (6)	C50B—C51B—C52B—C53B	1.8 (12)
C31—C30—C35—C36	178.5 (3)	C51B—C52B—C53B—C48B	−0.2 (11)
C31—C32—C33—C34	1.1 (6)	C51B—C52B—C53B—C54B	179.9 (8)
C32—C33—C34—C35	−1.3 (6)	C52B—C53B—C54B—C40B	−176.0 (8)
C33—C34—C35—C30	0.4 (6)	C52B—C53B—C54B—C82B	65.3 (10)
C33—C34—C35—C36	−176.9 (4)	C52B—C53B—C54B—C88B	−55.9 (11)
C34—C35—C36—C22	174.6 (4)	C53B—C48B—C49B—C50B	3.5 (12)
C34—C35—C36—C75	53.9 (5)	C53B—C54B—C82B—C83B	46.7 (10)
C34—C35—C36—C69	−58.8 (7)	C53B—C54B—C88B—C89B	−62.1 (9)
C34—C35—C36—C69B	−74.5 (6)	C54B—C40B—C41B—C42B	−174.6 (7)
C35—C30—C31—C32	−1.0 (5)	C54B—C82B—C83B—C84B	169.2 (8)
C35—C36—C75—C76	58.0 (5)	C54B—C88B—C89B—C90B	173.3 (6)
C35—C36—C69—C70	−53.9 (9)	C81B—O3B—C46B—C45B	5.1 (12)
C35—C36—C69B—C70B	−60.7 (10)	C81B—O3B—C46B—C47B	−174.8 (9)
C36—C22—C23—C24	177.5 (4)	C82B—C54B—C88B—C89B	174.8 (6)
C36—C75—C76—C77B	178.7 (6)	C82B—C83B—C84B—C85B	−172.4 (10)
C36—C75—C76—C77	−177.1 (5)	C83B—C84B—C85B—C86B	175.2 (10)
C36—C69—C70—C71	−176.9 (8)	C84B—C85B—C86B—C87B	−98.5 (12)
C36—C69B—C70B—C71B	165.7 (8)	C88B—C54B—C82B—C83B	170.1 (8)
C68—O2—C28—C27	10.9 (6)	C88B—C89B—C90B—C91B	−178.8 (6)
C68—O2—C28—C29	−169.8 (3)	C89B—C90B—C91B—C92B	177.5 (8)
C75—C36—C69—C70	−174.6 (7)	C90B—C91B—C92B—C93B	−148.6 (7)
C75—C36—C69B—C70B	175.3 (8)	O3—C46—C47—N3	178.5 (11)
C75—C76—C77B—C78B	−179.0 (6)	N3—C43—C44—C45	−0.9 (11)
C75—C76—C77—C78	−48.0 (11)	C37—C38—C39—C40	1.7 (11)
C76—C77B—C78B—C79B	162.1 (7)	C37—C38—C39—C48	−178.8 (8)
C76—C77—C78—C79	−169.4 (8)	C37—C42—C43—N3	2.8 (11)
O1—C10—C11—N1	170.5 (6)	C37—C42—C43—C44	−174.1 (7)
C1—C2—C3—C4	−2.1 (13)	C38—C37—C42—C41	−3.2 (11)
C1—C2—C3—C12	177.5 (8)	C38—C37—C42—C43	174.4 (7)
C2—C1—C6—C7	−173.3 (7)	C38—C39—C40—C41	−3.0 (11)
C2—C1—C6—C5	−3.9 (13)	C38—C39—C40—C54	177.7 (7)
C2—C3—C4—C5	0.4 (13)	C38—C39—C48—C49	−0.4 (14)
C2—C3—C4—C18	179.1 (7)	C38—C39—C48—C53	−179.3 (8)
C2—C3—C12—C13	0.9 (16)	C39—C40—C41—C42	1.1 (11)
C2—C3—C12—C17	−177.8 (8)	C39—C40—C54—C53	2.7 (8)
C3—C4—C5—C6	−0.4 (12)	C39—C40—C54—C82	122.3 (7)
C3—C4—C18—C17	−0.8 (9)	C39—C40—C54—C88	−113.4 (8)
C3—C4—C18—C56	118.5 (8)	C39—C48—C49—C50	−179.0 (8)
C3—C4—C18—C62	−119.2 (7)	C39—C48—C53—C52	179.6 (7)
C3—C12—C13—C14	−179.5 (8)	C39—C48—C53—C54	1.5 (9)
C3—C12—C17—C16	178.2 (7)	C40—C39—C48—C49	179.2 (8)
C3—C12—C17—C18	−2.4 (10)	C40—C39—C48—C53	0.3 (9)
C4—C3—C12—C13	−179.5 (9)	C40—C41—C42—C37	2.0 (11)
C4—C3—C12—C17	1.8 (10)	C40—C41—C42—C43	−175.5 (7)
C4—C5—C6—C7	171.9 (6)	C40—C54—C82—C83	−62.8 (9)
C4—C5—C6—C1	2.3 (12)	C40—C54—C88—C89	61.0 (10)
C4—C18—C56—C57	−63.4 (8)	C41—C40—C54—C53	−176.7 (8)
C4—C18—C62—C63	56.0 (9)	C41—C40—C54—C82	−57.0 (11)
C5—C4—C18—C17	177.8 (8)	C41—C40—C54—C88	67.2 (11)
C5—C4—C18—C56	−62.9 (11)	C41—C42—C43—N3	−179.6 (8)
C5—C4—C18—C62	59.4 (11)	C41—C42—C43—C44	3.5 (12)
C6—C7—C8—C9	168.9 (5)	C42—C37—C38—C39	1.3 (11)
C6—C1—C2—C3	3.8 (13)	C42—C43—C44—C45	176.0 (7)
C12—C3—C4—C5	−179.3 (7)	C43—N3—C47—C46	1 (2)
C12—C3—C4—C18	−0.6 (10)	C43—C44—C45—C46	−0.3 (11)
C12—C13—C14—C15	2.8 (13)	C44—C45—C46—O3	−179.0 (8)
C12—C17—C18—C4	2.0 (9)	C44—C45—C46—C47	1.8 (13)
C12—C17—C18—C56	−116.1 (8)	C45—C46—C47—N3	−2.1 (19)
C12—C17—C18—C62	119.4 (7)	C47—N3—C43—C42	−176.6 (11)
C13—C12—C17—C16	−0.7 (13)	C47—N3—C43—C44	0.6 (15)
C13—C12—C17—C18	178.7 (7)	C48—C39—C40—C41	177.4 (7)
C13—C14—C15—C16	−3.2 (13)	C48—C39—C40—C54	−2.0 (9)
C14—C15—C16—C17	1.6 (13)	C48—C49—C50—C51	1.1 (12)
C15—C16—C17—C12	0.3 (12)	C48—C53—C54—C40	−2.5 (9)
C15—C16—C17—C18	−178.9 (8)	C48—C53—C54—C82	−121.1 (7)
C16—C17—C18—C4	−178.7 (8)	C48—C53—C54—C88	114.9 (7)
C16—C17—C18—C56	63.3 (11)	C49—C48—C53—C52	0.6 (12)
C16—C17—C18—C62	−61.3 (11)	C49—C48—C53—C54	−177.5 (7)
C17—C12—C13—C14	−0.9 (13)	C49—C50—C51—C52	−2.2 (13)
C17—C18—C56—C57	48.0 (9)	C50—C51—C52—C53	2.5 (14)
C17—C18—C62—C63	−54.9 (9)	C51—C52—C53—C48	−1.7 (13)
C18—C4—C5—C6	−178.9 (8)	C51—C52—C53—C54	176.1 (8)
C18—C56—C57—C58	169.7 (6)	C52—C53—C54—C40	179.6 (8)
C18—C62—C63—C64	−174.2 (6)	C52—C53—C54—C82	61.0 (11)
C56—C18—C62—C63	179.0 (7)	C52—C53—C54—C88	−63.0 (11)
C56—C57—C58—C59	−175.2 (7)	C53—C48—C49—C50	−0.3 (12)
C57—C58—C59—C60	−64.6 (10)	C53—C54—C82—C83	49.3 (9)
C58—C59—C60—C61	89.6 (9)	C53—C54—C88—C89	−49.3 (9)
C62—C18—C56—C57	173.8 (6)	C54—C40—C41—C42	−179.6 (7)
C62—C63—C64—C65	−64.3 (11)	C54—C82—C83—C84	163.0 (7)
C63—C64—C65—C66	−63.7 (12)	C54—C88—C89—C90	124.3 (9)
C64—C65—C66—C67	−171.8 (8)	C77—C78—C79—C80	178.7 (8)
C69—C36—C75—C76	−174.7 (5)	C81—O3—C46—C45	1.0 (13)
C69—C70—C71—C72	−172.5 (10)	C81—O3—C46—C47	−179.7 (10)
C70—C71—C72—C73	−174.8 (10)	C82—C54—C88—C89	−174.5 (7)
C71—C72—C73—C74	−170.1 (11)	C82—C83—C84—C85	−178.9 (8)
O1B—C10—C11—N1	−169.6 (5)	C83—C84—C85—C86	166.6 (9)
C1B—C2B—C3B—C4B	2.0 (13)	C84—C85—C86—C87	174.7 (9)
C1B—C2B—C3B—C12B	−178.3 (9)	C88—C54—C82—C83	172.6 (7)
C2B—C1B—C6B—C7	170.5 (7)	C88—C89—C90—C91	149.7 (10)
C2B—C1B—C6B—C5B	−0.5 (13)	C89—C90—C91—C92	−146.9 (10)
C2B—C3B—C4B—C5B	−2.4 (13)	C90—C91—C92—C93	−131.9 (12)
C2B—C3B—C4B—C18B	177.8 (7)	C1S—C2S—C3S—C4S	−169.3 (10)
C2B—C3B—C12B—C13B	1.8 (16)	C1S—C2S'—C3S'—C4S'	−169.0 (13)
C2B—C3B—C12B—C17B	−177.1 (9)	C2S—C3S—C4S—C5S	−143.5 (11)
C3B—C4B—C5B—C6B	1.3 (12)	C2S'—C3S'—C4S'—C5S'	−84.0 (17)

### Di-µ2-chlorido-bis{bis[2-(5-fluoropyridin-2-yl)-9,9-dihexyl-9H-fluoren-3-yl]iridium} pentane 0.3-solvate (II) Crystal data

**Table:**

[Ir_2_(C_30_H_35_FN)_4_Cl_2_]·0.3C_5_H_12_	*Z* = 2
*M**_r_* = 2191.30	*F*(000) = 2249
Triclinic, *P*1	*D*_x_ = 1.377 Mg m^−^^3^
*a* = 12.2744 (7) Å	Mo *K*α radiation, λ = 0.71073 Å
*b* = 17.6132 (10) Å	Cell parameters from 5349 reflections
*c* = 25.3966 (15) Å	θ = 2.3–25.3°
α = 105.119 (2)°	µ = 2.62 mm^−^^1^
β = 93.787 (2)°	*T* = 120 K
γ = 90.779 (2)°	Block, orange
*V* = 5286.2 (5) Å^3^	0.14 × 0.1 × 0.04 mm

### Di-µ2-chlorido-bis{bis[2-(5-fluoropyridin-2-yl)-9,9-dihexyl-9H-fluoren-3-yl]iridium} pentane 0.3-solvate (II) Data collection

**Table:**

Bruker SMART CCD 6000 diffractometer	18623 independent reflections
Radiation source: sealed X-ray tube	12096 reflections with *I* > 2σ(*I*)
Graphite monochromator	*R*_int_ = 0.065
Detector resolution: 5.6 pixels mm^-1^	θ_max_ = 25.0°, θ_min_ = 1.2°
ω scans	*h* = −14→14
Absorption correction: integration (SADABS; Krause *et al.*, 2015)	*k* = −20→20
*T*_min_ = 0.735, *T*_max_ = 0.927	*l* = −30→30
48264 measured reflections	

### Di-µ2-chlorido-bis{bis[2-(5-fluoropyridin-2-yl)-9,9-dihexyl-9H-fluoren-3-yl]iridium} pentane 0.3-solvate (II) Refinement

**Table:**

Refinement on *F*^2^	Primary atom site location: structure-invariant direct methods
Least-squares matrix: full	Secondary atom site location: difference Fourier map
*R*[*F*^2^ > 2σ(*F*^2^)] = 0.041	Hydrogen site location: mixed
*wR*(*F*^2^) = 0.103	H-atom parameters constrained
*S* = 0.95	*w* = 1/[σ^2^(*F*_o_^2^) + (0.0472*P*)^2^] where *P* = (*F*_o_^2^ + 2*F*_c_^2^)/3
18623 reflections	(Δ/σ)_max_ = 0.002
1342 parameters	Δρ_max_ = 1.39 e Å^−^^3^
2905 restraints	Δρ_min_ = −0.76 e Å^−^^3^

### Di-µ2-chlorido-bis{bis[2-(5-fluoropyridin-2-yl)-9,9-dihexyl-9H-fluoren-3-yl]iridium} pentane 0.3-solvate (II) Special details

**Table:**

Experimental. The data collection nominally covered full sphere of reciprocal space, by a combination of 4 runs of 600 and 1 run of 50 narrow-frame ω-scans (scan width 0.3° ω, 40s exposure), every run at a different φ and/or 2θ angle. Crystal to detector distance 4.84 cm.
Geometry. All esds (except the esd in the dihedral angle between two l.s. planes) are estimated using the full covariance matrix. The cell esds are taken into account individually in the estimation of esds in distances, angles and torsion angles; correlations between esds in cell parameters are only used when they are defined by crystal symmetry. An approximate (isotropic) treatment of cell esds is used for estimating esds involving l.s. planes.
Refinement. Intense disorder of n-hexyl chains, overlapping with the pentane molecule of crystallisation which has the occupancy of 0.3.

### Di-µ2-chlorido-bis{bis[2-(5-fluoropyridin-2-yl)-9,9-dihexyl-9H-fluoren-3-yl]iridium} pentane 0.3-solvate (II) Fractional atomic coordinates and isotropic or equivalent isotropic displacement parameters (Å^2^)

**Table:**

	*x*	*y*	*z*	*U*_iso_*/*U*_eq_	Occ. (<1)
Ir1	0.64404 (2)	0.36376 (2)	0.19910 (2)	0.03102 (8)	
Ir2	0.54578 (2)	0.24992 (2)	0.29310 (2)	0.03270 (8)	
Cl1	0.66078 (13)	0.36958 (9)	0.29922 (6)	0.0346 (4)	
Cl2	0.54538 (13)	0.23529 (9)	0.19162 (6)	0.0365 (4)	
F1	0.2878 (3)	0.4982 (2)	0.31979 (15)	0.0502 (10)	
F2	0.8500 (4)	0.0463 (3)	0.21338 (17)	0.0788 (15)	
F3	0.2160 (3)	0.4031 (2)	0.16942 (16)	0.0569 (11)	
F4	0.9910 (4)	0.2071 (3)	0.24544 (18)	0.0780 (15)	
N1	0.4023 (4)	0.3079 (3)	0.29679 (19)	0.0318 (12)	
N2	0.6790 (4)	0.1836 (3)	0.2967 (2)	0.0368 (13)	
N3	0.5051 (4)	0.4258 (3)	0.20334 (18)	0.0293 (11)	
N4	0.7826 (4)	0.3022 (3)	0.1836 (2)	0.0381 (13)	
C1	0.4431 (5)	0.1584 (4)	0.2827 (3)	0.0372 (15)	
C2	0.4671 (5)	0.0790 (4)	0.2742 (2)	0.0361 (14)	
H2	0.541059	0.063919	0.275666	0.043*	
C3	0.3836 (5)	0.0219 (4)	0.2637 (2)	0.0344 (14)	
C4	0.2739 (5)	0.0420 (4)	0.2615 (3)	0.0373 (14)	
C5	0.2476 (5)	0.1200 (4)	0.2713 (3)	0.0414 (16)	
H5	0.173341	0.134399	0.270668	0.050*	
C6	0.3312 (5)	0.1777 (4)	0.2822 (3)	0.0396 (15)	
C7	0.3116 (5)	0.2606 (4)	0.2912 (3)	0.0396 (15)	
C8	0.2095 (6)	0.2952 (4)	0.2956 (3)	0.0517 (19)	
H8	0.145714	0.262595	0.292166	0.062*	
C9	0.1989 (6)	0.3752 (4)	0.3049 (3)	0.0533 (19)	
H9	0.129545	0.398611	0.307147	0.064*	
C10	0.2929 (6)	0.4186 (4)	0.3106 (3)	0.0413 (15)	
C11	0.3940 (5)	0.3859 (4)	0.3079 (2)	0.0345 (14)	
H11	0.458001	0.418823	0.314023	0.041*	
C12	0.3874 (5)	−0.0641 (4)	0.2533 (2)	0.0374 (14)	
C13	0.4752 (5)	−0.1121 (4)	0.2537 (2)	0.0392 (15)	
H13	0.547919	−0.090953	0.259368	0.047*	
C14	0.4542 (6)	−0.1921 (4)	0.2454 (3)	0.0469 (17)	
H14	0.513275	−0.226119	0.246359	0.056*	
C15	0.3487 (6)	−0.2227 (4)	0.2359 (3)	0.0477 (17)	
H15	0.335952	−0.277579	0.230407	0.057*	
C16	0.2621 (6)	−0.1752 (4)	0.2341 (3)	0.0486 (18)	
H16	0.189630	−0.196769	0.226386	0.058*	
C17	0.2820 (6)	−0.0953 (4)	0.2437 (3)	0.0392 (15)	
C19	0.5649 (5)	0.2663 (4)	0.3739 (2)	0.0361 (15)	
C20	0.5102 (5)	0.3175 (4)	0.4140 (2)	0.0386 (15)	
H20	0.452150	0.346860	0.403475	0.046*	
C21	0.5386 (6)	0.3266 (4)	0.4686 (3)	0.0422 (16)	
C22	0.6223 (6)	0.2841 (4)	0.4854 (3)	0.0506 (18)	
C23	0.6810 (6)	0.2344 (4)	0.4468 (3)	0.054 (2)	
H23	0.739510	0.205897	0.457842	0.065*	
C24	0.6537 (6)	0.2267 (4)	0.3920 (3)	0.0466 (18)	
C25	0.7125 (6)	0.1782 (4)	0.3477 (3)	0.0504 (19)	
C26	0.7959 (7)	0.1282 (5)	0.3542 (3)	0.079 (3)	
H26	0.820772	0.125245	0.389775	0.095*	
C27	0.8427 (7)	0.0828 (6)	0.3091 (3)	0.081 (3)	
H27	0.898635	0.047501	0.313146	0.097*	
C28	0.8065 (6)	0.0897 (5)	0.2580 (3)	0.061 (2)	
C29	0.7246 (5)	0.1386 (4)	0.2518 (3)	0.0452 (17)	
H29	0.699135	0.141554	0.216303	0.054*	
C30	0.4935 (6)	0.3753 (4)	0.5176 (3)	0.0455 (16)	
C31	0.4090 (6)	0.4299 (4)	0.5229 (3)	0.0469 (17)	
H31	0.373580	0.442106	0.491830	0.056*	
C32	0.3800 (6)	0.4650 (4)	0.5753 (3)	0.0509 (18)	
H32	0.324773	0.503000	0.580377	0.061*	
C33	0.4295 (6)	0.4461 (4)	0.6200 (3)	0.0523 (18)	
H33	0.404521	0.468694	0.655171	0.063*	
C34	0.5137 (6)	0.3955 (4)	0.6152 (3)	0.0532 (19)	
H34	0.548834	0.384743	0.646842	0.064*	
C35	0.5477 (6)	0.3597 (4)	0.5634 (3)	0.0516 (18)	
C36	0.6384 (7)	0.3026 (4)	0.5477 (3)	0.0606 (19)	
C37	0.7103 (5)	0.4728 (4)	0.2191 (2)	0.0333 (14)	
C38	0.8207 (5)	0.4959 (4)	0.2298 (2)	0.0333 (14)	
H38	0.875149	0.457545	0.221596	0.040*	
C39	0.8513 (5)	0.5744 (4)	0.2522 (2)	0.0321 (13)	
C40	0.7728 (5)	0.6323 (4)	0.2649 (2)	0.0356 (14)	
C41	0.6632 (5)	0.6111 (4)	0.2538 (2)	0.0353 (14)	
H41	0.609309	0.649774	0.262333	0.042*	
C42	0.6322 (5)	0.5325 (4)	0.2301 (2)	0.0323 (13)	
C43	0.5184 (5)	0.5046 (4)	0.2168 (2)	0.0326 (14)	
C44	0.4306 (5)	0.5511 (4)	0.2123 (3)	0.0402 (16)	
H44	0.441343	0.606625	0.220849	0.048*	
C45	0.3274 (5)	0.5182 (4)	0.1955 (3)	0.0457 (17)	
H45	0.266492	0.549695	0.192006	0.055*	
C46	0.3170 (5)	0.4387 (4)	0.1842 (3)	0.0425 (15)	
C47	0.4038 (5)	0.3922 (4)	0.1874 (2)	0.0388 (15)	
H47	0.393614	0.336660	0.178452	0.047*	
C48	0.9588 (5)	0.6130 (4)	0.2682 (2)	0.0326 (13)	
C49	1.0619 (5)	0.5829 (4)	0.2650 (3)	0.0369 (15)	
H49	1.071729	0.528367	0.249895	0.044*	
C50	1.1525 (5)	0.6350 (4)	0.2846 (3)	0.0404 (15)	
H50	1.224306	0.615586	0.282469	0.049*	
C51	1.1375 (5)	0.7138 (4)	0.3069 (3)	0.0410 (15)	
H51	1.199287	0.748038	0.320514	0.049*	
C52	1.0339 (5)	0.7441 (4)	0.3097 (3)	0.0407 (15)	
H52	1.024465	0.798821	0.324577	0.049*	
C53	0.9449 (5)	0.6939 (4)	0.2907 (2)	0.0362 (14)	
C54	0.8250 (5)	0.7124 (4)	0.2907 (3)	0.0436 (15)	
C55	0.6405 (5)	0.3491 (4)	0.1176 (2)	0.0361 (15)	
C56	0.5656 (5)	0.3785 (4)	0.0855 (2)	0.0379 (15)	
H56	0.512315	0.413985	0.101879	0.045*	
C57	0.5692 (5)	0.3556 (4)	0.0291 (2)	0.0402 (16)	
C58	0.6478 (6)	0.3044 (4)	0.0041 (3)	0.0525 (19)	
C59	0.7243 (6)	0.2768 (4)	0.0354 (3)	0.055 (2)	
H59	0.778854	0.242764	0.018764	0.066*	
C60	0.7212 (5)	0.2993 (4)	0.0923 (3)	0.0443 (17)	
C61	0.8003 (6)	0.2740 (4)	0.1290 (3)	0.0457 (17)	
C62	0.8878 (7)	0.2270 (5)	0.1139 (3)	0.071 (3)	
H62	0.901631	0.209550	0.076366	0.085*	
C63	0.9553 (7)	0.2049 (5)	0.1521 (3)	0.073 (3)	
H63	1.015383	0.172193	0.141843	0.088*	
C64	0.9326 (6)	0.2316 (5)	0.2055 (3)	0.059 (2)	
C65	0.8486 (5)	0.2816 (4)	0.2216 (3)	0.0467 (17)	
H65	0.837442	0.301309	0.259316	0.056*	
C66	0.4987 (5)	0.3764 (4)	−0.0136 (2)	0.0394 (15)	
C67	0.4109 (5)	0.4254 (4)	−0.0093 (3)	0.0417 (16)	
H67	0.387171	0.451617	0.025496	0.050*	
C68	0.3576 (6)	0.4355 (4)	−0.0573 (3)	0.0474 (17)	
H68	0.297780	0.469478	−0.055589	0.057*	
C69	0.3930 (6)	0.3954 (4)	−0.1076 (3)	0.0492 (18)	
H69	0.356125	0.402447	−0.139937	0.059*	
C70	0.4781 (6)	0.3465 (4)	−0.1121 (3)	0.0498 (18)	
H70	0.499715	0.319041	−0.147062	0.060*	
C71	0.5330 (6)	0.3373 (4)	−0.0649 (3)	0.0451 (16)	
C72	0.6298 (6)	0.2863 (4)	−0.0584 (3)	0.0574 (18)	
C82	0.0930 (10)	−0.0673 (7)	0.4255 (4)	0.116 (4)	
H82C	0.027750	−0.086675	0.439677	0.139*	0.67
H82D	0.141652	−0.111889	0.413186	0.139*	0.67
H82A	0.020250	−0.051112	0.414048	0.139*	0.33
H82B	0.107566	−0.119647	0.401457	0.139*	0.33
C85	0.7518 (7)	0.3415 (5)	0.5686 (3)	0.072 (2)	
H85A	0.757472	0.352184	0.608996	0.087*	
H85B	0.807278	0.302461	0.554753	0.087*	
C86	0.7833 (6)	0.4178 (5)	0.5545 (3)	0.065 (2)	
H86A	0.778654	0.408744	0.514209	0.079*	
H86B	0.730941	0.458795	0.569434	0.079*	
C87	0.8986 (6)	0.4468 (5)	0.5775 (3)	0.076 (2)	
H87A	0.904995	0.449300	0.616978	0.091*	
H87B	0.950903	0.407959	0.559371	0.091*	
C88	0.9306 (6)	0.5260 (6)	0.5706 (3)	0.080 (3)	
H88A	0.877039	0.564918	0.587337	0.096*	
H88B	0.928573	0.523296	0.531089	0.096*	
C89	1.0455 (7)	0.5534 (6)	0.5971 (4)	0.094 (3)	
H89A	1.048696	0.551810	0.635832	0.113*	
H89B	1.099215	0.516193	0.578413	0.113*	
C90	1.0773 (8)	0.6353 (7)	0.5946 (4)	0.107 (4)	
H90A	1.071429	0.638003	0.556411	0.161*	
H90B	1.152732	0.648095	0.609869	0.161*	
H90C	1.028379	0.673165	0.615750	0.161*	
C91	0.6265 (8)	0.2301 (5)	0.5705 (3)	0.082 (3)	
H91A	0.686897	0.194476	0.558293	0.098*	
H91B	0.634704	0.247767	0.610900	0.098*	
C92	0.5211 (8)	0.1849 (5)	0.5537 (4)	0.088 (3)	
H92A	0.522596	0.157612	0.514382	0.105*	0.5
H92B	0.462821	0.223804	0.556654	0.105*	0.5
H92C	0.508664	0.166734	0.513390	0.105*	0.5
H92D	0.458686	0.216306	0.568849	0.105*	0.5
C97	0.7927 (6)	0.7465 (4)	0.3500 (3)	0.062 (2)	
H97A	0.825250	0.800349	0.363692	0.075*	
H97B	0.712328	0.751005	0.348991	0.075*	
C98	0.8263 (6)	0.7003 (5)	0.3903 (3)	0.068 (2)	
H98A	0.906902	0.698104	0.393534	0.081*	
H98B	0.796198	0.645730	0.376632	0.081*	
C99	0.7857 (8)	0.7372 (7)	0.4468 (3)	0.111 (4)	
H99A	0.804330	0.794232	0.457215	0.133*	
H99B	0.705112	0.730912	0.444528	0.133*	
C100	0.8326 (9)	0.7022 (8)	0.4909 (3)	0.126 (5)	
H10C	0.907227	0.684879	0.482090	0.151*	0.25
H10D	0.787935	0.654701	0.490350	0.151*	0.25
H10A	0.911814	0.715805	0.496234	0.151*	0.75
H10B	0.825266	0.644221	0.476801	0.151*	0.75
C103	0.7993 (6)	0.7717 (4)	0.2570 (3)	0.067 (2)	
H10E	0.719459	0.779000	0.255319	0.080*	
H10F	0.834842	0.822967	0.276603	0.080*	
C104	0.8352 (8)	0.7492 (5)	0.1991 (3)	0.081 (3)	
H10G	0.795489	0.699281	0.179994	0.097*	
H10H	0.913409	0.736691	0.201994	0.097*	
C109	0.6047 (7)	0.1989 (4)	−0.0844 (3)	0.069 (2)	
H10I	0.597821	0.190047	−0.124604	0.082*	
H10J	0.667859	0.168997	−0.075397	0.082*	
C110	0.5023 (8)	0.1651 (4)	−0.0672 (3)	0.078 (3)	
H11A	0.439683	0.195456	−0.076171	0.094*	0.6
H11B	0.509556	0.174610	−0.026970	0.094*	0.6
H11C	0.435461	0.183672	−0.083310	0.094*	0.4
H11D	0.500549	0.179057	−0.026943	0.094*	0.4
C115	0.7326 (6)	0.3095 (5)	−0.0835 (3)	0.068 (2)	
H11E	0.794181	0.278972	−0.073624	0.081*	
H11F	0.719449	0.292812	−0.123838	0.081*	
C116	0.7680 (6)	0.3947 (5)	−0.0675 (3)	0.072 (2)	
H11G	0.775317	0.415974	−0.027245	0.086*	0.6
H11H	0.716173	0.426711	−0.083429	0.086*	0.6
H11I	0.776472	0.410602	−0.027028	0.086*	0.4
H11J	0.705369	0.423726	−0.078153	0.086*	0.4
C18	0.1972 (7)	−0.0316 (5)	0.2410 (4)	0.032 (2)	0.67
C73	0.1407 (9)	−0.0413 (7)	0.1833 (4)	0.046 (3)	0.67
H73A	0.110500	−0.095873	0.169647	0.055*	0.67
H73B	0.078472	−0.005603	0.186409	0.055*	0.67
C74	0.2141 (10)	−0.0245 (7)	0.1410 (4)	0.063 (3)	0.67
H74A	0.196198	−0.062957	0.105268	0.075*	0.67
H74B	0.291419	−0.030717	0.152489	0.075*	0.67
C75A	0.1979 (11)	0.0630 (8)	0.1348 (6)	0.078 (4)	0.67
H75A	0.192894	0.098528	0.171807	0.093*	0.67
H75B	0.126896	0.063752	0.114093	0.093*	0.67
C76	0.2902 (13)	0.0991 (8)	0.1053 (6)	0.091 (4)	0.67
H76A	0.361282	0.102279	0.126724	0.109*	0.67
H76B	0.297749	0.064005	0.068421	0.109*	0.67
C77	0.2610 (13)	0.1803 (8)	0.1004 (6)	0.093 (5)	0.67
H77A	0.260904	0.216387	0.137566	0.112*	0.67
H77B	0.186135	0.177800	0.082838	0.112*	0.67
C78	0.3393 (13)	0.2139 (10)	0.0675 (6)	0.099 (6)	0.67
H78A	0.316268	0.266339	0.065725	0.148*	0.67
H78B	0.413315	0.217826	0.085110	0.148*	0.67
H78C	0.338498	0.179215	0.030347	0.148*	0.67
C79	0.1089 (7)	−0.0342 (6)	0.2806 (3)	0.031 (2)	0.67
H79A	0.061246	−0.081563	0.264753	0.037*	0.67
H79B	0.063206	0.012301	0.283291	0.037*	0.67
C80	0.1507 (8)	−0.0358 (7)	0.3389 (4)	0.044 (3)	0.67
H80A	0.189702	−0.085112	0.337086	0.052*	0.67
H80B	0.203617	0.008758	0.353973	0.052*	0.67
C81	0.0589 (9)	−0.0303 (8)	0.3774 (4)	0.070 (4)	0.67
H81A	−0.007525	−0.058240	0.356789	0.084*	0.67
H81B	0.041511	0.025594	0.392399	0.084*	0.67
C83	0.1498 (15)	−0.0046 (9)	0.4675 (6)	0.112 (6)	0.67
H83A	0.104971	0.042356	0.479226	0.134*	0.67
H83B	0.221265	0.010699	0.456761	0.134*	0.67
C84	0.1615 (19)	−0.0535 (17)	0.5115 (9)	0.230 (13)	0.67
H84A	0.199547	−0.021065	0.545126	0.345*	0.67
H84B	0.088734	−0.069302	0.519359	0.345*	0.67
H84C	0.203336	−0.100498	0.497221	0.345*	0.67
C93	0.4825 (12)	0.1203 (8)	0.5840 (6)	0.084 (4)*	0.67
H93A	0.473968	0.147361	0.622845	0.101*	0.67
H93B	0.409658	0.098578	0.567413	0.101*	0.67
C94	0.5575 (13)	0.0529 (9)	0.5820 (8)	0.120 (5)*	0.67
H94A	0.619080	0.069783	0.610031	0.144*	0.67
H94B	0.587816	0.037092	0.545700	0.144*	0.67
C95	0.4949 (12)	−0.0186 (8)	0.5925 (7)	0.096 (4)*	0.67
H95A	0.468081	−0.005052	0.629582	0.115*	0.67
H95B	0.431870	−0.035707	0.565290	0.115*	0.67
C96	0.5779 (12)	−0.0827 (9)	0.5867 (7)	0.110 (5)*	0.67
H96A	0.614493	−0.087680	0.552877	0.164*	0.67
H96B	0.540716	−0.132809	0.585495	0.164*	0.67
H96C	0.632181	−0.069131	0.618038	0.164*	0.67
C101	0.7860 (13)	0.7246 (9)	0.5482 (5)	0.115 (5)	0.75
H10K	0.705661	0.728350	0.544291	0.139*	0.75
H10L	0.803316	0.683814	0.567665	0.139*	0.75
C102	0.8367 (15)	0.8019 (8)	0.5797 (5)	0.139 (7)	0.75
H10M	0.808271	0.817139	0.616031	0.208*	0.75
H10N	0.916130	0.797571	0.583533	0.208*	0.75
H10O	0.818814	0.841951	0.560240	0.208*	0.75
C105	0.794 (3)	0.8133 (17)	0.1725 (10)	0.111 (7)	0.67
H10P	0.713450	0.816360	0.173865	0.134*	0.67
H10Q	0.826954	0.864942	0.192949	0.134*	0.67
C106	0.8227 (18)	0.7950 (13)	0.1140 (8)	0.143 (7)*	0.67
H10R	0.750720	0.788176	0.093022	0.171*	0.67
H10S	0.853620	0.845663	0.110720	0.171*	0.67
C107	0.890 (2)	0.7356 (15)	0.0790 (10)	0.230 (12)*	0.67
H10T	0.867467	0.683541	0.083316	0.276*	0.67
H10U	0.966718	0.745944	0.094436	0.276*	0.67
C108	0.890 (2)	0.7282 (15)	0.0180 (9)	0.203 (10)*	0.67
H10V	0.938834	0.686372	0.001511	0.305*	0.67
H10W	0.815853	0.715352	0.001040	0.305*	0.67
H10X	0.915748	0.778160	0.012233	0.305*	0.67
C111	0.4737 (16)	0.0780 (7)	−0.0919 (9)	0.059 (5)	0.6
H11K	0.530236	0.048138	−0.076999	0.071*	0.6
H11L	0.482060	0.066949	−0.131667	0.071*	0.6
C112	0.3628 (13)	0.0421 (8)	−0.0852 (7)	0.071 (4)	0.6
H11M	0.346641	0.062715	−0.046504	0.086*	0.6
H11N	0.307286	0.062984	−0.107385	0.086*	0.6
C113	0.345 (2)	−0.0456 (9)	−0.0997 (7)	0.081 (6)	0.6
H11O	0.380021	−0.069343	−0.133956	0.098*	0.6
H11P	0.265705	−0.058441	−0.106427	0.098*	0.6
C114	0.3912 (18)	−0.0812 (11)	−0.0551 (6)	0.085 (5)	0.6
H11Q	0.378859	−0.138391	−0.066555	0.128*	0.6
H11R	0.354792	−0.059350	−0.021462	0.128*	0.6
H11S	0.469777	−0.068785	−0.048369	0.128*	0.6
C117	0.8800 (12)	0.3923 (9)	−0.0922 (9)	0.064 (5)	0.6
H11T	0.930692	0.359371	−0.076474	0.077*	0.6
H11U	0.871775	0.370432	−0.132406	0.077*	0.6
C118	0.9217 (11)	0.4759 (9)	−0.0777 (8)	0.068 (5)	0.6
H11V	0.928766	0.496950	−0.037468	0.081*	0.6
H11W	0.869132	0.508208	−0.092901	0.081*	0.6
C119	1.0319 (12)	0.4810 (9)	−0.1005 (7)	0.081 (5)	0.6
H11X	1.025756	0.456671	−0.140440	0.097*	0.6
H11Y	1.085543	0.451415	−0.083521	0.097*	0.6
C120	1.0717 (17)	0.5660 (10)	−0.0893 (8)	0.088 (5)	0.6
H12A	1.033368	0.590737	−0.115241	0.132*	0.6
H12B	1.150447	0.567901	−0.093412	0.132*	0.6
H12C	1.057054	0.594365	−0.051892	0.132*	0.6
C96B	0.422 (3)	−0.0451 (19)	0.5954 (18)	0.160 (18)*	0.33
H96D	0.385303	−0.077240	0.615511	0.240*	0.33
H96E	0.422974	−0.074291	0.556922	0.240*	0.33
H96F	0.497369	−0.032492	0.611182	0.240*	0.33
C211	0.514 (3)	0.0767 (8)	−0.0906 (19)	0.080 (11)	0.4
H21A	0.501978	0.061528	−0.130926	0.096*	0.4
H21B	0.587921	0.060241	−0.080354	0.096*	0.4
C212	0.427 (2)	0.0405 (12)	−0.0644 (10)	0.074 (6)	0.4
H21C	0.448640	0.047594	−0.025221	0.089*	0.4
H21D	0.357564	0.068251	−0.067001	0.089*	0.4
C213	0.407 (2)	−0.0457 (13)	−0.0917 (14)	0.091 (10)	0.4
H21E	0.473320	−0.074643	−0.085155	0.109*	0.4
H21F	0.393822	−0.053597	−0.131616	0.109*	0.4
C214	0.310 (2)	−0.0784 (18)	−0.0695 (13)	0.098 (9)	0.4
H21G	0.242674	−0.070843	−0.090358	0.147*	0.4
H21H	0.305539	−0.050746	−0.030895	0.147*	0.4
H21I	0.318638	−0.134704	−0.073020	0.147*	0.4
C93B	0.539 (2)	0.1162 (14)	0.5789 (13)	0.084 (4)*	0.33
H93C	0.560997	0.134687	0.618558	0.101*	0.33
H93D	0.595574	0.081073	0.560567	0.101*	0.33
C94B	0.424 (2)	0.0746 (19)	0.5681 (14)	0.120 (5)*	0.33
H94C	0.373085	0.116767	0.564848	0.144*	0.33
H94D	0.423957	0.039076	0.530810	0.144*	0.33
C95B	0.364 (2)	0.0278 (17)	0.5994 (12)	0.096 (4)*	0.33
H95C	0.362354	0.057840	0.638175	0.115*	0.33
H95D	0.287637	0.015861	0.583680	0.115*	0.33
C201	0.839 (3)	0.756 (2)	0.5486 (10)	0.115 (5)	0.25
H20A	0.838947	0.725519	0.576122	0.139*	0.25
H20B	0.905632	0.790656	0.555732	0.139*	0.25
C202	0.741 (3)	0.803 (3)	0.5503 (19)	0.100 (14)*	0.25
H20C	0.738816	0.839751	0.586583	0.150*	0.25
H20D	0.741941	0.832647	0.522586	0.150*	0.25
H20E	0.675690	0.767934	0.542843	0.150*	0.25
C217	0.871 (2)	0.4282 (12)	−0.0873 (17)	0.070 (8)	0.4
H21J	0.934923	0.405642	−0.071781	0.084*	0.4
H21K	0.868407	0.406015	−0.127453	0.084*	0.4
C218	0.8967 (16)	0.5165 (12)	−0.0766 (12)	0.067 (6)	0.4
H21L	0.911211	0.537771	−0.036562	0.080*	0.4
H21M	0.829395	0.540899	−0.087127	0.080*	0.4
C219	0.9906 (15)	0.5457 (17)	−0.1038 (10)	0.080 (6)	0.4
H21N	0.972985	0.532488	−0.143852	0.096*	0.4
H21O	0.998720	0.603649	−0.090289	0.096*	0.4
C220	1.096 (2)	0.510 (2)	−0.0923 (12)	0.092 (10)	0.4
H22A	1.153503	0.527465	−0.111753	0.138*	0.4
H22B	1.087759	0.452138	−0.104578	0.138*	0.4
H22C	1.116432	0.525538	−0.052862	0.138*	0.4
C205	0.831 (6)	0.800 (4)	0.159 (3)	0.111 (7)	0.33
H20F	0.757340	0.789503	0.139110	0.134*	0.33
H20G	0.830100	0.855351	0.181608	0.134*	0.33
C206	0.909 (4)	0.802 (3)	0.1147 (18)	0.143 (7)*	0.33
H20H	0.981569	0.801510	0.133730	0.171*	0.33
H20I	0.897487	0.749500	0.089005	0.171*	0.33
C207	0.929 (5)	0.854 (3)	0.078 (2)	0.230 (12)*	0.33
H20J	0.860036	0.857961	0.055880	0.276*	0.33
H20K	0.951809	0.907378	0.099785	0.276*	0.33
C208	1.015 (4)	0.821 (3)	0.040 (2)	0.203 (10)*	0.33
H20L	1.027096	0.854746	0.016332	0.305*	0.33
H20M	1.082702	0.817222	0.062086	0.305*	0.33
H20N	0.991105	0.767900	0.018266	0.305*	0.33
C75B	0.062 (2)	−0.0416 (17)	0.1128 (9)	0.081 (8)*	0.33
H75C	0.003189	−0.080944	0.111364	0.098*	0.33
H75D	0.030889	0.010929	0.125062	0.098*	0.33
C78B	0.065 (2)	−0.0100 (18)	−0.0212 (11)	0.099 (10)*	0.33
H78D	0.016386	−0.003936	−0.052037	0.148*	0.33
H78E	0.092714	0.041892	0.000482	0.148*	0.33
H78F	0.125829	−0.042704	−0.034972	0.148*	0.33
C76B	0.093 (2)	−0.051 (2)	0.0573 (9)	0.105 (11)*	0.33
H76C	0.128418	−0.101906	0.045581	0.126*	0.33
H76D	0.148233	−0.009165	0.058092	0.126*	0.33
C77B	0.0030 (18)	−0.0478 (15)	0.0134 (9)	0.065 (7)*	0.33
H77C	−0.024925	−0.101109	−0.006919	0.079*	0.33
H77D	−0.058388	−0.015627	0.028907	0.079*	0.33
C73B	0.1165 (18)	−0.0344 (15)	0.2154 (9)	0.055 (6)*	0.33
H73C	0.075511	0.014831	0.223475	0.066*	0.33
H73D	0.064392	−0.077379	0.216273	0.066*	0.33
C74B	0.149 (2)	−0.049 (2)	0.1570 (9)	0.068 (9)*	0.33
H74C	0.176644	−0.103082	0.146082	0.081*	0.33
H74D	0.210155	−0.012378	0.156911	0.081*	0.33
C79B	0.1465 (19)	−0.0258 (16)	0.3146 (9)	0.044 (7)*	0.33
H79C	0.091717	0.015738	0.320414	0.053*	0.33
H79D	0.106866	−0.076752	0.309955	0.053*	0.33
C80B	0.224 (2)	−0.0111 (19)	0.3657 (10)	0.090 (9)*	0.33
H80C	0.265158	0.038685	0.368447	0.108*	0.33
H80D	0.277646	−0.053432	0.358958	0.108*	0.33
C81B	0.183 (3)	−0.006 (2)	0.4223 (12)	0.109 (11)*	0.33
H81C	0.154924	0.047647	0.436378	0.131*	0.33
H81D	0.246982	−0.010687	0.447107	0.131*	0.33
C18B	0.2000 (13)	−0.0277 (9)	0.2614 (8)	0.040 (7)*	0.33
C83B	0.097 (5)	−0.070 (3)	0.4842 (9)	0.220 (17)	0.33
H83C	0.173621	−0.065669	0.499617	0.264*	0.33
H83D	0.056737	−0.025869	0.505862	0.264*	0.33
C84B	0.046 (2)	−0.1468 (19)	0.4873 (11)	0.112 (11)	0.33
H84D	0.048349	−0.148942	0.525555	0.167*	0.33
H84E	−0.030222	−0.150926	0.472325	0.167*	0.33
H84F	0.086362	−0.190624	0.466095	0.167*	0.33
C1S	0.1557 (19)	0.3347 (15)	0.0220 (10)	0.055 (7)*	0.3
H1SA	0.183661	0.367368	−0.000338	0.083*	0.3
H1SB	0.101026	0.296424	−0.000072	0.083*	0.3
H1SC	0.122049	0.368267	0.053413	0.083*	0.3
C2S	0.250 (2)	0.2911 (17)	0.0423 (12)	0.080 (9)*	0.3
H2SA	0.284880	0.257675	0.010666	0.096*	0.3
H2SB	0.305946	0.329665	0.064261	0.096*	0.3
C3S	0.208 (3)	0.240 (2)	0.0769 (15)	0.107 (12)*	0.3
H3SA	0.142194	0.208400	0.058969	0.128*	0.3
H3SB	0.191658	0.272115	0.113744	0.128*	0.3
C4S	0.308 (4)	0.189 (3)	0.079 (2)	0.13 (2)*	0.3
H4SA	0.313263	0.146002	0.045551	0.155*	0.3
H4SB	0.378179	0.220241	0.088549	0.155*	0.3
C5S	0.268 (4)	0.161 (3)	0.1272 (16)	0.112 (15)*	0.3
H5SA	0.320485	0.125298	0.137508	0.167*	0.3
H5SB	0.260611	0.206706	0.158421	0.167*	0.3
H5SC	0.196782	0.133710	0.116143	0.167*	0.3

### Di-µ2-chlorido-bis{bis[2-(5-fluoropyridin-2-yl)-9,9-dihexyl-9H-fluoren-3-yl]iridium} pentane 0.3-solvate (II) Atomic displacement parameters (Å^2^)

**Table:**

	*U*^11^	*U*^22^	*U*^33^	*U*^12^	*U*^13^	*U*^23^
Ir1	0.02894 (15)	0.03413 (16)	0.02566 (14)	0.00551 (12)	−0.00019 (11)	0.00046 (11)
Ir2	0.03295 (15)	0.03058 (15)	0.03104 (15)	0.00763 (12)	0.00348 (12)	0.00119 (12)
Cl1	0.0407 (9)	0.0321 (9)	0.0266 (8)	0.0021 (7)	−0.0021 (7)	0.0010 (7)
Cl2	0.0359 (9)	0.0345 (9)	0.0322 (8)	0.0026 (7)	−0.0007 (7)	−0.0027 (7)
F1	0.063 (3)	0.035 (2)	0.051 (2)	0.0159 (19)	0.007 (2)	0.0071 (18)
F2	0.080 (3)	0.096 (4)	0.052 (3)	0.058 (3)	0.013 (2)	0.000 (2)
F3	0.032 (2)	0.074 (3)	0.060 (3)	0.002 (2)	−0.0080 (19)	0.012 (2)
F4	0.061 (3)	0.093 (4)	0.068 (3)	0.040 (3)	−0.022 (2)	0.004 (3)
N1	0.037 (3)	0.028 (3)	0.028 (3)	0.010 (2)	0.006 (2)	0.002 (2)
N2	0.034 (3)	0.040 (3)	0.032 (3)	0.013 (2)	0.004 (2)	0.003 (2)
N3	0.030 (3)	0.036 (3)	0.018 (2)	0.007 (2)	0.001 (2)	−0.001 (2)
N4	0.033 (3)	0.040 (3)	0.036 (3)	0.005 (2)	−0.001 (2)	0.002 (2)
C1	0.033 (3)	0.045 (3)	0.034 (3)	0.012 (3)	0.008 (3)	0.010 (3)
C2	0.036 (3)	0.040 (3)	0.035 (3)	0.009 (3)	0.009 (3)	0.014 (3)
C3	0.038 (3)	0.037 (3)	0.029 (3)	0.011 (2)	0.011 (3)	0.008 (3)
C4	0.038 (3)	0.038 (3)	0.038 (4)	0.009 (3)	0.007 (3)	0.012 (3)
C5	0.032 (3)	0.038 (3)	0.057 (4)	0.011 (3)	0.014 (3)	0.014 (3)
C6	0.037 (3)	0.038 (3)	0.046 (4)	0.012 (3)	0.010 (3)	0.012 (3)
C7	0.035 (3)	0.035 (3)	0.046 (4)	0.008 (3)	0.008 (3)	0.005 (3)
C8	0.036 (4)	0.042 (4)	0.071 (5)	0.010 (3)	0.008 (4)	0.003 (4)
C9	0.044 (4)	0.046 (4)	0.066 (5)	0.016 (3)	0.002 (4)	0.006 (4)
C10	0.052 (4)	0.032 (3)	0.037 (4)	0.013 (3)	0.004 (3)	0.004 (3)
C11	0.043 (3)	0.029 (3)	0.031 (3)	0.007 (3)	0.003 (3)	0.005 (3)
C12	0.047 (3)	0.036 (3)	0.030 (3)	0.006 (3)	0.010 (3)	0.009 (3)
C13	0.042 (4)	0.038 (3)	0.035 (4)	0.009 (3)	0.012 (3)	0.003 (3)
C14	0.053 (4)	0.040 (4)	0.052 (4)	0.023 (3)	0.015 (4)	0.015 (3)
C15	0.062 (4)	0.031 (4)	0.054 (4)	0.009 (3)	0.019 (4)	0.016 (3)
C16	0.052 (4)	0.037 (3)	0.061 (5)	0.006 (3)	0.017 (4)	0.017 (4)
C17	0.047 (3)	0.035 (3)	0.039 (4)	0.007 (3)	0.016 (3)	0.013 (3)
C19	0.037 (4)	0.037 (4)	0.032 (3)	0.012 (3)	0.007 (3)	0.003 (3)
C20	0.050 (4)	0.031 (4)	0.035 (3)	0.010 (3)	0.010 (3)	0.007 (3)
C21	0.055 (4)	0.039 (4)	0.033 (3)	0.009 (3)	0.012 (3)	0.008 (3)
C22	0.061 (5)	0.059 (5)	0.032 (3)	0.021 (4)	0.008 (3)	0.010 (3)
C23	0.059 (5)	0.064 (5)	0.037 (3)	0.024 (4)	0.000 (3)	0.009 (3)
C24	0.055 (4)	0.051 (4)	0.035 (3)	0.026 (3)	0.008 (3)	0.012 (3)
C25	0.057 (5)	0.057 (5)	0.036 (3)	0.029 (4)	0.006 (3)	0.008 (3)
C26	0.077 (6)	0.109 (7)	0.044 (4)	0.059 (5)	−0.001 (4)	0.006 (4)
C27	0.071 (6)	0.115 (8)	0.055 (4)	0.064 (6)	0.011 (4)	0.016 (5)
C28	0.058 (5)	0.078 (6)	0.042 (4)	0.037 (4)	0.010 (4)	0.002 (4)
C29	0.041 (4)	0.052 (4)	0.036 (3)	0.015 (3)	0.006 (3)	−0.003 (3)
C30	0.056 (4)	0.042 (4)	0.035 (3)	0.005 (3)	0.014 (3)	0.002 (3)
C31	0.064 (5)	0.037 (4)	0.039 (3)	0.006 (3)	0.015 (3)	0.007 (3)
C32	0.065 (5)	0.037 (4)	0.044 (4)	0.004 (4)	0.018 (3)	−0.004 (3)
C33	0.069 (5)	0.044 (4)	0.041 (4)	0.000 (3)	0.024 (4)	0.001 (3)
C34	0.074 (5)	0.048 (4)	0.035 (3)	0.001 (4)	0.014 (4)	0.003 (3)
C35	0.063 (5)	0.053 (4)	0.040 (3)	0.011 (4)	0.015 (3)	0.009 (3)
C36	0.089 (5)	0.065 (5)	0.030 (3)	0.031 (4)	0.015 (4)	0.013 (3)
C37	0.038 (3)	0.035 (3)	0.025 (3)	0.006 (3)	0.002 (3)	0.003 (3)
C38	0.036 (3)	0.038 (3)	0.026 (3)	0.011 (3)	0.004 (3)	0.007 (3)
C39	0.034 (3)	0.037 (3)	0.027 (3)	0.001 (2)	0.000 (3)	0.011 (3)
C40	0.039 (3)	0.037 (3)	0.030 (3)	0.005 (3)	−0.002 (3)	0.008 (3)
C41	0.034 (3)	0.037 (3)	0.033 (3)	0.008 (3)	0.000 (3)	0.006 (3)
C42	0.033 (3)	0.037 (3)	0.027 (3)	0.004 (2)	0.000 (3)	0.010 (3)
C43	0.035 (3)	0.038 (3)	0.022 (3)	0.007 (3)	0.001 (3)	0.002 (3)
C44	0.037 (3)	0.043 (4)	0.038 (4)	0.012 (3)	0.000 (3)	0.007 (3)
C45	0.033 (3)	0.058 (4)	0.042 (4)	0.016 (3)	−0.003 (3)	0.008 (3)
C46	0.034 (3)	0.061 (4)	0.031 (3)	0.005 (3)	−0.001 (3)	0.010 (3)
C47	0.033 (3)	0.049 (4)	0.028 (3)	0.007 (3)	0.000 (3)	0.000 (3)
C48	0.033 (3)	0.038 (3)	0.029 (3)	0.003 (3)	0.002 (3)	0.013 (3)
C49	0.035 (3)	0.037 (4)	0.042 (4)	0.003 (3)	0.003 (3)	0.016 (3)
C50	0.034 (3)	0.048 (4)	0.042 (4)	−0.001 (3)	0.004 (3)	0.014 (3)
C51	0.039 (3)	0.047 (4)	0.040 (4)	−0.006 (3)	−0.006 (3)	0.020 (3)
C52	0.047 (4)	0.035 (4)	0.039 (4)	0.003 (3)	−0.006 (3)	0.008 (3)
C53	0.040 (3)	0.039 (3)	0.031 (3)	0.005 (3)	0.003 (3)	0.012 (3)
C54	0.035 (3)	0.039 (3)	0.050 (4)	0.004 (3)	−0.006 (3)	0.002 (3)
C55	0.026 (3)	0.039 (4)	0.036 (3)	0.006 (3)	0.003 (3)	−0.003 (3)
C56	0.036 (4)	0.046 (4)	0.027 (3)	0.008 (3)	0.004 (3)	0.001 (3)
C57	0.042 (4)	0.046 (4)	0.029 (3)	0.005 (3)	0.001 (3)	0.003 (3)
C58	0.060 (5)	0.065 (5)	0.030 (3)	0.021 (4)	0.011 (3)	0.005 (3)
C59	0.060 (5)	0.070 (5)	0.034 (3)	0.026 (4)	0.013 (3)	0.007 (3)
C60	0.038 (4)	0.055 (4)	0.035 (3)	0.018 (3)	0.010 (3)	0.003 (3)
C61	0.044 (4)	0.053 (4)	0.037 (3)	0.019 (3)	0.009 (3)	0.005 (3)
C62	0.070 (5)	0.091 (6)	0.049 (4)	0.049 (5)	0.015 (4)	0.010 (4)
C63	0.054 (5)	0.095 (7)	0.062 (4)	0.044 (5)	0.000 (4)	0.002 (5)
C64	0.043 (4)	0.072 (6)	0.057 (4)	0.018 (4)	−0.014 (4)	0.010 (4)
C65	0.038 (4)	0.048 (4)	0.045 (4)	0.006 (3)	−0.011 (3)	−0.001 (3)
C66	0.042 (4)	0.046 (4)	0.028 (3)	−0.002 (3)	0.002 (3)	0.006 (3)
C67	0.043 (4)	0.050 (4)	0.031 (3)	0.001 (3)	0.001 (3)	0.008 (3)
C68	0.041 (4)	0.060 (5)	0.042 (3)	−0.005 (3)	−0.007 (3)	0.018 (3)
C69	0.056 (4)	0.059 (5)	0.033 (3)	−0.009 (3)	−0.009 (3)	0.015 (3)
C70	0.064 (5)	0.055 (5)	0.028 (3)	−0.003 (3)	0.005 (3)	0.005 (3)
C71	0.053 (4)	0.048 (4)	0.033 (3)	0.000 (3)	0.010 (3)	0.006 (3)
C72	0.068 (5)	0.071 (5)	0.031 (3)	0.017 (4)	0.014 (3)	0.006 (3)
C82	0.127 (10)	0.159 (11)	0.058 (6)	0.002 (8)	0.009 (6)	0.019 (6)
C85	0.083 (5)	0.098 (6)	0.032 (4)	0.035 (4)	0.001 (4)	0.010 (4)
C86	0.054 (4)	0.096 (6)	0.039 (4)	0.024 (4)	0.002 (4)	0.004 (4)
C87	0.055 (5)	0.126 (7)	0.038 (4)	0.035 (4)	0.002 (4)	0.006 (5)
C88	0.058 (5)	0.127 (7)	0.047 (5)	0.020 (5)	0.006 (4)	0.006 (5)
C89	0.055 (5)	0.149 (9)	0.066 (6)	0.029 (5)	−0.003 (5)	0.005 (7)
C90	0.069 (7)	0.170 (10)	0.075 (7)	0.001 (7)	0.010 (6)	0.015 (8)
C91	0.127 (8)	0.066 (5)	0.049 (5)	0.024 (5)	−0.007 (5)	0.010 (4)
C92	0.119 (8)	0.069 (6)	0.070 (6)	0.017 (5)	0.002 (6)	0.008 (5)
C97	0.043 (4)	0.056 (5)	0.067 (4)	0.005 (4)	0.008 (4)	−0.021 (3)
C98	0.050 (5)	0.090 (6)	0.047 (4)	−0.006 (4)	0.010 (4)	−0.012 (4)
C99	0.080 (7)	0.162 (11)	0.061 (5)	−0.017 (7)	0.025 (5)	−0.028 (6)
C100	0.089 (8)	0.222 (13)	0.043 (5)	−0.032 (8)	0.018 (5)	−0.011 (6)
C103	0.055 (5)	0.034 (4)	0.106 (5)	−0.002 (4)	−0.031 (5)	0.019 (4)
C104	0.107 (7)	0.053 (5)	0.085 (5)	−0.029 (5)	−0.045 (5)	0.037 (5)
C109	0.103 (6)	0.067 (4)	0.032 (4)	0.024 (4)	0.015 (4)	0.003 (4)
C110	0.127 (8)	0.052 (4)	0.056 (5)	0.012 (5)	0.026 (5)	0.010 (4)
C115	0.063 (5)	0.105 (6)	0.030 (4)	0.013 (4)	0.013 (4)	0.007 (4)
C116	0.053 (5)	0.118 (6)	0.039 (4)	−0.005 (5)	0.014 (4)	0.009 (5)
C18	0.043 (5)	0.036 (5)	0.020 (5)	0.003 (3)	0.006 (4)	0.010 (4)
C73	0.066 (7)	0.048 (7)	0.024 (5)	0.005 (5)	−0.004 (5)	0.014 (5)
C74	0.072 (8)	0.082 (8)	0.039 (6)	0.032 (7)	0.012 (6)	0.023 (6)
C75A	0.082 (9)	0.084 (8)	0.074 (9)	0.017 (7)	0.011 (7)	0.030 (7)
C76	0.107 (11)	0.102 (10)	0.066 (9)	−0.011 (9)	0.016 (9)	0.024 (9)
C77	0.129 (14)	0.084 (9)	0.063 (10)	−0.060 (9)	−0.021 (9)	0.024 (8)
C78	0.126 (14)	0.108 (12)	0.041 (8)	−0.063 (12)	0.010 (8)	−0.016 (7)
C79	0.030 (5)	0.038 (5)	0.025 (4)	0.005 (4)	−0.002 (4)	0.009 (4)
C80	0.044 (6)	0.067 (8)	0.018 (5)	0.027 (6)	0.007 (4)	0.007 (5)
C81	0.074 (8)	0.096 (10)	0.042 (6)	0.022 (7)	0.034 (6)	0.014 (6)
C83	0.147 (16)	0.106 (12)	0.092 (10)	−0.004 (11)	0.000 (10)	0.044 (8)
C84	0.21 (3)	0.43 (3)	0.131 (14)	0.20 (2)	0.069 (14)	0.19 (2)
C101	0.099 (12)	0.157 (13)	0.073 (6)	−0.028 (10)	0.045 (7)	−0.009 (7)
C102	0.215 (19)	0.111 (12)	0.055 (8)	0.042 (11)	0.008 (11)	−0.039 (8)
C105	0.15 (3)	0.079 (13)	0.116 (16)	−0.001 (11)	−0.030 (11)	0.055 (14)
C111	0.090 (13)	0.054 (6)	0.033 (8)	0.030 (6)	0.013 (10)	0.007 (7)
C112	0.097 (11)	0.048 (7)	0.059 (10)	0.013 (8)	0.006 (9)	−0.005 (7)
C113	0.119 (16)	0.048 (7)	0.064 (10)	0.020 (9)	−0.015 (13)	−0.006 (8)
C114	0.119 (15)	0.070 (11)	0.066 (10)	0.003 (12)	−0.002 (11)	0.019 (9)
C117	0.056 (8)	0.098 (12)	0.044 (10)	0.008 (8)	0.006 (7)	0.025 (11)
C118	0.039 (8)	0.109 (12)	0.070 (11)	0.000 (8)	−0.006 (7)	0.053 (12)
C119	0.035 (7)	0.150 (14)	0.071 (11)	−0.017 (10)	−0.013 (8)	0.056 (11)
C120	0.053 (11)	0.144 (14)	0.074 (12)	−0.018 (11)	0.006 (10)	0.043 (13)
C211	0.10 (2)	0.058 (8)	0.08 (2)	0.018 (11)	0.010 (18)	0.013 (12)
C212	0.084 (17)	0.049 (9)	0.081 (17)	0.004 (11)	−0.018 (13)	0.008 (11)
C213	0.12 (2)	0.049 (11)	0.09 (2)	0.000 (14)	0.003 (19)	0.006 (13)
C214	0.12 (2)	0.075 (18)	0.10 (2)	−0.025 (17)	−0.025 (18)	0.037 (17)
C201	0.099 (12)	0.157 (13)	0.073 (6)	−0.028 (10)	0.045 (7)	−0.009 (7)
C217	0.033 (11)	0.094 (15)	0.08 (2)	0.020 (12)	0.028 (13)	0.013 (18)
C218	0.030 (10)	0.102 (15)	0.073 (15)	0.008 (10)	−0.003 (10)	0.032 (15)
C219	0.050 (12)	0.116 (19)	0.070 (15)	−0.003 (12)	0.010 (11)	0.017 (14)
C220	0.043 (13)	0.16 (3)	0.065 (16)	0.010 (17)	0.004 (12)	0.01 (2)
C205	0.15 (3)	0.079 (13)	0.116 (16)	−0.001 (11)	−0.030 (11)	0.055 (14)
C83B	0.25 (4)	0.33 (3)	0.164 (19)	0.05 (3)	0.08 (2)	0.20 (3)
C84B	0.10 (2)	0.20 (3)	0.056 (17)	0.10 (2)	0.037 (16)	0.062 (19)

### Di-µ2-chlorido-bis{bis[2-(5-fluoropyridin-2-yl)-9,9-dihexyl-9H-fluoren-3-yl]iridium} pentane 0.3-solvate (II) Geometric parameters (Å, º)

**Table:**

Ir1—Cl1	2.5123 (15)	C116—H11G	0.9900
Ir1—Cl2	2.5088 (16)	C116—H11H	0.9900
Ir1—N3	2.031 (5)	C116—H11I	0.9900
Ir1—N4	2.034 (5)	C116—H11J	0.9900
Ir1—C37	2.001 (6)	C116—C117	1.545 (11)
Ir1—C55	2.015 (6)	C116—C217	1.555 (13)
Ir2—Cl1	2.4854 (16)	C18—C73	1.546 (10)
Ir2—Cl2	2.5218 (15)	C18—C79	1.536 (9)
Ir2—N1	2.043 (5)	C18—C73B	1.14 (2)
Ir2—N2	2.034 (5)	C18—C79B	1.99 (2)
Ir2—C1	1.983 (7)	C18—C18B	0.504 (19)
Ir2—C19	1.994 (6)	C73—H73A	0.9900
F1—C10	1.364 (7)	C73—H73B	0.9900
F2—C28	1.339 (7)	C73—C74	1.529 (11)
F3—C46	1.365 (7)	C73—C75B	1.97 (2)
F4—C64	1.368 (8)	C73—C73B	0.86 (2)
N1—C7	1.358 (8)	C73—C74B	0.66 (2)
N1—C11	1.335 (7)	C73—C18B	2.02 (2)
N2—C25	1.359 (8)	C74—H74A	0.9900
N2—C29	1.366 (7)	C74—H74B	0.9900
N3—C43	1.345 (7)	C74—C75A	1.604 (16)
N3—C47	1.360 (7)	C74—C75B	1.94 (3)
N4—C61	1.377 (8)	C74—C74B	1.06 (3)
N4—C65	1.345 (8)	C75A—H75A	0.9900
C1—C2	1.398 (8)	C75A—H75B	0.9900
C1—C6	1.419 (8)	C75A—C76	1.609 (18)
C2—H2	0.9500	C76—H76A	0.9900
C2—C3	1.389 (8)	C76—H76B	0.9900
C3—C4	1.399 (8)	C76—C77	1.513 (12)
C3—C12	1.470 (8)	C77—H77A	0.9900
C4—C5	1.377 (8)	C77—H77B	0.9900
C4—C18	1.545 (10)	C77—C78	1.525 (13)
C4—C18B	1.516 (16)	C78—H78A	0.9800
C5—H5	0.9500	C78—H78B	0.9800
C5—C6	1.396 (9)	C78—H78C	0.9800
C6—C7	1.445 (9)	C79—H79A	0.9900
C7—C8	1.400 (9)	C79—H79B	0.9900
C8—H8	0.9500	C79—C80	1.540 (9)
C8—C9	1.375 (9)	C79—C73B	1.66 (2)
C9—H9	0.9500	C79—C79B	0.93 (2)
C9—C10	1.354 (9)	C79—C18B	1.265 (17)
C10—C11	1.373 (9)	C80—H80A	0.9900
C11—H11	0.9500	C80—H80B	0.9900
C12—C13	1.379 (8)	C80—C81	1.528 (10)
C12—C17	1.380 (9)	C80—C79B	0.68 (2)
C13—H13	0.9500	C80—C80B	1.10 (3)
C13—C14	1.387 (9)	C81—H81A	0.9900
C14—H14	0.9500	C81—H81B	0.9900
C14—C15	1.378 (10)	C81—C79B	2.00 (2)
C15—H15	0.9500	C81—C81B	1.82 (4)
C15—C16	1.368 (9)	C83—H83A	0.9900
C16—H16	0.9500	C83—H83B	0.9900
C16—C17	1.380 (9)	C83—C84	1.579 (12)
C17—C18	1.551 (10)	C83—C81B	1.24 (3)
C17—C18B	1.563 (16)	C83—C83B	1.49 (4)
C19—C20	1.388 (8)	C84—H84A	0.9800
C19—C24	1.417 (9)	C84—H84B	0.9800
C20—H20	0.9500	C84—H84C	0.9800
C20—C21	1.375 (9)	C84—C83B	1.01 (5)
C21—C22	1.388 (9)	C93—H93A	0.9900
C21—C30	1.461 (8)	C93—H93B	0.9900
C22—C23	1.383 (9)	C93—C94	1.505 (12)
C22—C36	1.527 (9)	C94—H94A	0.9900
C23—H23	0.9500	C94—H94B	0.9900
C23—C24	1.382 (9)	C94—C95	1.556 (12)
C24—C25	1.462 (9)	C95—H95A	0.9900
C25—C26	1.389 (9)	C95—H95B	0.9900
C26—H26	0.9500	C95—C96	1.516 (12)
C26—C27	1.379 (10)	C96—H96A	0.9800
C27—H27	0.9500	C96—H96B	0.9800
C27—C28	1.382 (10)	C96—H96C	0.9800
C28—C29	1.359 (9)	C101—H10K	0.9900
C29—H29	0.9500	C101—H10L	0.9900
C30—C31	1.414 (9)	C101—C102	1.492 (12)
C30—C35	1.393 (10)	C102—H10M	0.9800
C31—H31	0.9500	C102—H10N	0.9800
C31—C32	1.386 (9)	C102—H10O	0.9800
C32—H32	0.9500	C105—H10P	0.9900
C32—C33	1.372 (10)	C105—H10Q	0.9900
C33—H33	0.9500	C105—C106	1.504 (13)
C33—C34	1.364 (10)	C106—H10R	0.9900
C34—H34	0.9500	C106—H10S	0.9900
C34—C35	1.398 (9)	C106—C107	1.484 (11)
C35—C36	1.513 (10)	C107—H10T	0.9900
C36—C85	1.542 (9)	C107—H10U	0.9900
C36—C91	1.542 (9)	C107—C108	1.520 (11)
C37—C38	1.399 (8)	C108—H10V	0.9800
C37—C42	1.418 (8)	C108—H10W	0.9800
C38—H38	0.9500	C108—H10X	0.9800
C38—C39	1.387 (8)	C111—H11K	0.9900
C39—C40	1.403 (8)	C111—H11L	0.9900
C39—C48	1.460 (8)	C111—C112	1.530 (12)
C40—C41	1.382 (8)	C112—H11M	0.9900
C40—C54	1.506 (9)	C112—H11N	0.9900
C41—H41	0.9500	C112—C113	1.501 (12)
C41—C42	1.394 (8)	C113—H11O	0.9900
C42—C43	1.463 (8)	C113—H11P	0.9900
C43—C44	1.379 (8)	C113—C114	1.514 (12)
C44—H44	0.9500	C114—H11Q	0.9800
C44—C45	1.379 (9)	C114—H11R	0.9800
C45—H45	0.9500	C114—H11S	0.9800
C45—C46	1.356 (9)	C117—H11T	0.9900
C46—C47	1.363 (9)	C117—H11U	0.9900
C47—H47	0.9500	C117—C118	1.497 (12)
C48—C49	1.378 (8)	C118—H11V	0.9900
C48—C53	1.407 (8)	C118—H11W	0.9900
C49—H49	0.9500	C118—C119	1.516 (12)
C49—C50	1.409 (8)	C119—H11X	0.9900
C50—H50	0.9500	C119—H11Y	0.9900
C50—C51	1.375 (9)	C119—C120	1.517 (13)
C51—H51	0.9500	C120—H12A	0.9800
C51—C52	1.385 (9)	C120—H12B	0.9800
C52—H52	0.9500	C120—H12C	0.9800
C52—C53	1.375 (9)	C96B—H96D	0.9800
C53—C54	1.512 (9)	C96B—H96E	0.9800
C54—C97	1.549 (8)	C96B—H96F	0.9800
C54—C103	1.535 (8)	C96B—C95B	1.461 (14)
C55—C56	1.383 (8)	C211—H21A	0.9900
C55—C60	1.406 (8)	C211—H21B	0.9900
C56—H56	0.9500	C211—C212	1.519 (14)
C56—C57	1.388 (8)	C212—H21C	0.9900
C57—C58	1.398 (9)	C212—H21D	0.9900
C57—C66	1.465 (9)	C212—C213	1.504 (14)
C58—C59	1.366 (9)	C213—H21E	0.9900
C58—C72	1.535 (9)	C213—H21F	0.9900
C59—H59	0.9500	C213—C214	1.522 (14)
C59—C60	1.398 (9)	C214—H21G	0.9800
C60—C61	1.457 (9)	C214—H21H	0.9800
C61—C62	1.379 (9)	C214—H21I	0.9800
C62—H62	0.9500	C93B—H93C	0.9900
C62—C63	1.371 (10)	C93B—H93D	0.9900
C63—H63	0.9500	C93B—C94B	1.566 (14)
C63—C64	1.361 (10)	C94B—H94C	0.9900
C64—C65	1.378 (9)	C94B—H94D	0.9900
C65—H65	0.9500	C94B—C95B	1.500 (14)
C66—C67	1.383 (9)	C95B—H95C	0.9900
C66—C71	1.401 (8)	C95B—H95D	0.9900
C67—H67	0.9500	C201—H20A	0.9900
C67—C68	1.400 (9)	C201—H20B	0.9900
C68—H68	0.9500	C201—C202	1.46 (2)
C68—C69	1.388 (9)	C202—H20C	0.9800
C69—H69	0.9500	C202—H20D	0.9800
C69—C70	1.354 (10)	C202—H20E	0.9800
C70—H70	0.9500	C217—H21J	0.9900
C70—C71	1.383 (9)	C217—H21K	0.9900
C71—C72	1.525 (10)	C217—C218	1.530 (13)
C72—C109	1.526 (8)	C218—H21L	0.9900
C72—C115	1.548 (8)	C218—H21M	0.9900
C82—H82C	0.9900	C218—C219	1.533 (13)
C82—H82D	0.9900	C219—H21N	0.9900
C82—H82A	0.9900	C219—H21O	0.9900
C82—H82B	0.9900	C219—C220	1.496 (14)
C82—C81	1.565 (11)	C220—H22A	0.9800
C82—C83	1.453 (12)	C220—H22B	0.9800
C82—C81B	1.57 (3)	C220—H22C	0.9800
C82—C83B	1.502 (14)	C205—H20F	0.9900
C85—H85A	0.9900	C205—H20G	0.9900
C85—H85B	0.9900	C205—C206	1.527 (15)
C85—C86	1.530 (9)	C206—H20H	0.9900
C86—H86A	0.9900	C206—H20I	0.9900
C86—H86B	0.9900	C206—C207	1.49 (6)
C86—C87	1.523 (9)	C207—H20J	0.9900
C87—H87A	0.9900	C207—H20K	0.9900
C87—H87B	0.9900	C207—C208	1.49 (2)
C87—C88	1.500 (9)	C208—H20L	0.9800
C88—H88A	0.9900	C208—H20M	0.9800
C88—H88B	0.9900	C208—H20N	0.9800
C88—C89	1.537 (9)	C75B—H75C	0.9900
C89—H89A	0.9900	C75B—H75D	0.9900
C89—H89B	0.9900	C75B—C76B	1.450 (18)
C89—C90	1.509 (10)	C75B—C74B	1.523 (18)
C90—H90A	0.9800	C78B—H78D	0.9800
C90—H90B	0.9800	C78B—H78E	0.9800
C90—H90C	0.9800	C78B—H78F	0.9800
C91—H91A	0.9900	C78B—C77B	1.471 (8)
C91—H91B	0.9900	C76B—H76C	0.9900
C91—C92	1.488 (9)	C76B—H76D	0.9900
C92—H92A	0.9900	C76B—C77B	1.525 (13)
C92—H92B	0.9900	C77B—H77C	0.9900
C92—H92C	0.9900	C77B—H77D	0.9900
C92—H92D	0.9900	C73B—H73C	0.9900
C92—C93	1.613 (11)	C73B—H73D	0.9900
C92—C93B	1.522 (13)	C73B—C74B	1.517 (18)
C97—H97A	0.9900	C73B—C18B	1.483 (17)
C97—H97B	0.9900	C74B—H74C	0.9900
C97—C98	1.507 (9)	C74B—H74D	0.9900
C98—H98A	0.9900	C79B—H79C	0.9900
C98—H98B	0.9900	C79B—H79D	0.9900
C98—C99	1.532 (8)	C79B—C80B	1.521 (18)
C99—H99A	0.9900	C79B—C18B	1.533 (17)
C99—H99B	0.9900	C80B—H80C	0.9900
C99—C100	1.499 (11)	C80B—H80D	0.9900
C100—H10C	0.9900	C80B—C81B	1.530 (18)
C100—H10D	0.9900	C81B—H81C	0.9900
C100—H10A	0.9900	C81B—H81D	0.9900
C100—H10B	0.9900	C83B—H83C	0.9900
C100—C101	1.555 (12)	C83B—H83D	0.9900
C100—C201	1.523 (12)	C83B—C84B	1.501 (15)
C103—H10E	0.9900	C84B—H84D	0.9800
C103—H10F	0.9900	C84B—H84E	0.9800
C103—C104	1.516 (9)	C84B—H84F	0.9800
C104—H10G	0.9903	C1S—H1SA	0.9800
C104—H10H	0.9900	C1S—H1SB	0.9800
C104—C105	1.537 (12)	C1S—H1SC	0.9800
C104—C205	1.534 (14)	C1S—C2S	1.53 (2)
C109—H10I	0.9900	C2S—H2SA	0.9900
C109—H10J	0.9900	C2S—H2SB	0.9900
C109—C110	1.520 (9)	C2S—C3S	1.52 (3)
C110—H11A	0.9900	C3S—H3SA	0.9900
C110—H11B	0.9900	C3S—H3SB	0.9900
C110—H11C	0.9900	C3S—C4S	1.55 (3)
C110—H11D	0.9900	C4S—H4SA	0.9900
C110—C111	1.526 (11)	C4S—H4SB	0.9900
C110—C211	1.528 (14)	C4S—C5S	1.54 (3)
C115—H11E	0.9900	C5S—H5SA	0.9800
C115—H11F	0.9900	C5S—H5SB	0.9800
C115—C116	1.499 (9)	C5S—H5SC	0.9800
			
Cl2—Ir1—Cl1	83.11 (5)	C74B—C74—C75A	116 (2)
N3—Ir1—Cl1	94.59 (13)	C74B—C74—C75B	51.4 (14)
N3—Ir1—Cl2	94.04 (15)	C74—C75A—H75A	108.0
N3—Ir1—N4	171.84 (19)	C74—C75A—H75B	108.0
N4—Ir1—Cl1	93.53 (15)	C74—C75A—C76	117.2 (10)
N4—Ir1—Cl2	87.70 (15)	H75A—C75A—H75B	107.2
C37—Ir1—Cl1	87.52 (17)	C76—C75A—H75A	108.0
C37—Ir1—Cl2	168.90 (17)	C76—C75A—H75B	108.0
C37—Ir1—N3	80.7 (2)	C75A—C76—H76A	109.5
C37—Ir1—N4	98.9 (2)	C75A—C76—H76B	109.5
C37—Ir1—C55	95.6 (2)	H76A—C76—H76B	108.0
C55—Ir1—Cl1	173.97 (18)	C77—C76—C75A	110.9 (12)
C55—Ir1—Cl2	94.28 (19)	C77—C76—H76A	109.5
C55—Ir1—N3	91.0 (2)	C77—C76—H76B	109.5
C55—Ir1—N4	80.9 (2)	C76—C77—H77A	108.9
Cl1—Ir2—Cl2	83.39 (5)	C76—C77—H77B	108.9
N1—Ir2—Cl1	94.21 (15)	C76—C77—C78	113.2 (14)
N1—Ir2—Cl2	91.21 (14)	H77A—C77—H77B	107.7
N2—Ir2—Cl1	92.18 (16)	C78—C77—H77A	108.9
N2—Ir2—Cl2	94.92 (14)	C78—C77—H77B	108.9
N2—Ir2—N1	171.6 (2)	C77—C78—H78A	109.5
C1—Ir2—Cl1	173.83 (18)	C77—C78—H78B	109.5
C1—Ir2—Cl2	92.49 (18)	C77—C78—H78C	109.5
C1—Ir2—N1	81.3 (2)	H78A—C78—H78B	109.5
C1—Ir2—N2	92.7 (2)	H78A—C78—H78C	109.5
C1—Ir2—C19	93.4 (3)	H78B—C78—H78C	109.5
C19—Ir2—Cl1	91.01 (19)	C18—C79—H79A	108.3
C19—Ir2—Cl2	173.02 (19)	C18—C79—H79B	108.3
C19—Ir2—N1	93.3 (2)	C18—C79—C80	115.8 (7)
C19—Ir2—N2	81.1 (2)	C18—C79—C73B	41.6 (8)
Ir2—Cl1—Ir1	96.88 (5)	H79A—C79—H79B	107.4
Ir1—Cl2—Ir2	96.04 (5)	C80—C79—H79A	108.3
C7—N1—Ir2	114.3 (4)	C80—C79—H79B	108.3
C11—N1—Ir2	125.1 (4)	C80—C79—C73B	157.3 (10)
C11—N1—C7	120.4 (5)	C73B—C79—H79A	83.2
C25—N2—Ir2	114.9 (4)	C73B—C79—H79B	85.6
C25—N2—C29	120.5 (5)	C79B—C79—C18	104.9 (15)
C29—N2—Ir2	124.0 (4)	C79B—C79—H79A	122.0
C43—N3—Ir1	116.2 (4)	C79B—C79—H79B	105.2
C43—N3—C47	119.6 (5)	C79B—C79—C80	14.3 (16)
C47—N3—Ir1	123.6 (4)	C79B—C79—C73B	145.9 (17)
C61—N4—Ir1	115.1 (4)	C79B—C79—C18B	87.4 (15)
C65—N4—Ir1	124.8 (4)	C18B—C79—C18	17.6 (9)
C65—N4—C61	119.7 (6)	C18B—C79—H79A	119.9
C2—C1—Ir2	128.4 (5)	C18B—C79—H79B	113.4
C2—C1—C6	117.3 (6)	C18B—C79—C80	98.6 (11)
C6—C1—Ir2	114.3 (5)	C18B—C79—C73B	58.9 (9)
C1—C2—H2	119.8	C79—C80—H80A	109.0
C3—C2—C1	120.4 (6)	C79—C80—H80B	109.0
C3—C2—H2	119.8	H80A—C80—H80B	107.8
C2—C3—C4	121.3 (6)	C81—C80—C79	112.8 (8)
C2—C3—C12	130.8 (6)	C81—C80—H80A	109.0
C4—C3—C12	108.0 (6)	C81—C80—H80B	109.0
C3—C4—C18	111.6 (6)	C79B—C80—C79	20 (2)
C3—C4—C18B	110.6 (8)	C79B—C80—H80A	113.4
C5—C4—C3	119.6 (6)	C79B—C80—H80B	89.8
C5—C4—C18	128.4 (6)	C79B—C80—C81	125 (2)
C5—C4—C18B	128.1 (8)	C79B—C80—C80B	115 (3)
C18B—C4—C18	18.9 (7)	C80B—C80—C79	134.1 (17)
C4—C5—H5	120.3	C80B—C80—H80A	81.0
C4—C5—C6	119.4 (6)	C80B—C80—H80B	30.8
C6—C5—H5	120.3	C80B—C80—C81	104.9 (16)
C1—C6—C7	114.7 (6)	C82—C81—H81A	109.5
C5—C6—C1	122.0 (6)	C82—C81—H81B	109.5
C5—C6—C7	123.3 (6)	C82—C81—C79B	127.0 (11)
N1—C7—C6	115.5 (5)	C82—C81—C81B	54.4 (11)
N1—C7—C8	118.6 (6)	C80—C81—C82	110.8 (9)
C8—C7—C6	125.9 (6)	C80—C81—H81A	109.5
C7—C8—H8	119.1	C80—C81—H81B	109.5
C9—C8—C7	121.8 (7)	C80—C81—C79B	16.3 (8)
C9—C8—H8	119.1	C80—C81—C81B	75.1 (10)
C8—C9—H9	121.9	H81A—C81—H81B	108.1
C10—C9—C8	116.3 (7)	C79B—C81—H81A	99.1
C10—C9—H9	121.9	C79B—C81—H81B	102.3
F1—C10—C11	118.2 (6)	C81B—C81—H81A	162.8
C9—C10—F1	119.0 (6)	C81B—C81—H81B	85.0
C9—C10—C11	122.8 (6)	C81B—C81—C79B	88.6 (11)
N1—C11—C10	120.0 (6)	C82—C83—H83A	112.7
N1—C11—H11	120.0	C82—C83—H83B	112.7
C10—C11—H11	120.0	C82—C83—C84	95.0 (13)
C13—C12—C3	130.4 (6)	C82—C83—C83B	61.4 (11)
C13—C12—C17	120.8 (6)	H83A—C83—H83B	110.2
C17—C12—C3	108.8 (6)	C84—C83—H83A	112.7
C12—C13—H13	120.9	C84—C83—H83B	112.7
C12—C13—C14	118.1 (6)	C81B—C83—C82	70.5 (18)
C14—C13—H13	120.9	C81B—C83—H83A	108.4
C13—C14—H14	119.6	C81B—C83—H83B	47.9
C15—C14—C13	120.7 (6)	C81B—C83—C84	139 (3)
C15—C14—H14	119.7	C81B—C83—C83B	128 (2)
C14—C15—H15	119.5	C83B—C83—H83A	107.7
C16—C15—C14	121.0 (6)	C83B—C83—H83B	140.1
C16—C15—H15	119.5	C83B—C83—C84	38.1 (18)
C15—C16—H16	120.7	C83—C84—H84A	109.5
C15—C16—C17	118.7 (7)	C83—C84—H84B	109.5
C17—C16—H16	120.7	C83—C84—H84C	109.5
C12—C17—C18	111.9 (6)	H84A—C84—H84B	109.5
C12—C17—C18B	109.3 (8)	H84A—C84—H84C	109.5
C16—C17—C12	120.7 (6)	H84B—C84—H84C	109.5
C16—C17—C18	127.2 (7)	C83B—C84—C83	66.1 (19)
C16—C17—C18B	127.8 (8)	C83B—C84—H84A	154.4
C18—C17—C18B	18.6 (7)	C83B—C84—H84B	53.9
C20—C19—Ir2	128.1 (5)	C83B—C84—H84C	95.3
C20—C19—C24	116.9 (6)	C92—C93—H93A	108.3
C24—C19—Ir2	114.6 (4)	C92—C93—H93B	108.3
C19—C20—H20	119.4	H93A—C93—H93B	107.4
C21—C20—C19	121.2 (6)	C94—C93—C92	115.8 (11)
C21—C20—H20	119.4	C94—C93—H93A	108.3
C20—C21—C22	120.9 (6)	C94—C93—H93B	108.3
C20—C21—C30	131.3 (6)	C93—C94—H94A	109.5
C22—C21—C30	107.8 (6)	C93—C94—H94B	109.5
C21—C22—C36	111.9 (6)	C93—C94—C95	110.7 (12)
C23—C22—C21	119.7 (6)	H94A—C94—H94B	108.1
C23—C22—C36	128.4 (7)	C95—C94—H94A	109.5
C22—C23—H23	120.4	C95—C94—H94B	109.5
C24—C23—C22	119.2 (7)	C94—C95—H95A	110.7
C24—C23—H23	120.4	C94—C95—H95B	110.7
C19—C24—C25	114.0 (6)	H95A—C95—H95B	108.8
C23—C24—C19	122.0 (6)	C96—C95—C94	105.0 (12)
C23—C24—C25	124.0 (6)	C96—C95—H95A	110.7
N2—C25—C24	115.1 (6)	C96—C95—H95B	110.7
N2—C25—C26	119.6 (6)	C95—C96—H96A	109.5
C26—C25—C24	125.3 (6)	C95—C96—H96B	109.5
C25—C26—H26	119.9	C95—C96—H96C	109.5
C27—C26—C25	120.3 (7)	H96A—C96—H96B	109.5
C27—C26—H26	119.9	H96A—C96—H96C	109.5
C26—C27—H27	120.8	H96B—C96—H96C	109.5
C26—C27—C28	118.4 (7)	C100—C101—H10K	110.1
C28—C27—H27	120.8	C100—C101—H10L	110.1
F2—C28—C27	119.9 (6)	H10K—C101—H10L	108.4
F2—C28—C29	119.0 (7)	C102—C101—C100	108.2 (10)
C29—C28—C27	121.0 (7)	C102—C101—H10K	110.1
N2—C29—H29	120.0	C102—C101—H10L	110.1
C28—C29—N2	120.1 (6)	C101—C102—H10M	109.5
C28—C29—H29	120.0	C101—C102—H10N	109.5
C31—C30—C21	130.3 (7)	C101—C102—H10O	109.5
C35—C30—C21	108.6 (6)	H10M—C102—H10N	109.5
C35—C30—C31	121.1 (6)	H10M—C102—H10O	109.5
C30—C31—H31	121.4	H10N—C102—H10O	109.5
C32—C31—C30	117.2 (7)	C104—C105—H10P	109.6
C32—C31—H31	121.4	C104—C105—H10Q	109.6
C31—C32—H32	119.3	H10P—C105—H10Q	108.1
C33—C32—C31	121.4 (7)	C106—C105—C104	110.3 (15)
C33—C32—H32	119.3	C106—C105—H10P	109.6
C32—C33—H33	119.2	C106—C105—H10Q	109.6
C34—C33—C32	121.5 (7)	C105—C106—H10R	103.4
C34—C33—H33	119.2	C105—C106—H10S	103.4
C33—C34—H34	120.3	H10R—C106—H10S	105.2
C33—C34—C35	119.4 (7)	C107—C106—C105	135.1 (19)
C35—C34—H34	120.3	C107—C106—H10R	103.4
C30—C35—C34	119.3 (7)	C107—C106—H10S	103.4
C30—C35—C36	111.5 (6)	C106—C107—H10T	107.2
C34—C35—C36	129.2 (7)	C106—C107—H10U	107.2
C22—C36—C85	111.2 (6)	C106—C107—C108	120.5 (19)
C22—C36—C91	114.1 (6)	H10T—C107—H10U	106.8
C35—C36—C22	100.0 (6)	C108—C107—H10T	107.2
C35—C36—C85	111.7 (6)	C108—C107—H10U	107.2
C35—C36—C91	112.2 (7)	C107—C108—H10V	109.5
C85—C36—C91	107.6 (7)	C107—C108—H10W	109.5
C38—C37—Ir1	128.2 (5)	C107—C108—H10X	109.5
C38—C37—C42	117.7 (6)	H10V—C108—H10W	109.5
C42—C37—Ir1	113.6 (4)	H10V—C108—H10X	109.5
C37—C38—H38	119.8	H10W—C108—H10X	109.5
C39—C38—C37	120.4 (6)	C110—C111—H11K	106.9
C39—C38—H38	119.8	C110—C111—H11L	106.9
C38—C39—C40	121.1 (6)	C110—C111—C112	121.6 (11)
C38—C39—C48	131.1 (6)	H11K—C111—H11L	106.7
C40—C39—C48	107.8 (5)	C112—C111—H11K	106.9
C39—C40—C54	111.6 (5)	C112—C111—H11L	106.9
C41—C40—C39	119.6 (6)	C111—C112—H11M	107.3
C41—C40—C54	128.9 (6)	C111—C112—H11N	107.3
C40—C41—H41	120.2	H11M—C112—H11N	106.9
C40—C41—C42	119.5 (6)	C113—C112—C111	120.2 (14)
C42—C41—H41	120.2	C113—C112—H11M	107.3
C37—C42—C43	114.9 (5)	C113—C112—H11N	107.3
C41—C42—C37	121.6 (6)	C112—C113—H11O	109.1
C41—C42—C43	123.4 (6)	C112—C113—H11P	109.1
N3—C43—C42	113.8 (5)	C112—C113—C114	112.3 (16)
N3—C43—C44	120.3 (6)	H11O—C113—H11P	107.9
C44—C43—C42	125.6 (6)	C114—C113—H11O	109.1
C43—C44—H44	119.5	C114—C113—H11P	109.1
C43—C44—C45	121.0 (6)	C113—C114—H11Q	109.5
C45—C44—H44	119.5	C113—C114—H11R	109.5
C44—C45—H45	121.6	C113—C114—H11S	109.5
C46—C45—C44	116.8 (6)	H11Q—C114—H11R	109.5
C46—C45—H45	121.6	H11Q—C114—H11S	109.5
C45—C46—F3	119.3 (6)	H11R—C114—H11S	109.5
C45—C46—C47	122.5 (7)	C116—C117—H11T	110.7
C47—C46—F3	118.1 (6)	C116—C117—H11U	110.7
N3—C47—C46	119.7 (6)	H11T—C117—H11U	108.8
N3—C47—H47	120.2	C118—C117—C116	105.4 (11)
C46—C47—H47	120.2	C118—C117—H11T	110.7
C49—C48—C39	130.9 (6)	C118—C117—H11U	110.7
C49—C48—C53	120.5 (6)	C117—C118—H11V	109.6
C53—C48—C39	108.6 (5)	C117—C118—H11W	109.6
C48—C49—H49	120.8	C117—C118—C119	110.2 (12)
C48—C49—C50	118.4 (6)	H11V—C118—H11W	108.1
C50—C49—H49	120.8	C119—C118—H11V	109.6
C49—C50—H50	119.8	C119—C118—H11W	109.6
C51—C50—C49	120.4 (6)	C118—C119—H11X	109.5
C51—C50—H50	119.8	C118—C119—H11Y	109.5
C50—C51—H51	119.4	C118—C119—C120	110.7 (15)
C50—C51—C52	121.1 (6)	H11X—C119—H11Y	108.1
C52—C51—H51	119.4	C120—C119—H11X	109.5
C51—C52—H52	120.5	C120—C119—H11Y	109.5
C53—C52—C51	119.0 (6)	C119—C120—H12A	109.5
C53—C52—H52	120.5	C119—C120—H12B	109.5
C48—C53—C54	110.7 (6)	C119—C120—H12C	109.5
C52—C53—C48	120.5 (6)	H12A—C120—H12B	109.5
C52—C53—C54	128.7 (6)	H12A—C120—H12C	109.5
C40—C54—C53	101.4 (5)	H12B—C120—H12C	109.5
C40—C54—C97	112.0 (6)	H96D—C96B—H96E	109.5
C40—C54—C103	112.8 (5)	H96D—C96B—H96F	109.5
C53—C54—C97	110.3 (5)	H96E—C96B—H96F	109.5
C53—C54—C103	110.6 (6)	C95B—C96B—H96D	109.5
C103—C54—C97	109.5 (6)	C95B—C96B—H96E	109.5
C56—C55—Ir1	127.3 (4)	C95B—C96B—H96F	109.5
C56—C55—C60	119.0 (6)	C110—C211—H21A	111.1
C60—C55—Ir1	113.6 (5)	C110—C211—H21B	111.1
C55—C56—H56	120.4	H21A—C211—H21B	109.0
C55—C56—C57	119.2 (6)	C212—C211—C110	103.4 (16)
C57—C56—H56	120.4	C212—C211—H21A	111.1
C56—C57—C58	121.4 (6)	C212—C211—H21B	111.1
C56—C57—C66	129.9 (6)	C211—C212—H21C	109.3
C58—C57—C66	108.6 (6)	C211—C212—H21D	109.3
C57—C58—C72	110.4 (6)	H21C—C212—H21D	107.9
C59—C58—C57	119.9 (6)	C213—C212—C211	111.8 (17)
C59—C58—C72	129.7 (6)	C213—C212—H21C	109.3
C58—C59—H59	120.4	C213—C212—H21D	109.3
C58—C59—C60	119.1 (6)	C212—C213—H21E	109.5
C60—C59—H59	120.4	C212—C213—H21F	109.5
C55—C60—C61	115.7 (6)	C212—C213—C214	111 (2)
C59—C60—C55	121.2 (6)	H21E—C213—H21F	108.1
C59—C60—C61	123.0 (6)	C214—C213—H21E	109.5
N4—C61—C60	114.0 (5)	C214—C213—H21F	109.5
N4—C61—C62	119.8 (6)	C213—C214—H21G	109.5
C62—C61—C60	126.2 (6)	C213—C214—H21H	109.5
C61—C62—H62	119.4	C213—C214—H21I	109.5
C63—C62—C61	121.1 (7)	H21G—C214—H21H	109.5
C63—C62—H62	119.4	H21G—C214—H21I	109.5
C62—C63—H63	121.3	H21H—C214—H21I	109.5
C64—C63—C62	117.3 (7)	C92—C93B—H93C	111.5
C64—C63—H63	121.3	C92—C93B—H93D	111.5
F4—C64—C65	117.0 (7)	C92—C93B—C94B	101.4 (16)
C63—C64—F4	120.6 (7)	H93C—C93B—H93D	109.3
C63—C64—C65	122.4 (7)	C94B—C93B—H93C	111.5
N4—C65—C64	119.6 (6)	C94B—C93B—H93D	111.5
N4—C65—H65	120.2	C93B—C94B—H94C	104.4
C64—C65—H65	120.2	C93B—C94B—H94D	104.4
C67—C66—C57	130.2 (6)	H94C—C94B—H94D	105.6
C67—C66—C71	120.7 (6)	C95B—C94B—C93B	132 (3)
C71—C66—C57	109.1 (6)	C95B—C94B—H94C	104.4
C66—C67—H67	120.8	C95B—C94B—H94D	104.4
C66—C67—C68	118.5 (6)	C96B—C95B—C94B	107 (2)
C68—C67—H67	120.8	C96B—C95B—H95C	110.3
C67—C68—H68	120.3	C96B—C95B—H95D	110.3
C69—C68—C67	119.4 (7)	C94B—C95B—H95C	110.3
C69—C68—H68	120.3	C94B—C95B—H95D	110.3
C68—C69—H69	118.8	H95C—C95B—H95D	108.5
C70—C69—C68	122.4 (7)	C100—C201—H20A	110.8
C70—C69—H69	118.8	C100—C201—H20B	110.8
C69—C70—H70	120.6	H20A—C201—H20B	108.9
C69—C70—C71	118.8 (6)	C202—C201—C100	105 (3)
C71—C70—H70	120.6	C202—C201—H20A	110.8
C66—C71—C72	110.3 (6)	C202—C201—H20B	110.8
C70—C71—C66	120.2 (7)	C201—C202—H20C	109.5
C70—C71—C72	129.5 (6)	C201—C202—H20D	109.5
C58—C72—C115	110.9 (6)	C201—C202—H20E	109.5
C71—C72—C58	101.5 (5)	H20C—C202—H20D	109.5
C71—C72—C109	112.7 (6)	H20C—C202—H20E	109.5
C71—C72—C115	112.5 (6)	H20D—C202—H20E	109.5
C109—C72—C58	111.7 (6)	C116—C217—H21J	106.6
C109—C72—C115	107.6 (6)	C116—C217—H21K	106.6
H82C—C82—H82D	108.7	H21J—C217—H21K	106.5
H82C—C82—H82A	61.7	C218—C217—C116	123.1 (15)
H82C—C82—H82B	93.7	C218—C217—H21J	106.6
H82D—C82—H82A	137.4	C218—C217—H21K	106.6
H82D—C82—H82B	28.8	C217—C218—H21L	107.2
H82A—C82—H82B	108.7	C217—C218—H21M	107.2
C81—C82—H82C	110.6	C217—C218—C219	120 (2)
C81—C82—H82D	110.6	H21L—C218—H21M	106.9
C81—C82—H82A	50.4	C219—C218—H21L	107.2
C81—C82—H82B	94.7	C219—C218—H21M	107.2
C81—C82—C81B	71.2 (14)	C218—C219—H21N	109.3
C83—C82—H82C	110.6	C218—C219—H21O	109.3
C83—C82—H82D	110.6	H21N—C219—H21O	107.9
C83—C82—H82A	111.5	C220—C219—C218	112 (2)
C83—C82—H82B	139.4	C220—C219—H21N	109.3
C83—C82—C81	105.7 (11)	C220—C219—H21O	109.3
C83—C82—C81B	48.5 (12)	C219—C220—H22A	109.5
C83—C82—C83B	60.4 (16)	C219—C220—H22B	109.5
C81B—C82—H82C	155.7	C219—C220—H22C	109.5
C81B—C82—H82D	92.3	H22A—C220—H22B	109.5
C81B—C82—H82A	110.5	H22A—C220—H22C	109.5
C81B—C82—H82B	110.5	H22B—C220—H22C	109.5
C83B—C82—H82C	60.6	C104—C205—H20F	105.3
C83B—C82—H82D	96.0	C104—C205—H20G	105.3
C83B—C82—H82A	110.5	H20F—C205—H20G	106.0
C83B—C82—H82B	110.5	C206—C205—C104	128 (4)
C83B—C82—C81	153 (2)	C206—C205—H20F	105.3
C83B—C82—C81B	106 (2)	C206—C205—H20G	105.3
C36—C85—H85A	107.6	C205—C206—H20H	103.1
C36—C85—H85B	107.6	C205—C206—H20I	103.1
H85A—C85—H85B	107.1	H20H—C206—H20I	105.1
C86—C85—C36	118.8 (6)	C207—C206—C205	136 (4)
C86—C85—H85A	107.6	C207—C206—H20H	103.1
C86—C85—H85B	107.6	C207—C206—H20I	103.1
C85—C86—H86A	109.3	C206—C207—H20J	109.6
C85—C86—H86B	109.3	C206—C207—H20K	109.6
H86A—C86—H86B	108.0	H20J—C207—H20K	108.2
C87—C86—C85	111.5 (7)	C208—C207—C206	110 (5)
C87—C86—H86A	109.3	C208—C207—H20J	109.6
C87—C86—H86B	109.3	C208—C207—H20K	109.6
C86—C87—H87A	108.6	C207—C208—H20L	109.5
C86—C87—H87B	108.6	C207—C208—H20M	109.5
H87A—C87—H87B	107.6	C207—C208—H20N	109.5
C88—C87—C86	114.5 (7)	H20L—C208—H20M	109.5
C88—C87—H87A	108.6	H20L—C208—H20N	109.5
C88—C87—H87B	108.6	H20M—C208—H20N	109.5
C87—C88—H88A	109.3	C73—C75B—H75C	100.2
C87—C88—H88B	109.3	C73—C75B—H75D	96.7
C87—C88—C89	111.8 (8)	C74—C75B—C73	46.0 (7)
H88A—C88—H88B	107.9	C74—C75B—H75C	136.6
C89—C88—H88A	109.3	C74—C75B—H75D	103.8
C89—C88—H88B	109.3	H75C—C75B—H75D	106.9
C88—C89—H89A	108.9	C76B—C75B—C73	135.6 (19)
C88—C89—H89B	108.9	C76B—C75B—C74	91.5 (16)
H89A—C89—H89B	107.7	C76B—C75B—H75C	107.4
C90—C89—C88	113.3 (8)	C76B—C75B—H75D	107.4
C90—C89—H89A	108.9	C76B—C75B—C74B	120 (2)
C90—C89—H89B	108.9	C74B—C75B—C73	16.0 (11)
C89—C90—H90A	109.5	C74B—C75B—C74	33.0 (12)
C89—C90—H90B	109.5	C74B—C75B—H75C	107.4
C89—C90—H90C	109.5	C74B—C75B—H75D	107.4
H90A—C90—H90B	109.5	H78D—C78B—H78E	109.5
H90A—C90—H90C	109.5	H78D—C78B—H78F	109.5
H90B—C90—H90C	109.5	H78E—C78B—H78F	109.5
C36—C91—H91A	108.6	C77B—C78B—H78D	109.5
C36—C91—H91B	108.6	C77B—C78B—H78E	109.5
H91A—C91—H91B	107.6	C77B—C78B—H78F	109.5
C92—C91—C36	114.7 (7)	C75B—C76B—H76C	107.7
C92—C91—H91A	108.6	C75B—C76B—H76D	107.7
C92—C91—H91B	108.6	C75B—C76B—C77B	118 (2)
C91—C92—H92A	107.0	H76C—C76B—H76D	107.1
C91—C92—H92B	107.0	C77B—C76B—H76C	107.7
C91—C92—H92C	111.5	C77B—C76B—H76D	107.7
C91—C92—H92D	111.5	C78B—C77B—C76B	100.0 (17)
C91—C92—C93	121.4 (9)	C78B—C77B—H77C	111.8
C91—C92—C93B	101.2 (12)	C78B—C77B—H77D	111.8
H92A—C92—H92B	106.7	C76B—C77B—H77C	111.8
H92C—C92—H92D	109.3	C76B—C77B—H77D	111.8
C93—C92—H92A	107.0	H77C—C77B—H77D	109.5
C93—C92—H92B	107.0	C18—C73B—C79	63.1 (10)
C93B—C92—H92C	111.5	C18—C73B—H73C	114.9
C93B—C92—H92D	111.5	C18—C73B—H73D	115.8
C54—C97—H97A	108.2	C18—C73B—C74B	104.7 (19)
C54—C97—H97B	108.2	C18—C73B—C18B	16.4 (8)
H97A—C97—H97B	107.4	C73—C73B—C18	100 (2)
C98—C97—C54	116.2 (6)	C73—C73B—C79	161 (2)
C98—C97—H97A	108.2	C73—C73B—H73C	108.1
C98—C97—H97B	108.2	C73—C73B—H73D	111.1
C97—C98—H98A	109.3	C73—C73B—C74B	5.2 (16)
C97—C98—H98B	109.3	C73—C73B—C18B	116 (2)
C97—C98—C99	111.7 (7)	C79—C73B—H73C	87.5
H98A—C98—H98B	107.9	C79—C73B—H73D	72.7
C99—C98—H98A	109.3	H73C—C73B—H73D	106.8
C99—C98—H98B	109.3	C74B—C73B—C79	165 (2)
C98—C99—H99A	108.8	C74B—C73B—H73C	107.0
C98—C99—H99B	108.8	C74B—C73B—H73D	107.0
H99A—C99—H99B	107.7	C18B—C73B—C79	46.9 (9)
C100—C99—C98	114.0 (8)	C18B—C73B—H73C	107.0
C100—C99—H99A	108.8	C18B—C73B—H73D	107.0
C100—C99—H99B	108.8	C18B—C73B—C74B	121.2 (19)
C99—C100—H10C	108.4	C73—C74B—C74	124 (4)
C99—C100—H10D	108.4	C73—C74B—C75B	125 (4)
C99—C100—H10A	107.4	C73—C74B—C73B	7 (2)
C99—C100—H10B	107.4	C73—C74B—H74C	107.2
C99—C100—C101	119.7 (11)	C73—C74B—H74D	101.6
C99—C100—C201	116 (2)	C74—C74B—C75B	96 (2)
H10C—C100—H10D	107.5	C74—C74B—C73B	130 (3)
H10A—C100—H10B	106.9	C74—C74B—H74C	93.2
C101—C100—H10A	107.4	C74—C74B—H74D	22.8
C101—C100—H10B	107.4	C75B—C74B—H74C	107.6
C201—C100—H10C	108.4	C75B—C74B—H74D	107.6
C201—C100—H10D	108.4	C73B—C74B—C75B	119 (2)
C54—C103—H10E	108.4	C73B—C74B—H74C	107.6
C54—C103—H10F	108.4	C73B—C74B—H74D	107.6
H10E—C103—H10F	107.5	H74C—C74B—H74D	107.1
C104—C103—C54	115.5 (6)	C18—C79B—C81	165.1 (15)
C104—C103—H10E	108.4	C18—C79B—H79C	104.9
C104—C103—H10F	108.4	C18—C79B—H79D	104.1
C103—C104—H10G	106.7	C79—C79B—C18	48.3 (11)
C103—C104—H10H	106.4	C79—C79B—C81	117.0 (19)
C103—C104—C105	106.2 (10)	C79—C79B—H79C	76.5
C103—C104—C205	127 (2)	C79—C79B—H79D	76.5
H10G—C104—H10H	106.5	C79—C79B—C80B	171 (2)
C105—C104—H10G	107.7	C79—C79B—C18B	55.5 (13)
C105—C104—H10H	122.5	C80—C79B—C18	151 (3)
C205—C104—H10G	106.0	C80—C79B—C79	146 (4)
C205—C104—H10H	103.3	C80—C79B—C81	38.8 (18)
C72—C109—H10I	108.3	C80—C79B—H79C	103.5
C72—C109—H10J	108.3	C80—C79B—H79D	71.2
H10I—C109—H10J	107.4	C80—C79B—C80B	41 (2)
C110—C109—C72	115.9 (6)	C80—C79B—C18B	146 (3)
C110—C109—H10I	108.3	C81—C79B—H79C	69.6
C110—C109—H10J	108.3	C81—C79B—H79D	66.0
C109—C110—H11A	107.8	H79C—C79B—H79D	107.4
C109—C110—H11B	107.8	C80B—C79B—C18	122.8 (16)
C109—C110—H11C	111.3	C80B—C79B—C81	71.9 (14)
C109—C110—H11D	111.3	C80B—C79B—H79C	108.4
C109—C110—C111	118.0 (9)	C80B—C79B—H79D	108.4
C109—C110—C211	102.1 (11)	C80B—C79B—C18B	115.6 (18)
H11A—C110—H11B	107.1	C18B—C79B—C18	7.2 (7)
H11C—C110—H11D	109.2	C18B—C79B—C81	172.2 (18)
C111—C110—H11A	107.8	C18B—C79B—H79C	108.4
C111—C110—H11B	107.8	C18B—C79B—H79D	108.4
C211—C110—H11C	111.3	C80—C80B—C79B	24.1 (11)
C211—C110—H11D	111.3	C80—C80B—H80C	128.4
C72—C115—H11E	108.0	C80—C80B—H80D	105.3
C72—C115—H11F	108.0	C80—C80B—C81B	101 (3)
H11E—C115—H11F	107.2	C79B—C80B—H80C	106.8
C116—C115—C72	117.3 (6)	C79B—C80B—H80D	106.8
C116—C115—H11E	108.0	C79B—C80B—C81B	122 (2)
C116—C115—H11F	108.0	H80C—C80B—H80D	106.6
C115—C116—H11G	111.4	C81B—C80B—H80C	106.8
C115—C116—H11H	111.4	C81B—C80B—H80D	106.8
C115—C116—H11I	106.2	C82—C81B—C81	54.4 (12)
C115—C116—H11J	106.2	C82—C81B—H81C	108.1
C115—C116—C117	101.9 (8)	C82—C81B—H81D	108.1
C115—C116—C217	124.3 (11)	C81—C81B—H81C	87.9
H11G—C116—H11H	109.2	C81—C81B—H81D	160.6
H11I—C116—H11J	106.4	C83—C81B—C82	61.1 (14)
C117—C116—H11G	111.4	C83—C81B—C81	102 (2)
C117—C116—H11H	111.4	C83—C81B—C80B	177 (3)
C217—C116—H11I	106.2	C83—C81B—H81C	74.3
C217—C116—H11J	106.2	C83—C81B—H81D	71.9
C4—C18—C17	98.5 (6)	C80B—C81B—C82	117 (3)
C4—C18—C73	114.4 (7)	C80B—C81B—C81	77.2 (19)
C4—C18—C79B	94.0 (9)	C80B—C81B—H81C	108.1
C17—C18—C79B	94.2 (9)	C80B—C81B—H81D	108.1
C73—C18—C17	113.7 (8)	H81C—C81B—H81D	107.3
C73—C18—C79B	135.1 (10)	C18—C18B—C4	84 (2)
C79—C18—C4	112.2 (7)	C18—C18B—C79	113 (3)
C79—C18—C17	109.4 (7)	C18—C18B—C73B	40 (2)
C79—C18—C73	108.4 (7)	C79—C18B—C4	133.6 (13)
C79—C18—C79B	26.8 (7)	C79—C18B—C73B	74.1 (13)
C73B—C18—C4	125.1 (15)	C73B—C18B—C4	105.8 (15)
C73B—C18—C17	131.6 (15)	C82—C83B—H83C	109.8
C73B—C18—C73	33.5 (11)	C82—C83B—H83D	109.8
C73B—C18—C79	75.3 (13)	C83—C83B—C82	58.2 (9)
C73B—C18—C79B	101.8 (14)	C83—C83B—H83C	73.2
C18B—C18—C4	77 (2)	C83—C83B—H83D	80.7
C18B—C18—C17	82 (2)	C83—C83B—C84B	167 (3)
C18B—C18—C73	157 (2)	C84—C83B—C82	125 (4)
C18B—C18—C79	49 (2)	C84—C83B—C83	76 (3)
C18B—C18—C73B	124 (3)	C84—C83B—H83C	21.0
C18B—C18—C79B	22 (2)	C84—C83B—H83D	88.5
C18—C73—H73A	108.5	C84—C83B—C84B	112 (3)
C18—C73—H73B	108.5	H83C—C83B—H83D	108.3
C18—C73—C75B	173.6 (12)	C84B—C83B—C82	109 (2)
C18—C73—C18B	5.5 (6)	C84B—C83B—H83C	109.8
H73A—C73—H73B	107.5	C84B—C83B—H83D	109.8
C74—C73—C18	115.2 (9)	C83B—C84B—H84D	109.5
C74—C73—H73A	108.5	C83B—C84B—H84E	109.5
C74—C73—H73B	108.5	C83B—C84B—H84F	109.5
C74—C73—C75B	65.8 (10)	H84D—C84B—H84E	109.5
C74—C73—C18B	120.3 (9)	H84D—C84B—H84F	109.5
C75B—C73—H73A	76.6	H84E—C84B—H84F	109.5
C75B—C73—H73B	65.7	H1SA—C1S—H1SB	109.5
C75B—C73—C18B	169.7 (12)	H1SA—C1S—H1SC	109.5
C73B—C73—C18	46.7 (16)	H1SB—C1S—H1SC	109.5
C73B—C73—H73A	94.3	C2S—C1S—H1SA	109.5
C73B—C73—H73B	71.1	C2S—C1S—H1SB	109.5
C73B—C73—C74	156 (2)	C2S—C1S—H1SC	109.5
C73B—C73—C75B	130 (2)	C1S—C2S—H2SA	109.7
C73B—C73—C18B	41.2 (15)	C1S—C2S—H2SB	109.7
C74B—C73—C18	144 (3)	H2SA—C2S—H2SB	108.2
C74B—C73—H73A	78.0	C3S—C2S—C1S	110 (3)
C74B—C73—H73B	102.2	C3S—C2S—H2SA	109.7
C74B—C73—C74	35 (3)	C3S—C2S—H2SB	109.7
C74B—C73—C75B	39 (3)	C2S—C3S—H3SA	111.9
C74B—C73—C73B	168 (4)	C2S—C3S—H3SB	111.9
C74B—C73—C18B	150 (3)	C2S—C3S—C4S	99 (3)
C18B—C73—H73A	107.3	H3SA—C3S—H3SB	109.6
C18B—C73—H73B	104.1	C4S—C3S—H3SA	111.9
C73—C74—H74A	109.5	C4S—C3S—H3SB	111.9
C73—C74—H74B	109.5	C3S—C4S—H4SA	113.2
C73—C74—C75A	110.7 (9)	C3S—C4S—H4SB	113.2
C73—C74—C75B	68.2 (9)	H4SA—C4S—H4SB	110.6
H74A—C74—H74B	108.1	C5S—C4S—C3S	92 (3)
C75A—C74—H74A	109.5	C5S—C4S—H4SA	113.2
C75A—C74—H74B	109.5	C5S—C4S—H4SB	113.2
C75A—C74—C75B	84.4 (11)	C4S—C5S—H5SA	109.5
C75B—C74—H74A	61.2	C4S—C5S—H5SB	109.5
C75B—C74—H74B	165.3	C4S—C5S—H5SC	109.5
C74B—C74—C73	21.1 (14)	H5SA—C5S—H5SB	109.5
C74B—C74—H74A	88.9	H5SA—C5S—H5SC	109.5
C74B—C74—H74B	122.2	H5SB—C5S—H5SC	109.5
			
Ir1—N3—C43—C42	−4.3 (6)	C86—C87—C88—C89	177.3 (7)
Ir1—N3—C43—C44	169.7 (5)	C87—C88—C89—C90	−175.8 (8)
Ir1—N3—C47—C46	−170.6 (5)	C91—C36—C85—C86	−177.0 (7)
Ir1—N4—C61—C60	5.1 (8)	C91—C92—C93—C94	−59.9 (17)
Ir1—N4—C61—C62	−175.5 (6)	C91—C92—C93B—C94B	173 (2)
Ir1—N4—C65—C64	171.9 (6)	C92—C93—C94—C95	−159.0 (13)
Ir1—C37—C38—C39	−168.8 (4)	C92—C93B—C94B—C95B	−149 (3)
Ir1—C37—C42—C41	168.5 (5)	C97—C54—C103—C104	175.2 (6)
Ir1—C37—C42—C43	−9.1 (7)	C97—C98—C99—C100	−170.0 (8)
Ir1—C55—C56—C57	−173.3 (5)	C98—C99—C100—C101	−170.8 (9)
Ir1—C55—C60—C59	174.0 (6)	C98—C99—C100—C201	153 (2)
Ir1—C55—C60—C61	−6.6 (8)	C99—C100—C101—C102	−81.1 (17)
Ir2—N1—C7—C6	−1.5 (7)	C99—C100—C201—C202	36 (4)
Ir2—N1—C7—C8	177.1 (5)	C103—C54—C97—C98	−172.2 (6)
Ir2—N1—C11—C10	−178.4 (4)	C103—C104—C105—C106	−178 (2)
Ir2—N2—C25—C24	−5.5 (8)	C103—C104—C205—C206	147 (6)
Ir2—N2—C25—C26	173.7 (7)	C104—C105—C106—C107	−8 (6)
Ir2—N2—C29—C28	−173.1 (6)	C104—C205—C206—C207	−169 (6)
Ir2—C1—C2—C3	−175.3 (5)	C109—C72—C115—C116	175.2 (7)
Ir2—C1—C6—C5	175.1 (5)	C109—C110—C111—C112	−168.9 (15)
Ir2—C1—C6—C7	−1.9 (8)	C109—C110—C211—C212	169 (2)
Ir2—C19—C20—C21	−174.9 (5)	C110—C111—C112—C113	−167.5 (17)
Ir2—C19—C24—C23	177.3 (6)	C110—C211—C212—C213	167 (3)
Ir2—C19—C24—C25	−2.4 (8)	C115—C72—C109—C110	−177.0 (7)
F1—C10—C11—N1	−177.2 (5)	C115—C116—C117—C118	−179.8 (13)
F2—C28—C29—N2	−179.9 (7)	C115—C116—C217—C218	172 (2)
F3—C46—C47—N3	−178.3 (5)	C116—C117—C118—C119	−179.5 (14)
F4—C64—C65—N4	−174.4 (6)	C116—C217—C218—C219	−172 (3)
N1—C7—C8—C9	0.7 (11)	C18—C4—C5—C6	−170.9 (7)
N2—C25—C26—C27	−1.6 (14)	C18—C4—C18B—C79	−116 (3)
N3—C43—C44—C45	1.8 (10)	C18—C4—C18B—C73B	−33.6 (19)
N4—C61—C62—C63	2.8 (13)	C18—C73—C74—C75A	−98.3 (11)
C1—C2—C3—C4	−0.2 (9)	C18—C73—C74—C75B	−173.0 (13)
C1—C2—C3—C12	179.7 (6)	C18—C73—C74—C74B	153 (6)
C1—C6—C7—N1	2.2 (9)	C18—C73—C73B—C79	−24 (7)
C1—C6—C7—C8	−176.2 (7)	C18—C73—C73B—C74B	−161 (20)
C2—C1—C6—C5	−2.9 (10)	C18—C73—C73B—C18B	0.0 (9)
C2—C1—C6—C7	−180.0 (6)	C18—C73—C74B—C74	−44 (9)
C2—C3—C4—C5	−1.8 (10)	C18—C73—C74B—C75B	−172 (2)
C2—C3—C4—C18	171.7 (6)	C18—C73—C74B—C73B	156 (25)
C2—C3—C4—C18B	−168.1 (9)	C18—C79—C80—C81	174.4 (9)
C2—C3—C12—C13	3.5 (11)	C18—C79—C80—C79B	42 (7)
C2—C3—C12—C17	−178.5 (6)	C18—C79—C80—C80B	32 (3)
C3—C4—C5—C6	1.4 (10)	C18—C79—C73B—C73	27 (8)
C3—C4—C18—C17	10.8 (8)	C18—C79—C73B—C74B	41 (8)
C3—C4—C18—C73	−110.1 (8)	C18—C79—C73B—C18B	−3.4 (11)
C3—C4—C18—C79	125.9 (7)	C18—C79—C79B—C80	−141 (6)
C3—C4—C18—C73B	−146.9 (16)	C18—C79—C79B—C81	−177.1 (14)
C3—C4—C18—C79B	105.6 (9)	C18—C79—C79B—C18B	0.6 (9)
C3—C4—C18—C18B	91 (2)	C18—C79—C18B—C4	104 (3)
C3—C4—C18B—C18	−97 (2)	C18—C79—C18B—C73B	7 (2)
C3—C4—C18B—C79	146.8 (17)	C18—C73B—C74B—C73	−19 (21)
C3—C4—C18B—C73B	−130.3 (12)	C18—C73B—C74B—C74	−42 (5)
C3—C12—C13—C14	176.3 (6)	C18—C73B—C74B—C75B	−169 (2)
C3—C12—C17—C16	−178.4 (6)	C18—C73B—C18B—C4	59 (3)
C3—C12—C17—C18	6.0 (8)	C18—C73B—C18B—C79	−169 (3)
C3—C12—C17—C18B	−13.7 (10)	C18—C79B—C80B—C80	−148 (4)
C4—C3—C12—C13	−176.6 (6)	C18—C79B—C80B—C81B	179 (2)
C4—C3—C12—C17	1.4 (7)	C73—C18—C79—C80	172.7 (9)
C4—C5—C6—C1	1.0 (10)	C73—C18—C79—C73B	−5.0 (15)
C4—C5—C6—C7	177.8 (6)	C73—C18—C79—C79B	−177 (2)
C4—C18—C73—C74	42.8 (12)	C73—C18—C79—C18B	−175 (3)
C4—C18—C73—C73B	−117 (3)	C73—C18—C73B—C79	171 (3)
C4—C18—C73—C74B	69 (7)	C73—C18—C73B—C74B	1.7 (19)
C4—C18—C73—C18B	−117 (6)	C73—C18—C73B—C18B	−180 (3)
C4—C18—C79—C80	−59.9 (10)	C73—C18—C18B—C4	−124 (5)
C4—C18—C79—C73B	122.3 (16)	C73—C18—C18B—C79	11 (7)
C4—C18—C79—C79B	−50 (2)	C73—C18—C18B—C73B	0 (4)
C4—C18—C79—C18B	−48 (2)	C73—C74—C75A—C76	163.6 (11)
C4—C18—C73B—C73	82 (3)	C73—C74—C74B—C75B	139 (6)
C4—C18—C73B—C79	−107.0 (12)	C73—C74—C74B—C73B	3 (3)
C4—C18—C73B—C74B	83 (2)	C73—C75B—C76B—C77B	179 (2)
C4—C18—C73B—C18B	−98 (3)	C73—C75B—C74B—C74	−138 (7)
C4—C18—C18B—C79	135.1 (19)	C73—C75B—C74B—C73B	4 (3)
C4—C18—C18B—C73B	124 (2)	C73—C73B—C74B—C74	−22 (19)
C5—C4—C18—C17	−176.4 (7)	C73—C73B—C74B—C75B	−150 (22)
C5—C4—C18—C73	62.6 (11)	C73—C73B—C18B—C4	59 (3)
C5—C4—C18—C79	−61.4 (10)	C73—C73B—C18B—C18	0 (3)
C5—C4—C18—C73B	25.8 (18)	C73—C73B—C18B—C79	−169 (3)
C5—C4—C18—C79B	−81.6 (11)	C74—C73—C73B—C18	−48 (6)
C5—C4—C18—C18B	−97 (2)	C74—C73—C73B—C79	−73 (11)
C5—C4—C18B—C18	98 (2)	C74—C73—C73B—C74B	151 (24)
C5—C4—C18B—C79	−18 (3)	C74—C73—C73B—C18B	−48 (6)
C5—C4—C18B—C73B	64.9 (17)	C74—C73—C74B—C75B	−127 (8)
C5—C6—C7—N1	−174.8 (6)	C74—C73—C74B—C73B	−159 (17)
C5—C6—C7—C8	6.8 (11)	C74—C75A—C76—C77	177.6 (12)
C6—C1—C2—C3	2.5 (9)	C74—C75B—C76B—C77B	−166 (2)
C6—C7—C8—C9	179.1 (7)	C74—C75B—C74B—C73	138 (7)
C7—N1—C11—C10	−3.8 (9)	C74—C75B—C74B—C73B	142 (4)
C7—C8—C9—C10	−1.4 (11)	C75A—C74—C74B—C73	−80 (5)
C8—C9—C10—F1	179.8 (6)	C75A—C74—C74B—C75B	58 (2)
C8—C9—C10—C11	−0.5 (11)	C75A—C74—C74B—C73B	−77 (4)
C9—C10—C11—N1	3.1 (10)	C75A—C76—C77—C78	−173.9 (12)
C11—N1—C7—C6	−176.6 (6)	C79—C18—C73—C74	168.9 (9)
C11—N1—C7—C8	2.0 (9)	C79—C18—C73—C73B	9 (3)
C12—C3—C4—C5	178.3 (6)	C79—C18—C73—C74B	−165 (6)
C12—C3—C4—C18	−8.2 (8)	C79—C18—C73—C18B	9 (5)
C12—C3—C4—C18B	12.0 (10)	C79—C18—C73B—C73	−171 (3)
C12—C13—C14—C15	1.5 (10)	C79—C18—C73B—C74B	−170 (2)
C12—C17—C18—C4	−10.0 (8)	C79—C18—C73B—C18B	9 (3)
C12—C17—C18—C73	111.5 (8)	C79—C18—C18B—C4	−135.1 (19)
C12—C17—C18—C79	−127.2 (7)	C79—C18—C18B—C73B	−11 (3)
C12—C17—C18—C73B	145.5 (18)	C79—C80—C81—C82	155.5 (9)
C12—C17—C18—C79B	−104.6 (9)	C79—C80—C81—C79B	−18 (3)
C12—C17—C18—C18B	−86 (2)	C79—C80—C81—C81B	−162.7 (15)
C13—C12—C17—C16	−0.2 (10)	C79—C80—C79B—C18	−103 (11)
C13—C12—C17—C18	−175.8 (6)	C79—C80—C79B—C81	56 (8)
C13—C12—C17—C18B	164.5 (9)	C79—C80—C79B—C80B	−172 (6)
C13—C14—C15—C16	0.2 (11)	C79—C80—C79B—C18B	−114 (11)
C14—C15—C16—C17	−1.9 (11)	C79—C80—C80B—C79B	4 (3)
C15—C16—C17—C12	2.0 (10)	C79—C80—C80B—C81B	155.8 (18)
C15—C16—C17—C18	176.8 (7)	C79—C73B—C74B—C73	−56 (23)
C15—C16—C17—C18B	−159.7 (11)	C79—C73B—C74B—C74	−79 (10)
C16—C17—C18—C4	174.8 (6)	C79—C73B—C74B—C75B	154 (7)
C16—C17—C18—C73	−63.7 (10)	C79—C73B—C18B—C4	−131.6 (13)
C16—C17—C18—C79	57.6 (10)	C79—C73B—C18B—C18	169 (3)
C16—C17—C18—C73B	−30 (2)	C80—C79—C73B—C18	−5 (4)
C16—C17—C18—C79B	80.2 (10)	C80—C79—C73B—C73	22 (11)
C16—C17—C18—C18B	99 (2)	C80—C79—C73B—C74B	35 (10)
C17—C12—C13—C14	−1.5 (9)	C80—C79—C73B—C18B	−9 (3)
C17—C18—C73—C74	−69.3 (11)	C80—C79—C79B—C18	141 (6)
C17—C18—C73—C73B	131 (3)	C80—C79—C79B—C81	−36 (5)
C17—C18—C73—C74B	−43 (7)	C80—C79—C79B—C18B	142 (7)
C17—C18—C73—C18B	131 (6)	C80—C79—C18B—C4	−86 (2)
C17—C18—C79—C80	48.3 (10)	C80—C79—C18B—C18	169 (2)
C17—C18—C79—C73B	−129.4 (16)	C80—C79—C18B—C73B	176.6 (13)
C17—C18—C79—C79B	58 (2)	C80—C81—C81B—C82	−130.0 (14)
C17—C18—C79—C18B	60 (2)	C80—C81—C81B—C83	−171 (2)
C17—C18—C73B—C73	−68 (3)	C80—C81—C81B—C80B	6.7 (17)
C17—C18—C73B—C79	103.1 (14)	C80—C79B—C80B—C81B	−33 (4)
C17—C18—C73B—C74B	−67 (3)	C80—C80B—C81B—C82	29 (4)
C17—C18—C73B—C18B	112 (3)	C80—C80B—C81B—C81	−9 (2)
C17—C18—C18B—C4	100.6 (7)	C81—C82—C83—C84	173.9 (12)
C17—C18—C18B—C79	−124.3 (18)	C81—C82—C83—C81B	−46 (2)
C17—C18—C18B—C73B	−135 (2)	C81—C82—C83—C83B	155 (3)
C19—C20—C21—C22	−0.6 (11)	C81—C82—C81B—C83	133 (2)
C19—C20—C21—C30	−179.3 (7)	C81—C82—C81B—C80B	−48 (2)
C19—C24—C25—N2	5.2 (10)	C81—C82—C83B—C83	−64 (5)
C19—C24—C25—C26	−173.9 (8)	C81—C82—C83B—C84	−102 (8)
C20—C19—C24—C23	3.9 (11)	C81—C82—C83B—C84B	121 (4)
C20—C19—C24—C25	−175.8 (6)	C81—C80—C79B—C18	−160 (4)
C20—C21—C22—C23	2.6 (12)	C81—C80—C79B—C79	−56 (8)
C20—C21—C22—C36	−179.1 (7)	C81—C80—C79B—C80B	132 (4)
C20—C21—C30—C31	−2.5 (13)	C81—C80—C79B—C18B	−170 (4)
C20—C21—C30—C35	176.7 (8)	C81—C80—C80B—C79B	−141 (4)
C21—C22—C23—C24	−1.3 (12)	C81—C80—C80B—C81B	11 (3)
C21—C22—C36—C35	2.1 (8)	C81—C79B—C80B—C80	29 (3)
C21—C22—C36—C85	−116.0 (7)	C81—C79B—C80B—C81B	−4 (3)
C21—C22—C36—C91	122.1 (8)	C83—C82—C81—C80	86.9 (14)
C21—C30—C31—C32	176.9 (7)	C83—C82—C81—C79B	89.3 (17)
C21—C30—C35—C34	−175.7 (7)	C83—C82—C81—C81B	34.5 (16)
C21—C30—C35—C36	3.6 (9)	C83—C82—C81B—C81	−133 (2)
C22—C21—C30—C31	178.7 (7)	C83—C82—C81B—C80B	178 (4)
C22—C21—C30—C35	−2.1 (9)	C83—C82—C83B—C84	−38 (5)
C22—C23—C24—C19	−2.0 (12)	C83—C82—C83B—C84B	−175 (5)
C22—C23—C24—C25	177.7 (7)	C83—C84—C83B—C82	33 (4)
C22—C36—C85—C86	57.4 (9)	C83—C84—C83B—C84B	168 (5)
C22—C36—C91—C92	−55.7 (10)	C84—C83—C81B—C82	75 (3)
C23—C22—C36—C35	−179.9 (8)	C84—C83—C81B—C81	112 (3)
C23—C22—C36—C85	62.0 (11)	C84—C83—C83B—C82	−149 (5)
C23—C22—C36—C91	−59.9 (11)	C84—C83—C83B—C84B	−127 (24)
C23—C24—C25—N2	−174.5 (7)	C93—C94—C95—C96	177.5 (14)
C23—C24—C25—C26	6.4 (14)	C105—C106—C107—C108	172 (3)
C24—C19—C20—C21	−2.6 (10)	C111—C112—C113—C114	80 (3)
C24—C25—C26—C27	177.4 (9)	C117—C118—C119—C120	176.3 (16)
C25—N2—C29—C28	−2.0 (11)	C211—C212—C213—C214	−173 (3)
C25—C26—C27—C28	1.5 (16)	C93B—C94B—C95B—C96B	−68 (5)
C26—C27—C28—F2	−179.8 (9)	C217—C218—C219—C220	−55 (4)
C26—C27—C28—C29	−1.6 (15)	C205—C206—C207—C208	−176 (7)
C27—C28—C29—N2	1.9 (13)	C75B—C73—C74—C75A	74.7 (13)
C29—N2—C25—C24	−177.3 (6)	C75B—C73—C74—C74B	−34 (5)
C29—N2—C25—C26	1.9 (11)	C75B—C73—C73B—C18	−172.5 (15)
C30—C21—C22—C23	−178.4 (7)	C75B—C73—C73B—C79	163 (7)
C30—C21—C22—C36	−0.2 (9)	C75B—C73—C73B—C74B	26 (20)
C30—C31—C32—C33	−1.5 (11)	C75B—C73—C73B—C18B	−172.5 (15)
C30—C35—C36—C22	−3.4 (8)	C75B—C73—C74B—C74	127 (8)
C30—C35—C36—C85	114.4 (7)	C75B—C73—C74B—C73B	−32 (24)
C30—C35—C36—C91	−124.7 (7)	C75B—C74—C75A—C76	−132.3 (13)
C31—C30—C35—C34	3.7 (11)	C75B—C74—C74B—C73	−139 (6)
C31—C30—C35—C36	−177.1 (7)	C75B—C74—C74B—C73B	−136 (5)
C31—C32—C33—C34	4.0 (12)	C75B—C76B—C77B—C78B	146 (3)
C32—C33—C34—C35	−2.5 (11)	C76B—C75B—C74B—C73	172 (5)
C33—C34—C35—C30	−1.3 (11)	C76B—C75B—C74B—C74	34 (4)
C33—C34—C35—C36	179.6 (7)	C76B—C75B—C74B—C73B	176 (3)
C34—C35—C36—C22	175.7 (8)	C73B—C18—C73—C74	160 (3)
C34—C35—C36—C85	−66.5 (10)	C73B—C18—C73—C74B	−173 (7)
C34—C35—C36—C91	54.4 (11)	C73B—C18—C73—C18B	0 (6)
C35—C30—C31—C32	−2.3 (11)	C73B—C18—C79—C80	177.7 (16)
C35—C36—C85—C86	−53.4 (9)	C73B—C18—C79—C79B	−172 (2)
C35—C36—C91—C92	57.2 (9)	C73B—C18—C79—C18B	−170 (3)
C36—C22—C23—C24	−179.2 (8)	C73B—C18—C18B—C4	−124 (2)
C36—C85—C86—C87	−178.8 (6)	C73B—C18—C18B—C79	11 (3)
C36—C91—C92—C93	−165.7 (9)	C73B—C73—C74—C75A	−61 (5)
C36—C91—C92—C93B	176.1 (15)	C73B—C73—C74—C75B	−136 (5)
C37—C38—C39—C40	0.4 (9)	C73B—C73—C74—C74B	−170 (8)
C37—C38—C39—C48	178.2 (6)	C73B—C73—C74B—C74	159 (17)
C37—C42—C43—N3	8.8 (8)	C73B—C73—C74B—C75B	32 (24)
C37—C42—C43—C44	−164.9 (6)	C73B—C79—C80—C81	178 (3)
C38—C37—C42—C41	−3.8 (9)	C73B—C79—C80—C79B	46 (8)
C38—C37—C42—C43	178.5 (5)	C73B—C79—C80—C80B	36 (4)
C38—C39—C40—C41	−1.6 (9)	C73B—C79—C79B—C18	−9 (3)
C38—C39—C40—C54	177.9 (5)	C73B—C79—C79B—C80	−150 (5)
C38—C39—C48—C49	1.3 (11)	C73B—C79—C79B—C81	174 (2)
C38—C39—C48—C53	−178.0 (6)	C73B—C79—C79B—C18B	−8 (3)
C39—C40—C41—C42	0.0 (9)	C73B—C79—C18B—C4	97 (2)
C39—C40—C54—C53	0.6 (7)	C73B—C79—C18B—C18	−7 (2)
C39—C40—C54—C97	−117.0 (6)	C74B—C73—C74—C75A	108 (5)
C39—C40—C54—C103	118.9 (6)	C74B—C73—C74—C75B	34 (5)
C39—C48—C49—C50	−179.2 (6)	C74B—C73—C73B—C18	161 (20)
C39—C48—C53—C52	179.4 (5)	C74B—C73—C73B—C79	137 (19)
C39—C48—C53—C54	0.3 (7)	C74B—C73—C73B—C18B	161 (20)
C40—C39—C48—C49	179.3 (6)	C74B—C74—C75A—C76	−174.1 (19)
C40—C39—C48—C53	0.1 (7)	C74B—C75B—C76B—C77B	176 (3)
C40—C41—C42—C37	2.7 (9)	C74B—C73B—C18B—C4	61 (3)
C40—C41—C42—C43	−179.8 (6)	C74B—C73B—C18B—C18	2 (3)
C40—C54—C97—C98	61.8 (8)	C74B—C73B—C18B—C79	−167 (3)
C40—C54—C103—C104	−59.3 (8)	C79B—C18—C73—C74	167.2 (13)
C41—C40—C54—C53	180.0 (6)	C79B—C18—C73—C73B	7 (3)
C41—C40—C54—C97	62.4 (9)	C79B—C18—C73—C74B	−166 (6)
C41—C40—C54—C103	−61.7 (9)	C79B—C18—C73—C18B	7 (5)
C41—C42—C43—N3	−168.8 (5)	C79B—C18—C79—C80	−10 (2)
C41—C42—C43—C44	17.5 (10)	C79B—C18—C79—C73B	172 (2)
C42—C37—C38—C39	2.2 (9)	C79B—C18—C79—C18B	2 (3)
C42—C43—C44—C45	175.1 (6)	C79B—C18—C73B—C73	−175 (2)
C43—N3—C47—C46	1.1 (9)	C79B—C18—C73B—C79	−3.5 (11)
C43—C44—C45—C46	0.5 (10)	C79B—C18—C73B—C74B	−173 (2)
C44—C45—C46—F3	177.5 (6)	C79B—C18—C73B—C18B	5 (3)
C44—C45—C46—C47	−2.0 (10)	C79B—C18—C18B—C4	−137 (5)
C45—C46—C47—N3	1.2 (10)	C79B—C18—C18B—C79	−2 (4)
C47—N3—C43—C42	−176.7 (5)	C79B—C18—C18B—C73B	−14 (7)
C47—N3—C43—C44	−2.6 (9)	C79B—C79—C80—C81	132 (7)
C48—C39—C40—C41	−179.9 (5)	C79B—C79—C80—C80B	−11 (7)
C48—C39—C40—C54	−0.4 (7)	C79B—C79—C73B—C18	13 (4)
C48—C49—C50—C51	0.6 (9)	C79B—C79—C73B—C73	40 (10)
C48—C53—C54—C40	−0.6 (7)	C79B—C79—C73B—C74B	54 (10)
C48—C53—C54—C97	118.3 (6)	C79B—C79—C73B—C18B	10 (4)
C48—C53—C54—C103	−120.5 (6)	C79B—C79—C18B—C4	−78 (3)
C49—C48—C53—C52	0.0 (9)	C79B—C79—C18B—C18	178 (3)
C49—C48—C53—C54	−179.0 (5)	C79B—C79—C18B—C73B	−174 (2)
C49—C50—C51—C52	−1.1 (10)	C79B—C80—C81—C82	173 (3)
C50—C51—C52—C53	1.1 (10)	C79B—C80—C81—C81B	−145 (4)
C51—C52—C53—C48	−0.5 (9)	C79B—C80—C80B—C81B	152 (3)
C51—C52—C53—C54	178.3 (6)	C79B—C81—C81B—C82	−139.2 (14)
C52—C53—C54—C40	−179.5 (6)	C79B—C81—C81B—C83	−180 (2)
C52—C53—C54—C97	−60.6 (9)	C79B—C81—C81B—C80B	−2.5 (19)
C52—C53—C54—C103	60.6 (9)	C79B—C80B—C81B—C82	42 (4)
C53—C48—C49—C50	0.0 (9)	C79B—C80B—C81B—C81	4 (3)
C53—C54—C97—C98	−50.3 (8)	C80B—C80—C81—C82	−51 (2)
C53—C54—C103—C104	53.5 (8)	C80B—C80—C81—C79B	136 (4)
C54—C40—C41—C42	−179.3 (6)	C80B—C80—C81—C81B	−9 (2)
C54—C97—C98—C99	−177.4 (7)	C80B—C80—C79B—C18	68 (7)
C54—C103—C104—C105	175.9 (18)	C80B—C80—C79B—C79	172 (6)
C54—C103—C104—C205	−173 (4)	C80B—C80—C79B—C81	−132 (4)
C55—C56—C57—C58	−0.9 (11)	C80B—C80—C79B—C18B	58 (7)
C55—C56—C57—C66	178.4 (7)	C81B—C82—C81—C80	52.4 (15)
C55—C60—C61—N4	1.0 (10)	C81B—C82—C81—C79B	54.8 (18)
C55—C60—C61—C62	−178.3 (8)	C81B—C82—C83—C84	−140 (3)
C56—C55—C60—C59	−2.4 (11)	C81B—C82—C83—C83B	−159 (4)
C56—C55—C60—C61	177.0 (6)	C81B—C82—C83B—C83	16 (3)
C56—C57—C58—C59	−1.0 (12)	C81B—C82—C83B—C84	−22 (7)
C56—C57—C58—C72	177.5 (6)	C81B—C82—C83B—C84B	−159 (3)
C56—C57—C66—C67	1.3 (13)	C81B—C83—C84—C83B	−93 (5)
C56—C57—C66—C71	−178.7 (7)	C81B—C83—C83B—C82	−25 (4)
C57—C58—C59—C60	1.2 (12)	C81B—C83—C83B—C84	124 (4)
C57—C58—C72—C71	2.4 (8)	C81B—C83—C83B—C84B	−3 (23)
C57—C58—C72—C109	−118.0 (7)	C18B—C4—C5—C6	165.0 (11)
C57—C58—C72—C115	122.1 (7)	C18B—C4—C18—C17	−80 (2)
C57—C66—C67—C68	179.5 (7)	C18B—C4—C18—C73	159 (2)
C57—C66—C71—C70	179.1 (6)	C18B—C4—C18—C79	35 (2)
C57—C66—C71—C72	1.0 (8)	C18B—C4—C18—C73B	122 (3)
C58—C57—C66—C67	−179.3 (7)	C18B—C4—C18—C79B	15 (2)
C58—C57—C66—C71	0.6 (8)	C18B—C17—C18—C4	76 (2)
C58—C59—C60—C55	0.5 (12)	C18B—C17—C18—C73	−163 (2)
C58—C59—C60—C61	−178.9 (7)	C18B—C17—C18—C79	−42 (2)
C58—C72—C109—C110	61.2 (9)	C18B—C17—C18—C73B	−129 (3)
C58—C72—C115—C116	−62.4 (9)	C18B—C17—C18—C79B	−19 (2)
C59—C58—C72—C71	−179.3 (8)	C18B—C18—C73—C74	160 (5)
C59—C58—C72—C109	60.4 (11)	C18B—C18—C73—C73B	0 (6)
C59—C58—C72—C115	−59.6 (11)	C18B—C18—C73—C74B	−174 (8)
C59—C60—C61—N4	−179.6 (7)	C18B—C18—C79—C80	−12 (3)
C59—C60—C61—C62	1.1 (13)	C18B—C18—C79—C73B	170 (3)
C60—C55—C56—C57	2.6 (10)	C18B—C18—C79—C79B	−2 (3)
C60—C61—C62—C63	−177.9 (8)	C18B—C18—C73B—C73	180 (3)
C61—N4—C65—C64	−0.7 (10)	C18B—C18—C73B—C79	−9 (3)
C61—C62—C63—C64	−0.4 (14)	C18B—C18—C73B—C74B	−178 (3)
C62—C63—C64—F4	174.9 (8)	C18B—C73—C74—C75A	−96.1 (12)
C62—C63—C64—C65	−2.7 (14)	C18B—C73—C74—C75B	−170.8 (13)
C63—C64—C65—N4	3.3 (13)	C18B—C73—C74—C74B	155 (6)
C65—N4—C61—C60	178.4 (6)	C18B—C73—C73B—C18	0.0 (9)
C65—N4—C61—C62	−2.2 (11)	C18B—C73—C73B—C79	−24 (6)
C66—C57—C58—C59	179.6 (7)	C18B—C73—C73B—C74B	−161 (20)
C66—C57—C58—C72	−1.9 (9)	C18B—C73—C74B—C74	−46 (10)
C66—C67—C68—C69	1.0 (10)	C18B—C73—C74B—C75B	−173 (3)
C66—C71—C72—C58	−2.0 (8)	C18B—C73—C74B—C73B	155 (26)
C66—C71—C72—C109	117.6 (7)	C18B—C79—C80—C81	170.8 (11)
C66—C71—C72—C115	−120.5 (6)	C18B—C79—C80—C79B	39 (7)
C67—C66—C71—C70	−1.0 (11)	C18B—C79—C80—C80B	28 (3)
C67—C66—C71—C72	−179.1 (6)	C18B—C79—C73B—C18	3.4 (11)
C67—C68—C69—C70	−0.2 (11)	C18B—C79—C73B—C73	31 (8)
C68—C69—C70—C71	−1.2 (11)	C18B—C79—C73B—C74B	44 (8)
C69—C70—C71—C66	1.8 (11)	C18B—C79—C79B—C18	−0.6 (9)
C69—C70—C71—C72	179.5 (7)	C18B—C79—C79B—C80	−142 (7)
C70—C71—C72—C58	−179.9 (7)	C18B—C79—C79B—C81	−177.7 (19)
C70—C71—C72—C109	−60.3 (10)	C18B—C73B—C74B—C73	−20 (21)
C70—C71—C72—C115	61.6 (10)	C18B—C73B—C74B—C74	−42 (5)
C71—C66—C67—C68	−0.4 (10)	C18B—C73B—C74B—C75B	−170 (2)
C71—C72—C109—C110	−52.4 (9)	C18B—C79B—C80B—C80	−148 (4)
C71—C72—C115—C116	50.5 (9)	C18B—C79B—C80B—C81B	179 (3)
C72—C58—C59—C60	−177.0 (8)	C83B—C82—C81—C80	141 (4)
C72—C109—C110—C111	−179.9 (13)	C83B—C82—C81—C79B	144 (4)
C72—C109—C110—C211	−169 (2)	C83B—C82—C81—C81B	89 (5)
C72—C115—C116—C117	171.1 (11)	C83B—C82—C83—C84	19 (3)
C72—C115—C116—C217	178 (2)	C83B—C82—C83—C81B	159 (4)
C82—C81—C81B—C83	−40.6 (17)	C83B—C82—C81B—C81	−152 (3)
C82—C81—C81B—C80B	137 (2)	C83B—C82—C81B—C83	−19 (3)
C82—C83—C84—C83B	−27 (4)	C83B—C82—C81B—C80B	159 (3)
C82—C83—C81B—C81	37.3 (14)	C83B—C83—C81B—C82	23 (4)
C82—C83—C83B—C84	149 (5)	C83B—C83—C81B—C81	61 (4)
C82—C83—C83B—C84B	22 (20)	C1S—C2S—C3S—C4S	166 (3)
C85—C36—C91—C92	−179.6 (7)	C2S—C3S—C4S—C5S	164 (4)
C85—C86—C87—C88	−173.5 (7)		

### Di-µ2-cyanato-bis{bis[9,9-dihexyl-2-(5-methoxypyridin-2-yl)-9H-fluoren-1-yl]iridium} pentane monosolvate (III) Crystal data

**Table:**

[Ir_2_(C_31_H_38_NO)_4_(NCO)_2_]·C_5_H_12_	*F*(000) = 9552
*M**_r_* = 2303.07	*D*_x_ = 1.335 Mg m^−^^3^
Monoclinic, *P*2_1_/*c*	Mo *K*α radiation, λ = 0.71073 Å
*a* = 38.976 (6) Å	Cell parameters from 5377 reflections
*b* = 21.615 (3) Å	θ = 2.2–25.0°
*c* = 28.759 (4) Å	µ = 2.38 mm^−^^1^
β = 108.920 (3)°	*T* = 120 K
*V* = 22920 (6) Å^3^	Plate, yellow
*Z* = 8	0.2 × 0.1 × 0.01 mm

### Di-µ2-cyanato-bis{bis[9,9-dihexyl-2-(5-methoxypyridin-2-yl)-9H-fluoren-1-yl]iridium} pentane monosolvate (III) Data collection

**Table:**

Bruker SMART CCD 6000 area detector diffractometer	40676 independent reflections
Radiation source: sealed X-ray tube	19348 reflections with *I* > 2σ(*I*)
Graphite monochromator	*R*_int_ = 0.143
Detector resolution: 5.6 pixels mm^-1^	θ_max_ = 25.3°, θ_min_ = 1.2°
ω scans	*h* = −42→46
Absorption correction: multi-scan (SADABS; Krause *et al.*, 2015)	*k* = −23→25
*T*_min_ = 0.809, *T*_max_ = 1.000	*l* = −34→26
95765 measured reflections	

### Di-µ2-cyanato-bis{bis[9,9-dihexyl-2-(5-methoxypyridin-2-yl)-9H-fluoren-1-yl]iridium} pentane monosolvate (III) Refinement

**Table:**

Refinement on *F*^2^	Primary atom site location: dual
Least-squares matrix: full	Secondary atom site location: difference Fourier map
*R*[*F*^2^ > 2σ(*F*^2^)] = 0.077	Hydrogen site location: inferred from neighbouring sites
*wR*(*F*^2^) = 0.185	H-atom parameters constrained
*S* = 0.97	*w* = 1/[σ^2^(*F*_o_^2^) + (0.064*P*)^2^] where *P* = (*F*_o_^2^ + 2*F*_c_^2^)/3
40676 reflections	(Δ/σ)_max_ = 0.002
2479 parameters	Δρ_max_ = 2.80 e Å^−^^3^
2600 restraints	Δρ_min_ = −2.20 e Å^−^^3^

### Di-µ2-cyanato-bis{bis[9,9-dihexyl-2-(5-methoxypyridin-2-yl)-9H-fluoren-1-yl]iridium} pentane monosolvate (III) Special details

**Table:**

Experimental. The data collection nominally covered a full sphere of reciprocal space by a combination of 2 sets of 600 and 344 ω scans, each set at different φ angles and each scan (60 s exposure) covering -0.3° in ω. The crystal to detector distance was 5.84 cm.
Geometry. All esds (except the esd in the dihedral angle between two l.s. planes) are estimated using the full covariance matrix. The cell esds are taken into account individually in the estimation of esds in distances, angles and torsion angles; correlations between esds in cell parameters are only used when they are defined by crystal symmetry. An approximate (isotropic) treatment of cell esds is used for estimating esds involving l.s. planes.
Refinement. Extensive disorder of n-hexyl chains. Disordered solvent (presumably pentane) could not be modelled at atomic resolution and was masked.

### Di-µ2-cyanato-bis{bis[9,9-dihexyl-2-(5-methoxypyridin-2-yl)-9H-fluoren-1-yl]iridium} pentane monosolvate (III) Fractional atomic coordinates and isotropic or equivalent isotropic displacement parameters (Å^2^)

**Table:**

	*x*	*y*	*z*	*U*_iso_*/*U*_eq_	Occ. (<1)
Ir1	0.38516 (2)	0.33601 (2)	0.61355 (2)	0.02057 (11)	
Ir2	0.42702 (2)	0.33949 (2)	0.73765 (2)	0.02300 (12)	
O1	0.4959 (3)	0.3018 (5)	0.6592 (4)	0.070 (3)	
O2	0.3151 (3)	0.3372 (8)	0.6939 (4)	0.141 (7)	
O3	0.4252 (2)	0.0935 (4)	0.6313 (3)	0.040 (2)	
O4	0.3555 (2)	0.5731 (4)	0.6482 (3)	0.042 (2)	
O5	0.3651 (2)	0.1113 (4)	0.7197 (3)	0.040 (2)	
O6	0.4769 (2)	0.5511 (4)	0.6824 (3)	0.044 (2)	
N1	0.3782 (2)	0.2425 (4)	0.6066 (3)	0.021 (2)	
N01	0.4409 (2)	0.3274 (4)	0.6679 (3)	0.02057 (11)	
N02	0.3720 (2)	0.3438 (4)	0.6830 (3)	0.02057 (11)	
N2	0.3876 (2)	0.4302 (4)	0.6089 (3)	0.021 (2)	
N3	0.4246 (2)	0.2455 (4)	0.7482 (3)	0.020 (2)*	
N4	0.4344 (2)	0.4315 (4)	0.7348 (3)	0.027 (2)	
C01	0.4667 (3)	0.3152 (6)	0.6639 (4)	0.035 (3)	
C1	0.3339 (3)	0.3342 (5)	0.5705 (3)	0.012 (2)*	
C02	0.3442 (3)	0.3383 (7)	0.6887 (4)	0.050 (4)	
C2	0.3103 (3)	0.3846 (6)	0.5509 (4)	0.026 (3)	
H2A	0.319531	0.425605	0.555252	0.032*	
C3	0.2743 (3)	0.3747 (5)	0.5259 (4)	0.023 (2)	
C4	0.2600 (3)	0.3155 (5)	0.5171 (4)	0.025 (2)	
C5	0.2817 (3)	0.2643 (5)	0.5347 (4)	0.030 (3)	
H5A	0.272013	0.223609	0.529200	0.036*	
C6	0.3187 (3)	0.2745 (5)	0.5609 (4)	0.023 (2)	
C7	0.3442 (3)	0.2234 (5)	0.5784 (4)	0.027 (2)	
C8	0.3381 (3)	0.1608 (5)	0.5688 (4)	0.029 (3)	
H8	0.314557	0.147633	0.549232	0.035*	
C9	0.3640 (3)	0.1181 (6)	0.5859 (4)	0.034 (3)	
H9	0.358851	0.075481	0.579018	0.041*	
C10	0.3988 (3)	0.1374 (5)	0.6142 (4)	0.030 (3)	
C11	0.4048 (3)	0.1995 (5)	0.6239 (4)	0.025 (2)	
H11	0.428345	0.212814	0.643230	0.030*	
C12	0.2450 (3)	0.4199 (6)	0.5032 (4)	0.028 (3)	
C13	0.2458 (3)	0.4825 (6)	0.5018 (4)	0.031 (3)	
H13	0.267775	0.503955	0.517626	0.038*	
C14	0.2148 (3)	0.5150 (6)	0.4773 (4)	0.038 (3)	
H14	0.215184	0.558913	0.476601	0.046*	
C15	0.1827 (3)	0.4825 (7)	0.4533 (4)	0.043 (3)	
H15	0.161443	0.504231	0.434982	0.052*	
C16	0.1822 (3)	0.4197 (6)	0.4567 (4)	0.037 (3)	
H16	0.160202	0.398011	0.441766	0.044*	
C17	0.2132 (3)	0.3871 (6)	0.4812 (4)	0.029 (2)	
C18	0.2193 (3)	0.3170 (6)	0.4879 (4)	0.033 (3)	
C19	0.4062 (3)	0.3423 (5)	0.5594 (4)	0.022 (2)	
C20	0.4160 (3)	0.2960 (5)	0.5326 (4)	0.023 (3)	
H6	0.409484	0.254396	0.536381	0.027*	
C21	0.4349 (3)	0.3087 (5)	0.5007 (4)	0.024 (2)	
C22	0.4436 (3)	0.3688 (5)	0.4922 (4)	0.025 (3)	
C23	0.4341 (3)	0.4173 (5)	0.5167 (4)	0.021 (2)	
H23	0.439600	0.458763	0.510747	0.025*	
C24	0.4161 (3)	0.4038 (5)	0.5507 (4)	0.022 (2)	
C25	0.4033 (3)	0.4528 (5)	0.5764 (4)	0.029 (3)	
C26	0.4040 (3)	0.5161 (5)	0.5699 (4)	0.030 (3)	
H26	0.415868	0.532235	0.548451	0.036*	
C27	0.3879 (3)	0.5563 (5)	0.5937 (4)	0.032 (3)	
H27	0.388224	0.599631	0.588221	0.038*	
C28	0.3712 (3)	0.5325 (6)	0.6258 (4)	0.032 (3)	
C29	0.3724 (3)	0.4701 (5)	0.6338 (4)	0.026 (3)	
H29	0.362348	0.453780	0.657298	0.031*	
C30	0.4487 (3)	0.2650 (5)	0.4701 (4)	0.027 (3)	
C31	0.4461 (3)	0.2019 (5)	0.4650 (4)	0.030 (3)	
H31	0.434090	0.177821	0.482657	0.036*	
C32	0.4618 (3)	0.1746 (5)	0.4332 (4)	0.033 (3)	
H32	0.460880	0.130949	0.429288	0.039*	
C33	0.4786 (3)	0.2094 (6)	0.4073 (4)	0.031 (3)	
H33	0.488986	0.189617	0.385555	0.037*	
C34	0.4808 (3)	0.2727 (6)	0.4122 (4)	0.030 (3)	
H34	0.492352	0.296213	0.393675	0.036*	
C35	0.4659 (3)	0.3024 (5)	0.4444 (4)	0.024 (2)	
C36	0.4645 (3)	0.3702 (5)	0.4548 (4)	0.028 (2)	
C37	0.4775 (3)	0.3258 (5)	0.7796 (4)	0.023 (2)	
C38	0.5062 (3)	0.3675 (5)	0.7951 (4)	0.024 (3)	
H38	0.501422	0.410380	0.789104	0.029*	
C39	0.5422 (3)	0.3485 (5)	0.8195 (4)	0.027 (3)	
C40	0.5496 (3)	0.2858 (6)	0.8324 (4)	0.032 (3)	
C41	0.5223 (3)	0.2417 (5)	0.8182 (4)	0.026 (3)	
H41	0.527385	0.199251	0.825901	0.031*	
C42	0.4867 (3)	0.2619 (5)	0.7919 (4)	0.024 (2)	
C43	0.4565 (3)	0.2191 (5)	0.7741 (4)	0.025 (3)	
C44	0.4576 (3)	0.1557 (5)	0.7816 (4)	0.032 (3)	
H44	0.479939	0.136736	0.799669	0.039*	
C45	0.4273 (3)	0.1200 (6)	0.7635 (4)	0.035 (3)	
H45	0.428322	0.076415	0.768276	0.042*	
C46	0.3947 (3)	0.1492 (6)	0.7380 (4)	0.035 (3)	
C47	0.3936 (3)	0.2125 (5)	0.7302 (4)	0.028 (3)	
H47	0.371445	0.232429	0.712609	0.033*	
C48	0.5759 (3)	0.3822 (5)	0.8353 (4)	0.031 (3)	
C49	0.5827 (3)	0.4438 (6)	0.8293 (4)	0.034 (3)	
H49	0.563422	0.471465	0.814045	0.041*	
C50	0.6191 (3)	0.4650 (6)	0.8465 (5)	0.055 (4)	
H50	0.624485	0.507184	0.842608	0.066*	
C51	0.6465 (4)	0.4239 (6)	0.8689 (6)	0.060 (4)	
H51	0.670914	0.437927	0.880239	0.072*	
C52	0.6391 (4)	0.3629 (7)	0.8749 (6)	0.063 (5)	
H52	0.658272	0.335363	0.890861	0.076*	
C53	0.6042 (3)	0.3418 (6)	0.8581 (4)	0.036 (3)	
C54	0.5897 (3)	0.2771 (6)	0.8609 (4)	0.040 (3)	
C55	0.4075 (3)	0.3666 (5)	0.7903 (4)	0.022 (2)	
C56	0.3929 (3)	0.3280 (5)	0.8201 (4)	0.026 (3)	
H56	0.395740	0.284313	0.820199	0.032*	
C57	0.3743 (3)	0.3558 (5)	0.8488 (4)	0.026 (2)	
C58	0.3706 (3)	0.4198 (5)	0.8511 (4)	0.028 (3)	
C59	0.3866 (3)	0.4582 (5)	0.8253 (4)	0.028 (3)	
H59	0.385557	0.501956	0.828033	0.034*	
C60	0.4043 (3)	0.4309 (5)	0.7948 (4)	0.021 (2)	
C61	0.4215 (3)	0.4666 (5)	0.7648 (4)	0.021 (3)*	
C62	0.4264 (3)	0.5304 (5)	0.7668 (4)	0.032 (3)	
H62	0.416723	0.554598	0.787151	0.038*	
C63	0.4452 (3)	0.5591 (6)	0.7395 (4)	0.043 (3)	
H63	0.449153	0.602494	0.741357	0.051*	
C64	0.4582 (3)	0.5223 (6)	0.7093 (4)	0.031 (3)	
C65	0.4528 (3)	0.4596 (6)	0.7087 (4)	0.029 (3)	
H65	0.462556	0.434767	0.688790	0.035*	
C66	0.3560 (3)	0.3267 (5)	0.8805 (4)	0.025 (2)	
C67	0.3531 (3)	0.2654 (6)	0.8927 (4)	0.035 (3)	
H67	0.364012	0.233725	0.879416	0.042*	
C68	0.3342 (3)	0.2509 (6)	0.9242 (4)	0.039 (3)	
H68	0.332529	0.209112	0.933372	0.047*	
C69	0.3175 (3)	0.2976 (7)	0.9427 (5)	0.048 (3)	
H69	0.303643	0.287515	0.963388	0.058*	
C70	0.3213 (3)	0.3579 (6)	0.9310 (4)	0.036 (3)	
H70	0.310604	0.389505	0.944664	0.043*	
C71	0.3402 (3)	0.3740 (5)	0.8999 (4)	0.032 (3)	
C72	0.3479 (3)	0.4374 (5)	0.8832 (4)	0.035 (3)	
C73	0.1960 (3)	0.2896 (6)	0.5167 (4)	0.033 (3)	
H73C	0.170150	0.297601	0.498015	0.040*	
H73D	0.199420	0.244137	0.518381	0.040*	
C74	0.2036 (3)	0.3139 (7)	0.5688 (4)	0.048 (4)	
H74C	0.201648	0.359560	0.567557	0.057*	
H74D	0.228908	0.303286	0.588354	0.057*	
C75	0.1782 (4)	0.2884 (7)	0.5950 (5)	0.072 (5)	
H75C	0.181587	0.313572	0.624928	0.086*	
H75D	0.152960	0.295194	0.573250	0.086*	
C76	0.1817 (6)	0.2208 (8)	0.6103 (9)	0.157 (10)	
H76C	0.204210	0.214748	0.638329	0.189*	
H76D	0.183170	0.194874	0.582628	0.189*	
C77	0.1479 (5)	0.2005 (10)	0.6255 (7)	0.122 (8)	
H77C	0.125591	0.206671	0.597138	0.147*	
H77D	0.149981	0.155861	0.633496	0.147*	
C78	0.1446 (7)	0.2367 (12)	0.6697 (8)	0.198 (14)	
H78D	0.123265	0.222216	0.677484	0.297*	
H78E	0.166406	0.230056	0.698125	0.297*	
H78F	0.142004	0.280890	0.661750	0.297*	
C79	0.2109 (3)	0.2835 (6)	0.4389 (4)	0.042 (3)	
H79E	0.184288	0.283915	0.422418	0.050*	
H79F	0.218372	0.239698	0.445514	0.050*	
C80	0.2289 (3)	0.3098 (6)	0.4034 (4)	0.040 (3)	
H80E	0.216566	0.348830	0.389450	0.048*	
H80F	0.254458	0.319814	0.422036	0.048*	
C81	0.2281 (4)	0.2663 (7)	0.3616 (4)	0.052 (4)	
H81E	0.240266	0.227244	0.375729	0.063*	
H81F	0.202488	0.256419	0.343096	0.063*	
C82	0.2460 (4)	0.2913 (7)	0.3258 (4)	0.054 (4)	
H82C	0.231280	0.326169	0.307432	0.065*	
H82D	0.270162	0.307724	0.344755	0.065*	
C83	0.2504 (5)	0.2441 (9)	0.2894 (6)	0.100 (7)	
H83C	0.226178	0.228285	0.270012	0.120*	
H83D	0.264676	0.208837	0.307788	0.120*	
C84	0.2691 (6)	0.2685 (10)	0.2538 (7)	0.144 (10)	
H84D	0.270905	0.235213	0.231572	0.216*	
H84E	0.254900	0.302725	0.234568	0.216*	
H84F	0.293471	0.283240	0.272416	0.216*	
C85	0.5027 (3)	0.3980 (5)	0.4776 (3)	0.028 (3)	
H85C	0.516771	0.389722	0.455177	0.034*	
H85D	0.500227	0.443443	0.479497	0.034*	
C86	0.5242 (3)	0.3747 (5)	0.5280 (4)	0.034 (3)	
H86C	0.526489	0.329201	0.526450	0.041*	
H86D	0.510524	0.383782	0.550800	0.041*	
C87	0.5618 (3)	0.4023 (6)	0.5489 (4)	0.038 (3)	
H87C	0.559815	0.447867	0.546091	0.046*	
H87D	0.576577	0.388285	0.528610	0.046*	
C88	0.5818 (3)	0.3856 (6)	0.6025 (4)	0.050 (4)	
H88C	0.584510	0.340033	0.605265	0.059*	
H88D	0.606497	0.403643	0.612178	0.059*	
C89	0.5631 (3)	0.4078 (7)	0.6384 (4)	0.054 (4)	
H89C	0.539562	0.386184	0.631107	0.065*	
H89D	0.558119	0.452599	0.633319	0.065*	
C90	0.5855 (4)	0.3965 (8)	0.6920 (4)	0.094 (6)	
H90D	0.572168	0.411654	0.713345	0.142*	
H90E	0.590105	0.352071	0.697517	0.142*	
H90F	0.608673	0.418522	0.699730	0.142*	
C91	0.4428 (3)	0.4065 (5)	0.4086 (4)	0.029 (3)	
H91C	0.442119	0.450476	0.417804	0.035*	
H91D	0.456025	0.404264	0.384476	0.035*	
C92	0.4042 (3)	0.3848 (5)	0.3836 (4)	0.028 (3)	
H92C	0.393735	0.371239	0.409003	0.034*	
H92D	0.404566	0.348629	0.362656	0.034*	
C93	0.3797 (3)	0.4353 (6)	0.3517 (4)	0.036 (3)	
H93C	0.379264	0.471119	0.372911	0.043*	
H93D	0.390644	0.449337	0.326877	0.043*	
C94	0.3420 (3)	0.4155 (6)	0.3261 (4)	0.044 (3)	
H94C	0.331507	0.397733	0.350161	0.053*	
H94D	0.341898	0.383016	0.301769	0.053*	
C95	0.3189 (4)	0.4700 (8)	0.2999 (6)	0.084 (6)	
H95C	0.328718	0.485478	0.274350	0.101*	
H95D	0.321233	0.503772	0.323999	0.101*	
C96	0.2786 (4)	0.4556 (9)	0.2756 (6)	0.109 (7)	
H96D	0.265883	0.493027	0.259785	0.163*	
H96E	0.275786	0.423128	0.250898	0.163*	
H96F	0.268285	0.441462	0.300652	0.163*	
C97	0.6066 (4)	0.2289 (6)	0.8358 (4)	0.055 (4)	
H97C	0.632833	0.226021	0.854616	0.067*	
H97D	0.595695	0.188182	0.838195	0.067*	
C98	0.6027 (3)	0.2405 (6)	0.7820 (5)	0.056 (4)	
H98C	0.610266	0.283276	0.777728	0.067*	
H98D	0.577021	0.235324	0.761252	0.067*	
C99	0.6267 (4)	0.1938 (7)	0.7668 (5)	0.082 (5)	
H99C	0.652385	0.202749	0.785456	0.099*	
H99D	0.621149	0.151795	0.776016	0.099*	
C100	0.6220 (5)	0.1945 (8)	0.7137 (5)	0.097 (6)	
H10C	0.627966	0.236303	0.704412	0.116*	0.33
H10D	0.596320	0.185974	0.694858	0.116*	0.33
H10A	0.622913	0.237833	0.702924	0.116*	0.67
H10B	0.597879	0.177499	0.695209	0.116*	0.67
C103	0.5956 (4)	0.2555 (6)	0.9141 (4)	0.052 (4)	
H10E	0.583165	0.215212	0.912559	0.063*	
H10F	0.621831	0.248223	0.930167	0.063*	
C104	0.5826 (3)	0.2984 (6)	0.9466 (4)	0.046 (4)	
H10G	0.558503	0.314314	0.926592	0.056*	0.67
H10H	0.599279	0.334293	0.954575	0.056*	0.67
H10I	0.555942	0.303870	0.934343	0.056*	0.33
H10J	0.594617	0.339326	0.950407	0.056*	0.33
C109	0.3133 (3)	0.4708 (6)	0.8524 (4)	0.042 (3)	
H10K	0.298911	0.481493	0.873958	0.050*	
H10L	0.320306	0.510050	0.840095	0.050*	
C110	0.2896 (3)	0.4341 (6)	0.8089 (4)	0.061 (4)	
H11A	0.280861	0.396325	0.821047	0.073*	
H11B	0.304155	0.421086	0.788155	0.073*	
C111	0.2567 (4)	0.4718 (8)	0.7776 (6)	0.118 (8)	
H11C	0.245933	0.492981	0.800012	0.142*	
H11D	0.265614	0.504174	0.759984	0.142*	
C112	0.2267 (6)	0.4354 (13)	0.7397 (8)	0.210 (13)	
H11E	0.205597	0.462628	0.724495	0.252*	
H11F	0.218583	0.400530	0.755902	0.252*	
C113	0.2417 (6)	0.4106 (12)	0.7000 (9)	0.230 (13)	
H11G	0.223062	0.383475	0.677653	0.275*	
H11H	0.263079	0.384456	0.716279	0.275*	
C114	0.2530 (7)	0.4593 (14)	0.6684 (11)	0.291 (16)	
H11I	0.262070	0.438292	0.644490	0.437*	
H11J	0.231935	0.484719	0.650897	0.437*	
H11K	0.272089	0.485704	0.689657	0.437*	
C115	0.3677 (3)	0.4801 (5)	0.9259 (4)	0.037 (3)	
H11L	0.373074	0.519351	0.911854	0.045*	
H11M	0.350874	0.490022	0.944233	0.045*	
C116	0.4026 (3)	0.4568 (5)	0.9622 (4)	0.036 (3)	
H11N	0.397506	0.417836	0.976914	0.043*	
H11O	0.419741	0.446949	0.944320	0.043*	
C117	0.4207 (3)	0.5014 (6)	1.0031 (4)	0.048 (4)	
H11P	0.404597	0.507529	1.023229	0.058*	
H11Q	0.423133	0.541867	0.988341	0.058*	
C118	0.4577 (3)	0.4818 (5)	1.0367 (4)	0.038 (3)	
H11R	0.473739	0.474366	1.016740	0.046*	
H11S	0.455283	0.442278	1.052824	0.046*	
C119	0.4751 (3)	0.5294 (6)	1.0758 (4)	0.057 (4)	
H11T	0.474975	0.569891	1.059664	0.068*	
H11U	0.460067	0.533681	1.097612	0.068*	
C120	0.5134 (3)	0.5145 (6)	1.1069 (4)	0.055 (4)	
H12A	0.522671	0.547401	1.131227	0.082*	
H12B	0.513773	0.475080	1.123905	0.082*	
H12C	0.528705	0.511351	1.085893	0.082*	
C121	0.4607 (3)	0.1149 (6)	0.6556 (5)	0.047 (3)	
H12D	0.461178	0.137570	0.685346	0.071*	
H12E	0.468319	0.142498	0.633721	0.071*	
H12F	0.477357	0.079633	0.664707	0.071*	
C122	0.3334 (3)	0.5470 (6)	0.6746 (5)	0.049 (4)	
H12G	0.348528	0.522124	0.702108	0.074*	
H12H	0.314619	0.520684	0.652488	0.074*	
H12I	0.321831	0.580353	0.687276	0.074*	
C123	0.3310 (4)	0.1408 (7)	0.6956 (5)	0.056 (4)	
H12J	0.324223	0.166598	0.719353	0.083*	
H12K	0.312306	0.109282	0.682379	0.083*	
H12L	0.333081	0.166817	0.668770	0.083*	
C124	0.4945 (4)	0.5122 (6)	0.6565 (4)	0.049 (4)	
H12M	0.511817	0.484933	0.679973	0.074*	
H12N	0.507490	0.538027	0.639688	0.074*	
H12O	0.476363	0.487119	0.632333	0.074*	
C101	0.6512 (7)	0.1569 (16)	0.7016 (10)	0.083 (7)*	0.67
H10M	0.674900	0.177415	0.716952	0.099*	0.67
H10N	0.652503	0.115522	0.716800	0.099*	0.67
C102	0.6455 (8)	0.1487 (13)	0.6477 (8)	0.107 (11)	0.67
H10O	0.665416	0.124107	0.643460	0.161*	0.67
H10P	0.622452	0.127252	0.632116	0.161*	0.67
H10Q	0.644899	0.189286	0.632269	0.161*	0.67
C105	0.5792 (6)	0.2745 (9)	0.9948 (6)	0.052 (6)	0.67
H10R	0.603385	0.260907	1.016175	0.062*	0.67
H10S	0.563124	0.237767	0.987618	0.062*	0.67
C106	0.5642 (8)	0.3214 (8)	1.0229 (7)	0.050 (6)	0.67
H10T	0.541035	0.337942	1.000563	0.060*	0.67
H10U	0.581436	0.356353	1.032884	0.060*	0.67
C107	0.5576 (8)	0.2953 (10)	1.0688 (8)	0.100 (11)	0.67
H10V	0.580955	0.281672	1.092523	0.120*	0.67
H10W	0.541539	0.258726	1.059487	0.120*	0.67
C108	0.5401 (8)	0.3432 (13)	1.0936 (10)	0.101 (9)*	0.67
H10X	0.536352	0.324727	1.122721	0.151*	0.67
H10Y	0.556119	0.379209	1.103523	0.151*	0.67
H	0.516712	0.356269	1.070495	0.151*	0.67
C10A	0.5952 (14)	0.260 (2)	0.9941 (11)	0.055 (16)*	0.33
H10Z	0.583319	0.219343	0.988111	0.066*	0.33
HA	0.621712	0.253841	1.003701	0.066*	0.33
C10B	0.5864 (10)	0.292 (3)	1.0362 (16)	0.075 (17)*	0.33
H10	0.601464	0.329661	1.045261	0.090*	0.33
HB	0.593842	0.263848	1.064987	0.090*	0.33
C10C	0.5466 (10)	0.310 (2)	1.0268 (17)	0.052 (15)*	0.33
H7	0.539043	0.334868	0.996126	0.062*	0.33
HC	0.532278	0.271614	1.019867	0.062*	0.33
C10D	0.5346 (17)	0.346 (3)	1.0646 (19)	0.101 (9)*	0.33
H12	0.508353	0.353388	1.051711	0.151*	0.33
HD	0.540420	0.322377	1.095181	0.151*	0.33
HE	0.547237	0.386121	1.071257	0.151*	0.33
C125	0.6868 (12)	0.145 (3)	0.714 (2)	0.10 (2)*	0.33
H12P	0.694063	0.108423	0.699209	0.155*	0.33
H12Q	0.695513	0.182350	0.702221	0.155*	0.33
H12R	0.697399	0.142506	0.749889	0.155*	0.33
C126	0.6460 (13)	0.147 (3)	0.700 (2)	0.083 (7)*	0.33
H12S	0.639028	0.106706	0.710476	0.099*	0.33
H12T	0.637166	0.146041	0.663417	0.099*	0.33
Ir3	0.09363 (2)	0.64428 (3)	0.16694 (2)	0.04359 (17)	
Ir4	0.13022 (2)	0.60856 (3)	0.28941 (2)	0.04372 (17)	
O1'	0.2040 (4)	0.6189 (11)	0.2151 (6)	0.212 (10)	
O2'	0.0184 (3)	0.6253 (6)	0.2385 (4)	0.098 (4)	
O3'	0.0587 (3)	0.3980 (5)	0.1665 (4)	0.073 (3)	
O4'	0.1683 (3)	0.8598 (6)	0.2213 (4)	0.097 (4)	
O5'	0.1744 (3)	0.3953 (6)	0.2268 (4)	0.088 (4)	
O6'	0.0751 (3)	0.8402 (5)	0.2827 (4)	0.081 (4)	
N01'	0.1484 (3)	0.6288 (5)	0.2228 (3)	0.042 (3)	
N1'	0.0876 (3)	0.5542 (5)	0.1441 (3)	0.043 (3)	
N2'	0.0994 (3)	0.7329 (6)	0.1783 (3)	0.054 (3)	
N02'	0.0771 (3)	0.6190 (5)	0.2328 (3)	0.043 (3)	
N3'	0.1339 (3)	0.5140 (5)	0.2814 (4)	0.048 (3)	
N4'	0.1317 (3)	0.6942 (5)	0.3046 (3)	0.046 (2)	
C01'	0.1748 (4)	0.6244 (9)	0.2196 (6)	0.084 (6)	
C1'	0.1104 (3)	0.6535 (6)	0.1081 (4)	0.041 (3)	
C02'	0.0479 (4)	0.6216 (8)	0.2351 (5)	0.065 (5)	
C2'	0.1221 (3)	0.7059 (6)	0.0894 (4)	0.040 (3)	
H2'	0.121706	0.744816	0.104569	0.048*	
C3'	0.1340 (3)	0.7028 (6)	0.0502 (4)	0.038 (3)	
C4'	0.1372 (4)	0.6457 (7)	0.0292 (5)	0.052 (4)	
C5'	0.1262 (4)	0.5920 (6)	0.0456 (4)	0.050 (4)	
H5'	0.126855	0.553599	0.029785	0.060*	
C6'	0.1138 (3)	0.5954 (6)	0.0869 (5)	0.043 (3)	
C7'	0.1005 (3)	0.5421 (6)	0.1062 (4)	0.041 (3)	
C8'	0.0984 (4)	0.4825 (7)	0.0881 (5)	0.057 (4)	
H8'	0.106535	0.474131	0.060931	0.068*	
C9'	0.0847 (4)	0.4351 (7)	0.1086 (5)	0.058 (4)	
H9'	0.084220	0.393902	0.096907	0.070*	
C10'	0.0715 (4)	0.4489 (7)	0.1474 (5)	0.056 (4)	
C11'	0.0739 (3)	0.5091 (7)	0.1645 (4)	0.050 (4)	
H11'	0.065695	0.518575	0.191378	0.060*	
C12'	0.1474 (3)	0.7518 (6)	0.0250 (4)	0.038 (3)	
C13'	0.1492 (3)	0.8147 (6)	0.0308 (4)	0.043 (3)	
H13'	0.143303	0.833652	0.057058	0.052*	
C14'	0.1601 (3)	0.8510 (7)	−0.0026 (4)	0.044 (3)	
H14'	0.159504	0.894812	−0.000737	0.053*	
C15'	0.1716 (4)	0.8237 (7)	−0.0377 (5)	0.058 (4)	
H15'	0.182010	0.848220	−0.057085	0.070*	
C16'	0.1682 (4)	0.7618 (7)	−0.0452 (5)	0.055 (4)	
H16'	0.173045	0.744094	−0.072676	0.066*	
C17'	0.1577 (4)	0.7241 (7)	−0.0132 (5)	0.050 (4)	
C18'	0.1512 (4)	0.6538 (7)	−0.0148 (5)	0.055 (3)	
C19'	0.0431 (3)	0.6709 (6)	0.1287 (4)	0.036 (3)*	
C20'	0.0135 (4)	0.6339 (6)	0.1067 (4)	0.043 (3)	
H20'	0.015730	0.590199	0.105674	0.052*	
C21'	−0.0204 (4)	0.6635 (7)	0.0857 (5)	0.049 (3)	
C22'	−0.0257 (4)	0.7259 (7)	0.0856 (5)	0.047 (3)	
C23'	0.0046 (4)	0.7628 (6)	0.1074 (5)	0.048 (3)	
H23'	0.002183	0.806579	0.107053	0.058*	
C24'	0.0384 (4)	0.7358 (7)	0.1298 (4)	0.046 (3)	
C25'	0.0707 (4)	0.7708 (7)	0.1550 (5)	0.049 (3)	
C26'	0.0727 (4)	0.8367 (8)	0.1547 (5)	0.065 (4)	
H26'	0.051592	0.860560	0.139199	0.078*	
C27'	0.1067 (5)	0.8665 (8)	0.1779 (5)	0.069 (4)	
H27'	0.109255	0.910201	0.178406	0.083*	
C28'	0.1359 (4)	0.8270 (9)	0.2000 (5)	0.064 (4)	
C29'	0.1319 (4)	0.7650 (8)	0.2005 (5)	0.065 (4)	
H29'	0.152711	0.741243	0.217261	0.078*	
C30'	−0.0558 (4)	0.6327 (7)	0.0623 (5)	0.050 (3)	
C31'	−0.0676 (4)	0.5709 (7)	0.0559 (5)	0.052 (4)	
H31'	−0.050438	0.538236	0.065771	0.062*	
C32'	−0.1037 (4)	0.5576 (7)	0.0356 (5)	0.062 (4)	
H32'	−0.111650	0.515859	0.033242	0.075*	
C33'	−0.1283 (4)	0.6039 (7)	0.0187 (6)	0.068 (5)	
H33'	−0.152979	0.593490	0.002664	0.081*	
C34'	−0.1184 (4)	0.6650 (7)	0.0241 (5)	0.058 (4)	
H34'	−0.136183	0.696237	0.011861	0.070*	
C35'	−0.0828 (4)	0.6813 (7)	0.0471 (5)	0.054 (4)	
C36'	−0.0652 (4)	0.7432 (7)	0.0602 (5)	0.053 (3)	
C37'	0.1097 (3)	0.5805 (6)	0.3408 (4)	0.041 (3)	
C38'	0.0962 (4)	0.6175 (7)	0.3724 (4)	0.047 (3)	
H38'	0.098965	0.661171	0.373480	0.057*	
C39'	0.0790 (3)	0.5875 (6)	0.4016 (4)	0.036 (3)	
C40'	0.0744 (3)	0.5241 (6)	0.4016 (4)	0.040 (3)	
C41'	0.0883 (3)	0.4869 (6)	0.3731 (5)	0.046 (3)	
H41'	0.085680	0.443254	0.373605	0.055*	
C42'	0.1066 (4)	0.5148 (6)	0.3426 (5)	0.044 (3)	
C43'	0.1226 (3)	0.4799 (7)	0.3120 (4)	0.045 (3)	
C44'	0.1285 (4)	0.4153 (7)	0.3148 (5)	0.062 (4)	
H44'	0.120651	0.390550	0.336739	0.074*	
C45'	0.1461 (4)	0.3887 (8)	0.2848 (6)	0.071 (4)	
H45'	0.150110	0.345344	0.285295	0.085*	
C46'	0.1577 (4)	0.4264 (9)	0.2542 (6)	0.069 (4)	
C47'	0.1511 (3)	0.4868 (7)	0.2524 (5)	0.053 (4)	
H47'	0.158646	0.511731	0.230323	0.064*	
C48'	0.0619 (3)	0.6140 (6)	0.4360 (5)	0.038 (3)	
C49'	0.0622 (3)	0.6745 (6)	0.4526 (4)	0.038 (3)	
H49'	0.072829	0.706994	0.439653	0.045*	
C50'	0.0460 (4)	0.6862 (6)	0.4900 (5)	0.047 (3)	
H50'	0.046531	0.726655	0.503156	0.056*	
C51'	0.0302 (3)	0.6402 (7)	0.5061 (5)	0.045 (3)	
H51'	0.019064	0.649182	0.530251	0.055*	
C52'	0.0290 (4)	0.5793 (7)	0.4895 (5)	0.051 (4)	
H52'	0.017332	0.547509	0.501442	0.061*	
C53'	0.0457 (3)	0.5676 (6)	0.4547 (5)	0.044 (3)	
C54'	0.0515 (4)	0.5045 (7)	0.4334 (5)	0.056 (3)	
C55'	0.1810 (3)	0.6147 (6)	0.3346 (4)	0.032 (3)	
C56'	0.2065 (3)	0.5677 (6)	0.3539 (4)	0.033 (3)	
H56'	0.199440	0.525536	0.348596	0.040*	
C57'	0.2419 (3)	0.5826 (6)	0.3808 (4)	0.033 (3)	
C58'	0.2535 (4)	0.6437 (6)	0.3899 (4)	0.036 (3)	
C59'	0.2285 (3)	0.6912 (6)	0.3730 (4)	0.034 (3)	
H59'	0.235776	0.733131	0.379816	0.040*	
C60'	0.1925 (4)	0.6767 (6)	0.3457 (4)	0.041 (3)	
C61'	0.1635 (4)	0.7237 (7)	0.3287 (4)	0.041 (2)	
C62'	0.1677 (4)	0.7873 (7)	0.3350 (5)	0.053 (3)	
H62'	0.191123	0.804698	0.349736	0.063*	
C63'	0.1370 (4)	0.8256 (7)	0.3194 (4)	0.052 (3)	
H63'	0.139532	0.869191	0.323494	0.063*	
C64'	0.1051 (4)	0.8011 (8)	0.2991 (5)	0.054 (3)	
C65'	0.1021 (4)	0.7383 (7)	0.2906 (5)	0.055 (3)	
H65'	0.078498	0.722484	0.274142	0.066*	
C66'	0.2727 (3)	0.5424 (6)	0.4035 (4)	0.037 (3)	
C67'	0.2754 (3)	0.4781 (6)	0.4058 (4)	0.037 (3)	
H67'	0.254728	0.453062	0.390844	0.044*	
C68'	0.3084 (3)	0.4514 (6)	0.4300 (4)	0.043 (3)	
H68'	0.310106	0.407640	0.432985	0.051*	
C69'	0.3392 (4)	0.4872 (7)	0.4501 (5)	0.052 (4)	
H69'	0.361923	0.467683	0.465394	0.063*	
C70'	0.3372 (4)	0.5515 (7)	0.4480 (5)	0.050 (3)	
H70'	0.358217	0.576119	0.462390	0.059*	
C71'	0.3040 (3)	0.5788 (6)	0.4246 (5)	0.041 (3)	
C72'	0.2942 (4)	0.6467 (7)	0.4168 (5)	0.049 (3)	
C73'	0.1214 (4)	0.6329 (7)	−0.0652 (5)	0.058 (4)	
H73A	0.132400	0.635029	−0.091694	0.070*	
H73B	0.114966	0.589199	−0.061993	0.070*	
C74'	0.0876 (4)	0.6701 (7)	−0.0803 (5)	0.071 (4)	
H74A	0.091132	0.704897	−0.100566	0.085*	
H74B	0.084606	0.688158	−0.050171	0.085*	
C75'	0.0520 (5)	0.6383 (9)	−0.1087 (6)	0.102 (6)	
H75A	0.033153	0.670464	−0.121181	0.122*	
H75B	0.055200	0.617100	−0.137501	0.122*	
C76'	0.0386 (4)	0.5922 (8)	−0.0797 (6)	0.073 (5)	
H76A	0.059662	0.569354	−0.057669	0.088*	
H76B	0.022797	0.561957	−0.102629	0.088*	
C77'	0.0178 (5)	0.6212 (9)	−0.0492 (6)	0.090 (6)	
H77A	0.032671	0.654120	−0.028109	0.108*	
H77B	−0.004689	0.640419	−0.071109	0.108*	
C78'	0.0085 (4)	0.5737 (9)	−0.0178 (6)	0.099 (7)	
H78A	−0.005023	0.593208	0.001703	0.148*	
H78B	−0.006537	0.541521	−0.038757	0.148*	
H78C	0.030761	0.555200	0.004172	0.148*	
C79'	0.1849 (4)	0.6186 (8)	−0.0091 (6)	0.083 (5)	
H79C	0.196653	0.638508	−0.030993	0.100*	0.4
H79D	0.177281	0.576875	−0.022715	0.100*	0.4
H79A	0.190294	0.620036	−0.040406	0.100*	0.6
H79B	0.181051	0.574808	−0.002029	0.100*	0.6
C81'	0.2500 (7)	0.6057 (14)	0.0270 (11)	0.239 (16)*	
H81C	0.253887	0.560375	0.027794	0.287*	0.4
H81D	0.268015	0.620849	0.057723	0.287*	0.4
H81A	0.241987	0.562203	0.019875	0.287*	0.6
H81B	0.268868	0.606034	0.059756	0.287*	0.6
C82'	0.2690 (8)	0.6260 (16)	−0.0126 (12)	0.268 (18)*	
H82A	0.252349	0.612817	−0.044984	0.322*	
H82B	0.268856	0.671795	−0.012813	0.322*	
C83'	0.3074 (7)	0.6066 (12)	−0.0116 (9)	0.175 (11)*	
H83A	0.326549	0.626325	0.015908	0.210*	
H83B	0.310582	0.561151	−0.009633	0.210*	
C84'	0.3071 (8)	0.6317 (14)	−0.0612 (10)	0.253 (17)*	
H84A	0.330397	0.622668	−0.065928	0.380*	
H84B	0.287488	0.612068	−0.087380	0.380*	
H84C	0.303290	0.676566	−0.062104	0.380*	
C85'	−0.0692 (4)	0.7833 (6)	0.0155 (5)	0.056 (4)	
H85A	−0.056872	0.823201	0.026767	0.067*	
H85B	−0.095340	0.792287	−0.000479	0.067*	
C86'	−0.0544 (4)	0.7562 (7)	−0.0232 (5)	0.061 (4)	
H86A	−0.028567	0.745271	−0.006826	0.074*	
H86B	−0.067557	0.717250	−0.035567	0.074*	
C87'	−0.0567 (4)	0.7969 (6)	−0.0666 (5)	0.051 (4)	
H87A	−0.082608	0.805658	−0.084347	0.061*	
H87B	−0.044732	0.836750	−0.054329	0.061*	
C88'	−0.0399 (4)	0.7703 (8)	−0.1026 (5)	0.073 (5)	
H88A	−0.054685	0.734529	−0.119227	0.087*	
H88B	−0.015473	0.754637	−0.083979	0.087*	
C89'	−0.0364 (4)	0.8146 (7)	−0.1416 (6)	0.073 (5)	
H89A	−0.061006	0.826718	−0.162747	0.088*	
H89B	−0.023902	0.852467	−0.125249	0.088*	
C90'	−0.0160 (5)	0.7889 (8)	−0.1736 (6)	0.098 (6)	
H90A	−0.014920	0.820166	−0.197798	0.147*	
H90B	0.008635	0.777813	−0.153223	0.147*	
H90C	−0.028529	0.752023	−0.190782	0.147*	
C91'	−0.0809 (4)	0.7770 (7)	0.0955 (5)	0.064 (4)	
H91A	−0.067032	0.815671	0.106641	0.076*	
H91B	−0.106328	0.788664	0.077556	0.076*	
C92'	−0.0801 (5)	0.7393 (10)	0.1402 (7)	0.118 (8)	
H92A	−0.092219	0.699342	0.128345	0.142*	
H92B	−0.054392	0.729899	0.158538	0.142*	
C93'	−0.0958 (6)	0.7639 (11)	0.1739 (7)	0.129 (8)	
H93A	−0.120791	0.777119	0.155284	0.155*	
H93B	−0.082007	0.801447	0.188640	0.155*	
C94'	−0.0975 (8)	0.7226 (14)	0.2147 (9)	0.172 (11)	
H94A	−0.101088	0.748268	0.241328	0.207*	
H94B	−0.074125	0.700604	0.228352	0.207*	
C95'	−0.1300 (8)	0.6722 (12)	0.1973 (9)	0.165 (11)	
H95A	−0.152512	0.692104	0.176109	0.198*	
H95B	−0.123354	0.638729	0.178230	0.198*	
C96'	−0.1358 (7)	0.6456 (15)	0.2434 (10)	0.247 (18)	
H96A	−0.155341	0.614920	0.233893	0.370*	
H96B	−0.113389	0.625924	0.263960	0.370*	
H96C	−0.142432	0.679089	0.261847	0.370*	
C97'	0.0734 (4)	0.4608 (7)	0.4761 (5)	0.058 (4)	
H97A	0.057277	0.447664	0.494740	0.069*	
H97B	0.080437	0.423299	0.461677	0.069*	
C98'	0.1071 (4)	0.4896 (7)	0.5114 (5)	0.056 (4)	
H98A	0.100108	0.524470	0.528839	0.067*	
H98B	0.122402	0.506311	0.492812	0.067*	
C99'	0.1288 (5)	0.4422 (7)	0.5490 (6)	0.084 (5)	
H99A	0.112715	0.422571	0.565162	0.101*	
H99B	0.137678	0.409408	0.531683	0.101*	
C200	0.1607 (5)	0.4711 (10)	0.5876 (6)	0.111 (7)	
H20A	0.172863	0.501394	0.572325	0.134*	
H20B	0.178543	0.438915	0.604213	0.134*	
C201	0.1463 (7)	0.5033 (11)	0.6245 (8)	0.159 (10)	
H20C	0.127715	0.533865	0.606795	0.190*	
H20D	0.166385	0.526252	0.648233	0.190*	
C202	0.1283 (8)	0.4561 (13)	0.6551 (9)	0.220 (16)	
H20E	0.119606	0.480016	0.678008	0.329*	
H20F	0.107887	0.433944	0.631972	0.329*	
H20G	0.146609	0.426321	0.673467	0.329*	
C203	0.0151 (4)	0.4751 (7)	0.4020 (5)	0.066 (4)	
H20H	0.019640	0.432047	0.393719	0.079*	0.6
H20I	−0.001396	0.473295	0.421923	0.079*	0.6
H20J	0.021755	0.438555	0.385993	0.079*	0.4
H20K	0.003966	0.458592	0.425924	0.079*	0.4
C206	−0.0490 (6)	0.5164 (10)	0.2748 (6)	0.138 (9)	
H20N	−0.071651	0.495533	0.274638	0.166*	0.4
H20O	−0.050645	0.561483	0.279690	0.166*	0.4
H20L	−0.075823	0.518494	0.263123	0.166*	0.6
H20M	−0.040177	0.558883	0.284729	0.166*	0.6
C207	−0.0378 (5)	0.5005 (8)	0.2303 (6)	0.109 (7)	
H20P	−0.039181	0.455103	0.225222	0.131*	
H20Q	−0.012332	0.513365	0.236423	0.131*	
C208	−0.0625 (6)	0.5329 (10)	0.1836 (6)	0.165 (11)	
H20R	−0.054640	0.521824	0.155576	0.247*	
H20S	−0.087607	0.519596	0.177246	0.247*	
H20T	−0.060785	0.577799	0.188435	0.247*	
C209	0.3024 (4)	0.6806 (6)	0.4668 (4)	0.051 (4)	
H20U	0.328834	0.678712	0.483984	0.061*	
H20V	0.295841	0.724709	0.459990	0.061*	
C210	0.2834 (4)	0.6566 (7)	0.5013 (5)	0.058 (4)	
H21A	0.256842	0.657941	0.484329	0.070*	
H21B	0.290251	0.612781	0.509073	0.070*	
C211	0.2919 (4)	0.6921 (7)	0.5486 (5)	0.069 (5)	
H21C	0.279230	0.732454	0.541171	0.083*	0.5
H21D	0.318237	0.700890	0.560310	0.083*	0.5
H21E	0.291352	0.737063	0.541755	0.083*	0.5
H21F	0.316538	0.681306	0.570413	0.083*	0.5
C215	0.3146 (4)	0.6794 (7)	0.3854 (5)	0.063 (4)	
H21G	0.307904	0.723716	0.382548	0.075*	
H21H	0.340979	0.676631	0.402950	0.075*	
C216	0.3070 (5)	0.6533 (9)	0.3346 (6)	0.101 (7)	
H21I	0.280509	0.655697	0.317435	0.121*	0.5
H21J	0.313707	0.609004	0.337738	0.121*	0.5
H21K	0.284141	0.630336	0.330003	0.121*	0.5
H21L	0.325823	0.620917	0.340246	0.121*	0.5
C221	0.0431 (4)	0.4146 (8)	0.2036 (6)	0.093 (6)	
H22A	0.062546	0.424561	0.234159	0.139*	
H22B	0.028835	0.379881	0.209302	0.139*	
H22C	0.027395	0.450747	0.192668	0.139*	
C222	0.2012 (5)	0.8206 (10)	0.2354 (6)	0.110 (7)	
H22D	0.205608	0.804389	0.268603	0.165*	
H22E	0.222086	0.845421	0.234623	0.165*	
H22F	0.197755	0.786141	0.212170	0.165*	
C223	0.1877 (5)	0.4334 (9)	0.1955 (6)	0.097 (6)	
H22G	0.205696	0.462526	0.215653	0.146*	
H22H	0.167508	0.456423	0.172823	0.146*	
H22I	0.198997	0.407212	0.176732	0.146*	
C224	0.0389 (4)	0.8137 (9)	0.2602 (6)	0.107 (7)	
H22J	0.032774	0.787487	0.284144	0.160*	
H22K	0.038591	0.788867	0.231565	0.160*	
H22L	0.021072	0.847209	0.249735	0.160*	
C21B	0.2822 (8)	0.6626 (16)	0.5914 (10)	0.076 (8)*	0.5
H21M	0.292260	0.620143	0.595948	0.091*	0.5
H21N	0.295187	0.686058	0.621591	0.091*	0.5
C21C	0.2431 (9)	0.658 (2)	0.5887 (15)	0.16 (2)*	0.5
H21O	0.229629	0.638052	0.557170	0.188*	0.5
H21P	0.233611	0.700859	0.587443	0.188*	0.5
C21D	0.2342 (9)	0.6237 (16)	0.6298 (12)	0.086 (11)*	0.5
H21Q	0.207896	0.624419	0.623348	0.128*	0.5
H21R	0.242432	0.580732	0.630913	0.128*	0.5
H21S	0.246432	0.643829	0.661327	0.128*	0.5
C80'	0.2166 (5)	0.6437 (11)	0.0313 (8)	0.048 (7)*	0.6
H80A	0.213491	0.637174	0.063775	0.058*	0.6
H80B	0.219787	0.688451	0.026683	0.058*	0.6
C204	−0.0039 (6)	0.5106 (10)	0.3540 (7)	0.062 (8)*	0.7
H20W	−0.010593	0.552373	0.362262	0.075*	0.7
H20X	0.013236	0.515570	0.335337	0.075*	0.7
C205	−0.0381 (7)	0.4774 (13)	0.3217 (8)	0.118 (11)	0.7
H20Y	−0.032699	0.434190	0.314910	0.141*	0.7
HF	−0.057340	0.477381	0.337199	0.141*	0.7
C217	0.3262 (11)	0.684 (2)	0.3023 (11)	0.136 (13)*	0.5
H21T	0.351997	0.671619	0.313205	0.163*	0.5
H21U	0.325210	0.729772	0.305601	0.163*	0.5
C218	0.3081 (9)	0.6658 (18)	0.2483 (12)	0.096 (9)*	0.5
H21V	0.301258	0.621700	0.247999	0.116*	0.5
H21W	0.326952	0.668372	0.232143	0.116*	0.5
C219	0.2745 (10)	0.701 (2)	0.2150 (14)	0.135 (12)*	0.5
H21X	0.252287	0.687181	0.221694	0.162*	0.5
H21Y	0.277406	0.745799	0.219941	0.162*	0.5
C220	0.2731 (15)	0.683 (3)	0.1636 (16)	0.22 (2)*	0.5
H22M	0.252310	0.702889	0.139630	0.324*	0.5
H22N	0.295517	0.695880	0.158009	0.324*	0.5
H22O	0.270579	0.637686	0.159749	0.324*	0.5
C20B	−0.0156 (12)	0.507 (2)	0.3623 (11)	0.060 (18)*	0.3
H20Z	−0.012967	0.552024	0.365823	0.072*	0.3
HG	−0.039164	0.494767	0.365790	0.072*	0.3
C20C	−0.0145 (10)	0.487 (2)	0.3112 (12)	0.059 (15)*	0.3
H20	−0.015117	0.441142	0.307936	0.071*	0.3
HH	0.007664	0.502562	0.305446	0.071*	0.3
C21E	0.3026 (13)	0.6737 (18)	0.2821 (8)	0.136 (13)*	0.5
H21Z	0.326131	0.692338	0.283679	0.163*	0.5
HI	0.284892	0.708095	0.275005	0.163*	0.5
C21F	0.2912 (11)	0.6324 (16)	0.2344 (12)	0.096 (9)*	0.5
H21	0.315575	0.614089	0.240588	0.116*	0.5
HJ	0.278450	0.600936	0.247706	0.116*	0.5
C21G	0.2762 (12)	0.6099 (18)	0.1815 (11)	0.135 (12)*	0.5
H1	0.296352	0.613337	0.167772	0.162*	0.5
HK	0.257840	0.640832	0.163852	0.162*	0.5
C21H	0.2589 (14)	0.547 (2)	0.1653 (17)	0.22 (2)*	0.5
H2	0.251421	0.543513	0.129368	0.324*	0.5
HL	0.276532	0.514020	0.180011	0.324*	0.5
HM	0.237625	0.541797	0.176051	0.324*	0.5
C80B	0.2148 (7)	0.609 (2)	0.0411 (10)	0.098 (18)*	0.4
H80C	0.210945	0.570240	0.056942	0.118*	0.4
H80D	0.215689	0.644174	0.063516	0.118*	0.4
C212	0.2643 (8)	0.6770 (16)	0.5742 (12)	0.076 (8)*	0.5
H3	0.239576	0.679526	0.550157	0.091*	0.5
HN	0.268245	0.634054	0.586866	0.091*	0.5
C213	0.2673 (11)	0.7203 (18)	0.6159 (13)	0.127 (16)*	0.5
H4	0.258447	0.761697	0.602594	0.153*	0.5
HO	0.293103	0.724428	0.636240	0.153*	0.5
C214	0.2455 (12)	0.698 (2)	0.6483 (15)	0.153 (19)*	0.5
H5	0.248164	0.727636	0.675050	0.230*	0.5
HP	0.219825	0.694687	0.628490	0.230*	0.5
HQ	0.254473	0.657426	0.662128	0.230*	0.5

### Di-µ2-cyanato-bis{bis[9,9-dihexyl-2-(5-methoxypyridin-2-yl)-9H-fluoren-1-yl]iridium} pentane monosolvate (III) Atomic displacement parameters (Å^2^)

**Table:**

	*U*^11^	*U*^22^	*U*^33^	*U*^12^	*U*^13^	*U*^23^
Ir1	0.0230 (3)	0.0237 (3)	0.0155 (2)	−0.0003 (2)	0.00689 (18)	0.0022 (2)
Ir2	0.0266 (3)	0.0262 (3)	0.0161 (2)	−0.0034 (2)	0.00675 (19)	0.0027 (2)
O1	0.036 (6)	0.092 (9)	0.088 (8)	0.000 (6)	0.028 (6)	−0.004 (7)
O2	0.039 (4)	0.31 (2)	0.086 (9)	0.001 (6)	0.041 (5)	0.028 (11)
O3	0.044 (5)	0.029 (5)	0.049 (6)	0.017 (4)	0.021 (4)	0.016 (4)
O4	0.049 (6)	0.032 (5)	0.051 (6)	0.000 (4)	0.025 (4)	−0.009 (4)
O5	0.049 (5)	0.035 (5)	0.041 (5)	−0.021 (4)	0.022 (4)	−0.005 (4)
O6	0.054 (6)	0.048 (6)	0.034 (5)	−0.014 (5)	0.019 (4)	0.001 (4)
N1	0.034 (5)	0.012 (5)	0.018 (5)	0.000 (4)	0.012 (4)	0.004 (4)
N01	0.0230 (3)	0.0237 (3)	0.0155 (2)	−0.0003 (2)	0.00689 (18)	0.0022 (2)
N02	0.0230 (3)	0.0237 (3)	0.0155 (2)	−0.0003 (2)	0.00689 (18)	0.0022 (2)
N2	0.026 (6)	0.018 (5)	0.018 (5)	−0.004 (4)	0.003 (4)	−0.004 (4)
N4	0.019 (6)	0.037 (6)	0.024 (5)	−0.005 (4)	0.007 (4)	0.001 (4)
C01	0.028 (6)	0.071 (9)	0.006 (6)	−0.020 (5)	0.003 (5)	−0.001 (6)
C02	0.028 (3)	0.096 (12)	0.030 (7)	−0.010 (4)	0.015 (4)	−0.005 (8)
C2	0.020 (6)	0.039 (8)	0.021 (6)	0.000 (5)	0.007 (4)	0.007 (5)
C3	0.018 (5)	0.033 (6)	0.018 (6)	−0.001 (4)	0.003 (4)	0.002 (5)
C4	0.025 (5)	0.031 (5)	0.021 (6)	−0.002 (4)	0.009 (4)	0.002 (5)
C5	0.028 (6)	0.028 (6)	0.031 (7)	−0.008 (5)	0.004 (5)	0.000 (5)
C6	0.029 (6)	0.021 (6)	0.019 (6)	−0.003 (5)	0.009 (4)	−0.004 (5)
C7	0.031 (6)	0.023 (5)	0.032 (7)	0.005 (4)	0.019 (5)	0.006 (5)
C8	0.036 (7)	0.030 (5)	0.019 (6)	−0.006 (5)	0.004 (5)	−0.007 (5)
C9	0.053 (7)	0.029 (7)	0.021 (6)	0.002 (5)	0.013 (5)	0.005 (5)
C10	0.052 (7)	0.014 (5)	0.025 (7)	0.010 (5)	0.015 (5)	0.006 (5)
C11	0.030 (7)	0.021 (5)	0.028 (6)	0.001 (4)	0.017 (5)	0.008 (5)
C12	0.021 (6)	0.035 (5)	0.028 (6)	0.003 (4)	0.007 (5)	0.003 (5)
C13	0.025 (6)	0.037 (6)	0.030 (7)	0.006 (5)	0.006 (5)	−0.001 (5)
C14	0.026 (7)	0.046 (8)	0.043 (8)	0.012 (5)	0.013 (5)	0.015 (6)
C15	0.032 (7)	0.061 (7)	0.031 (7)	0.009 (6)	0.002 (5)	0.002 (6)
C16	0.017 (6)	0.058 (7)	0.034 (7)	0.004 (5)	0.007 (5)	−0.010 (6)
C17	0.021 (6)	0.041 (6)	0.025 (6)	0.002 (4)	0.007 (4)	−0.002 (5)
C18	0.020 (6)	0.042 (6)	0.037 (6)	−0.002 (5)	0.010 (5)	0.000 (5)
C19	0.018 (6)	0.023 (5)	0.026 (6)	0.003 (5)	0.009 (4)	0.005 (5)
C20	0.028 (7)	0.024 (6)	0.016 (6)	0.002 (5)	0.008 (5)	0.004 (5)
C21	0.030 (7)	0.026 (5)	0.016 (6)	0.003 (5)	0.007 (5)	−0.002 (5)
C22	0.036 (7)	0.026 (5)	0.015 (6)	−0.008 (5)	0.011 (5)	0.000 (4)
C23	0.023 (7)	0.012 (5)	0.028 (6)	−0.007 (5)	0.008 (5)	−0.001 (4)
C24	0.025 (7)	0.024 (5)	0.016 (6)	0.002 (5)	0.004 (4)	0.000 (4)
C25	0.041 (8)	0.027 (5)	0.023 (6)	−0.007 (5)	0.015 (5)	−0.007 (5)
C26	0.044 (8)	0.023 (5)	0.025 (7)	−0.004 (5)	0.014 (5)	−0.004 (5)
C27	0.042 (8)	0.015 (6)	0.038 (7)	−0.004 (5)	0.013 (5)	−0.009 (5)
C28	0.036 (8)	0.030 (6)	0.031 (7)	−0.009 (5)	0.012 (5)	−0.012 (5)
C29	0.024 (7)	0.029 (5)	0.024 (6)	−0.009 (5)	0.007 (5)	−0.008 (5)
C30	0.029 (7)	0.031 (5)	0.026 (6)	−0.001 (5)	0.015 (5)	−0.001 (5)
C31	0.044 (8)	0.030 (6)	0.019 (6)	−0.003 (6)	0.013 (5)	0.004 (5)
C32	0.049 (8)	0.031 (7)	0.021 (6)	−0.006 (6)	0.017 (5)	−0.007 (5)
C33	0.037 (8)	0.042 (6)	0.018 (6)	0.002 (6)	0.015 (5)	0.001 (5)
C34	0.028 (7)	0.040 (6)	0.023 (6)	−0.009 (6)	0.011 (5)	−0.009 (5)
C35	0.026 (7)	0.032 (5)	0.008 (5)	0.001 (5)	−0.001 (4)	0.002 (4)
C36	0.030 (6)	0.032 (6)	0.022 (6)	−0.004 (5)	0.011 (4)	−0.006 (5)
C37	0.028 (6)	0.029 (6)	0.014 (5)	0.002 (4)	0.008 (4)	−0.003 (4)
C38	0.031 (6)	0.019 (6)	0.019 (6)	0.003 (4)	0.003 (5)	−0.005 (5)
C39	0.027 (5)	0.025 (6)	0.025 (6)	0.000 (4)	0.004 (4)	−0.003 (5)
C40	0.032 (6)	0.031 (6)	0.031 (7)	−0.002 (4)	0.006 (5)	0.004 (5)
C41	0.032 (6)	0.018 (6)	0.030 (7)	0.001 (4)	0.014 (5)	0.004 (5)
C42	0.028 (6)	0.030 (5)	0.021 (6)	0.000 (4)	0.017 (4)	0.002 (5)
C43	0.033 (7)	0.026 (5)	0.021 (6)	−0.001 (5)	0.014 (5)	−0.011 (5)
C44	0.039 (7)	0.030 (6)	0.032 (7)	0.002 (5)	0.017 (5)	−0.004 (6)
C45	0.052 (7)	0.021 (7)	0.044 (8)	−0.007 (5)	0.031 (6)	0.003 (5)
C46	0.045 (6)	0.036 (6)	0.033 (7)	−0.016 (5)	0.025 (5)	−0.006 (5)
C47	0.030 (8)	0.037 (6)	0.019 (6)	−0.007 (5)	0.012 (5)	−0.001 (5)
C48	0.027 (6)	0.030 (6)	0.039 (7)	−0.002 (4)	0.015 (5)	−0.002 (5)
C49	0.037 (7)	0.031 (6)	0.030 (7)	−0.005 (5)	0.003 (5)	0.000 (6)
C50	0.033 (7)	0.022 (7)	0.093 (11)	−0.009 (5)	−0.002 (7)	−0.012 (7)
C51	0.030 (8)	0.039 (7)	0.101 (12)	−0.006 (6)	0.007 (7)	−0.004 (8)
C52	0.022 (7)	0.040 (7)	0.120 (14)	0.005 (6)	0.015 (7)	0.001 (8)
C53	0.023 (5)	0.040 (6)	0.041 (7)	−0.002 (5)	0.005 (5)	−0.006 (6)
C54	0.027 (6)	0.038 (6)	0.050 (7)	0.009 (5)	0.005 (5)	0.005 (6)
C55	0.023 (7)	0.019 (5)	0.022 (6)	−0.004 (5)	0.004 (5)	0.002 (4)
C56	0.029 (7)	0.025 (6)	0.021 (6)	−0.003 (5)	0.003 (5)	0.004 (5)
C57	0.023 (7)	0.029 (5)	0.024 (6)	−0.003 (5)	0.006 (5)	0.000 (5)
C58	0.035 (7)	0.026 (5)	0.020 (6)	−0.010 (5)	0.007 (5)	−0.005 (5)
C59	0.038 (8)	0.019 (6)	0.028 (7)	−0.007 (5)	0.012 (5)	−0.003 (5)
C60	0.028 (7)	0.022 (5)	0.012 (6)	−0.002 (5)	0.004 (4)	0.006 (5)
C62	0.032 (8)	0.034 (8)	0.027 (7)	0.001 (6)	0.008 (5)	−0.011 (6)
C63	0.046 (9)	0.042 (8)	0.041 (8)	−0.004 (7)	0.015 (6)	−0.003 (6)
C64	0.036 (8)	0.036 (6)	0.022 (6)	−0.008 (6)	0.010 (5)	0.007 (5)
C65	0.023 (7)	0.039 (6)	0.028 (7)	−0.002 (5)	0.010 (5)	0.001 (5)
C66	0.027 (7)	0.032 (5)	0.011 (5)	−0.001 (5)	0.001 (4)	−0.001 (4)
C67	0.039 (8)	0.035 (6)	0.033 (7)	0.010 (6)	0.016 (6)	0.008 (6)
C68	0.037 (8)	0.043 (7)	0.040 (8)	−0.001 (6)	0.015 (6)	0.011 (6)
C69	0.038 (9)	0.062 (8)	0.052 (9)	−0.009 (7)	0.027 (7)	0.001 (7)
C70	0.045 (8)	0.039 (7)	0.026 (7)	−0.011 (6)	0.015 (6)	−0.017 (5)
C71	0.045 (8)	0.038 (6)	0.013 (6)	−0.013 (5)	0.011 (5)	−0.010 (5)
C72	0.049 (7)	0.033 (6)	0.024 (6)	−0.003 (5)	0.015 (4)	−0.007 (5)
C73	0.015 (6)	0.041 (8)	0.042 (6)	−0.008 (6)	0.007 (5)	0.008 (6)
C74	0.035 (8)	0.073 (10)	0.035 (7)	0.008 (7)	0.011 (6)	0.009 (6)
C75	0.069 (12)	0.100 (12)	0.055 (10)	−0.017 (10)	0.032 (8)	−0.004 (9)
C76	0.19 (2)	0.089 (14)	0.24 (3)	−0.046 (16)	0.14 (2)	−0.006 (16)
C77	0.089 (16)	0.16 (2)	0.106 (16)	−0.038 (14)	0.011 (12)	0.038 (14)
C78	0.27 (3)	0.25 (3)	0.12 (2)	0.07 (3)	0.12 (2)	0.072 (19)
C79	0.043 (9)	0.046 (8)	0.032 (6)	0.001 (7)	0.007 (5)	0.000 (5)
C80	0.029 (8)	0.061 (9)	0.021 (6)	0.005 (7)	−0.002 (5)	−0.004 (6)
C81	0.050 (10)	0.068 (10)	0.033 (7)	0.002 (8)	0.005 (6)	−0.008 (6)
C82	0.058 (10)	0.073 (11)	0.029 (7)	0.005 (8)	0.011 (6)	−0.005 (7)
C83	0.107 (16)	0.121 (16)	0.092 (14)	−0.053 (13)	0.061 (11)	−0.062 (11)
C84	0.17 (2)	0.16 (2)	0.15 (2)	−0.073 (18)	0.124 (18)	−0.070 (16)
C85	0.038 (6)	0.026 (7)	0.025 (6)	−0.009 (5)	0.018 (4)	0.000 (5)
C86	0.047 (7)	0.020 (7)	0.032 (6)	−0.005 (5)	0.009 (5)	0.000 (5)
C87	0.042 (7)	0.043 (8)	0.029 (6)	−0.005 (6)	0.011 (5)	−0.010 (6)
C88	0.055 (9)	0.046 (9)	0.044 (7)	−0.010 (7)	0.011 (6)	−0.002 (7)
C89	0.064 (11)	0.060 (10)	0.046 (7)	0.002 (8)	0.025 (7)	0.013 (7)
C90	0.125 (16)	0.109 (16)	0.043 (8)	−0.002 (13)	0.017 (9)	0.023 (10)
C91	0.042 (6)	0.022 (6)	0.026 (6)	−0.007 (5)	0.014 (5)	−0.003 (5)
C92	0.035 (6)	0.026 (7)	0.030 (7)	0.005 (5)	0.019 (5)	0.003 (5)
C93	0.045 (7)	0.039 (8)	0.028 (7)	0.011 (6)	0.021 (5)	0.006 (5)
C94	0.054 (7)	0.060 (9)	0.016 (6)	0.000 (6)	0.006 (5)	−0.007 (6)
C95	0.069 (10)	0.116 (15)	0.064 (11)	0.032 (10)	0.016 (8)	0.034 (10)
C96	0.072 (11)	0.124 (18)	0.114 (16)	0.040 (12)	0.008 (10)	0.000 (13)
C97	0.044 (9)	0.044 (8)	0.074 (8)	0.001 (7)	0.013 (7)	−0.014 (7)
C98	0.039 (9)	0.054 (10)	0.079 (8)	−0.007 (7)	0.025 (7)	−0.016 (8)
C99	0.115 (15)	0.051 (11)	0.098 (11)	−0.002 (10)	0.058 (11)	−0.032 (10)
C100	0.133 (18)	0.085 (15)	0.088 (11)	−0.016 (13)	0.058 (12)	−0.021 (11)
C103	0.049 (10)	0.051 (9)	0.047 (7)	0.005 (7)	0.002 (6)	0.009 (6)
C104	0.043 (9)	0.055 (9)	0.035 (7)	0.003 (7)	0.002 (6)	0.010 (6)
C109	0.039 (7)	0.045 (8)	0.045 (8)	−0.002 (6)	0.019 (5)	−0.002 (6)
C110	0.051 (10)	0.068 (11)	0.050 (9)	0.001 (8)	−0.001 (6)	0.009 (7)
C111	0.079 (14)	0.128 (19)	0.106 (15)	0.007 (11)	−0.027 (10)	0.036 (12)
C112	0.10 (2)	0.38 (4)	0.102 (17)	−0.06 (2)	−0.031 (11)	0.004 (18)
C113	0.15 (2)	0.36 (3)	0.18 (2)	−0.18 (2)	0.052 (18)	−0.05 (2)
C114	0.22 (3)	0.37 (4)	0.33 (3)	−0.21 (3)	0.16 (3)	−0.04 (3)
C115	0.054 (8)	0.034 (7)	0.027 (6)	0.003 (6)	0.018 (5)	−0.001 (5)
C116	0.049 (8)	0.034 (8)	0.025 (6)	−0.006 (6)	0.013 (5)	−0.003 (5)
C117	0.061 (9)	0.045 (9)	0.029 (7)	−0.001 (7)	0.002 (5)	0.001 (6)
C118	0.050 (8)	0.039 (8)	0.023 (6)	−0.015 (6)	0.009 (5)	−0.002 (5)
C119	0.058 (9)	0.049 (9)	0.051 (9)	−0.016 (7)	0.000 (6)	−0.005 (7)
C120	0.053 (9)	0.056 (10)	0.050 (9)	−0.021 (8)	0.010 (6)	0.012 (7)
C121	0.050 (7)	0.047 (9)	0.049 (9)	0.014 (7)	0.022 (6)	0.008 (7)
C122	0.041 (9)	0.046 (9)	0.072 (10)	−0.005 (7)	0.033 (7)	−0.014 (7)
C123	0.050 (8)	0.072 (11)	0.042 (9)	−0.024 (7)	0.011 (6)	−0.001 (7)
C124	0.078 (11)	0.045 (9)	0.039 (8)	−0.015 (7)	0.039 (7)	0.004 (7)
C102	0.14 (3)	0.11 (3)	0.09 (2)	0.05 (2)	0.06 (2)	0.023 (19)
C105	0.043 (17)	0.064 (15)	0.048 (11)	−0.010 (12)	0.013 (10)	−0.004 (10)
C106	0.076 (18)	0.033 (13)	0.047 (12)	−0.026 (12)	0.030 (12)	0.001 (9)
C107	0.22 (3)	0.050 (17)	0.046 (15)	−0.018 (19)	0.068 (18)	−0.007 (12)
Ir3	0.0371 (4)	0.0704 (4)	0.0235 (3)	−0.0001 (3)	0.0102 (2)	0.0080 (3)
Ir4	0.0394 (4)	0.0702 (4)	0.0224 (3)	0.0159 (3)	0.0112 (2)	0.0049 (3)
O1'	0.045 (9)	0.47 (3)	0.136 (14)	0.020 (13)	0.052 (9)	0.001 (17)
O2'	0.036 (7)	0.166 (13)	0.096 (9)	−0.004 (7)	0.026 (6)	−0.006 (8)
O3'	0.073 (8)	0.082 (8)	0.063 (7)	−0.033 (6)	0.021 (6)	0.015 (6)
O4'	0.083 (8)	0.146 (12)	0.061 (8)	−0.060 (8)	0.021 (6)	−0.028 (7)
O5'	0.081 (9)	0.121 (10)	0.068 (8)	0.032 (8)	0.031 (6)	−0.031 (7)
O6'	0.090 (7)	0.090 (9)	0.074 (8)	0.054 (7)	0.043 (6)	0.034 (7)
N01'	0.022 (6)	0.084 (9)	0.019 (5)	0.006 (6)	0.004 (4)	−0.009 (5)
N1'	0.041 (7)	0.058 (7)	0.022 (5)	−0.011 (5)	0.000 (4)	0.015 (5)
N2'	0.034 (6)	0.118 (9)	0.014 (6)	0.007 (5)	0.013 (4)	−0.002 (5)
N02'	0.029 (6)	0.083 (9)	0.021 (5)	0.017 (6)	0.013 (5)	0.022 (5)
N3'	0.045 (7)	0.071 (7)	0.027 (6)	0.018 (6)	0.010 (5)	−0.004 (5)
N4'	0.048 (4)	0.071 (4)	0.026 (5)	0.012 (3)	0.022 (3)	0.026 (4)
C01'	0.027 (8)	0.161 (19)	0.067 (11)	0.001 (9)	0.020 (8)	0.000 (11)
C1'	0.039 (8)	0.055 (7)	0.029 (7)	0.010 (7)	0.011 (6)	0.014 (5)
C02'	0.038 (8)	0.119 (15)	0.038 (9)	0.012 (9)	0.014 (7)	0.014 (9)
C2'	0.035 (8)	0.054 (8)	0.037 (7)	−0.001 (7)	0.018 (6)	0.002 (6)
C3'	0.038 (8)	0.053 (7)	0.023 (6)	0.001 (6)	0.011 (5)	0.003 (5)
C4'	0.071 (10)	0.051 (7)	0.039 (7)	−0.016 (7)	0.027 (7)	−0.004 (6)
C5'	0.074 (11)	0.049 (8)	0.031 (7)	−0.004 (7)	0.023 (7)	−0.001 (6)
C6'	0.047 (9)	0.046 (7)	0.037 (7)	−0.004 (7)	0.016 (6)	0.010 (6)
C7'	0.043 (8)	0.052 (7)	0.021 (6)	−0.002 (6)	0.001 (5)	0.020 (5)
C8'	0.066 (11)	0.047 (7)	0.065 (10)	0.005 (8)	0.032 (8)	0.018 (7)
C9'	0.051 (10)	0.059 (9)	0.059 (9)	−0.006 (8)	0.011 (7)	0.017 (7)
C10'	0.040 (9)	0.063 (8)	0.060 (9)	−0.005 (7)	0.010 (7)	0.024 (7)
C11'	0.047 (9)	0.072 (8)	0.025 (7)	−0.010 (8)	0.003 (6)	0.023 (6)
C12'	0.038 (8)	0.047 (6)	0.034 (7)	0.002 (6)	0.019 (6)	0.001 (6)
C13'	0.055 (9)	0.047 (6)	0.035 (7)	−0.005 (7)	0.025 (6)	−0.004 (6)
C14'	0.048 (9)	0.057 (8)	0.030 (7)	−0.019 (7)	0.014 (6)	−0.003 (6)
C15'	0.066 (11)	0.065 (8)	0.056 (9)	−0.039 (8)	0.035 (8)	−0.017 (7)
C16'	0.070 (11)	0.058 (8)	0.048 (9)	−0.022 (8)	0.035 (8)	−0.009 (7)
C17'	0.053 (10)	0.059 (7)	0.048 (8)	−0.011 (7)	0.034 (7)	−0.011 (6)
C18'	0.079 (9)	0.050 (7)	0.052 (8)	−0.006 (7)	0.042 (6)	−0.009 (7)
C20'	0.054 (8)	0.049 (9)	0.030 (7)	0.012 (7)	0.019 (6)	0.000 (6)
C21'	0.045 (7)	0.052 (7)	0.047 (8)	0.003 (5)	0.013 (6)	−0.005 (7)
C22'	0.048 (7)	0.054 (7)	0.039 (8)	0.004 (5)	0.013 (6)	0.011 (6)
C23'	0.059 (7)	0.040 (8)	0.044 (8)	0.008 (6)	0.014 (6)	−0.005 (6)
C24'	0.037 (7)	0.081 (10)	0.022 (7)	−0.008 (6)	0.014 (5)	0.001 (6)
C25'	0.050 (7)	0.075 (8)	0.026 (7)	−0.014 (6)	0.020 (5)	−0.007 (6)
C26'	0.077 (10)	0.079 (8)	0.039 (9)	−0.014 (8)	0.018 (7)	−0.006 (8)
C27'	0.086 (10)	0.068 (10)	0.056 (10)	−0.029 (7)	0.025 (8)	−0.006 (8)
C28'	0.063 (8)	0.101 (10)	0.033 (8)	−0.023 (7)	0.022 (7)	−0.003 (8)
C29'	0.052 (9)	0.098 (10)	0.047 (9)	−0.001 (8)	0.019 (7)	−0.002 (9)
C30'	0.049 (7)	0.051 (7)	0.045 (8)	0.000 (5)	0.009 (6)	0.012 (7)
C31'	0.057 (8)	0.045 (7)	0.052 (9)	0.008 (6)	0.016 (7)	0.017 (7)
C32'	0.065 (9)	0.048 (9)	0.066 (11)	−0.003 (7)	0.011 (8)	0.013 (8)
C33'	0.051 (9)	0.054 (8)	0.084 (12)	−0.007 (6)	0.001 (8)	0.023 (8)
C34'	0.040 (7)	0.050 (7)	0.081 (11)	0.009 (6)	0.014 (7)	0.016 (8)
C35'	0.042 (7)	0.054 (7)	0.066 (10)	0.001 (5)	0.015 (6)	0.011 (7)
C36'	0.048 (7)	0.046 (7)	0.063 (9)	0.008 (6)	0.017 (6)	0.018 (6)
C37'	0.037 (8)	0.054 (7)	0.031 (7)	0.012 (6)	0.011 (5)	0.002 (6)
C38'	0.055 (10)	0.059 (8)	0.032 (8)	0.007 (7)	0.020 (6)	0.003 (6)
C39'	0.030 (8)	0.045 (6)	0.032 (7)	0.000 (6)	0.008 (5)	−0.008 (6)
C40'	0.046 (8)	0.040 (6)	0.031 (7)	−0.006 (6)	0.008 (6)	−0.004 (5)
C41'	0.048 (9)	0.043 (8)	0.040 (8)	−0.004 (7)	0.007 (6)	−0.010 (6)
C42'	0.047 (9)	0.052 (7)	0.033 (7)	−0.001 (7)	0.012 (6)	−0.007 (6)
C43'	0.038 (8)	0.068 (8)	0.025 (7)	0.022 (7)	0.003 (5)	0.002 (6)
C44'	0.061 (11)	0.062 (8)	0.060 (10)	0.019 (8)	0.017 (7)	−0.014 (7)
C45'	0.061 (11)	0.081 (11)	0.073 (12)	0.003 (9)	0.025 (8)	−0.033 (8)
C46'	0.051 (11)	0.093 (10)	0.063 (11)	0.027 (9)	0.018 (8)	−0.017 (8)
C47'	0.029 (8)	0.089 (9)	0.035 (8)	0.021 (8)	0.001 (6)	−0.019 (7)
C48'	0.032 (8)	0.041 (6)	0.045 (8)	−0.003 (6)	0.021 (6)	−0.002 (6)
C49'	0.035 (8)	0.039 (7)	0.044 (8)	0.004 (6)	0.019 (6)	0.004 (6)
C50'	0.051 (10)	0.049 (8)	0.044 (8)	0.008 (7)	0.021 (7)	0.003 (6)
C51'	0.044 (9)	0.067 (8)	0.034 (8)	−0.005 (7)	0.025 (6)	0.000 (6)
C52'	0.063 (10)	0.059 (8)	0.040 (8)	0.007 (8)	0.029 (7)	0.015 (7)
C53'	0.039 (9)	0.053 (7)	0.045 (8)	−0.008 (6)	0.018 (6)	0.006 (6)
C54'	0.062 (9)	0.052 (7)	0.057 (9)	−0.010 (7)	0.024 (6)	0.004 (6)
C55'	0.032 (7)	0.039 (6)	0.030 (6)	0.009 (5)	0.017 (5)	0.008 (5)
C56'	0.037 (6)	0.037 (7)	0.024 (6)	0.006 (5)	0.007 (5)	−0.002 (5)
C57'	0.039 (6)	0.046 (6)	0.015 (6)	−0.003 (5)	0.009 (4)	0.009 (5)
C58'	0.048 (6)	0.042 (6)	0.026 (6)	−0.001 (5)	0.021 (5)	0.014 (5)
C59'	0.052 (6)	0.033 (7)	0.023 (6)	−0.001 (5)	0.022 (5)	−0.001 (5)
C60'	0.050 (5)	0.049 (5)	0.026 (6)	0.010 (4)	0.015 (4)	0.003 (5)
C61'	0.053 (4)	0.057 (4)	0.021 (5)	0.016 (3)	0.025 (4)	0.013 (4)
C62'	0.068 (6)	0.056 (4)	0.044 (7)	0.019 (4)	0.031 (5)	0.012 (4)
C63'	0.072 (6)	0.064 (6)	0.033 (7)	0.024 (4)	0.033 (5)	0.015 (5)
C64'	0.070 (5)	0.076 (5)	0.030 (7)	0.022 (5)	0.034 (5)	0.018 (5)
C65'	0.059 (5)	0.077 (6)	0.031 (7)	0.022 (4)	0.018 (5)	0.014 (5)
C66'	0.039 (6)	0.047 (6)	0.026 (7)	0.001 (5)	0.012 (5)	0.009 (5)
C67'	0.034 (7)	0.046 (6)	0.034 (7)	0.007 (6)	0.018 (5)	−0.001 (6)
C68'	0.047 (7)	0.053 (8)	0.039 (8)	0.016 (6)	0.029 (6)	0.025 (6)
C69'	0.038 (8)	0.081 (8)	0.037 (8)	0.017 (7)	0.012 (6)	0.013 (7)
C70'	0.034 (7)	0.076 (8)	0.039 (8)	0.014 (6)	0.013 (6)	0.010 (7)
C71'	0.033 (6)	0.051 (7)	0.043 (8)	−0.001 (5)	0.017 (5)	0.010 (6)
C72'	0.046 (7)	0.052 (7)	0.048 (7)	−0.001 (6)	0.013 (6)	0.004 (6)
C73'	0.095 (10)	0.049 (9)	0.038 (7)	−0.020 (7)	0.031 (7)	0.003 (6)
C74'	0.104 (11)	0.043 (10)	0.060 (10)	−0.018 (7)	0.020 (8)	−0.007 (8)
C75'	0.098 (12)	0.106 (16)	0.075 (13)	−0.023 (10)	−0.007 (9)	0.041 (10)
C76'	0.069 (12)	0.077 (12)	0.069 (11)	−0.005 (9)	0.018 (8)	0.009 (9)
C77'	0.083 (15)	0.107 (16)	0.081 (14)	0.008 (11)	0.030 (10)	−0.006 (10)
C78'	0.053 (12)	0.154 (19)	0.086 (14)	−0.006 (12)	0.017 (9)	0.015 (12)
C79'	0.088 (12)	0.075 (12)	0.098 (14)	0.007 (9)	0.046 (10)	−0.004 (11)
C85'	0.060 (10)	0.049 (9)	0.052 (8)	−0.006 (7)	0.008 (7)	0.004 (6)
C86'	0.052 (10)	0.083 (12)	0.041 (8)	−0.007 (8)	0.004 (7)	0.015 (7)
C87'	0.043 (9)	0.051 (10)	0.049 (8)	−0.009 (7)	0.002 (6)	0.014 (7)
C88'	0.079 (13)	0.085 (13)	0.051 (10)	0.008 (10)	0.015 (8)	0.026 (8)
C89'	0.083 (13)	0.074 (12)	0.060 (11)	−0.012 (9)	0.021 (8)	0.011 (8)
C90'	0.127 (18)	0.088 (15)	0.095 (15)	0.010 (12)	0.060 (12)	0.033 (11)
C91'	0.071 (11)	0.049 (10)	0.071 (10)	0.016 (8)	0.023 (8)	0.002 (7)
C92'	0.128 (19)	0.15 (2)	0.094 (15)	0.037 (15)	0.062 (13)	0.036 (13)
C93'	0.136 (19)	0.18 (2)	0.077 (14)	0.066 (15)	0.037 (12)	0.030 (13)
C94'	0.22 (3)	0.22 (3)	0.10 (2)	0.037 (19)	0.07 (2)	0.041 (17)
C95'	0.21 (3)	0.16 (3)	0.13 (2)	0.085 (16)	0.06 (2)	0.068 (19)
C96'	0.21 (3)	0.39 (5)	0.16 (3)	−0.05 (3)	0.07 (2)	0.07 (3)
C97'	0.074 (10)	0.051 (9)	0.064 (9)	0.002 (7)	0.044 (6)	0.013 (7)
C98'	0.068 (10)	0.062 (10)	0.050 (9)	0.014 (7)	0.038 (6)	0.011 (7)
C99'	0.114 (15)	0.051 (11)	0.077 (11)	0.022 (9)	0.017 (8)	0.019 (8)
C200	0.112 (16)	0.14 (2)	0.066 (12)	0.042 (13)	0.009 (9)	−0.006 (11)
C201	0.25 (3)	0.13 (2)	0.086 (16)	0.085 (19)	0.051 (16)	0.001 (13)
C202	0.34 (4)	0.20 (3)	0.20 (3)	0.09 (3)	0.19 (3)	0.01 (2)
C203	0.072 (10)	0.059 (11)	0.075 (11)	−0.019 (8)	0.034 (7)	−0.013 (8)
C206	0.20 (2)	0.14 (2)	0.049 (11)	0.015 (17)	0.003 (13)	−0.018 (11)
C207	0.15 (2)	0.085 (15)	0.077 (12)	0.008 (13)	0.013 (12)	−0.007 (11)
C208	0.24 (3)	0.19 (3)	0.037 (11)	0.04 (2)	0.017 (14)	−0.003 (13)
C209	0.047 (9)	0.058 (9)	0.039 (7)	−0.013 (7)	0.001 (6)	0.004 (6)
C210	0.066 (11)	0.053 (10)	0.053 (8)	−0.002 (8)	0.017 (7)	−0.001 (7)
C211	0.071 (12)	0.071 (12)	0.059 (9)	−0.008 (9)	0.015 (8)	−0.006 (8)
C215	0.068 (10)	0.048 (10)	0.082 (10)	−0.006 (8)	0.038 (9)	0.017 (8)
C216	0.125 (17)	0.122 (17)	0.090 (11)	0.003 (13)	0.082 (12)	0.007 (11)
C221	0.087 (14)	0.128 (17)	0.067 (12)	−0.016 (12)	0.032 (9)	0.037 (11)
C222	0.069 (11)	0.22 (2)	0.051 (11)	−0.054 (11)	0.027 (9)	−0.024 (12)
C223	0.089 (15)	0.139 (18)	0.070 (13)	0.040 (12)	0.034 (10)	−0.014 (11)
C224	0.064 (9)	0.17 (2)	0.099 (15)	0.062 (11)	0.039 (10)	0.051 (13)
C205	0.07 (2)	0.16 (3)	0.105 (19)	−0.03 (2)	0.000 (16)	0.033 (18)

### Di-µ2-cyanato-bis{bis[9,9-dihexyl-2-(5-methoxypyridin-2-yl)-9H-fluoren-1-yl]iridium} pentane monosolvate (III) Geometric parameters (Å, º)

**Table:**

Ir1—N1	2.041 (8)	Ir4—C55'	1.990 (12)
Ir1—N01	2.237 (9)	O1'—C01'	1.191 (17)
Ir1—N02	2.222 (8)	O2'—C02'	1.185 (16)
Ir1—N2	2.045 (9)	O3'—C10'	1.393 (16)
Ir1—C1	1.981 (9)	O3'—C221	1.433 (17)
Ir1—C19	1.986 (10)	O4'—C28'	1.404 (18)
Ir2—N01	2.255 (8)	O4'—C222	1.48 (2)
Ir2—N02	2.211 (8)	O5'—C46'	1.353 (16)
Ir2—N3	2.061 (9)	O5'—C223	1.435 (19)
Ir2—N4	2.015 (9)	O6'—C64'	1.394 (16)
Ir2—C37	1.969 (11)	O6'—C224	1.464 (19)
Ir2—C55	1.990 (11)	N01'—C01'	1.068 (16)
O1—C01	1.222 (14)	N1'—C7'	1.365 (15)
O2—C02	1.190 (14)	N1'—C11'	1.334 (15)
O3—C10	1.370 (13)	N2'—C25'	1.373 (17)
O3—C121	1.411 (14)	N2'—C29'	1.404 (17)
O4—C28	1.346 (13)	N02'—C02'	1.162 (16)
O4—C122	1.439 (13)	N3'—C43'	1.328 (16)
O5—C46	1.372 (13)	N3'—C47'	1.362 (14)
O5—C123	1.436 (14)	N4'—C61'	1.365 (16)
O6—C64	1.370 (12)	N4'—C65'	1.450 (16)
O6—C124	1.438 (13)	C1'—C2'	1.392 (16)
N1—C7	1.373 (13)	C1'—C6'	1.420 (17)
N1—C11	1.361 (13)	C2'—H2'	0.9500
N01—C01	1.082 (13)	C2'—C3'	1.352 (15)
N02—C02	1.155 (13)	C3'—C4'	1.398 (17)
N2—C25	1.363 (13)	C3'—C12'	1.471 (16)
N2—C29	1.373 (13)	C4'—C5'	1.374 (17)
N3—C43	1.351 (13)	C4'—C18'	1.540 (17)
N3—C47	1.353 (13)	C5'—H5'	0.9500
N4—C61	1.363 (13)	C5'—C6'	1.421 (16)
N4—C65	1.339 (13)	C6'—C7'	1.448 (16)
C1—C2	1.418 (14)	C7'—C8'	1.384 (18)
C1—C6	1.411 (14)	C8'—H8'	0.9500
C2—H2A	0.9500	C8'—C9'	1.374 (17)
C2—C3	1.370 (14)	C9'—H9'	0.9500
C3—C4	1.386 (15)	C9'—C10'	1.404 (19)
C3—C12	1.482 (15)	C10'—C11'	1.384 (19)
C4—C5	1.387 (15)	C11'—H11'	0.9500
C4—C18	1.537 (15)	C12'—C13'	1.369 (16)
C5—H5A	0.9500	C12'—C17'	1.419 (16)
C5—C6	1.410 (14)	C13'—H13'	0.9500
C6—C7	1.463 (15)	C13'—C14'	1.410 (16)
C7—C8	1.386 (15)	C14'—H14'	0.9500
C8—H8	0.9500	C14'—C15'	1.363 (17)
C8—C9	1.338 (15)	C15'—H15'	0.9500
C9—H9	0.9500	C15'—C16'	1.354 (18)
C9—C10	1.399 (16)	C16'—H16'	0.9500
C10—C11	1.376 (15)	C16'—C17'	1.388 (17)
C11—H11	0.9500	C17'—C18'	1.539 (18)
C12—C13	1.354 (15)	C18'—C73'	1.601 (19)
C12—C17	1.391 (15)	C18'—C79'	1.480 (19)
C13—H13	0.9500	C19'—C20'	1.379 (16)
C13—C14	1.377 (15)	C19'—C24'	1.417 (17)
C14—H14	0.9500	C20'—H20'	0.9500
C14—C15	1.406 (16)	C20'—C21'	1.415 (17)
C15—H15	0.9500	C21'—C22'	1.364 (18)
C15—C16	1.361 (17)	C21'—C30'	1.483 (18)
C16—H16	0.9500	C22'—C23'	1.395 (17)
C16—C17	1.379 (15)	C22'—C36'	1.522 (18)
C17—C18	1.536 (16)	C23'—H23'	0.9500
C18—C73	1.533 (11)	C23'—C24'	1.394 (17)
C18—C79	1.523 (15)	C24'—C25'	1.446 (17)
C19—C20	1.390 (14)	C25'—C26'	1.426 (19)
C19—C24	1.429 (15)	C26'—H26'	0.9500
C20—H6	0.9500	C26'—C27'	1.429 (19)
C20—C21	1.379 (14)	C27'—H27'	0.9500
C21—C22	1.383 (14)	C27'—C28'	1.40 (2)
C21—C30	1.502 (14)	C28'—C29'	1.35 (2)
C22—C23	1.379 (14)	C29'—H29'	0.9500
C22—C36	1.546 (14)	C30'—C31'	1.404 (18)
C23—H23	0.9500	C30'—C35'	1.450 (18)
C23—C24	1.407 (13)	C31'—H31'	0.9500
C24—C25	1.469 (14)	C31'—C32'	1.367 (18)
C25—C26	1.382 (15)	C32'—H32'	0.9500
C26—H26	0.9500	C32'—C33'	1.360 (18)
C26—C27	1.376 (14)	C33'—H33'	0.9500
C27—H27	0.9500	C33'—C34'	1.371 (18)
C27—C28	1.392 (15)	C34'—H34'	0.9500
C28—C29	1.367 (15)	C34'—C35'	1.374 (18)
C29—H29	0.9500	C35'—C36'	1.495 (19)
C30—C31	1.373 (15)	C36'—C85'	1.516 (17)
C30—C35	1.405 (14)	C36'—C91'	1.534 (18)
C31—H31	0.9500	C37'—C38'	1.432 (16)
C31—C32	1.388 (14)	C37'—C42'	1.427 (18)
C32—H32	0.9500	C38'—H38'	0.9500
C32—C33	1.365 (14)	C38'—C39'	1.392 (16)
C33—H33	0.9500	C39'—C40'	1.383 (17)
C33—C34	1.374 (15)	C39'—C48'	1.478 (16)
C34—H34	0.9500	C40'—C41'	1.377 (16)
C34—C35	1.398 (14)	C40'—C54'	1.530 (17)
C35—C36	1.498 (15)	C41'—H41'	0.9500
C36—C85	1.541 (10)	C41'—C42'	1.430 (17)
C36—C91	1.538 (15)	C42'—C43'	1.444 (17)
C37—C38	1.394 (14)	C43'—C44'	1.414 (18)
C37—C42	1.443 (15)	C44'—H44'	0.9500
C38—H38	0.9500	C44'—C45'	1.390 (18)
C38—C39	1.411 (14)	C45'—H45'	0.9500
C39—C40	1.409 (15)	C45'—C46'	1.38 (2)
C39—C48	1.439 (15)	C46'—C47'	1.33 (2)
C40—C41	1.386 (15)	C47'—H47'	0.9500
C40—C54	1.525 (15)	C48'—C49'	1.390 (16)
C41—H41	0.9500	C48'—C53'	1.382 (16)
C41—C42	1.418 (14)	C49'—H49'	0.9500
C42—C43	1.453 (15)	C49'—C50'	1.435 (16)
C43—C44	1.384 (15)	C50'—H50'	0.9500
C44—H44	0.9500	C50'—C51'	1.331 (17)
C44—C45	1.367 (15)	C51'—H51'	0.9500
C45—H45	0.9500	C51'—C52'	1.395 (18)
C45—C46	1.395 (16)	C52'—H52'	0.9500
C46—C47	1.386 (16)	C52'—C53'	1.382 (16)
C47—H47	0.9500	C53'—C54'	1.542 (18)
C48—C49	1.378 (15)	C54'—C97'	1.564 (18)
C48—C53	1.394 (16)	C54'—C203	1.551 (12)
C49—H49	0.9500	C55'—C56'	1.404 (15)
C49—C50	1.417 (16)	C55'—C60'	1.417 (17)
C50—H50	0.9500	C56'—H56'	0.9500
C50—C51	1.380 (17)	C56'—C57'	1.383 (15)
C51—H51	0.9500	C57'—C58'	1.394 (16)
C51—C52	1.371 (18)	C57'—C66'	1.453 (16)
C52—H52	0.9500	C58'—C59'	1.390 (16)
C52—C53	1.366 (16)	C58'—C72'	1.525 (17)
C53—C54	1.520 (16)	C59'—H59'	0.9500
C54—C97	1.531 (11)	C59'—C60'	1.405 (16)
C54—C103	1.543 (11)	C60'—C61'	1.479 (17)
C55—C56	1.439 (14)	C61'—C62'	1.390 (18)
C55—C60	1.406 (14)	C62'—H62'	0.9500
C56—H56	0.9500	C62'—C63'	1.403 (17)
C56—C57	1.397 (14)	C63'—H63'	0.9500
C57—C58	1.396 (15)	C63'—C64'	1.305 (19)
C57—C66	1.470 (14)	C64'—C65'	1.377 (19)
C58—C59	1.389 (14)	C65'—H65'	0.9500
C58—C72	1.521 (15)	C66'—C67'	1.394 (16)
C59—H59	0.9500	C66'—C71'	1.413 (17)
C59—C60	1.409 (14)	C67'—H67'	0.9500
C60—C61	1.471 (14)	C67'—C68'	1.374 (15)
C61—C62	1.390 (15)	C68'—H68'	0.9500
C62—H62	0.9500	C68'—C69'	1.389 (17)
C62—C63	1.382 (15)	C69'—H69'	0.9500
C63—H63	0.9500	C69'—C70'	1.392 (18)
C63—C64	1.389 (16)	C70'—H70'	0.9500
C64—C65	1.371 (15)	C70'—C71'	1.382 (16)
C65—H65	0.9500	C71'—C72'	1.515 (18)
C66—C67	1.383 (15)	C72'—C209	1.551 (17)
C66—C71	1.399 (15)	C72'—C215	1.551 (17)
C67—H67	0.9500	C73'—H73A	0.9900
C67—C68	1.376 (15)	C73'—H73B	0.9900
C68—H68	0.9500	C73'—C74'	1.482 (19)
C68—C69	1.396 (17)	C74'—H74A	0.9900
C69—H69	0.9500	C74'—H74B	0.9900
C69—C70	1.366 (17)	C74'—C75'	1.52 (2)
C70—H70	0.9500	C75'—H75A	0.9900
C70—C71	1.374 (15)	C75'—H75B	0.9900
C71—C72	1.515 (15)	C75'—C76'	1.497 (19)
C72—C109	1.532 (11)	C76'—H76A	0.9900
C72—C115	1.529 (11)	C76'—H76B	0.9900
C73—H73C	0.9900	C76'—C77'	1.512 (16)
C73—H73D	0.9900	C77'—H77A	0.9900
C73—C74	1.523 (11)	C77'—H77B	0.9900
C74—H74C	0.9900	C77'—C78'	1.487 (16)
C74—H74D	0.9900	C78'—H78A	0.9800
C74—C75	1.531 (12)	C78'—H78B	0.9800
C75—H75C	0.9900	C78'—H78C	0.9800
C75—H75D	0.9900	C79'—H79C	0.9900
C75—C76	1.518 (14)	C79'—H79D	0.9900
C76—H76C	0.9900	C79'—H79A	0.9900
C76—H76D	0.9900	C79'—H79B	0.9900
C76—C77	1.576 (14)	C79'—C80'	1.498 (16)
C77—H77C	0.9900	C79'—C80B	1.548 (19)
C77—H77D	0.9900	C81'—H81C	0.9900
C77—C78	1.534 (15)	C81'—H81D	0.9900
C78—H78D	0.9800	C81'—H81A	0.9900
C78—H78E	0.9800	C81'—H81B	0.9900
C78—H78F	0.9800	C81'—C82'	1.608 (18)
C79—H79E	0.9900	C81'—C80'	1.576 (18)
C79—H79F	0.9900	C81'—C80B	1.550 (19)
C79—C80	1.523 (15)	C82'—H82A	0.9900
C80—H80E	0.9900	C82'—H82B	0.9900
C80—H80F	0.9900	C82'—C83'	1.546 (18)
C80—C81	1.519 (16)	C83'—H83A	0.9900
C81—H81E	0.9900	C83'—H83B	0.9900
C81—H81F	0.9900	C83'—C84'	1.521 (17)
C81—C82	1.516 (17)	C84'—H84A	0.9800
C82—H82C	0.9900	C84'—H84B	0.9800
C82—H82D	0.9900	C84'—H84C	0.9800
C82—C83	1.512 (18)	C85'—H85A	0.9900
C83—H83C	0.9900	C85'—H85B	0.9900
C83—H83D	0.9900	C85'—C86'	1.528 (18)
C83—C84	1.53 (2)	C86'—H86A	0.9900
C84—H84D	0.9800	C86'—H86B	0.9900
C84—H84E	0.9800	C86'—C87'	1.507 (16)
C84—H84F	0.9800	C87'—H87A	0.9900
C85—H85C	0.9900	C87'—H87B	0.9900
C85—H85D	0.9900	C87'—C88'	1.506 (18)
C85—C86	1.506 (10)	C88'—H88A	0.9900
C86—H86C	0.9900	C88'—H88B	0.9900
C86—H86D	0.9900	C88'—C89'	1.513 (18)
C86—C87	1.515 (10)	C89'—H89A	0.9900
C87—H87C	0.9900	C89'—H89B	0.9900
C87—H87D	0.9900	C89'—C90'	1.504 (19)
C87—C88	1.529 (11)	C90'—H90A	0.9800
C88—H88C	0.9900	C90'—H90B	0.9800
C88—H88D	0.9900	C90'—H90C	0.9800
C88—C89	1.520 (11)	C91'—H91A	0.9900
C89—H89C	0.9900	C91'—H91B	0.9900
C89—H89D	0.9900	C91'—C92'	1.51 (2)
C89—C90	1.527 (11)	C92'—H92A	0.9900
C90—H90D	0.9800	C92'—H92B	0.9900
C90—H90E	0.9800	C92'—C93'	1.41 (2)
C90—H90F	0.9800	C93'—H93A	0.9900
C91—H91C	0.9900	C93'—H93B	0.9900
C91—H91D	0.9900	C93'—C94'	1.49 (3)
C91—C92	1.516 (14)	C94'—H94A	0.9900
C92—H92C	0.9900	C94'—H94B	0.9900
C92—H92D	0.9900	C94'—C95'	1.62 (3)
C92—C93	1.541 (14)	C95'—H95A	0.9900
C93—H93C	0.9900	C95'—H95B	0.9900
C93—H93D	0.9900	C95'—C96'	1.53 (3)
C93—C94	1.478 (15)	C96'—H96A	0.9800
C94—H94C	0.9900	C96'—H96B	0.9800
C94—H94D	0.9900	C96'—H96C	0.9800
C94—C95	1.525 (18)	C97'—H97A	0.9900
C95—H95C	0.9900	C97'—H97B	0.9900
C95—H95D	0.9900	C97'—C98'	1.510 (18)
C95—C96	1.53 (2)	C98'—H98A	0.9900
C96—H96D	0.9800	C98'—H98B	0.9900
C96—H96E	0.9800	C98'—C99'	1.531 (18)
C96—H96F	0.9800	C99'—H99A	0.9900
C97—H97C	0.9900	C99'—H99B	0.9900
C97—H97D	0.9900	C99'—C200	1.51 (2)
C97—C98	1.526 (11)	C200—H20A	0.9900
C98—H98C	0.9900	C200—H20B	0.9900
C98—H98D	0.9900	C200—C201	1.52 (2)
C98—C99	1.534 (11)	C201—H20C	0.9900
C99—H99C	0.9900	C201—H20D	0.9900
C99—H99D	0.9900	C201—C202	1.65 (3)
C99—C100	1.478 (12)	C202—H20E	0.9800
C100—H10C	0.9900	C202—H20F	0.9800
C100—H10D	0.9900	C202—H20G	0.9800
C100—H10A	0.9900	C203—H20H	0.9900
C100—H10B	0.9900	C203—H20I	0.9900
C100—C101	1.526 (17)	C203—H20J	0.9900
C100—C126	1.526 (12)	C203—H20K	0.9900
C103—H10E	0.9900	C203—C204	1.543 (12)
C103—H10F	0.9900	C203—C20B	1.520 (19)
C103—C104	1.515 (11)	C206—H20N	0.9900
C104—H10G	0.9900	C206—H20O	0.9900
C104—H10H	0.9900	C206—H20L	0.9900
C104—H10I	0.9900	C206—H20M	0.9900
C104—H10J	0.9900	C206—C207	1.519 (14)
C104—C105	1.526 (11)	C206—C205	1.529 (12)
C104—C10A	1.534 (19)	C206—C20C	1.550 (13)
C109—H10K	0.9900	C207—H20P	0.9900
C109—H10L	0.9900	C207—H20Q	0.9900
C109—C110	1.517 (11)	C207—C208	1.543 (14)
C110—H11A	0.9900	C208—H20R	0.9800
C110—H11B	0.9900	C208—H20S	0.9800
C110—C111	1.537 (12)	C208—H20T	0.9800
C111—H11C	0.9900	C209—H20U	0.9900
C111—H11D	0.9900	C209—H20V	0.9900
C111—C112	1.534 (11)	C209—C210	1.511 (17)
C112—H11E	0.9900	C210—H21A	0.9900
C112—H11F	0.9900	C210—H21B	0.9900
C112—C113	1.539 (11)	C210—C211	1.503 (18)
C113—H11G	0.9900	C211—H21C	0.9900
C113—H11H	0.9900	C211—H21D	0.9900
C113—C114	1.545 (12)	C211—H21E	0.9900
C114—H11I	0.9800	C211—H21F	0.9900
C114—H11J	0.9800	C211—C21B	1.538 (18)
C114—H11K	0.9800	C211—C212	1.521 (18)
C115—H11L	0.9900	C215—H21G	0.9900
C115—H11M	0.9900	C215—H21H	0.9900
C115—C116	1.508 (11)	C215—C216	1.50 (2)
C116—H11N	0.9900	C216—H21I	0.9900
C116—H11O	0.9900	C216—H21J	0.9900
C116—C117	1.507 (11)	C216—H21K	0.9900
C117—H11P	0.9900	C216—H21L	0.9900
C117—H11Q	0.9900	C216—C217	1.525 (14)
C117—C118	1.514 (11)	C216—C21E	1.529 (14)
C118—H11R	0.9900	C221—H22A	0.9800
C118—H11S	0.9900	C221—H22B	0.9800
C118—C119	1.514 (11)	C221—H22C	0.9800
C119—H11T	0.9900	C222—H22D	0.9800
C119—H11U	0.9900	C222—H22E	0.9800
C119—C120	1.507 (11)	C222—H22F	0.9800
C120—H12A	0.9800	C223—H22G	0.9800
C120—H12B	0.9800	C223—H22H	0.9800
C120—H12C	0.9800	C223—H22I	0.9800
C121—H12D	0.9800	C224—H22J	0.9800
C121—H12E	0.9800	C224—H22K	0.9800
C121—H12F	0.9800	C224—H22L	0.9800
C122—H12G	0.9800	C21B—H21M	0.9900
C122—H12H	0.9800	C21B—H21N	0.9900
C122—H12I	0.9800	C21B—C21C	1.502 (19)
C123—H12J	0.9800	C21C—H21O	0.9900
C123—H12K	0.9800	C21C—H21P	0.9900
C123—H12L	0.9800	C21C—C21D	1.530 (19)
C124—H12M	0.9800	C21D—H21Q	0.9800
C124—H12N	0.9800	C21D—H21R	0.9800
C124—H12O	0.9800	C21D—H21S	0.9800
C101—H10M	0.9900	C80'—H80A	0.9900
C101—H10N	0.9900	C80'—H80B	0.9900
C101—C102	1.505 (19)	C204—H20W	0.9900
C102—H10O	0.9800	C204—H20X	0.9900
C102—H10P	0.9800	C204—C205	1.533 (12)
C102—H10Q	0.9800	C205—H20Y	0.9900
C105—H10R	0.9900	C205—HF	0.9900
C105—H10S	0.9900	C217—H21T	0.9900
C105—C106	1.526 (11)	C217—H21U	0.9900
C106—H10T	0.9900	C217—C218	1.536 (14)
C106—H10U	0.9900	C218—H21V	0.9900
C106—C107	1.531 (16)	C218—H21W	0.9900
C107—H10V	0.9900	C218—C219	1.541 (19)
C107—H10W	0.9900	C219—H21X	0.9900
C107—C108	1.536 (18)	C219—H21Y	0.9900
C108—H10X	0.9800	C219—C220	1.513 (19)
C108—H10Y	0.9800	C220—H22M	0.9800
C108—H	0.9800	C220—H22N	0.9800
C10A—H10Z	0.9900	C220—H22O	0.9800
C10A—HA	0.9900	C20B—H20Z	0.9900
C10A—C10B	1.52 (2)	C20B—HG	0.9900
C10B—H10	0.9900	C20B—C20C	1.55 (2)
C10B—HB	0.9900	C20C—H20	0.9900
C10B—C10C	1.54 (2)	C20C—HH	0.9900
C10C—H7	0.9900	C21E—H21Z	0.9900
C10C—HC	0.9900	C21E—HI	0.9900
C10C—C10D	1.530 (12)	C21E—C21F	1.574 (19)
C10D—H12	0.9800	C21F—H21	0.9900
C10D—HD	0.9800	C21F—HJ	0.9900
C10D—HE	0.9800	C21F—C21G	1.523 (14)
C125—H12P	0.9800	C21G—H1	0.9900
C125—H12Q	0.9800	C21G—HK	0.9900
C125—H12R	0.9800	C21G—C21H	1.528 (14)
C125—C126	1.511 (14)	C21H—H2	0.9800
C126—H12S	0.9900	C21H—HL	0.9800
C126—H12T	0.9900	C21H—HM	0.9800
Ir3—N01'	2.243 (10)	C80B—H80C	0.9900
Ir3—N1'	2.043 (11)	C80B—H80D	0.9900
Ir3—N2'	1.943 (13)	C212—H3	0.9900
Ir3—N02'	2.257 (9)	C212—HN	0.9900
Ir3—C1'	2.013 (12)	C212—C213	1.496 (19)
Ir3—C19'	2.002 (12)	C213—H4	0.9900
Ir4—N01'	2.289 (10)	C213—HO	0.9900
Ir4—N02'	2.192 (10)	C213—C214	1.528 (19)
Ir4—N3'	2.067 (11)	C214—H5	0.9800
Ir4—N4'	1.899 (12)	C214—HP	0.9800
Ir4—C37'	1.990 (12)	C214—HQ	0.9800
			
N1—Ir1—N01	93.1 (3)	C37'—Ir4—N02'	94.1 (4)
N1—Ir1—N02	95.8 (3)	C37'—Ir4—N3'	80.8 (5)
N1—Ir1—N2	171.0 (3)	C55'—Ir4—N01'	91.2 (4)
N02—Ir1—N01	80.4 (3)	C55'—Ir4—N02'	168.2 (4)
N2—Ir1—N01	94.2 (3)	C55'—Ir4—N3'	92.8 (5)
N2—Ir1—N02	90.7 (3)	C55'—Ir4—C37'	95.2 (5)
C1—Ir1—N1	81.0 (4)	C10'—O3'—C221	112.8 (13)
C1—Ir1—N01	172.1 (3)	C28'—O4'—C222	114.0 (15)
C1—Ir1—N02	94.8 (3)	C46'—O5'—C223	114.9 (15)
C1—Ir1—N2	92.2 (4)	C64'—O6'—C224	119.7 (14)
C1—Ir1—C19	95.8 (4)	Ir3—N01'—Ir4	98.4 (4)
C19—Ir1—N1	93.8 (4)	C01'—N01'—Ir3	132.2 (12)
C19—Ir1—N01	89.9 (4)	C01'—N01'—Ir4	128.7 (12)
C19—Ir1—N02	166.7 (4)	C7'—N1'—Ir3	113.9 (8)
C19—Ir1—N2	80.9 (4)	C11'—N1'—Ir3	125.2 (10)
N02—Ir2—N01	80.2 (3)	C11'—N1'—C7'	120.9 (12)
N3—Ir2—N01	93.0 (3)	C25'—N2'—Ir3	118.4 (10)
N3—Ir2—N02	93.8 (3)	C25'—N2'—C29'	113.4 (14)
N4—Ir2—N01	90.0 (3)	C29'—N2'—Ir3	127.0 (11)
N4—Ir2—N02	92.7 (3)	Ir4—N02'—Ir3	100.9 (4)
N4—Ir2—N3	173.2 (3)	C02'—N02'—Ir3	125.9 (10)
C37—Ir2—N01	92.7 (4)	C02'—N02'—Ir4	132.1 (10)
C37—Ir2—N02	171.1 (4)	C43'—N3'—Ir4	115.2 (9)
C37—Ir2—N3	81.2 (4)	C43'—N3'—C47'	120.1 (13)
C37—Ir2—N4	92.6 (4)	C47'—N3'—Ir4	124.1 (10)
C37—Ir2—C55	97.5 (4)	C61'—N4'—Ir4	121.8 (9)
C55—Ir2—N01	166.4 (4)	C61'—N4'—C65'	110.1 (12)
C55—Ir2—N02	90.4 (4)	C65'—N4'—Ir4	127.9 (10)
C55—Ir2—N3	97.5 (4)	N01'—C01'—O1'	179 (2)
C55—Ir2—N4	80.5 (4)	C2'—C1'—Ir3	129.9 (10)
C10—O3—C121	116.9 (10)	C2'—C1'—C6'	117.7 (11)
C28—O4—C122	116.0 (9)	C6'—C1'—Ir3	112.0 (9)
C46—O5—C123	116.9 (10)	N02'—C02'—O2'	178 (2)
C64—O6—C124	117.2 (9)	C1'—C2'—H2'	119.2
C7—N1—Ir1	115.4 (7)	C3'—C2'—C1'	121.7 (13)
C11—N1—Ir1	125.4 (8)	C3'—C2'—H2'	119.2
C11—N1—C7	119.0 (9)	C2'—C3'—C4'	120.5 (13)
Ir1—N01—Ir2	98.8 (4)	C2'—C3'—C12'	130.3 (13)
C01—N01—Ir1	132.2 (8)	C4'—C3'—C12'	109.1 (11)
C01—N01—Ir2	128.6 (8)	C3'—C4'—C18'	111.1 (12)
Ir2—N02—Ir1	100.6 (3)	C5'—C4'—C3'	121.1 (12)
C02—N02—Ir1	128.4 (9)	C5'—C4'—C18'	127.6 (13)
C02—N02—Ir2	129.3 (9)	C4'—C5'—H5'	121.0
C25—N2—Ir1	116.3 (7)	C4'—C5'—C6'	118.1 (13)
C25—N2—C29	119.9 (9)	C6'—C5'—H5'	121.0
C29—N2—Ir1	123.6 (7)	C1'—C6'—C5'	120.7 (12)
C43—N3—Ir2	114.9 (7)	C1'—C6'—C7'	116.3 (12)
C43—N3—C47	122.4 (10)	C5'—C6'—C7'	122.7 (13)
C47—N3—Ir2	122.7 (8)	N1'—C7'—C6'	115.2 (12)
C61—N4—Ir2	116.0 (7)	N1'—C7'—C8'	119.2 (12)
C65—N4—Ir2	125.4 (8)	C8'—C7'—C6'	125.5 (13)
C65—N4—C61	118.5 (10)	C7'—C8'—H8'	119.5
N01—C01—O1	179.6 (17)	C9'—C8'—C7'	121.0 (14)
C2—C1—Ir1	128.8 (8)	C9'—C8'—H8'	119.5
C6—C1—Ir1	114.5 (7)	C8'—C9'—H9'	120.7
C6—C1—C2	116.7 (10)	C8'—C9'—C10'	118.5 (15)
N02—C02—O2	175.3 (19)	C10'—C9'—H9'	120.7
C1—C2—H2A	119.6	O3'—C10'—C9'	114.6 (15)
C3—C2—C1	120.7 (11)	C11'—C10'—O3'	126.6 (14)
C3—C2—H2A	119.6	C11'—C10'—C9'	118.8 (14)
C2—C3—C4	121.5 (11)	N1'—C11'—C10'	121.5 (14)
C2—C3—C12	129.8 (11)	N1'—C11'—H11'	119.2
C4—C3—C12	108.7 (10)	C10'—C11'—H11'	119.2
C3—C4—C5	120.6 (11)	C13'—C12'—C3'	131.9 (11)
C3—C4—C18	111.4 (10)	C13'—C12'—C17'	119.8 (12)
C5—C4—C18	128.0 (11)	C17'—C12'—C3'	108.2 (12)
C4—C5—H5A	121.0	C12'—C13'—H13'	120.5
C4—C5—C6	117.9 (11)	C12'—C13'—C14'	119.0 (12)
C6—C5—H5A	121.0	C14'—C13'—H13'	120.5
C1—C6—C7	115.4 (10)	C13'—C14'—H14'	119.7
C5—C6—C1	122.6 (10)	C15'—C14'—C13'	120.6 (14)
C5—C6—C7	122.0 (10)	C15'—C14'—H14'	119.7
N1—C7—C6	113.2 (10)	C14'—C15'—H15'	119.8
N1—C7—C8	118.9 (10)	C16'—C15'—C14'	120.4 (13)
C8—C7—C6	127.9 (11)	C16'—C15'—H15'	119.8
C7—C8—H8	118.7	C15'—C16'—H16'	119.7
C9—C8—C7	122.5 (12)	C15'—C16'—C17'	120.6 (13)
C9—C8—H8	118.7	C17'—C16'—H16'	119.7
C8—C9—H9	120.6	C12'—C17'—C18'	110.7 (11)
C8—C9—C10	118.8 (12)	C16'—C17'—C12'	119.0 (13)
C10—C9—H9	120.6	C16'—C17'—C18'	129.9 (12)
O3—C10—C9	118.5 (11)	C4'—C18'—C73'	111.5 (12)
O3—C10—C11	122.9 (12)	C17'—C18'—C4'	100.8 (10)
C11—C10—C9	118.6 (11)	C17'—C18'—C73'	111.6 (12)
N1—C11—C10	122.1 (11)	C79'—C18'—C4'	112.8 (13)
N1—C11—H11	118.9	C79'—C18'—C17'	111.9 (13)
C10—C11—H11	118.9	C79'—C18'—C73'	108.2 (12)
C13—C12—C3	130.5 (11)	C20'—C19'—Ir3	127.9 (10)
C13—C12—C17	121.5 (11)	C20'—C19'—C24'	119.3 (12)
C17—C12—C3	108.0 (11)	C24'—C19'—Ir3	112.5 (9)
C12—C13—H13	120.0	C19'—C20'—H20'	121.2
C12—C13—C14	120.0 (12)	C19'—C20'—C21'	117.5 (13)
C14—C13—H13	120.0	C21'—C20'—H20'	121.2
C13—C14—H14	120.3	C20'—C21'—C30'	126.4 (14)
C13—C14—C15	119.3 (13)	C22'—C21'—C20'	124.4 (14)
C15—C14—H14	120.3	C22'—C21'—C30'	109.1 (13)
C14—C15—H15	120.2	C21'—C22'—C23'	117.5 (13)
C16—C15—C14	119.6 (12)	C21'—C22'—C36'	111.8 (13)
C16—C15—H15	120.2	C23'—C22'—C36'	130.7 (14)
C15—C16—H16	119.4	C22'—C23'—H23'	119.9
C15—C16—C17	121.1 (12)	C24'—C23'—C22'	120.2 (14)
C17—C16—H16	119.4	C24'—C23'—H23'	119.9
C12—C17—C18	111.6 (10)	C19'—C24'—C25'	115.6 (13)
C16—C17—C12	118.4 (12)	C23'—C24'—C19'	121.0 (13)
C16—C17—C18	130.0 (11)	C23'—C24'—C25'	123.5 (15)
C17—C18—C4	100.2 (9)	N2'—C25'—C24'	111.7 (14)
C73—C18—C4	112.6 (9)	N2'—C25'—C26'	124.3 (14)
C73—C18—C17	110.8 (9)	C26'—C25'—C24'	124.0 (15)
C79—C18—C4	111.7 (9)	C25'—C26'—H26'	120.3
C79—C18—C17	112.1 (10)	C25'—C26'—C27'	119.3 (16)
C79—C18—C73	109.2 (10)	C27'—C26'—H26'	120.3
C20—C19—Ir1	130.1 (8)	C26'—C27'—H27'	122.2
C20—C19—C24	115.7 (9)	C28'—C27'—C26'	115.6 (16)
C24—C19—Ir1	113.9 (8)	C28'—C27'—H27'	122.2
C19—C20—H6	119.1	C27'—C28'—O4'	112.0 (17)
C21—C20—C19	121.9 (11)	C29'—C28'—O4'	126.1 (17)
C21—C20—H6	119.1	C29'—C28'—C27'	121.9 (16)
C20—C21—C22	121.3 (10)	N2'—C29'—H29'	117.3
C20—C21—C30	129.3 (11)	C28'—C29'—N2'	125.3 (16)
C22—C21—C30	109.4 (9)	C28'—C29'—H29'	117.3
C21—C22—C36	110.8 (10)	C31'—C30'—C21'	134.6 (14)
C23—C22—C21	120.1 (10)	C31'—C30'—C35'	118.4 (13)
C23—C22—C36	129.1 (10)	C35'—C30'—C21'	106.7 (13)
C22—C23—H23	120.8	C30'—C31'—H31'	119.9
C22—C23—C24	118.4 (10)	C32'—C31'—C30'	120.1 (14)
C24—C23—H23	120.8	C32'—C31'—H31'	119.9
C19—C24—C25	115.3 (9)	C31'—C32'—H32'	119.8
C23—C24—C19	122.6 (10)	C33'—C32'—C31'	120.4 (15)
C23—C24—C25	121.9 (10)	C33'—C32'—H32'	119.8
N2—C25—C24	112.8 (10)	C32'—C33'—H33'	119.1
N2—C25—C26	118.7 (10)	C32'—C33'—C34'	121.9 (15)
C26—C25—C24	128.4 (10)	C34'—C33'—H33'	119.1
C25—C26—H26	119.1	C33'—C34'—H34'	119.9
C27—C26—C25	121.7 (11)	C33'—C34'—C35'	120.2 (14)
C27—C26—H26	119.1	C35'—C34'—H34'	119.9
C26—C27—H27	120.6	C30'—C35'—C36'	110.1 (13)
C26—C27—C28	118.9 (11)	C34'—C35'—C30'	118.6 (14)
C28—C27—H27	120.6	C34'—C35'—C36'	131.3 (14)
O4—C28—C27	117.3 (11)	C22'—C36'—C91'	111.1 (12)
O4—C28—C29	124.1 (11)	C35'—C36'—C22'	102.2 (12)
C29—C28—C27	118.6 (11)	C35'—C36'—C85'	112.7 (12)
N2—C29—H29	119.0	C35'—C36'—C91'	110.1 (12)
C28—C29—N2	121.9 (10)	C85'—C36'—C22'	111.0 (11)
C28—C29—H29	119.0	C85'—C36'—C91'	109.6 (12)
C31—C30—C21	131.3 (10)	C38'—C37'—Ir4	128.3 (10)
C31—C30—C35	123.1 (10)	C42'—C37'—Ir4	112.9 (9)
C35—C30—C21	105.6 (10)	C42'—C37'—C38'	118.7 (12)
C30—C31—H31	121.3	C37'—C38'—H38'	121.0
C30—C31—C32	117.4 (10)	C39'—C38'—C37'	118.1 (13)
C32—C31—H31	121.3	C39'—C38'—H38'	121.0
C31—C32—H32	119.4	C38'—C39'—C48'	129.3 (12)
C33—C32—C31	121.2 (11)	C40'—C39'—C38'	123.3 (12)
C33—C32—H32	119.4	C40'—C39'—C48'	107.4 (11)
C32—C33—H33	119.4	C39'—C40'—C54'	112.0 (12)
C32—C33—C34	121.1 (11)	C41'—C40'—C39'	120.2 (12)
C34—C33—H33	119.4	C41'—C40'—C54'	127.7 (13)
C33—C34—H34	120.0	C40'—C41'—H41'	120.4
C33—C34—C35	120.1 (11)	C40'—C41'—C42'	119.2 (13)
C35—C34—H34	120.0	C42'—C41'—H41'	120.4
C30—C35—C36	113.8 (10)	C37'—C42'—C41'	120.4 (12)
C34—C35—C30	117.1 (11)	C37'—C42'—C43'	116.1 (12)
C34—C35—C36	129.1 (10)	C41'—C42'—C43'	123.5 (13)
C35—C36—C22	100.4 (9)	N3'—C43'—C42'	114.5 (13)
C35—C36—C85	111.9 (9)	N3'—C43'—C44'	120.2 (13)
C35—C36—C91	111.9 (9)	C44'—C43'—C42'	125.2 (14)
C85—C36—C22	111.4 (8)	C43'—C44'—H44'	120.7
C91—C36—C22	110.1 (9)	C45'—C44'—C43'	118.5 (16)
C91—C36—C85	110.7 (9)	C45'—C44'—H44'	120.7
C38—C37—Ir2	129.7 (9)	C44'—C45'—H45'	120.6
C38—C37—C42	115.6 (10)	C46'—C45'—C44'	118.8 (17)
C42—C37—Ir2	114.4 (8)	C46'—C45'—H45'	120.6
C37—C38—H38	118.8	O5'—C46'—C45'	113.4 (17)
C37—C38—C39	122.5 (11)	C47'—C46'—O5'	126.1 (17)
C39—C38—H38	118.8	C47'—C46'—C45'	120.5 (15)
C38—C39—C48	131.8 (11)	N3'—C47'—H47'	119.1
C40—C39—C38	119.6 (11)	C46'—C47'—N3'	121.9 (16)
C40—C39—C48	108.6 (10)	C46'—C47'—H47'	119.1
C39—C40—C54	110.2 (10)	C49'—C48'—C39'	129.7 (12)
C41—C40—C39	120.9 (11)	C53'—C48'—C39'	109.8 (12)
C41—C40—C54	128.9 (11)	C53'—C48'—C49'	120.4 (12)
C40—C41—H41	121.0	C48'—C49'—H49'	121.1
C40—C41—C42	118.1 (11)	C48'—C49'—C50'	117.7 (12)
C42—C41—H41	121.0	C50'—C49'—H49'	121.1
C37—C42—C43	114.6 (10)	C49'—C50'—H50'	120.2
C41—C42—C37	123.1 (10)	C51'—C50'—C49'	119.7 (13)
C41—C42—C43	122.3 (11)	C51'—C50'—H50'	120.2
N3—C43—C42	114.8 (10)	C50'—C51'—H51'	118.2
N3—C43—C44	118.8 (11)	C50'—C51'—C52'	123.5 (12)
C44—C43—C42	126.4 (11)	C52'—C51'—H51'	118.2
C43—C44—H44	119.3	C51'—C52'—H52'	121.6
C45—C44—C43	121.3 (12)	C53'—C52'—C51'	116.9 (13)
C45—C44—H44	119.3	C53'—C52'—H52'	121.6
C44—C45—H45	120.9	C48'—C53'—C52'	121.8 (13)
C44—C45—C46	118.1 (12)	C48'—C53'—C54'	110.2 (11)
C46—C45—H45	120.9	C52'—C53'—C54'	127.9 (13)
O5—C46—C45	116.1 (11)	C40'—C54'—C53'	100.4 (11)
O5—C46—C47	123.3 (12)	C40'—C54'—C97'	111.2 (12)
C47—C46—C45	120.6 (11)	C40'—C54'—C203	111.6 (11)
N3—C47—C46	118.8 (11)	C53'—C54'—C97'	109.5 (11)
N3—C47—H47	120.6	C53'—C54'—C203	111.7 (12)
C46—C47—H47	120.6	C203—C54'—C97'	111.8 (11)
C49—C48—C39	130.0 (12)	C56'—C55'—Ir4	129.6 (10)
C49—C48—C53	120.6 (11)	C56'—C55'—C60'	117.7 (12)
C53—C48—C39	109.4 (11)	C60'—C55'—Ir4	112.6 (9)
C48—C49—H49	120.6	C55'—C56'—H56'	119.9
C48—C49—C50	118.8 (12)	C57'—C56'—C55'	120.2 (12)
C50—C49—H49	120.6	C57'—C56'—H56'	119.9
C49—C50—H50	120.3	C56'—C57'—C58'	122.0 (12)
C51—C50—C49	119.3 (13)	C56'—C57'—C66'	129.9 (12)
C51—C50—H50	120.3	C58'—C57'—C66'	108.2 (11)
C50—C51—H51	119.5	C57'—C58'—C72'	110.9 (11)
C52—C51—C50	121.0 (13)	C59'—C58'—C57'	119.1 (12)
C52—C51—H51	119.5	C59'—C58'—C72'	130.0 (12)
C51—C52—H52	119.9	C58'—C59'—H59'	120.3
C53—C52—C51	120.2 (14)	C58'—C59'—C60'	119.4 (12)
C53—C52—H52	119.9	C60'—C59'—H59'	120.3
C48—C53—C54	110.6 (10)	C55'—C60'—C61'	115.1 (12)
C52—C53—C48	120.1 (13)	C59'—C60'—C55'	121.4 (12)
C52—C53—C54	129.3 (12)	C59'—C60'—C61'	123.4 (13)
C40—C54—C97	110.8 (10)	N4'—C61'—C60'	108.5 (12)
C40—C54—C103	111.5 (10)	N4'—C61'—C62'	125.3 (13)
C53—C54—C40	101.0 (9)	C62'—C61'—C60'	126.2 (14)
C53—C54—C97	112.2 (10)	C61'—C62'—H62'	120.4
C53—C54—C103	113.0 (10)	C61'—C62'—C63'	119.3 (15)
C97—C54—C103	108.2 (10)	C63'—C62'—H62'	120.4
C56—C55—Ir2	127.3 (8)	C62'—C63'—H63'	120.2
C60—C55—Ir2	115.4 (8)	C64'—C63'—C62'	119.6 (16)
C60—C55—C56	117.0 (10)	C64'—C63'—H63'	120.2
C55—C56—H56	120.6	C63'—C64'—O6'	118.6 (16)
C57—C56—C55	118.9 (11)	C63'—C64'—C65'	119.6 (16)
C57—C56—H56	120.6	C65'—C64'—O6'	121.6 (15)
C56—C57—C66	129.2 (11)	N4'—C65'—H65'	117.1
C58—C57—C56	122.4 (11)	C64'—C65'—N4'	125.7 (15)
C58—C57—C66	108.4 (10)	C64'—C65'—H65'	117.1
C57—C58—C72	111.5 (10)	C67'—C66'—C57'	131.0 (12)
C59—C58—C57	119.8 (11)	C67'—C66'—C71'	119.6 (12)
C59—C58—C72	128.7 (11)	C71'—C66'—C57'	109.4 (12)
C58—C59—H59	120.8	C66'—C67'—H67'	120.4
C58—C59—C60	118.5 (10)	C68'—C67'—C66'	119.1 (13)
C60—C59—H59	120.8	C68'—C67'—H67'	120.4
C55—C60—C59	123.3 (10)	C67'—C68'—H68'	119.4
C55—C60—C61	113.2 (9)	C67'—C68'—C69'	121.2 (14)
C59—C60—C61	123.5 (10)	C69'—C68'—H68'	119.4
N4—C61—C60	114.2 (10)	C68'—C69'—H69'	119.7
N4—C61—C62	120.2 (10)	C68'—C69'—C70'	120.7 (13)
C62—C61—C60	125.4 (10)	C70'—C69'—H69'	119.7
C61—C62—H62	119.6	C69'—C70'—H70'	120.8
C63—C62—C61	120.9 (11)	C71'—C70'—C69'	118.5 (14)
C63—C62—H62	119.6	C71'—C70'—H70'	120.8
C62—C63—H63	121.1	C66'—C71'—C72'	109.6 (11)
C62—C63—C64	117.8 (12)	C70'—C71'—C66'	120.9 (14)
C64—C63—H63	121.1	C70'—C71'—C72'	129.5 (13)
O6—C64—C63	117.4 (11)	C58'—C72'—C209	110.9 (11)
O6—C64—C65	123.4 (11)	C58'—C72'—C215	111.5 (11)
C65—C64—C63	119.2 (11)	C71'—C72'—C58'	101.8 (11)
N4—C65—C64	123.3 (11)	C71'—C72'—C209	110.7 (11)
N4—C65—H65	118.3	C71'—C72'—C215	112.1 (11)
C64—C65—H65	118.3	C215—C72'—C209	109.6 (12)
C67—C66—C57	131.5 (11)	C18'—C73'—H73A	108.4
C67—C66—C71	121.0 (10)	C18'—C73'—H73B	108.4
C71—C66—C57	107.5 (10)	H73A—C73'—H73B	107.5
C66—C67—H67	120.3	C74'—C73'—C18'	115.3 (12)
C68—C67—C66	119.3 (12)	C74'—C73'—H73A	108.4
C68—C67—H67	120.3	C74'—C73'—H73B	108.4
C67—C68—H68	120.0	C73'—C74'—H74A	107.6
C67—C68—C69	120.1 (12)	C73'—C74'—H74B	107.6
C69—C68—H68	120.0	C73'—C74'—C75'	118.8 (14)
C68—C69—H69	120.2	H74A—C74'—H74B	107.1
C70—C69—C68	119.6 (12)	C75'—C74'—H74A	107.6
C70—C69—H69	120.2	C75'—C74'—H74B	107.6
C69—C70—H70	119.1	C74'—C75'—H75A	108.5
C69—C70—C71	121.7 (12)	C74'—C75'—H75B	108.5
C71—C70—H70	119.1	H75A—C75'—H75B	107.5
C66—C71—C72	112.1 (10)	C76'—C75'—C74'	114.9 (14)
C70—C71—C66	118.2 (11)	C76'—C75'—H75A	108.5
C70—C71—C72	129.7 (11)	C76'—C75'—H75B	108.5
C58—C72—C109	110.0 (9)	C75'—C76'—H76A	108.9
C58—C72—C115	113.3 (10)	C75'—C76'—H76B	108.9
C71—C72—C58	100.4 (9)	C75'—C76'—C77'	113.4 (16)
C71—C72—C109	112.5 (10)	H76A—C76'—H76B	107.7
C71—C72—C115	113.1 (9)	C77'—C76'—H76A	108.9
C115—C72—C109	107.5 (9)	C77'—C76'—H76B	108.9
C18—C73—H73C	108.3	C76'—C77'—H77A	109.6
C18—C73—H73D	108.3	C76'—C77'—H77B	109.6
H73C—C73—H73D	107.4	H77A—C77'—H77B	108.1
C74—C73—C18	115.9 (10)	C78'—C77'—C76'	110.4 (16)
C74—C73—H73C	108.3	C78'—C77'—H77A	109.6
C74—C73—H73D	108.3	C78'—C77'—H77B	109.6
C73—C74—H74C	108.7	C77'—C78'—H78A	109.5
C73—C74—H74D	108.7	C77'—C78'—H78B	109.5
C73—C74—C75	114.1 (11)	C77'—C78'—H78C	109.5
H74C—C74—H74D	107.6	H78A—C78'—H78B	109.5
C75—C74—H74C	108.7	H78A—C78'—H78C	109.5
C75—C74—H74D	108.7	H78B—C78'—H78C	109.5
C74—C75—H75C	107.7	C18'—C79'—H79C	106.6
C74—C75—H75D	107.7	C18'—C79'—H79D	106.6
H75C—C75—H75D	107.1	C18'—C79'—H79A	109.1
C76—C75—C74	118.5 (13)	C18'—C79'—H79B	109.1
C76—C75—H75C	107.7	C18'—C79'—C80'	112.3 (15)
C76—C75—H75D	107.7	C18'—C79'—C80B	123.1 (19)
C75—C76—H76C	109.7	H79C—C79'—H79D	106.5
C75—C76—H76D	109.7	H79A—C79'—H79B	107.9
C75—C76—C77	110.0 (16)	C80'—C79'—H79A	109.1
H76C—C76—H76D	108.2	C80'—C79'—H79B	109.1
C77—C76—H76C	109.7	C80B—C79'—H79C	106.6
C77—C76—H76D	109.7	C80B—C79'—H79D	106.6
C76—C77—H77C	109.0	H81C—C81'—H81D	104.3
C76—C77—H77D	109.0	H81A—C81'—H81B	106.9
H77C—C77—H77D	107.8	C82'—C81'—H81C	100.6
C78—C77—C76	113.0 (18)	C82'—C81'—H81D	100.6
C78—C77—H77C	109.0	C82'—C81'—H81A	107.4
C78—C77—H77D	109.0	C82'—C81'—H81B	107.4
C77—C78—H78D	109.5	C80'—C81'—H81A	107.4
C77—C78—H78E	109.5	C80'—C81'—H81B	107.4
C77—C78—H78F	109.5	C80'—C81'—C82'	120 (2)
H78D—C78—H78E	109.5	C80B—C81'—H81C	100.6
H78D—C78—H78F	109.5	C80B—C81'—H81D	100.6
H78E—C78—H78F	109.5	C80B—C81'—C82'	145 (3)
C18—C79—H79E	108.3	C81'—C82'—H82A	106.0
C18—C79—H79F	108.3	C81'—C82'—H82B	106.0
C18—C79—C80	115.8 (10)	H82A—C82'—H82B	106.3
H79E—C79—H79F	107.4	C83'—C82'—C81'	125 (2)
C80—C79—H79E	108.3	C83'—C82'—H82A	106.0
C80—C79—H79F	108.3	C83'—C82'—H82B	106.0
C79—C80—H80E	108.8	C82'—C83'—H83A	111.8
C79—C80—H80F	108.8	C82'—C83'—H83B	111.8
H80E—C80—H80F	107.7	H83A—C83'—H83B	109.5
C81—C80—C79	113.8 (11)	C84'—C83'—C82'	100 (2)
C81—C80—H80E	108.8	C84'—C83'—H83A	111.8
C81—C80—H80F	108.8	C84'—C83'—H83B	111.8
C80—C81—H81E	108.5	C83'—C84'—H84A	109.5
C80—C81—H81F	108.5	C83'—C84'—H84B	109.5
H81E—C81—H81F	107.5	C83'—C84'—H84C	109.5
C82—C81—C80	115.0 (12)	H84A—C84'—H84B	109.5
C82—C81—H81E	108.5	H84A—C84'—H84C	109.5
C82—C81—H81F	108.5	H84B—C84'—H84C	109.5
C81—C82—H82C	108.7	C36'—C85'—H85A	108.3
C81—C82—H82D	108.7	C36'—C85'—H85B	108.3
H82C—C82—H82D	107.6	C36'—C85'—C86'	115.9 (12)
C83—C82—C81	114.4 (13)	H85A—C85'—H85B	107.4
C83—C82—H82C	108.7	C86'—C85'—H85A	108.3
C83—C82—H82D	108.7	C86'—C85'—H85B	108.3
C82—C83—H83C	108.6	C85'—C86'—H86A	108.2
C82—C83—H83D	108.6	C85'—C86'—H86B	108.2
C82—C83—C84	114.8 (15)	H86A—C86'—H86B	107.4
H83C—C83—H83D	107.6	C87'—C86'—C85'	116.3 (13)
C84—C83—H83C	108.6	C87'—C86'—H86A	108.2
C84—C83—H83D	108.6	C87'—C86'—H86B	108.2
C83—C84—H84D	109.5	C86'—C87'—H87A	108.5
C83—C84—H84E	109.5	C86'—C87'—H87B	108.5
C83—C84—H84F	109.5	H87A—C87'—H87B	107.5
H84D—C84—H84E	109.5	C88'—C87'—C86'	115.1 (13)
H84D—C84—H84F	109.5	C88'—C87'—H87A	108.5
H84E—C84—H84F	109.5	C88'—C87'—H87B	108.5
C36—C85—H85C	108.3	C87'—C88'—H88A	108.4
C36—C85—H85D	108.3	C87'—C88'—H88B	108.4
H85C—C85—H85D	107.4	C87'—C88'—C89'	115.6 (14)
C86—C85—C36	116.1 (9)	H88A—C88'—H88B	107.4
C86—C85—H85C	108.3	C89'—C88'—H88A	108.4
C86—C85—H85D	108.3	C89'—C88'—H88B	108.4
C85—C86—H86C	108.6	C88'—C89'—H89A	108.7
C85—C86—H86D	108.6	C88'—C89'—H89B	108.7
C85—C86—C87	114.6 (9)	H89A—C89'—H89B	107.6
H86C—C86—H86D	107.6	C90'—C89'—C88'	114.4 (14)
C87—C86—H86C	108.6	C90'—C89'—H89A	108.7
C87—C86—H86D	108.6	C90'—C89'—H89B	108.7
C86—C87—H87C	108.6	C89'—C90'—H90A	109.5
C86—C87—H87D	108.6	C89'—C90'—H90B	109.5
C86—C87—C88	114.8 (10)	C89'—C90'—H90C	109.5
H87C—C87—H87D	107.5	H90A—C90'—H90B	109.5
C88—C87—H87C	108.6	H90A—C90'—H90C	109.5
C88—C87—H87D	108.6	H90B—C90'—H90C	109.5
C87—C88—H88C	108.7	C36'—C91'—H91A	108.8
C87—C88—H88D	108.7	C36'—C91'—H91B	108.8
H88C—C88—H88D	107.6	H91A—C91'—H91B	107.7
C89—C88—C87	114.1 (10)	C92'—C91'—C36'	113.7 (13)
C89—C88—H88C	108.7	C92'—C91'—H91A	108.8
C89—C88—H88D	108.7	C92'—C91'—H91B	108.8
C88—C89—H89C	109.0	C91'—C92'—H92A	107.5
C88—C89—H89D	109.0	C91'—C92'—H92B	107.5
C88—C89—C90	113.1 (12)	H92A—C92'—H92B	107.0
H89C—C89—H89D	107.8	C93'—C92'—C91'	119.1 (19)
C90—C89—H89C	109.0	C93'—C92'—H92A	107.5
C90—C89—H89D	109.0	C93'—C92'—H92B	107.5
C89—C90—H90D	109.5	C92'—C93'—H93A	108.0
C89—C90—H90E	109.5	C92'—C93'—H93B	108.0
C89—C90—H90F	109.5	C92'—C93'—C94'	117 (2)
H90D—C90—H90E	109.5	H93A—C93'—H93B	107.3
H90D—C90—H90F	109.5	C94'—C93'—H93A	108.0
H90E—C90—H90F	109.5	C94'—C93'—H93B	108.0
C36—C91—H91C	108.3	C93'—C94'—H94A	109.0
C36—C91—H91D	108.3	C93'—C94'—H94B	109.0
H91C—C91—H91D	107.4	C93'—C94'—C95'	113 (2)
C92—C91—C36	115.8 (9)	H94A—C94'—H94B	107.8
C92—C91—H91C	108.3	C95'—C94'—H94A	109.0
C92—C91—H91D	108.3	C95'—C94'—H94B	109.0
C91—C92—H92C	109.0	C94'—C95'—H95A	110.2
C91—C92—H92D	109.0	C94'—C95'—H95B	110.2
C91—C92—C93	113.0 (9)	H95A—C95'—H95B	108.5
H92C—C92—H92D	107.8	C96'—C95'—C94'	108 (2)
C93—C92—H92C	109.0	C96'—C95'—H95A	110.2
C93—C92—H92D	109.0	C96'—C95'—H95B	110.2
C92—C93—H93C	108.6	C95'—C96'—H96A	109.5
C92—C93—H93D	108.6	C95'—C96'—H96B	109.5
H93C—C93—H93D	107.6	C95'—C96'—H96C	109.5
C94—C93—C92	114.6 (10)	H96A—C96'—H96B	109.5
C94—C93—H93C	108.6	H96A—C96'—H96C	109.5
C94—C93—H93D	108.6	H96B—C96'—H96C	109.5
C93—C94—H94C	109.5	C54'—C97'—H97A	108.7
C93—C94—H94D	109.5	C54'—C97'—H97B	108.7
C93—C94—C95	110.9 (12)	H97A—C97'—H97B	107.6
H94C—C94—H94D	108.1	C98'—C97'—C54'	114.4 (12)
C95—C94—H94C	109.5	C98'—C97'—H97A	108.7
C95—C94—H94D	109.5	C98'—C97'—H97B	108.7
C94—C95—H95C	108.5	C97'—C98'—H98A	109.4
C94—C95—H95D	108.5	C97'—C98'—H98B	109.4
C94—C95—C96	115.0 (15)	C97'—C98'—C99'	111.1 (13)
H95C—C95—H95D	107.5	H98A—C98'—H98B	108.0
C96—C95—H95C	108.5	C99'—C98'—H98A	109.4
C96—C95—H95D	108.5	C99'—C98'—H98B	109.4
C95—C96—H96D	109.5	C98'—C99'—H99A	109.2
C95—C96—H96E	109.5	C98'—C99'—H99B	109.2
C95—C96—H96F	109.5	H99A—C99'—H99B	107.9
H96D—C96—H96E	109.5	C200—C99'—C98'	112.2 (15)
H96D—C96—H96F	109.5	C200—C99'—H99A	109.2
H96E—C96—H96F	109.5	C200—C99'—H99B	109.2
C54—C97—H97C	108.1	C99'—C200—H20A	110.2
C54—C97—H97D	108.1	C99'—C200—H20B	110.2
H97C—C97—H97D	107.3	C99'—C200—C201	107.5 (18)
C98—C97—C54	116.6 (10)	H20A—C200—H20B	108.5
C98—C97—H97C	108.1	C201—C200—H20A	110.2
C98—C97—H97D	108.1	C201—C200—H20B	110.2
C97—C98—H98C	110.1	C200—C201—H20C	108.7
C97—C98—H98D	110.1	C200—C201—H20D	108.7
C97—C98—C99	108.1 (11)	C200—C201—C202	114 (2)
H98C—C98—H98D	108.4	H20C—C201—H20D	107.6
C99—C98—H98C	110.1	C202—C201—H20C	108.7
C99—C98—H98D	110.1	C202—C201—H20D	108.7
C98—C99—H99C	108.8	C201—C202—H20E	109.5
C98—C99—H99D	108.8	C201—C202—H20F	109.5
H99C—C99—H99D	107.7	C201—C202—H20G	109.5
C100—C99—C98	114.0 (13)	H20E—C202—H20F	109.5
C100—C99—H99C	108.8	H20E—C202—H20G	109.5
C100—C99—H99D	108.8	H20F—C202—H20G	109.5
C99—C100—H10C	109.1	C54'—C203—H20H	108.9
C99—C100—H10D	109.1	C54'—C203—H20I	108.9
C99—C100—H10A	109.1	C54'—C203—H20J	105.6
C99—C100—H10B	109.1	C54'—C203—H20K	105.6
C99—C100—C101	112.3 (16)	H20H—C203—H20I	107.7
C99—C100—C126	113 (3)	H20J—C203—H20K	106.1
H10C—C100—H10D	107.8	C204—C203—C54'	113.5 (13)
H10A—C100—H10B	107.9	C204—C203—H20H	108.9
C101—C100—H10A	109.2	C204—C203—H20I	108.9
C101—C100—H10B	109.1	C20B—C203—C54'	127 (3)
C126—C100—H10C	109.1	C20B—C203—H20J	105.6
C126—C100—H10D	109.1	C20B—C203—H20K	105.6
C54—C103—H10E	108.0	H20N—C206—H20O	110.5
C54—C103—H10F	108.0	H20L—C206—H20M	106.6
H10E—C103—H10F	107.3	C207—C206—H20N	113.1
C104—C103—C54	117.1 (10)	C207—C206—H20O	113.1
C104—C103—H10E	108.0	C207—C206—H20L	106.6
C104—C103—H10F	108.0	C207—C206—H20M	106.6
C103—C104—H10G	107.3	C207—C206—C205	123 (2)
C103—C104—H10H	107.3	C207—C206—C20C	92.9 (19)
C103—C104—H10I	112.1	C205—C206—H20L	106.6
C103—C104—H10J	112.1	C205—C206—H20M	106.6
C103—C104—C105	119.9 (12)	C20C—C206—H20N	113.1
C103—C104—C10A	98.5 (17)	C20C—C206—H20O	113.1
H10G—C104—H10H	106.9	C206—C207—H20P	109.4
H10I—C104—H10J	109.7	C206—C207—H20Q	109.4
C105—C104—H10G	107.3	C206—C207—C208	111.2 (16)
C105—C104—H10H	107.3	H20P—C207—H20Q	108.0
C10A—C104—H10I	112.1	C208—C207—H20P	109.4
C10A—C104—H10J	112.1	C208—C207—H20Q	109.4
C72—C109—H10K	108.6	C207—C208—H20R	109.5
C72—C109—H10L	108.6	C207—C208—H20S	109.5
H10K—C109—H10L	107.6	C207—C208—H20T	109.5
C110—C109—C72	114.7 (10)	H20R—C208—H20S	109.5
C110—C109—H10K	108.6	H20R—C208—H20T	109.5
C110—C109—H10L	108.6	H20S—C208—H20T	109.5
C109—C110—H11A	109.2	C72'—C209—H20U	108.1
C109—C110—H11B	109.2	C72'—C209—H20V	108.1
C109—C110—C111	112.1 (12)	H20U—C209—H20V	107.3
H11A—C110—H11B	107.9	C210—C209—C72'	116.8 (11)
C111—C110—H11A	109.2	C210—C209—H20U	108.1
C111—C110—H11B	109.2	C210—C209—H20V	108.1
C110—C111—H11C	108.2	C209—C210—H21A	108.7
C110—C111—H11D	108.2	C209—C210—H21B	108.7
H11C—C111—H11D	107.4	H21A—C210—H21B	107.6
C112—C111—C110	116.3 (16)	C211—C210—C209	114.1 (12)
C112—C111—H11C	108.2	C211—C210—H21A	108.7
C112—C111—H11D	108.2	C211—C210—H21B	108.7
C111—C112—H11E	110.0	C210—C211—H21C	107.8
C111—C112—H11F	110.0	C210—C211—H21D	107.8
C111—C112—C113	109 (2)	C210—C211—H21E	109.6
H11E—C112—H11F	108.3	C210—C211—H21F	109.6
C113—C112—H11E	110.0	C210—C211—C21B	118.2 (18)
C113—C112—H11F	110.0	C210—C211—C212	110.1 (17)
C112—C113—H11G	108.1	H21C—C211—H21D	107.1
C112—C113—H11H	108.1	H21E—C211—H21F	108.1
C112—C113—C114	117 (3)	C21B—C211—H21C	107.8
H11G—C113—H11H	107.3	C21B—C211—H21D	107.8
C114—C113—H11G	108.1	C212—C211—H21E	109.6
C114—C113—H11H	108.1	C212—C211—H21F	109.6
C113—C114—H11I	109.5	C72'—C215—H21G	108.6
C113—C114—H11J	109.5	C72'—C215—H21H	108.6
C113—C114—H11K	109.5	H21G—C215—H21H	107.6
H11I—C114—H11J	109.5	C216—C215—C72'	114.7 (13)
H11I—C114—H11K	109.5	C216—C215—H21G	108.6
H11J—C114—H11K	109.5	C216—C215—H21H	108.6
C72—C115—H11L	107.9	C215—C216—H21I	108.1
C72—C115—H11M	107.9	C215—C216—H21J	108.1
H11L—C115—H11M	107.2	C215—C216—H21K	101.8
C116—C115—C72	117.5 (9)	C215—C216—H21L	101.8
C116—C115—H11L	107.9	C215—C216—C217	117 (2)
C116—C115—H11M	107.9	C215—C216—C21E	141 (2)
C115—C116—H11N	108.6	H21I—C216—H21J	107.3
C115—C116—H11O	108.6	H21K—C216—H21L	104.7
H11N—C116—H11O	107.6	C217—C216—H21I	108.1
C117—C116—C115	114.5 (9)	C217—C216—H21J	108.1
C117—C116—H11N	108.6	C21E—C216—H21K	101.8
C117—C116—H11O	108.6	C21E—C216—H21L	101.8
C116—C117—H11P	108.5	O3'—C221—H22A	109.5
C116—C117—H11Q	108.5	O3'—C221—H22B	109.5
C116—C117—C118	115.2 (10)	O3'—C221—H22C	109.5
H11P—C117—H11Q	107.5	H22A—C221—H22B	109.5
C118—C117—H11P	108.5	H22A—C221—H22C	109.5
C118—C117—H11Q	108.5	H22B—C221—H22C	109.5
C117—C118—H11R	109.0	O4'—C222—H22D	109.5
C117—C118—H11S	109.0	O4'—C222—H22E	109.5
C117—C118—C119	112.8 (10)	O4'—C222—H22F	109.5
H11R—C118—H11S	107.8	H22D—C222—H22E	109.5
C119—C118—H11R	109.0	H22D—C222—H22F	109.5
C119—C118—H11S	109.0	H22E—C222—H22F	109.5
C118—C119—H11T	108.6	O5'—C223—H22G	109.5
C118—C119—H11U	108.6	O5'—C223—H22H	109.5
H11T—C119—H11U	107.6	O5'—C223—H22I	109.5
C120—C119—C118	114.5 (11)	H22G—C223—H22H	109.5
C120—C119—H11T	108.6	H22G—C223—H22I	109.5
C120—C119—H11U	108.6	H22H—C223—H22I	109.5
C119—C120—H12A	109.5	O6'—C224—H22J	109.5
C119—C120—H12B	109.5	O6'—C224—H22K	109.5
C119—C120—H12C	109.5	O6'—C224—H22L	109.5
H12A—C120—H12B	109.5	H22J—C224—H22K	109.5
H12A—C120—H12C	109.5	H22J—C224—H22L	109.5
H12B—C120—H12C	109.5	H22K—C224—H22L	109.5
O3—C121—H12D	109.5	C211—C21B—H21M	107.5
O3—C121—H12E	109.5	C211—C21B—H21N	107.5
O3—C121—H12F	109.5	H21M—C21B—H21N	107.0
H12D—C121—H12E	109.5	C21C—C21B—C211	119 (2)
H12D—C121—H12F	109.5	C21C—C21B—H21M	107.5
H12E—C121—H12F	109.5	C21C—C21B—H21N	107.5
O4—C122—H12G	109.5	C21B—C21C—H21O	107.8
O4—C122—H12H	109.5	C21B—C21C—H21P	107.8
O4—C122—H12I	109.5	C21B—C21C—C21D	118 (3)
H12G—C122—H12H	109.5	H21O—C21C—H21P	107.1
H12G—C122—H12I	109.5	C21D—C21C—H21O	107.8
H12H—C122—H12I	109.5	C21D—C21C—H21P	107.8
O5—C123—H12J	109.5	C21C—C21D—H21Q	109.5
O5—C123—H12K	109.5	C21C—C21D—H21R	109.5
O5—C123—H12L	109.5	C21C—C21D—H21S	109.5
H12J—C123—H12K	109.5	H21Q—C21D—H21R	109.5
H12J—C123—H12L	109.5	H21Q—C21D—H21S	109.5
H12K—C123—H12L	109.5	H21R—C21D—H21S	109.5
O6—C124—H12M	109.5	C79'—C80'—C81'	104.2 (18)
O6—C124—H12N	109.5	C79'—C80'—H80A	110.9
O6—C124—H12O	109.5	C79'—C80'—H80B	110.9
H12M—C124—H12N	109.5	C81'—C80'—H80A	110.9
H12M—C124—H12O	109.5	C81'—C80'—H80B	110.9
H12N—C124—H12O	109.5	H80A—C80'—H80B	108.9
C100—C101—H10M	108.5	C203—C204—H20W	109.1
C100—C101—H10N	108.5	C203—C204—H20X	109.1
H10M—C101—H10N	107.5	H20W—C204—H20X	107.9
C102—C101—C100	115 (2)	C205—C204—C203	112.4 (15)
C102—C101—H10M	108.5	C205—C204—H20W	109.1
C102—C101—H10N	108.5	C205—C204—H20X	109.1
C101—C102—H10O	109.5	C206—C205—C204	102.5 (15)
C101—C102—H10P	109.5	C206—C205—H20Y	111.3
C101—C102—H10Q	109.5	C206—C205—HF	111.3
H10O—C102—H10P	109.5	C204—C205—H20Y	111.3
H10O—C102—H10Q	109.5	C204—C205—HF	111.3
H10P—C102—H10Q	109.5	H20Y—C205—HF	109.2
C104—C105—H10R	108.6	C216—C217—H21T	109.6
C104—C105—H10S	108.6	C216—C217—H21U	109.6
C104—C105—C106	114.8 (14)	C216—C217—C218	110 (3)
H10R—C105—H10S	107.6	H21T—C217—H21U	108.1
C106—C105—H10R	108.6	C218—C217—H21T	109.6
C106—C105—H10S	108.6	C218—C217—H21U	109.6
C105—C106—H10T	108.7	C217—C218—H21V	107.0
C105—C106—H10U	108.7	C217—C218—H21W	107.0
C105—C106—C107	114.4 (15)	C217—C218—C219	121 (4)
H10T—C106—H10U	107.6	H21V—C218—H21W	106.7
C107—C106—H10T	108.7	C219—C218—H21V	107.0
C107—C106—H10U	108.7	C219—C218—H21W	107.0
C106—C107—H10V	109.2	C218—C219—H21X	111.0
C106—C107—H10W	109.2	C218—C219—H21Y	111.0
C106—C107—C108	112 (2)	H21X—C219—H21Y	109.0
H10V—C107—H10W	107.9	C220—C219—C218	104 (3)
C108—C107—H10V	109.2	C220—C219—H21X	111.0
C108—C107—H10W	109.2	C220—C219—H21Y	111.0
C107—C108—H10X	109.5	C219—C220—H22M	109.5
C107—C108—H10Y	109.5	C219—C220—H22N	109.5
C107—C108—H	109.5	C219—C220—H22O	109.5
H10X—C108—H10Y	109.5	H22M—C220—H22N	109.5
H10X—C108—H	109.5	H22M—C220—H22O	109.5
H10Y—C108—H	109.5	H22N—C220—H22O	109.5
C104—C10A—H10Z	109.1	C203—C20B—H20Z	109.8
C104—C10A—HA	109.1	C203—C20B—HG	109.8
H10Z—C10A—HA	107.9	C203—C20B—C20C	109 (2)
C10B—C10A—C104	112 (3)	H20Z—C20B—HG	108.2
C10B—C10A—H10Z	109.1	C20C—C20B—H20Z	109.8
C10B—C10A—HA	109.1	C20C—C20B—HG	109.8
C10A—C10B—H10	108.1	C206—C20C—H20	111.0
C10A—C10B—HB	108.1	C206—C20C—HH	111.0
C10A—C10B—C10C	117 (4)	C20B—C20C—C206	104.0 (19)
H10—C10B—HB	107.3	C20B—C20C—H20	111.0
C10C—C10B—H10	108.1	C20B—C20C—HH	111.0
C10C—C10B—HB	108.1	H20—C20C—HH	109.0
C10B—C10C—H7	106.8	C216—C21E—H21Z	105.4
C10B—C10C—HC	106.8	C216—C21E—HI	105.4
H7—C10C—HC	106.6	C216—C21E—C21F	127 (3)
C10D—C10C—C10B	122 (4)	H21Z—C21E—HI	106.0
C10D—C10C—H7	106.8	C21F—C21E—H21Z	105.4
C10D—C10C—HC	106.8	C21F—C21E—HI	105.4
C10C—C10D—H12	109.5	C21E—C21F—H21	95.0
C10C—C10D—HD	109.5	C21E—C21F—HJ	95.0
C10C—C10D—HE	109.5	H21—C21F—HJ	103.2
H12—C10D—HD	109.5	C21G—C21F—C21E	164 (3)
H12—C10D—HE	109.5	C21G—C21F—H21	95.0
HD—C10D—HE	109.5	C21G—C21F—HJ	95.0
H12P—C125—H12Q	109.5	C21F—C21G—H1	106.0
H12P—C125—H12R	109.5	C21F—C21G—HK	106.0
H12Q—C125—H12R	109.5	C21F—C21G—C21H	125 (3)
C126—C125—H12P	109.5	H1—C21G—HK	106.3
C126—C125—H12Q	109.5	C21H—C21G—H1	106.0
C126—C125—H12R	109.5	C21H—C21G—HK	106.0
C100—C126—H12S	105.0	C21G—C21H—H2	109.5
C100—C126—H12T	105.0	C21G—C21H—HL	109.5
C125—C126—C100	129 (4)	C21G—C21H—HM	109.5
C125—C126—H12S	105.0	H2—C21H—HL	109.5
C125—C126—H12T	105.0	H2—C21H—HM	109.5
H12S—C126—H12T	105.9	HL—C21H—HM	109.5
N01'—Ir3—N02'	80.0 (4)	C79'—C80B—C81'	103 (2)
N1'—Ir3—N01'	94.1 (4)	C79'—C80B—H80C	111.1
N1'—Ir3—N02'	90.3 (4)	C79'—C80B—H80D	111.1
N2'—Ir3—N01'	89.7 (4)	C81'—C80B—H80C	111.1
N2'—Ir3—N1'	171.0 (4)	C81'—C80B—H80D	111.1
N2'—Ir3—N02'	98.4 (4)	H80C—C80B—H80D	109.1
N2'—Ir3—C1'	89.6 (5)	C211—C212—H3	109.3
N2'—Ir3—C19'	81.3 (5)	C211—C212—HN	109.3
C1'—Ir3—N01'	97.1 (4)	H3—C212—HN	107.9
C1'—Ir3—N1'	81.9 (5)	C213—C212—C211	112 (2)
C1'—Ir3—N02'	171.5 (5)	C213—C212—H3	109.3
C19'—Ir3—N01'	166.9 (4)	C213—C212—HN	109.3
C19'—Ir3—N1'	96.2 (5)	C212—C213—H4	109.2
C19'—Ir3—N02'	91.9 (4)	C212—C213—HO	109.2
C19'—Ir3—C1'	92.3 (5)	C212—C213—C214	112 (3)
N02'—Ir4—N01'	80.4 (3)	H4—C213—HO	107.9
N3'—Ir4—N01'	92.6 (4)	C214—C213—H4	109.2
N3'—Ir4—N02'	95.9 (4)	C214—C213—HO	109.2
N4'—Ir4—N01'	90.7 (4)	C213—C214—H5	109.5
N4'—Ir4—N02'	91.2 (4)	C213—C214—HP	109.5
N4'—Ir4—N3'	172.6 (4)	C213—C214—HQ	109.5
N4'—Ir4—C37'	96.6 (5)	H5—C214—HP	109.5
N4'—Ir4—C55'	80.5 (5)	H5—C214—HQ	109.5
C37'—Ir4—N01'	171.0 (5)	HP—C214—HQ	109.5
			
Ir1—N1—C7—C6	−3.4 (11)	Ir3—C19'—C24'—C25'	3.8 (14)
Ir1—N1—C7—C8	175.1 (8)	Ir4—N3'—C43'—C42'	5.3 (15)
Ir1—N1—C11—C10	−174.3 (8)	Ir4—N3'—C43'—C44'	−171.0 (10)
Ir1—N2—C25—C24	−0.8 (12)	Ir4—N3'—C47'—C46'	169.3 (11)
Ir1—N2—C25—C26	176.7 (8)	Ir4—N4'—C61'—C60'	−8.3 (12)
Ir1—N2—C29—C28	−172.8 (8)	Ir4—N4'—C61'—C62'	170.2 (9)
Ir1—C1—C2—C3	−173.0 (7)	Ir4—N4'—C65'—C64'	−174.3 (9)
Ir1—C1—C6—C5	174.3 (8)	Ir4—C37'—C38'—C39'	172.3 (9)
Ir1—C1—C6—C7	−8.2 (11)	Ir4—C37'—C42'—C41'	−172.2 (10)
Ir1—C19—C20—C21	−171.0 (8)	Ir4—C37'—C42'—C43'	7.2 (15)
Ir1—C19—C24—C23	174.8 (8)	Ir4—C55'—C56'—C57'	173.1 (8)
Ir1—C19—C24—C25	−10.5 (12)	Ir4—C55'—C60'—C59'	−173.6 (8)
Ir2—N3—C43—C42	2.5 (11)	Ir4—C55'—C60'—C61'	9.4 (13)
Ir2—N3—C43—C44	−177.9 (7)	O3'—C10'—C11'—N1'	178.2 (12)
Ir2—N3—C47—C46	178.3 (7)	O4'—C28'—C29'—N2'	−179.0 (11)
Ir2—N4—C61—C60	0.3 (12)	O5'—C46'—C47'—N3'	179.6 (13)
Ir2—N4—C61—C62	177.7 (8)	O6'—C64'—C65'—N4'	177.8 (10)
Ir2—N4—C65—C64	−177.5 (8)	N01'—Ir4—N4'—C61'	−80.1 (8)
Ir2—C37—C38—C39	−170.6 (8)	N01'—Ir4—N4'—C65'	95.1 (9)
Ir2—C37—C42—C41	174.5 (8)	N1'—C7'—C8'—C9'	−2 (2)
Ir2—C37—C42—C43	−4.2 (11)	N2'—C25'—C26'—C27'	3 (2)
Ir2—C55—C56—C57	−168.3 (8)	N02'—Ir4—N4'—C61'	−160.6 (8)
Ir2—C55—C60—C59	171.1 (9)	N02'—Ir4—N4'—C65'	14.7 (9)
Ir2—C55—C60—C61	−9.2 (12)	N3'—C43'—C44'—C45'	0 (2)
O3—C10—C11—N1	−179.9 (9)	N4'—C61'—C62'—C63'	5.6 (18)
O4—C28—C29—N2	177.8 (10)	C1'—C2'—C3'—C4'	−4 (2)
O5—C46—C47—N3	−177.5 (9)	C1'—C2'—C3'—C12'	−180.0 (12)
O6—C64—C65—N4	−179.6 (10)	C1'—C6'—C7'—N1'	−2.7 (17)
N1—C7—C8—C9	0.0 (16)	C1'—C6'—C7'—C8'	174.2 (13)
N2—C25—C26—C27	−3.0 (18)	C2'—C1'—C6'—C5'	−4.6 (19)
N3—C43—C44—C45	−0.3 (16)	C2'—C1'—C6'—C7'	−178.3 (11)
N4—C61—C62—C63	−1.7 (17)	C2'—C3'—C4'—C5'	4 (2)
C1—C2—C3—C4	−2.6 (16)	C2'—C3'—C4'—C18'	179.9 (12)
C1—C2—C3—C12	179.2 (10)	C2'—C3'—C12'—C13'	−6 (2)
C1—C6—C7—N1	7.6 (13)	C2'—C3'—C12'—C17'	178.1 (13)
C1—C6—C7—C8	−170.9 (10)	C3'—C4'—C5'—C6'	−4 (2)
C2—C1—C6—C5	−1.8 (15)	C3'—C4'—C18'—C17'	3.5 (16)
C2—C1—C6—C7	175.7 (9)	C3'—C4'—C18'—C73'	−115.1 (13)
C2—C3—C4—C5	1.7 (16)	C3'—C4'—C18'—C79'	123.0 (14)
C2—C3—C4—C18	−179.3 (9)	C3'—C12'—C13'—C14'	−173.5 (13)
C2—C3—C12—C13	−1 (2)	C3'—C12'—C17'—C16'	174.3 (12)
C2—C3—C12—C17	178.9 (10)	C3'—C12'—C17'—C18'	0.2 (16)
C3—C4—C5—C6	−0.9 (16)	C4'—C3'—C12'—C13'	178.4 (14)
C3—C4—C18—C17	0.6 (11)	C4'—C3'—C12'—C17'	2.1 (16)
C3—C4—C18—C73	−117.1 (11)	C4'—C5'—C6'—C1'	5 (2)
C3—C4—C18—C79	119.5 (11)	C4'—C5'—C6'—C7'	177.9 (13)
C3—C12—C13—C14	178.8 (11)	C4'—C18'—C73'—C74'	63.8 (16)
C3—C12—C17—C16	−178.6 (9)	C4'—C18'—C79'—C80'	−68 (2)
C3—C12—C17—C18	−0.1 (12)	C4'—C18'—C79'—C80B	−35 (3)
C4—C3—C12—C13	−179.6 (12)	C5'—C4'—C18'—C17'	178.7 (15)
C4—C3—C12—C17	0.5 (12)	C5'—C4'—C18'—C73'	60 (2)
C4—C5—C6—C1	1.1 (16)	C5'—C4'—C18'—C79'	−62 (2)
C4—C5—C6—C7	−176.3 (10)	C5'—C6'—C7'—N1'	−176.2 (12)
C4—C18—C73—C74	49.7 (14)	C5'—C6'—C7'—C8'	1 (2)
C4—C18—C79—C80	−61.0 (14)	C6'—C1'—C2'—C3'	4.4 (19)
C5—C4—C18—C17	179.5 (11)	C6'—C7'—C8'—C9'	−179.0 (13)
C5—C4—C18—C73	61.8 (15)	C7'—N1'—C11'—C10'	−1.5 (19)
C5—C4—C18—C79	−61.6 (15)	C7'—C8'—C9'—C10'	3 (2)
C5—C6—C7—N1	−174.9 (9)	C8'—C9'—C10'—O3'	−179.1 (12)
C5—C6—C7—C8	6.6 (17)	C8'—C9'—C10'—C11'	−2 (2)
C6—C1—C2—C3	2.5 (14)	C9'—C10'—C11'—N1'	2 (2)
C6—C7—C8—C9	178.3 (11)	C11'—N1'—C7'—C6'	178.8 (11)
C7—N1—C11—C10	−0.4 (15)	C11'—N1'—C7'—C8'	1.6 (18)
C7—C8—C9—C10	−0.8 (17)	C12'—C3'—C4'—C5'	−179.2 (13)
C8—C9—C10—O3	−179.5 (10)	C12'—C3'—C4'—C18'	−3.6 (16)
C8—C9—C10—C11	1.0 (16)	C12'—C13'—C14'—C15'	−6 (2)
C9—C10—C11—N1	−0.4 (16)	C12'—C17'—C18'—C4'	−2.1 (16)
C11—N1—C7—C6	−178.0 (9)	C12'—C17'—C18'—C73'	116.3 (13)
C11—N1—C7—C8	0.6 (14)	C12'—C17'—C18'—C79'	−122.3 (13)
C12—C3—C4—C5	−179.7 (10)	C13'—C12'—C17'—C16'	−2 (2)
C12—C3—C4—C18	−0.7 (12)	C13'—C12'—C17'—C18'	−176.6 (12)
C12—C13—C14—C15	−0.9 (17)	C13'—C14'—C15'—C16'	9 (2)
C12—C17—C18—C4	−0.3 (11)	C14'—C15'—C16'—C17'	−9 (2)
C12—C17—C18—C73	118.8 (10)	C15'—C16'—C17'—C12'	6 (2)
C12—C17—C18—C79	−118.9 (11)	C15'—C16'—C17'—C18'	178.4 (15)
C13—C12—C17—C16	1.5 (17)	C16'—C17'—C18'—C4'	−175.4 (15)
C13—C12—C17—C18	−180.0 (10)	C16'—C17'—C18'—C73'	−57 (2)
C13—C14—C15—C16	3.1 (18)	C16'—C17'—C18'—C79'	64 (2)
C14—C15—C16—C17	−3.0 (18)	C17'—C12'—C13'—C14'	2 (2)
C15—C16—C17—C12	0.7 (17)	C17'—C18'—C73'—C74'	−48.1 (16)
C15—C16—C17—C18	−177.5 (11)	C17'—C18'—C79'—C80'	45 (2)
C16—C17—C18—C4	178.0 (11)	C17'—C18'—C79'—C80B	78 (3)
C16—C17—C18—C73	−62.9 (15)	C18'—C4'—C5'—C6'	−179.2 (13)
C16—C17—C18—C79	59.4 (15)	C18'—C73'—C74'—C75'	−148.1 (13)
C17—C12—C13—C14	−1.4 (17)	C18'—C79'—C80'—C81'	−173.4 (18)
C17—C18—C73—C74	−61.6 (14)	C18'—C79'—C80B—C81'	−149 (2)
C17—C18—C79—C80	50.6 (14)	C19'—C20'—C21'—C22'	0 (2)
C18—C4—C5—C6	−179.7 (10)	C19'—C20'—C21'—C30'	−177.0 (12)
C18—C73—C74—C75	176.3 (11)	C19'—C24'—C25'—N2'	−7.1 (15)
C18—C79—C80—C81	164.8 (11)	C19'—C24'—C25'—C26'	171.6 (12)
C19—C20—C21—C22	−3.0 (17)	C20'—C19'—C24'—C23'	−2.3 (18)
C19—C20—C21—C30	177.9 (11)	C20'—C19'—C24'—C25'	178.1 (11)
C19—C24—C25—N2	7.3 (14)	C20'—C21'—C22'—C23'	0 (2)
C19—C24—C25—C26	−169.9 (11)	C20'—C21'—C22'—C36'	−179.9 (12)
C20—C19—C24—C23	0.8 (16)	C20'—C21'—C30'—C31'	4 (3)
C20—C19—C24—C25	175.6 (10)	C20'—C21'—C30'—C35'	178.8 (12)
C20—C21—C22—C23	1.5 (18)	C21'—C22'—C23'—C24'	−2.2 (19)
C20—C21—C22—C36	−179.7 (10)	C21'—C22'—C36'—C35'	2.3 (15)
C20—C21—C30—C31	1 (2)	C21'—C22'—C36'—C85'	−118.2 (14)
C20—C21—C30—C35	−180.0 (11)	C21'—C22'—C36'—C91'	119.7 (13)
C21—C22—C23—C24	1.0 (17)	C21'—C30'—C31'—C32'	174.6 (14)
C21—C22—C36—C35	−0.2 (12)	C21'—C30'—C35'—C34'	179.8 (12)
C21—C22—C36—C85	−118.8 (11)	C21'—C30'—C35'—C36'	0.4 (16)
C21—C22—C36—C91	117.9 (11)	C22'—C21'—C30'—C31'	−173.5 (16)
C21—C30—C31—C32	179.7 (11)	C22'—C21'—C30'—C35'	1.1 (16)
C21—C30—C35—C34	−178.9 (9)	C22'—C23'—C24'—C19'	3.1 (19)
C21—C30—C35—C36	−0.9 (13)	C22'—C23'—C24'—C25'	−177.3 (11)
C22—C21—C30—C31	−178.5 (12)	C22'—C36'—C85'—C86'	56.3 (17)
C22—C21—C30—C35	0.8 (13)	C22'—C36'—C91'—C92'	−60.3 (18)
C22—C23—C24—C19	−2.2 (16)	C23'—C22'—C36'—C35'	−178.2 (14)
C22—C23—C24—C25	−176.6 (10)	C23'—C22'—C36'—C85'	61.4 (19)
C22—C36—C85—C86	46.0 (14)	C23'—C22'—C36'—C91'	−60.8 (19)
C22—C36—C91—C92	−54.5 (12)	C23'—C24'—C25'—N2'	173.3 (11)
C23—C22—C36—C35	178.6 (11)	C23'—C24'—C25'—C26'	−8 (2)
C23—C22—C36—C85	59.9 (16)	C24'—C19'—C20'—C21'	0.6 (17)
C23—C22—C36—C91	−63.3 (15)	C24'—C25'—C26'—C27'	−175.6 (12)
C23—C24—C25—N2	−177.9 (10)	C25'—N2'—C29'—C28'	0.0 (19)
C23—C24—C25—C26	4.9 (19)	C25'—C26'—C27'—C28'	0 (2)
C24—C19—C20—C21	1.8 (16)	C26'—C27'—C28'—O4'	179.2 (11)
C24—C25—C26—C27	174.0 (11)	C26'—C27'—C28'—C29'	−2 (2)
C25—N2—C29—C28	2.3 (16)	C27'—C28'—C29'—N2'	2 (2)
C25—C26—C27—C28	1.3 (18)	C29'—N2'—C25'—C24'	176.1 (10)
C26—C27—C28—O4	−179.5 (10)	C29'—N2'—C25'—C26'	−2.7 (17)
C26—C27—C28—C29	2.1 (18)	C30'—C21'—C22'—C23'	178.2 (11)
C27—C28—C29—N2	−3.9 (17)	C30'—C21'—C22'—C36'	−2.2 (16)
C29—N2—C25—C24	−176.2 (9)	C30'—C31'—C32'—C33'	4 (2)
C29—N2—C25—C26	1.2 (16)	C30'—C35'—C36'—C22'	−1.5 (15)
C30—C21—C22—C23	−179.2 (10)	C30'—C35'—C36'—C85'	117.7 (13)
C30—C21—C22—C36	−0.4 (13)	C30'—C35'—C36'—C91'	−119.6 (13)
C30—C31—C32—C33	−1.0 (18)	C31'—C30'—C35'—C34'	−5 (2)
C30—C35—C36—C22	0.7 (12)	C31'—C30'—C35'—C36'	176.1 (12)
C30—C35—C36—C85	119.0 (10)	C31'—C32'—C33'—C34'	−4 (2)
C30—C35—C36—C91	−116.1 (11)	C32'—C33'—C34'—C35'	0 (2)
C31—C30—C35—C34	0.5 (17)	C33'—C34'—C35'—C30'	4 (2)
C31—C30—C35—C36	178.4 (10)	C33'—C34'—C35'—C36'	−176.3 (15)
C31—C32—C33—C34	0.5 (19)	C34'—C35'—C36'—C22'	179.1 (15)
C32—C33—C34—C35	0.6 (18)	C34'—C35'—C36'—C85'	−62 (2)
C33—C34—C35—C30	−1.1 (16)	C34'—C35'—C36'—C91'	61 (2)
C33—C34—C35—C36	−178.7 (11)	C35'—C30'—C31'—C32'	0 (2)
C34—C35—C36—C22	178.4 (11)	C35'—C36'—C85'—C86'	−57.8 (17)
C34—C35—C36—C85	−63.4 (14)	C35'—C36'—C91'—C92'	52.2 (19)
C34—C35—C36—C91	61.6 (15)	C36'—C22'—C23'—C24'	178.3 (12)
C35—C30—C31—C32	0.6 (18)	C36'—C85'—C86'—C87'	−177.3 (12)
C35—C36—C85—C86	−65.5 (12)	C36'—C91'—C92'—C93'	−176.2 (19)
C35—C36—C91—C92	56.3 (13)	C37'—Ir4—N4'—C61'	105.2 (9)
C36—C22—C23—C24	−177.6 (10)	C37'—Ir4—N4'—C65'	−79.6 (10)
C36—C85—C86—C87	179.0 (10)	C37'—C38'—C39'—C40'	0 (2)
C36—C91—C92—C93	157.6 (9)	C37'—C38'—C39'—C48'	−178.0 (12)
C37—C38—C39—C40	−5.2 (16)	C37'—C42'—C43'—N3'	−8.3 (18)
C37—C38—C39—C48	174.9 (11)	C37'—C42'—C43'—C44'	167.8 (13)
C37—C42—C43—N3	1.0 (13)	C38'—C37'—C42'—C41'	4.2 (19)
C37—C42—C43—C44	−178.5 (10)	C38'—C37'—C42'—C43'	−176.5 (12)
C38—C37—C42—C41	0.4 (15)	C38'—C39'—C40'—C41'	2 (2)
C38—C37—C42—C43	−178.3 (9)	C38'—C39'—C40'—C54'	−174.6 (12)
C38—C39—C40—C41	5.0 (17)	C38'—C39'—C48'—C49'	−9 (2)
C38—C39—C40—C54	−176.6 (9)	C38'—C39'—C48'—C53'	175.7 (13)
C38—C39—C48—C49	−3 (2)	C39'—C40'—C41'—C42'	−2 (2)
C38—C39—C48—C53	179.6 (11)	C39'—C40'—C54'—C53'	−3.6 (14)
C39—C40—C41—C42	−2.2 (16)	C39'—C40'—C54'—C97'	−119.5 (13)
C39—C40—C54—C53	−4.7 (12)	C39'—C40'—C54'—C203	114.9 (13)
C39—C40—C54—C97	−123.8 (11)	C39'—C48'—C49'—C50'	−174.1 (13)
C39—C40—C54—C103	115.6 (11)	C39'—C48'—C53'—C52'	177.2 (12)
C39—C48—C49—C50	−176.8 (12)	C39'—C48'—C53'—C54'	0.4 (16)
C39—C48—C53—C52	177.9 (12)	C40'—C39'—C48'—C49'	172.9 (13)
C39—C48—C53—C54	−2.9 (13)	C40'—C39'—C48'—C53'	−2.8 (15)
C40—C39—C48—C49	177.3 (12)	C40'—C41'—C42'—C37'	−2 (2)
C40—C39—C48—C53	−0.3 (13)	C40'—C41'—C42'—C43'	179.0 (12)
C40—C41—C42—C37	−0.5 (15)	C40'—C54'—C97'—C98'	61.1 (16)
C40—C41—C42—C43	178.1 (10)	C40'—C54'—C203—C204	−44 (2)
C40—C54—C97—C98	55.5 (15)	C40'—C54'—C203—C20B	−64 (3)
C40—C54—C103—C104	−61.1 (15)	C41'—C40'—C54'—C53'	179.8 (13)
C41—C40—C54—C53	173.6 (11)	C41'—C40'—C54'—C97'	63.9 (18)
C41—C40—C54—C97	54.5 (16)	C41'—C40'—C54'—C203	−61.7 (19)
C41—C40—C54—C103	−66.1 (15)	C41'—C42'—C43'—N3'	171.0 (12)
C41—C42—C43—N3	−177.7 (9)	C41'—C42'—C43'—C44'	−13 (2)
C41—C42—C43—C44	2.8 (16)	C42'—C37'—C38'—C39'	−3.4 (19)
C42—C37—C38—C39	2.5 (15)	C42'—C43'—C44'—C45'	−176.3 (14)
C42—C43—C44—C45	179.2 (10)	C43'—N3'—C47'—C46'	−1 (2)
C43—N3—C47—C46	−1.2 (15)	C43'—C44'—C45'—C46'	1 (2)
C43—C44—C45—C46	−1.2 (16)	C44'—C45'—C46'—O5'	−180.0 (13)
C44—C45—C46—O5	178.8 (9)	C44'—C45'—C46'—C47'	−2 (3)
C44—C45—C46—C47	1.5 (17)	C45'—C46'—C47'—N3'	2 (2)
C45—C46—C47—N3	−0.4 (16)	C47'—N3'—C43'—C42'	176.6 (11)
C47—N3—C43—C42	−178.1 (9)	C47'—N3'—C43'—C44'	0 (2)
C47—N3—C43—C44	1.5 (15)	C48'—C39'—C40'—C41'	−179.1 (12)
C48—C39—C40—C41	−175.1 (10)	C48'—C39'—C40'—C54'	4.0 (15)
C48—C39—C40—C54	3.4 (13)	C48'—C49'—C50'—C51'	−2.4 (19)
C48—C49—C50—C51	0 (2)	C48'—C53'—C54'—C40'	1.8 (15)
C48—C53—C54—C40	4.6 (12)	C48'—C53'—C54'—C97'	118.9 (13)
C48—C53—C54—C97	122.6 (11)	C48'—C53'—C54'—C203	−116.6 (13)
C48—C53—C54—C103	−114.6 (11)	C49'—C48'—C53'—C52'	1 (2)
C49—C48—C53—C52	0.0 (19)	C49'—C48'—C53'—C54'	−175.7 (12)
C49—C48—C53—C54	179.2 (10)	C49'—C50'—C51'—C52'	2 (2)
C49—C50—C51—C52	0 (2)	C50'—C51'—C52'—C53'	1 (2)
C50—C51—C52—C53	1 (2)	C51'—C52'—C53'—C48'	−2 (2)
C51—C52—C53—C48	−1 (2)	C51'—C52'—C53'—C54'	174.2 (13)
C51—C52—C53—C54	−179.8 (13)	C52'—C53'—C54'—C40'	−174.7 (14)
C52—C53—C54—C40	−176.3 (13)	C52'—C53'—C54'—C97'	−57.6 (18)
C52—C53—C54—C97	−58.3 (17)	C52'—C53'—C54'—C203	66.9 (18)
C52—C53—C54—C103	64.5 (17)	C53'—C48'—C49'—C50'	1.1 (19)
C53—C48—C49—C50	0.6 (18)	C53'—C54'—C97'—C98'	−49.1 (15)
C53—C54—C97—C98	−56.6 (15)	C53'—C54'—C203—C204	67.8 (19)
C53—C54—C103—C104	51.8 (15)	C53'—C54'—C203—C20B	48 (3)
C54—C40—C41—C42	179.7 (10)	C54'—C40'—C41'—C42'	174.8 (12)
C54—C97—C98—C99	170.5 (12)	C54'—C97'—C98'—C99'	−174.4 (12)
C54—C103—C104—C105	165.6 (14)	C54'—C203—C204—C205	175 (2)
C54—C103—C104—C10A	−178 (2)	C54'—C203—C20B—C20C	103 (4)
C55—C56—C57—C58	−2.4 (17)	C55'—Ir4—N4'—C61'	11.0 (8)
C55—C56—C57—C66	177.5 (10)	C55'—Ir4—N4'—C65'	−173.8 (10)
C55—C60—C61—N4	5.7 (14)	C55'—C56'—C57'—C58'	0.5 (17)
C55—C60—C61—C62	−171.5 (10)	C55'—C56'—C57'—C66'	−179.1 (11)
C56—C55—C60—C59	−2.3 (16)	C55'—C60'—C61'—N4'	−1.4 (14)
C56—C55—C60—C61	177.3 (9)	C55'—C60'—C61'—C62'	−179.8 (11)
C56—C57—C58—C59	−1.9 (18)	C56'—C55'—C60'—C59'	3.3 (17)
C56—C57—C58—C72	177.5 (10)	C56'—C55'—C60'—C61'	−173.7 (10)
C56—C57—C66—C67	3 (2)	C56'—C57'—C58'—C59'	2.3 (17)
C56—C57—C66—C71	−177.6 (11)	C56'—C57'—C58'—C72'	−175.7 (10)
C57—C58—C59—C60	3.9 (17)	C56'—C57'—C66'—C67'	−1 (2)
C57—C58—C72—C71	1.5 (12)	C56'—C57'—C66'—C71'	176.6 (11)
C57—C58—C72—C109	−117.2 (11)	C57'—C58'—C59'—C60'	−2.2 (16)
C57—C58—C72—C115	122.4 (11)	C57'—C58'—C72'—C71'	−3.3 (13)
C57—C66—C67—C68	179.3 (12)	C57'—C58'—C72'—C209	−121.1 (12)
C57—C66—C71—C70	180.0 (10)	C57'—C58'—C72'—C215	116.5 (12)
C57—C66—C71—C72	−1.4 (13)	C57'—C66'—C67'—C68'	179.9 (11)
C58—C57—C66—C67	−176.8 (12)	C57'—C66'—C71'—C70'	−179.0 (11)
C58—C57—C66—C71	2.3 (13)	C57'—C66'—C71'—C72'	1.0 (14)
C58—C59—C60—C55	−1.8 (17)	C58'—C57'—C66'—C67'	178.9 (12)
C58—C59—C60—C61	178.6 (10)	C58'—C57'—C66'—C71'	−3.1 (13)
C58—C72—C109—C110	57.9 (14)	C58'—C59'—C60'—C55'	−0.6 (17)
C58—C72—C115—C116	−59.2 (14)	C58'—C59'—C60'—C61'	176.2 (10)
C59—C58—C72—C71	−179.2 (11)	C58'—C72'—C209—C210	53.8 (16)
C59—C58—C72—C109	62.1 (15)	C58'—C72'—C215—C216	−53.0 (18)
C59—C58—C72—C115	−58.3 (16)	C59'—C58'—C72'—C71'	179.0 (11)
C59—C60—C61—N4	−174.6 (10)	C59'—C58'—C72'—C209	61.2 (16)
C59—C60—C61—C62	8.1 (18)	C59'—C58'—C72'—C215	−61.2 (17)
C60—C55—C56—C57	4.4 (15)	C59'—C60'—C61'—N4'	−178.4 (10)
C60—C61—C62—C63	175.3 (11)	C59'—C60'—C61'—C62'	3.2 (19)
C61—N4—C65—C64	−2.4 (17)	C60'—C55'—C56'—C57'	−3.2 (16)
C61—C62—C63—C64	1.5 (18)	C60'—C61'—C62'—C63'	−176.2 (11)
C62—C63—C64—O6	−179.9 (10)	C61'—N4'—C65'—C64'	1.4 (16)
C62—C63—C64—C65	−1.7 (19)	C61'—C62'—C63'—C64'	−0.2 (18)
C63—C64—C65—N4	2.3 (18)	C62'—C63'—C64'—O6'	−178.4 (10)
C65—N4—C61—C60	−175.3 (9)	C62'—C63'—C64'—C65'	−3.9 (19)
C65—N4—C61—C62	2.1 (16)	C63'—C64'—C65'—N4'	3 (2)
C66—C57—C58—C59	178.2 (10)	C65'—N4'—C61'—C60'	175.8 (9)
C66—C57—C58—C72	−2.4 (13)	C65'—N4'—C61'—C62'	−5.8 (15)
C66—C67—C68—C69	1.4 (19)	C66'—C57'—C58'—C59'	−178.0 (10)
C66—C71—C72—C58	0.0 (12)	C66'—C57'—C58'—C72'	4.0 (13)
C66—C71—C72—C109	116.9 (11)	C66'—C67'—C68'—C69'	−3.0 (17)
C66—C71—C72—C115	−121.1 (11)	C66'—C71'—C72'—C58'	1.3 (13)
C67—C66—C71—C70	−0.8 (18)	C66'—C71'—C72'—C209	119.3 (12)
C67—C66—C71—C72	177.8 (10)	C66'—C71'—C72'—C215	−118.0 (12)
C67—C68—C69—C70	−3 (2)	C67'—C66'—C71'—C70'	−0.7 (18)
C68—C69—C70—C71	2 (2)	C67'—C66'—C71'—C72'	179.2 (11)
C69—C70—C71—C66	−0.5 (19)	C67'—C68'—C69'—C70'	2.7 (19)
C69—C70—C71—C72	−178.8 (12)	C68'—C69'—C70'—C71'	−1.4 (19)
C70—C71—C72—C58	178.4 (12)	C69'—C70'—C71'—C66'	0.4 (19)
C70—C71—C72—C109	−64.7 (16)	C69'—C70'—C71'—C72'	−179.6 (13)
C70—C71—C72—C115	57.3 (17)	C70'—C71'—C72'—C58'	−178.7 (12)
C71—C66—C67—C68	0.3 (18)	C70'—C71'—C72'—C209	−60.8 (18)
C71—C72—C109—C110	−53.1 (14)	C70'—C71'—C72'—C215	62.0 (18)
C71—C72—C115—C116	54.2 (14)	C71'—C66'—C67'—C68'	2.0 (17)
C72—C58—C59—C60	−175.3 (10)	C71'—C72'—C209—C210	−58.5 (16)
C72—C109—C110—C111	−176.1 (13)	C71'—C72'—C215—C216	60.5 (18)
C72—C115—C116—C117	179.7 (11)	C72'—C58'—C59'—C60'	175.4 (11)
C73—C18—C79—C80	173.8 (10)	C72'—C209—C210—C211	−179.0 (12)
C73—C74—C75—C76	70 (2)	C72'—C215—C216—C217	180 (2)
C74—C75—C76—C77	−167.4 (15)	C72'—C215—C216—C21E	145 (4)
C75—C76—C77—C78	−62 (3)	C73'—C18'—C79'—C80'	168.3 (15)
C79—C18—C73—C74	174.4 (10)	C73'—C18'—C79'—C80B	−159 (3)
C79—C80—C81—C82	−179.8 (11)	C73'—C74'—C75'—C76'	69 (2)
C80—C81—C82—C83	170.5 (13)	C74'—C75'—C76'—C77'	83 (2)
C81—C82—C83—C84	−178.9 (16)	C75'—C76'—C77'—C78'	−174.6 (14)
C85—C36—C91—C92	−178.0 (9)	C79'—C18'—C73'—C74'	−171.6 (13)
C85—C86—C87—C88	172.2 (10)	C81'—C82'—C83'—C84'	172 (3)
C86—C87—C88—C89	−61.6 (15)	C82'—C81'—C80'—C79'	82 (3)
C87—C88—C89—C90	−174.0 (12)	C82'—C81'—C80B—C79'	26 (8)
C91—C36—C85—C86	168.9 (10)	C85'—C36'—C91'—C92'	176.7 (14)
C91—C92—C93—C94	179.0 (9)	C85'—C86'—C87'—C88'	176.7 (12)
C92—C93—C94—C95	174.0 (10)	C86'—C87'—C88'—C89'	−170.1 (13)
C93—C94—C95—C96	−175.3 (12)	C87'—C88'—C89'—C90'	173.9 (14)
C97—C54—C103—C104	176.8 (11)	C91'—C36'—C85'—C86'	179.3 (12)
C97—C98—C99—C100	172.8 (13)	C91'—C92'—C93'—C94'	174 (2)
C98—C99—C100—C101	169 (2)	C92'—C93'—C94'—C95'	−78 (3)
C98—C99—C100—C126	−179 (3)	C93'—C94'—C95'—C96'	−165 (2)
C99—C100—C101—C102	172 (2)	C97'—C54'—C203—C204	−169.1 (16)
C99—C100—C126—C125	−66 (8)	C97'—C54'—C203—C20B	171 (2)
C103—C54—C97—C98	178.0 (11)	C97'—C98'—C99'—C200	−174.6 (14)
C103—C104—C105—C106	−177.3 (17)	C98'—C99'—C200—C201	79 (2)
C103—C104—C10A—C10B	179 (3)	C99'—C200—C201—C202	64 (2)
C104—C105—C106—C107	175 (2)	C203—C54'—C97'—C98'	−173.4 (12)
C104—C10A—C10B—C10C	54 (6)	C203—C204—C205—C206	−173.3 (19)
C109—C72—C115—C116	179.0 (10)	C203—C20B—C20C—C206	174 (3)
C109—C110—C111—C112	−167.1 (17)	C207—C206—C205—C204	95 (3)
C110—C111—C112—C113	−67 (3)	C207—C206—C20C—C20B	171 (4)
C111—C112—C113—C114	−64 (3)	C209—C72'—C215—C216	−176.2 (13)
C115—C72—C109—C110	−178.3 (11)	C209—C210—C211—C21B	−164.6 (17)
C115—C116—C117—C118	−173.6 (10)	C209—C210—C211—C212	164.3 (19)
C116—C117—C118—C119	177.9 (11)	C210—C211—C21B—C21C	−72 (4)
C117—C118—C119—C120	−174.3 (11)	C210—C211—C212—C213	−169 (3)
C121—O3—C10—C9	174.1 (10)	C211—C21B—C21C—C21D	175 (3)
C121—O3—C10—C11	−6.4 (15)	C211—C212—C213—C214	−168 (3)
C122—O4—C28—C27	170.0 (11)	C215—C72'—C209—C210	177.4 (12)
C122—O4—C28—C29	−11.8 (17)	C215—C216—C217—C218	−164 (3)
C123—O5—C46—C45	176.6 (10)	C215—C216—C21E—C21F	−175 (3)
C123—O5—C46—C47	−6.2 (15)	C216—C217—C218—C219	85 (5)
C124—O6—C64—C63	171.6 (11)	C216—C21E—C21F—C21G	152 (12)
C124—O6—C64—C65	−6.6 (17)	C221—O3'—C10'—C9'	−175.9 (13)
C105—C106—C107—C108	−176 (2)	C221—O3'—C10'—C11'	8 (2)
C10A—C10B—C10C—C10D	−176 (5)	C222—O4'—C28'—C27'	−167.4 (12)
Ir3—N1'—C7'—C6'	−3.8 (14)	C222—O4'—C28'—C29'	14 (2)
Ir3—N1'—C7'—C8'	179.1 (10)	C223—O5'—C46'—C45'	−178.3 (14)
Ir3—N1'—C11'—C10'	−178.6 (10)	C223—O5'—C46'—C47'	4 (2)
Ir3—N2'—C25'—C24'	7.5 (13)	C224—O6'—C64'—C63'	−179.6 (12)
Ir3—N2'—C25'—C26'	−171.3 (10)	C224—O6'—C64'—C65'	6.0 (18)
Ir3—N2'—C29'—C28'	167.3 (11)	C80'—C81'—C82'—C83'	161 (3)
Ir3—C1'—C2'—C3'	177.1 (9)	C205—C206—C207—C208	161 (2)
Ir3—C1'—C6'—C5'	−178.5 (10)	C217—C218—C219—C220	163 (4)
Ir3—C1'—C6'—C7'	7.8 (15)	C20C—C206—C207—C208	−170 (3)
Ir3—C19'—C20'—C21'	174.0 (9)	C21E—C21F—C21G—C21H	−146 (12)
Ir3—C19'—C24'—C23'	−176.7 (9)	C80B—C81'—C82'—C83'	−176 (5)

### {µ-N,N'-Bis[3,5-bis(trifluoromethyl)phenyl]oxamidato}bis(bis{2-[4-(2,4,6-trimethylphenyl)pyridin-2-yl]phenyl-κ2C1,N'}iridium)–chlorobenzene–pentane (1/2.3/0.4) (IV) Crystal data

**Table:**

[Ir_2_(C_20_H_19_N)_4_(C_18_H_6_F_12_N_2_O_2_)]·2.3C_6_H_5_Cl·0.4C_5_H_12_	*Z* = 1
*M**_r_* = 2271.78	*F*(000) = 1136
Triclinic, *P*1	*D*_x_ = 1.538 Mg m^−^^3^
*a* = 11.8734 (5) Å	Mo *K*α radiation, λ = 0.71073 Å
*b* = 14.2267 (6) Å	Cell parameters from 9713 reflections
*c* = 16.6076 (7) Å	θ = 2.4–29.9°
α = 110.386 (2)°	µ = 2.85 mm^−^^1^
β = 106.524 (2)°	*T* = 120 K
γ = 96.303 (2)°	Block, yellow
*V* = 2452.00 (18) Å^3^	0.12 × 0.08 × 0.04 mm

### {µ-N,N'-Bis[3,5-bis(trifluoromethyl)phenyl]oxamidato}bis(bis{2-[4-(2,4,6-trimethylphenyl)pyridin-2-yl]phenyl-κ2C1,N'}iridium)–chlorobenzene–pentane (1/2.3/0.4) (IV) Data collection

**Table:**

Bruker D8 Venture diffractometer	14405 independent reflections
Radiation source: microfocus sealed X-ray tube, Incoatec IµS microsource	12696 reflections with *I* > 2σ(*I*)
Focusing mirrors monochromator	*R*_int_ = 0.032
Detector resolution: 10.4 pixels mm^-1^	θ_max_ = 30.1°, θ_min_ = 2.1°
ω–scan	*h* = −16→16
Absorption correction: integration (SADABS; Krause *et al.*, 2015)	*k* = −20→20
*T*_min_ = 0.770, *T*_max_ = 0.913	*l* = −23→23
54411 measured reflections	

### {µ-N,N'-Bis[3,5-bis(trifluoromethyl)phenyl]oxamidato}bis(bis{2-[4-(2,4,6-trimethylphenyl)pyridin-2-yl]phenyl-κ2C1,N'}iridium)–chlorobenzene–pentane (1/2.3/0.4) (IV) Refinement

**Table:**

Refinement on *F*^2^	Primary atom site location: heavy-atom method
Least-squares matrix: full	Secondary atom site location: difference Fourier map
*R*[*F*^2^ > 2σ(*F*^2^)] = 0.028	Hydrogen site location: mixed
*wR*(*F*^2^) = 0.073	H atoms treated by a mixture of independent and constrained refinement
*S* = 1.06	*w* = 1/[σ^2^(*F*_o_^2^) + (0.0372*P*)^2^ + 2.3279*P*] where *P* = (*F*_o_^2^ + 2*F*_c_^2^)/3
14405 reflections	(Δ/σ)_max_ = 0.003
702 parameters	Δρ_max_ = 0.96 e Å^−^^3^
593 restraints	Δρ_min_ = −1.01 e Å^−^^3^

### {µ-N,N'-Bis[3,5-bis(trifluoromethyl)phenyl]oxamidato}bis(bis{2-[4-(2,4,6-trimethylphenyl)pyridin-2-yl]phenyl-κ2C1,N'}iridium)–chlorobenzene–pentane (1/2.3/0.4) (IV) Special details

**Table:**

Experimental. Data were collected in shutterless mode. Full sphere of reciprocal space was nominally covered by 4 runs of 340 narrow-frame ω-scans (scan width 0.5°, 30s exposure), every run at a different φ angle. Two runs of 358 φ-scans (scan width 1°, 3s exposure) were used for scaling overflowing intensities. Crystal to detector distance 3.49 cm.
Geometry. All esds (except the esd in the dihedral angle between two l.s. planes) are estimated using the full covariance matrix. The cell esds are taken into account individually in the estimation of esds in distances, angles and torsion angles; correlations between esds in cell parameters are only used when they are defined by crystal symmetry. An approximate (isotropic) treatment of cell esds is used for estimating esds involving l.s. planes.
Refinement. A strong (4.2 eÅ^-3^) peak of electron density near the Ir atom was interpreted as an alternative position of the same atom with the occupancy of 0.0183 (6), corresponding to a rotation of the whole molecule by ca. 12 °. The alternative positions of light atoms cannot be resolved. The C(30)F_3_ group is disordered by rotation and tilt between orientations A and B with occupancies 0.586 (15) and 0.414 (15), the C(31)F_3_ group is disordered by rotation alone [with the C(31) ordered] between orientations A and B with occupancies 0.776 (5) and 0.224 (5), respectively. The opposite F atoms were refined with identical ADP. The PhCl molecule [C(50) to C(55)] has the Cl atom disordered between positions Cl(1) and Cl(2) with equal occupancies, the former is sterically incompatible with its inversion equivalent. The void of 204 Å^3^ around the inversion centre (0, 0, 0) is shared by disordered PhCl and pentane molecules, with the occupancies tentatively estimated as 0.15 and 0.2, respectively. Methyl group C(48)H_3_ was refined as ideally disordered, other methyl groups as rigid bodies rotating around C—C bonds, with a common refined U for three H atoms. Other H atoms: riding model.

### {µ-N,N'-Bis[3,5-bis(trifluoromethyl)phenyl]oxamidato}bis(bis{2-[4-(2,4,6-trimethylphenyl)pyridin-2-yl]phenyl-κ2C1,N'}iridium)–chlorobenzene–pentane (1/2.3/0.4) (IV) Fractional atomic coordinates and isotropic or equivalent isotropic displacement parameters (Å^2^)

**Table:**

	*x*	*y*	*z*	*U*_iso_*/*U*_eq_	Occ. (<1)
O1	0.44213 (16)	0.40519 (14)	0.53296 (12)	0.0202 (4)	
N1	0.37318 (18)	0.43068 (16)	0.39899 (14)	0.0168 (4)	
N2	0.5291 (2)	0.54309 (18)	0.74258 (15)	0.0199 (4)	
N3	0.6642 (2)	0.35031 (17)	0.56890 (15)	0.0200 (4)	
C1	0.4488 (2)	0.45259 (18)	0.48149 (16)	0.0164 (4)	
C2	0.7416 (2)	0.5027 (2)	0.77180 (17)	0.0195 (5)	
C3	0.8524 (2)	0.4756 (2)	0.7863 (2)	0.0256 (6)	
H3	0.865589	0.424637	0.737133	0.031*	
C4	0.9441 (3)	0.5214 (3)	0.8713 (2)	0.0315 (6)	
H4	1.019180	0.501879	0.879077	0.038*	
C5	0.9276 (3)	0.5951 (3)	0.9445 (2)	0.0354 (7)	
H5	0.991573	0.627520	1.001858	0.042*	
C6	0.8165 (3)	0.6212 (2)	0.9335 (2)	0.0302 (6)	
H6	0.803662	0.670946	0.983629	0.036*	
C7	0.7238 (2)	0.5744 (2)	0.84862 (18)	0.0219 (5)	
C8	0.6009 (2)	0.5903 (2)	0.83196 (18)	0.0210 (5)	
C9	0.5518 (3)	0.6384 (2)	0.89820 (19)	0.0248 (5)	
H9	0.602846	0.672393	0.960529	0.030*	
C10	0.4295 (3)	0.6375 (2)	0.87446 (19)	0.0242 (5)	
C11	0.3605 (3)	0.5938 (2)	0.7816 (2)	0.0287 (6)	
H11	0.277713	0.595695	0.762022	0.034*	
C12	0.4124 (3)	0.5480 (2)	0.71836 (19)	0.0264 (6)	
H12	0.364084	0.518702	0.655240	0.032*	
C13	0.5551 (2)	0.3276 (2)	0.67928 (18)	0.0202 (5)	
C14	0.5066 (3)	0.3197 (2)	0.74506 (19)	0.0254 (5)	
H14	0.496839	0.379959	0.788143	0.030*	
C15	0.4726 (3)	0.2245 (2)	0.7479 (2)	0.0303 (6)	
H15	0.439964	0.220699	0.793019	0.036*	
C16	0.4855 (3)	0.1355 (2)	0.6862 (2)	0.0343 (7)	
H16	0.460195	0.070799	0.688127	0.041*	
C17	0.5353 (3)	0.1407 (2)	0.6217 (2)	0.0306 (6)	
H17	0.545247	0.079808	0.579427	0.037*	
C18	0.5709 (2)	0.2363 (2)	0.61902 (18)	0.0228 (5)	
C19	0.6296 (2)	0.2496 (2)	0.55617 (18)	0.0220 (5)	
C20	0.6518 (3)	0.1718 (2)	0.48812 (19)	0.0264 (6)	
H20	0.627753	0.102136	0.479835	0.032*	
C21	0.7082 (3)	0.1937 (2)	0.43204 (19)	0.0258 (5)	
C22	0.7454 (3)	0.2967 (2)	0.4480 (2)	0.0266 (6)	
H22	0.786309	0.315052	0.412247	0.032*	
C23	0.7224 (2)	0.3720 (2)	0.51617 (19)	0.0234 (5)	
H23	0.748595	0.442040	0.526522	0.028*	
C24	0.2774 (2)	0.3402 (2)	0.36003 (17)	0.0190 (5)	
C25	0.2970 (3)	0.2468 (2)	0.3648 (2)	0.0259 (6)	
H25	0.376434	0.241224	0.392612	0.031*	
C26	0.2006 (3)	0.1623 (2)	0.3288 (2)	0.0318 (6)	
C27	0.0840 (3)	0.1684 (2)	0.2881 (2)	0.0334 (7)	
H27	0.018131	0.110643	0.264945	0.040*	
C28	0.0652 (3)	0.2602 (2)	0.2817 (2)	0.0289 (6)	
C29	0.1609 (2)	0.3458 (2)	0.31702 (18)	0.0225 (5)	
H29	0.146642	0.408140	0.311688	0.027*	
C31	−0.0597 (3)	0.2692 (3)	0.2380 (2)	0.0409 (8)	
C32	0.3731 (2)	0.6731 (2)	0.94620 (19)	0.0254 (5)	
C33	0.3216 (3)	0.6002 (2)	0.9721 (2)	0.0287 (6)	
C34	0.2694 (3)	0.6321 (3)	1.0394 (2)	0.0323 (6)	
H34	0.235092	0.582728	1.057505	0.039*	
C35	0.2665 (3)	0.7346 (3)	1.0806 (2)	0.0329 (7)	
C36	0.3169 (3)	0.8052 (3)	1.0535 (2)	0.0374 (7)	
H36	0.315310	0.875588	1.081340	0.045*	
C37	0.3703 (3)	0.7760 (2)	0.9860 (2)	0.0330 (6)	
C38	0.3230 (4)	0.4870 (3)	0.9286 (3)	0.0453 (9)	
H38A	0.278057	0.459579	0.862655	0.086 (10)*	
H38B	0.285261	0.448142	0.956388	0.086 (10)*	
H38C	0.406693	0.480281	0.938809	0.086 (10)*	
C39	0.2095 (3)	0.7662 (3)	1.1531 (2)	0.0450 (9)	
H39A	0.270928	0.813855	1.211392	0.097 (11)*	
H39B	0.175236	0.704936	1.160351	0.097 (11)*	
H39C	0.145249	0.800287	1.135032	0.097 (11)*	
C40	0.4177 (4)	0.8541 (3)	0.9548 (3)	0.0466 (9)	
H40A	0.502842	0.855475	0.961969	0.077 (9)*	
H40B	0.410483	0.922340	0.991740	0.077 (9)*	
H40C	0.370914	0.835528	0.890202	0.077 (9)*	
C41	0.7266 (3)	0.1100 (2)	0.3561 (2)	0.0306 (6)	
C42	0.6447 (4)	0.0778 (3)	0.2669 (2)	0.0397 (8)	
C43	0.6617 (4)	−0.0006 (3)	0.1963 (3)	0.0513 (10)	
H43	0.605787	−0.023665	0.135712	0.062*	
C44	0.7583 (5)	−0.0455 (3)	0.2126 (3)	0.0544 (11)	
C45	0.8376 (4)	−0.0127 (3)	0.3002 (3)	0.0504 (9)	
H45	0.904509	−0.043287	0.311232	0.060*	
C46	0.8233 (3)	0.0649 (3)	0.3743 (3)	0.0397 (8)	
C47	0.5372 (4)	0.1248 (4)	0.2468 (3)	0.0555 (10)	
H47A	0.491260	0.120515	0.286420	0.086 (10)*	
H47B	0.485398	0.087211	0.182531	0.086 (10)*	
H47C	0.565461	0.197287	0.258418	0.086 (10)*	
C48	0.7757 (6)	−0.1311 (4)	0.1348 (4)	0.0853 (19)	
H48A	0.711099	−0.144750	0.077264	0.102*	0.49 (6)
H48B	0.854058	−0.110100	0.129994	0.102*	0.49 (6)
H48C	0.773044	−0.193831	0.146991	0.102*	0.49 (6)
H48D	0.847702	−0.154371	0.158902	0.102*	0.51 (6)
H48E	0.704742	−0.189020	0.106172	0.102*	0.51 (6)
H48F	0.785757	−0.105290	0.089175	0.102*	0.51 (6)
C49	0.9111 (4)	0.0981 (3)	0.4691 (3)	0.0586 (11)	
H49A	0.939459	0.173347	0.496631	0.088 (10)*	
H49B	0.980080	0.066492	0.466851	0.088 (10)*	
H49C	0.871471	0.076294	0.506083	0.088 (10)*	
Ir1	0.60082 (2)	0.45280 (2)	0.65562 (2)	0.01591 (4)	0.9817 (6)
F1A	0.3215 (4)	0.0447 (4)	0.3300 (6)	0.110 (3)	0.774 (5)
F2A	0.2218 (10)	0.0665 (4)	0.4187 (4)	0.133 (3)	0.774 (5)
F3A	0.1348 (3)	−0.0165 (2)	0.2794 (4)	0.0836 (15)	0.774 (5)
F4A	−0.0993 (8)	0.3252 (12)	0.3001 (5)	0.132 (6)	0.586 (15)
F5A	−0.0690 (5)	0.3071 (8)	0.1789 (5)	0.071 (3)	0.586 (15)
F6A	−0.1381 (6)	0.1809 (6)	0.1988 (9)	0.124 (5)	0.586 (15)
C30A	0.2207 (5)	0.0654 (5)	0.3405 (4)	0.0432 (10)*	0.774 (5)
F1B	0.1533 (17)	0.0148 (18)	0.350 (3)	0.110 (3)	0.226 (5)
F2B	0.275 (3)	0.0131 (14)	0.2641 (13)	0.133 (3)	0.226 (5)
F3B	0.3179 (15)	0.0763 (9)	0.4024 (15)	0.0836 (15)	0.226 (5)
F4B	−0.1086 (10)	0.2027 (15)	0.1528 (5)	0.141 (9)	0.414 (15)
F5B	−0.1364 (7)	0.2479 (9)	0.2736 (8)	0.065 (3)	0.414 (15)
F6B	−0.0671 (7)	0.3626 (8)	0.2420 (15)	0.106 (7)	0.414 (15)
C30B	0.2306 (15)	0.0613 (16)	0.3244 (12)	0.0432 (10)*	0.226 (5)
C50	0.0992 (4)	0.3843 (4)	0.5805 (3)	0.0616 (11)	
H50	0.072881	0.428370	0.550858	0.074*	
C51	0.0560 (4)	0.3792 (4)	0.6477 (4)	0.0669 (13)	
H51	0.001526	0.419267	0.665464	0.080*	
C52	0.0945 (4)	0.3146 (5)	0.6878 (4)	0.0747 (15)	
H52	0.063664	0.306635	0.732506	0.090*	
C53	0.1778 (4)	0.2602 (4)	0.6645 (4)	0.0711 (13)	
H53	0.206506	0.217124	0.694538	0.085*	
C54	0.2183 (4)	0.2691 (4)	0.5981 (3)	0.0677 (13)	
H54	0.276172	0.232433	0.581874	0.081*	
C55	0.1770 (4)	0.3294 (4)	0.5552 (3)	0.0642 (12)	
H55	0.202888	0.333102	0.507062	0.077*	
Cl2	0.0335 (3)	0.2747 (2)	0.7559 (2)	0.0821 (8)	0.5
Cl1	0.0600 (3)	0.4641 (2)	0.5229 (2)	0.0797 (8)	0.5
Cl3	0.2563 (8)	0.0961 (7)	0.0951 (6)	0.066 (2)	0.15
C56	0.1129 (12)	0.0482 (11)	0.0450 (12)	0.055 (4)*	0.3
C57	0.0353 (15)	0.1095 (13)	0.0364 (13)	0.066 (5)*	0.3
H57	0.059102	0.182588	0.060265	0.079*	0.3
C58	0.0752 (13)	−0.0569 (11)	0.0089 (10)	0.058 (4)*	0.3
H58	0.136555	−0.093519	0.018609	0.069*	0.3
C59	0.2059 (15)	0.1337 (12)	0.0963 (8)	0.074 (3)	0.4
C60	0.0793 (19)	0.1029 (15)	0.0474 (17)	0.060 (6)*	0.2
C61	0.000000	0.000000	0.000000	0.074 (3)	0.4
C62	−0.1331 (15)	−0.025 (2)	−0.0543 (16)	0.051 (6)*	0.2
Ir2	0.5679 (7)	0.4090 (8)	0.6317 (5)	0.01591 (4)	0.0183 (6)

### {µ-N,N'-Bis[3,5-bis(trifluoromethyl)phenyl]oxamidato}bis(bis{2-[4-(2,4,6-trimethylphenyl)pyridin-2-yl]phenyl-κ2C1,N'}iridium)–chlorobenzene–pentane (1/2.3/0.4) (IV) Atomic displacement parameters (Å^2^)

**Table:**

	*U*^11^	*U*^22^	*U*^33^	*U*^12^	*U*^13^	*U*^23^
O1	0.0200 (9)	0.0217 (9)	0.0191 (8)	0.0003 (7)	0.0056 (7)	0.0107 (7)
N1	0.0156 (9)	0.0173 (10)	0.0161 (9)	0.0023 (8)	0.0043 (8)	0.0063 (8)
N2	0.0190 (10)	0.0264 (11)	0.0179 (10)	0.0065 (9)	0.0070 (8)	0.0120 (9)
N3	0.0201 (10)	0.0223 (11)	0.0198 (10)	0.0053 (8)	0.0078 (8)	0.0101 (9)
C1	0.0164 (11)	0.0162 (11)	0.0171 (11)	0.0048 (9)	0.0072 (9)	0.0058 (9)
C2	0.0172 (11)	0.0235 (12)	0.0205 (12)	0.0023 (9)	0.0061 (9)	0.0131 (10)
C3	0.0222 (13)	0.0329 (15)	0.0276 (13)	0.0091 (11)	0.0109 (11)	0.0162 (12)
C4	0.0186 (13)	0.0460 (18)	0.0323 (15)	0.0092 (12)	0.0063 (11)	0.0197 (14)
C5	0.0212 (13)	0.0478 (19)	0.0266 (14)	0.0013 (13)	0.0002 (11)	0.0113 (14)
C6	0.0247 (13)	0.0363 (16)	0.0233 (13)	0.0035 (12)	0.0058 (11)	0.0078 (12)
C7	0.0194 (12)	0.0256 (13)	0.0207 (12)	0.0033 (10)	0.0064 (10)	0.0101 (10)
C8	0.0209 (12)	0.0232 (13)	0.0197 (12)	0.0044 (10)	0.0064 (9)	0.0100 (10)
C9	0.0237 (13)	0.0272 (14)	0.0204 (12)	0.0053 (11)	0.0068 (10)	0.0067 (11)
C10	0.0252 (13)	0.0266 (14)	0.0243 (13)	0.0073 (11)	0.0112 (10)	0.0116 (11)
C11	0.0229 (13)	0.0407 (17)	0.0253 (13)	0.0138 (12)	0.0088 (11)	0.0140 (12)
C12	0.0223 (13)	0.0365 (16)	0.0207 (12)	0.0102 (11)	0.0052 (10)	0.0124 (12)
C13	0.0193 (12)	0.0233 (12)	0.0200 (12)	0.0028 (10)	0.0059 (9)	0.0124 (10)
C14	0.0244 (13)	0.0321 (14)	0.0222 (13)	0.0057 (11)	0.0091 (10)	0.0132 (11)
C15	0.0307 (15)	0.0392 (16)	0.0287 (14)	0.0044 (13)	0.0144 (12)	0.0203 (13)
C16	0.0418 (18)	0.0297 (15)	0.0337 (16)	−0.0015 (13)	0.0138 (14)	0.0175 (13)
C17	0.0385 (16)	0.0235 (14)	0.0290 (15)	0.0002 (12)	0.0126 (13)	0.0108 (12)
C18	0.0236 (13)	0.0240 (13)	0.0193 (12)	0.0004 (10)	0.0060 (10)	0.0097 (10)
C19	0.0222 (12)	0.0232 (12)	0.0211 (12)	0.0025 (10)	0.0063 (10)	0.0110 (10)
C20	0.0333 (15)	0.0204 (13)	0.0260 (13)	0.0022 (11)	0.0130 (12)	0.0087 (11)
C21	0.0289 (14)	0.0240 (13)	0.0236 (13)	0.0029 (11)	0.0113 (11)	0.0077 (11)
C22	0.0308 (14)	0.0265 (14)	0.0274 (14)	0.0054 (11)	0.0167 (12)	0.0117 (11)
C23	0.0249 (13)	0.0230 (13)	0.0271 (13)	0.0048 (10)	0.0141 (11)	0.0118 (11)
C24	0.0188 (11)	0.0198 (12)	0.0177 (11)	0.0031 (9)	0.0073 (9)	0.0063 (9)
C25	0.0247 (13)	0.0205 (13)	0.0310 (14)	0.0056 (10)	0.0097 (11)	0.0083 (11)
C26	0.0333 (15)	0.0209 (13)	0.0390 (17)	0.0038 (12)	0.0135 (13)	0.0094 (12)
C27	0.0280 (14)	0.0290 (15)	0.0347 (16)	−0.0065 (12)	0.0090 (12)	0.0084 (13)
C28	0.0196 (12)	0.0358 (16)	0.0274 (14)	−0.0016 (11)	0.0047 (11)	0.0133 (12)
C29	0.0191 (12)	0.0269 (13)	0.0225 (12)	0.0037 (10)	0.0070 (10)	0.0113 (11)
C31	0.0213 (14)	0.054 (2)	0.0461 (19)	−0.0018 (14)	0.0052 (13)	0.0271 (17)
C32	0.0229 (13)	0.0338 (15)	0.0215 (12)	0.0110 (11)	0.0092 (10)	0.0111 (11)
C33	0.0281 (14)	0.0329 (15)	0.0258 (14)	0.0088 (12)	0.0113 (11)	0.0105 (12)
C34	0.0287 (15)	0.0444 (18)	0.0258 (14)	0.0063 (13)	0.0117 (12)	0.0151 (13)
C35	0.0257 (14)	0.0445 (18)	0.0235 (14)	0.0084 (13)	0.0085 (11)	0.0078 (13)
C36	0.0415 (18)	0.0336 (17)	0.0357 (17)	0.0125 (14)	0.0184 (14)	0.0069 (14)
C37	0.0368 (16)	0.0317 (15)	0.0332 (16)	0.0104 (13)	0.0156 (13)	0.0126 (13)
C38	0.059 (2)	0.0353 (18)	0.053 (2)	0.0135 (17)	0.0338 (19)	0.0187 (16)
C39	0.043 (2)	0.055 (2)	0.0333 (17)	0.0127 (17)	0.0191 (15)	0.0089 (16)
C40	0.060 (2)	0.0376 (19)	0.057 (2)	0.0166 (17)	0.034 (2)	0.0235 (17)
C41	0.0418 (17)	0.0193 (13)	0.0308 (14)	−0.0011 (12)	0.0214 (13)	0.0054 (11)
C42	0.058 (2)	0.0305 (16)	0.0300 (15)	−0.0022 (15)	0.0212 (15)	0.0097 (13)
C43	0.084 (3)	0.0358 (19)	0.0299 (17)	−0.0007 (19)	0.0270 (19)	0.0067 (14)
C44	0.091 (3)	0.0269 (17)	0.049 (2)	0.0002 (18)	0.050 (2)	0.0044 (15)
C45	0.060 (2)	0.0287 (17)	0.068 (2)	0.0074 (16)	0.043 (2)	0.0098 (17)
C46	0.0448 (19)	0.0250 (15)	0.0474 (19)	0.0051 (14)	0.0252 (16)	0.0058 (14)
C47	0.064 (3)	0.062 (3)	0.0316 (18)	0.008 (2)	0.0095 (18)	0.0164 (18)
C48	0.147 (6)	0.047 (3)	0.066 (3)	0.018 (3)	0.070 (4)	0.002 (2)
C49	0.048 (2)	0.050 (2)	0.063 (3)	0.0204 (19)	0.0114 (19)	0.009 (2)
Ir1	0.01556 (6)	0.01830 (9)	0.01506 (6)	0.00312 (6)	0.00533 (4)	0.00823 (6)
F1A	0.062 (2)	0.066 (3)	0.266 (8)	0.045 (2)	0.090 (4)	0.104 (4)
F2A	0.284 (10)	0.056 (3)	0.090 (3)	0.062 (4)	0.078 (4)	0.049 (3)
F3A	0.063 (2)	0.0151 (13)	0.137 (4)	−0.0029 (13)	0.015 (2)	0.0082 (18)
F4A	0.060 (5)	0.264 (16)	0.072 (4)	0.096 (8)	0.028 (4)	0.043 (6)
F5A	0.032 (2)	0.120 (7)	0.089 (5)	0.015 (4)	0.008 (3)	0.085 (5)
F6A	0.036 (3)	0.084 (5)	0.213 (12)	−0.031 (3)	−0.036 (6)	0.091 (7)
F1B	0.062 (2)	0.066 (3)	0.266 (8)	0.045 (2)	0.090 (4)	0.104 (4)
F2B	0.284 (10)	0.056 (3)	0.090 (3)	0.062 (4)	0.078 (4)	0.049 (3)
F3B	0.063 (2)	0.0151 (13)	0.137 (4)	−0.0029 (13)	0.015 (2)	0.0082 (18)
F4B	0.057 (7)	0.237 (18)	0.038 (4)	0.070 (9)	−0.022 (3)	−0.030 (6)
F5B	0.033 (4)	0.100 (8)	0.087 (7)	0.013 (5)	0.034 (5)	0.058 (6)
F6B	0.021 (3)	0.089 (6)	0.229 (19)	0.009 (4)	0.016 (7)	0.111 (9)
C50	0.061 (3)	0.066 (3)	0.072 (3)	0.014 (2)	0.033 (2)	0.036 (2)
C51	0.058 (3)	0.087 (4)	0.077 (3)	0.027 (3)	0.044 (3)	0.038 (3)
C52	0.054 (3)	0.116 (5)	0.073 (3)	0.012 (3)	0.025 (2)	0.059 (3)
C53	0.047 (2)	0.075 (3)	0.082 (3)	0.006 (2)	0.001 (2)	0.040 (3)
C54	0.041 (2)	0.076 (3)	0.060 (3)	0.011 (2)	0.007 (2)	0.006 (2)
C55	0.045 (2)	0.079 (3)	0.056 (3)	0.002 (2)	0.027 (2)	0.009 (2)
Cl2	0.097 (2)	0.105 (2)	0.105 (2)	0.0543 (17)	0.0757 (18)	0.0701 (18)
Cl1	0.0867 (18)	0.0660 (15)	0.106 (2)	0.0037 (13)	0.0439 (16)	0.0529 (15)
Cl3	0.068 (5)	0.070 (5)	0.051 (4)	0.019 (4)	0.025 (4)	0.008 (4)
C59	0.120 (10)	0.084 (8)	0.046 (5)	0.034 (7)	0.056 (6)	0.031 (5)
C61	0.120 (10)	0.084 (8)	0.046 (5)	0.034 (7)	0.056 (6)	0.031 (5)
Ir2	0.01556 (6)	0.01830 (9)	0.01506 (6)	0.00312 (6)	0.00533 (4)	0.00823 (6)

### {µ-N,N'-Bis[3,5-bis(trifluoromethyl)phenyl]oxamidato}bis(bis{2-[4-(2,4,6-trimethylphenyl)pyridin-2-yl]phenyl-κ2C1,N'}iridium)–chlorobenzene–pentane (1/2.3/0.4) (IV) Geometric parameters (Å, º)

**Table:**

O1—C1	1.273 (3)	C33—C38	1.523 (5)
O1—Ir1	2.1757 (18)	C34—H34	0.9500
N1—C1	1.312 (3)	C34—C35	1.386 (5)
N1—C24	1.431 (3)	C35—C36	1.380 (5)
N1—Ir1^i^	2.182 (2)	C35—C39	1.505 (4)
N2—C8	1.357 (3)	C36—H36	0.9500
N2—C12	1.346 (3)	C36—C37	1.404 (4)
N2—Ir1	2.027 (2)	C37—C40	1.500 (5)
N3—C19	1.371 (3)	C38—H38A	0.9799
N3—C23	1.351 (3)	C38—H38B	0.9801
N3—Ir1	2.039 (2)	C38—H38C	0.9802
C1—C1^i^	1.521 (5)	C39—H39A	0.9803
C2—C3	1.390 (4)	C39—H39B	0.9811
C2—C7	1.417 (4)	C39—H39C	0.9798
C2—Ir1	1.993 (3)	C40—H40A	0.9807
C3—H3	0.9500	C40—H40B	0.9797
C3—C4	1.390 (4)	C40—H40C	0.9795
C4—H4	0.9500	C41—C42	1.401 (5)
C4—C5	1.382 (5)	C41—C46	1.386 (5)
C5—H5	0.9500	C42—C43	1.390 (5)
C5—C6	1.390 (4)	C42—C47	1.513 (6)
C6—H6	0.9500	C43—H43	0.9500
C6—C7	1.394 (4)	C43—C44	1.379 (7)
C7—C8	1.463 (4)	C44—C45	1.368 (6)
C8—C9	1.395 (4)	C44—C48	1.519 (5)
C9—H9	0.9500	C45—H45	0.9500
C9—C10	1.390 (4)	C45—C46	1.405 (5)
C10—C11	1.392 (4)	C46—C49	1.493 (6)
C10—C32	1.493 (4)	C47—H47A	0.9800
C11—H11	0.9500	C47—H47B	0.9800
C11—C12	1.374 (4)	C47—H47C	0.9800
C12—H12	0.9500	C48—H48A	0.9800
C13—C14	1.403 (4)	C48—H48B	0.9800
C13—C18	1.406 (4)	C48—H48C	0.9800
C13—Ir1	2.004 (3)	C48—H48D	0.9800
C14—H14	0.9500	C48—H48E	0.9800
C14—C15	1.391 (4)	C48—H48F	0.9800
C15—H15	0.9500	C49—H49A	0.9800
C15—C16	1.380 (5)	C49—H49B	0.9800
C16—H16	0.9500	C49—H49C	0.9800
C16—C17	1.383 (4)	F1A—C30A	1.307 (6)
C17—H17	0.9500	F2A—C30A	1.290 (6)
C17—C18	1.400 (4)	F3A—C30A	1.325 (6)
C18—C19	1.460 (4)	F1B—C30B	1.326 (14)
C19—C20	1.390 (4)	F2B—C30B	1.283 (14)
C20—H20	0.9500	F3B—C30B	1.338 (14)
C20—C21	1.388 (4)	C50—H50	0.9500
C21—C22	1.390 (4)	C50—C51	1.375 (6)
C21—C41	1.491 (4)	C50—C55	1.337 (6)
C22—H22	0.9500	C50—Cl1	1.743 (5)
C22—C23	1.377 (4)	C51—H51	0.9500
C23—H23	0.9500	C51—C52	1.358 (7)
C24—C25	1.399 (4)	C52—H52	0.9500
C24—C29	1.390 (4)	C52—C53	1.379 (8)
C25—H25	0.9500	C52—Cl2	1.717 (5)
C25—C26	1.386 (4)	C53—H53	0.9500
C26—C27	1.384 (4)	C53—C54	1.361 (7)
C26—C30A	1.493 (7)	C54—H54	0.9500
C26—C30B	1.50 (2)	C54—C55	1.343 (7)
C27—H27	0.9500	C55—H55	0.9500
C27—C28	1.383 (5)	Cl3—C56	1.613 (14)
C28—C29	1.393 (4)	C56—C57	1.349 (16)
C28—C31	1.495 (4)	C56—C58	1.368 (15)
C29—H29	0.9500	C57—H57	0.9500
C31—F4A	1.305 (7)	C57—C58^ii^	1.295 (15)
C31—F5A	1.261 (6)	C58—H58	0.9503
C31—F6A	1.303 (6)	C59—C60	1.423 (17)
C31—F4B	1.310 (8)	C59—C62^ii^	1.49 (3)
C31—F5B	1.280 (7)	C60—C61	1.456 (16)
C31—F6B	1.321 (8)	C60—C62^ii^	1.36 (3)
C32—C33	1.398 (4)	C61—C62^ii^	1.509 (16)
C32—C37	1.389 (4)	C61—C62	1.509 (16)
C33—C34	1.395 (4)		
			
C1—O1—Ir1	114.42 (15)	C36—C37—C40	120.1 (3)
C1—N1—C24	116.9 (2)	C33—C38—H38A	109.5
C1—N1—Ir1^i^	114.86 (16)	C33—C38—H38B	109.4
C24—N1—Ir1^i^	127.88 (16)	C33—C38—H38C	109.5
C8—N2—Ir1	116.55 (17)	H38A—C38—H38B	109.5
C12—N2—C8	119.4 (2)	H38A—C38—H38C	109.5
C12—N2—Ir1	123.55 (19)	H38B—C38—H38C	109.4
C19—N3—Ir1	115.17 (17)	C35—C39—H39A	109.5
C23—N3—C19	118.8 (2)	C35—C39—H39B	109.5
C23—N3—Ir1	125.44 (18)	C35—C39—H39C	109.6
O1—C1—N1	126.8 (2)	H39A—C39—H39B	109.4
O1—C1—C1^i^	118.3 (3)	H39A—C39—H39C	109.5
N1—C1—C1^i^	114.9 (3)	H39B—C39—H39C	109.4
C3—C2—C7	117.1 (2)	C37—C40—H40A	109.5
C3—C2—Ir1	128.4 (2)	C37—C40—H40B	109.4
C7—C2—Ir1	114.49 (18)	C37—C40—H40C	109.5
C2—C3—H3	119.3	H40A—C40—H40B	109.5
C4—C3—C2	121.4 (3)	H40A—C40—H40C	109.4
C4—C3—H3	119.3	H40B—C40—H40C	109.5
C3—C4—H4	119.6	C42—C41—C21	119.0 (3)
C5—C4—C3	120.9 (3)	C46—C41—C21	120.1 (3)
C5—C4—H4	119.6	C46—C41—C42	120.8 (3)
C4—C5—H5	120.3	C41—C42—C47	121.2 (3)
C4—C5—C6	119.3 (3)	C43—C42—C41	118.9 (4)
C6—C5—H5	120.3	C43—C42—C47	119.8 (4)
C5—C6—H6	120.1	C42—C43—H43	119.4
C5—C6—C7	119.9 (3)	C44—C43—C42	121.1 (4)
C7—C6—H6	120.1	C44—C43—H43	119.4
C2—C7—C8	114.7 (2)	C43—C44—C48	120.6 (5)
C6—C7—C2	121.3 (2)	C45—C44—C43	119.1 (3)
C6—C7—C8	124.0 (2)	C45—C44—C48	120.3 (5)
N2—C8—C7	113.3 (2)	C44—C45—H45	119.0
N2—C8—C9	119.8 (2)	C44—C45—C46	122.0 (4)
C9—C8—C7	126.5 (2)	C46—C45—H45	119.0
C8—C9—H9	119.5	C41—C46—C45	117.9 (4)
C10—C9—C8	121.1 (3)	C41—C46—C49	121.5 (3)
C10—C9—H9	119.5	C45—C46—C49	120.6 (4)
C9—C10—C11	117.2 (2)	C42—C47—H47A	109.5
C9—C10—C32	120.9 (2)	C42—C47—H47B	109.5
C11—C10—C32	121.6 (3)	C42—C47—H47C	109.5
C10—C11—H11	120.1	H47A—C47—H47B	109.5
C12—C11—C10	119.9 (3)	H47A—C47—H47C	109.5
C12—C11—H11	120.1	H47B—C47—H47C	109.5
N2—C12—C11	122.4 (3)	C44—C48—H48A	109.5
N2—C12—H12	118.8	C44—C48—H48B	109.5
C11—C12—H12	118.8	C44—C48—H48C	109.5
C14—C13—C18	117.2 (2)	H48A—C48—H48B	109.5
C14—C13—Ir1	128.5 (2)	H48A—C48—H48C	109.5
C18—C13—Ir1	114.28 (18)	H48B—C48—H48C	109.5
C13—C14—H14	119.6	H48D—C48—H48E	109.5
C15—C14—C13	120.7 (3)	H48D—C48—H48F	109.5
C15—C14—H14	119.6	H48E—C48—H48F	109.5
C14—C15—H15	119.5	C46—C49—H49A	109.5
C16—C15—C14	121.0 (3)	C46—C49—H49B	109.5
C16—C15—H15	119.5	C46—C49—H49C	109.5
C15—C16—H16	120.1	H49A—C49—H49B	109.5
C15—C16—C17	119.8 (3)	H49A—C49—H49C	109.5
C17—C16—H16	120.1	H49B—C49—H49C	109.5
C16—C17—H17	120.3	O1—Ir1—N1^i^	75.81 (7)
C16—C17—C18	119.5 (3)	N2—Ir1—O1	96.15 (8)
C18—C17—H17	120.3	N2—Ir1—N1^i^	91.38 (8)
C13—C18—C19	115.2 (2)	N2—Ir1—N3	174.60 (9)
C17—C18—C13	121.7 (3)	N3—Ir1—O1	82.90 (8)
C17—C18—C19	123.1 (3)	N3—Ir1—N1^i^	93.54 (8)
N3—C19—C18	113.7 (2)	C2—Ir1—O1	176.58 (9)
N3—C19—C20	119.8 (2)	C2—Ir1—N1^i^	103.47 (9)
C20—C19—C18	126.5 (3)	C2—Ir1—N2	80.51 (10)
C19—C20—H20	119.3	C2—Ir1—N3	100.49 (10)
C21—C20—C19	121.4 (3)	C2—Ir1—C13	87.62 (10)
C21—C20—H20	119.3	C13—Ir1—O1	93.36 (9)
C20—C21—C22	117.6 (3)	C13—Ir1—N1^i^	168.24 (9)
C20—C21—C41	121.4 (3)	C13—Ir1—N2	94.38 (10)
C22—C21—C41	121.0 (2)	C13—Ir1—N3	80.38 (10)
C21—C22—H22	120.2	F1A—C30A—C26	111.9 (4)
C23—C22—C21	119.5 (3)	F1A—C30A—F3A	105.4 (5)
C23—C22—H22	120.2	F2A—C30A—C26	113.6 (5)
N3—C23—C22	122.8 (3)	F2A—C30A—F1A	108.2 (6)
N3—C23—H23	118.6	F2A—C30A—F3A	104.7 (6)
C22—C23—H23	118.6	F3A—C30A—C26	112.6 (4)
C25—C24—N1	122.2 (2)	F1B—C30B—C26	107.8 (13)
C29—C24—N1	118.8 (2)	F1B—C30B—F3B	88.7 (15)
C29—C24—C25	119.0 (2)	F2B—C30B—C26	119.4 (14)
C24—C25—H25	120.0	F2B—C30B—F1B	124 (2)
C26—C25—C24	120.0 (3)	F2B—C30B—F3B	101.8 (18)
C26—C25—H25	120.0	F3B—C30B—C26	109.4 (14)
C25—C26—C30A	119.6 (3)	C51—C50—H50	118.8
C25—C26—C30B	116.7 (7)	C51—C50—Cl1	123.1 (4)
C27—C26—C25	121.2 (3)	C55—C50—H50	118.8
C27—C26—C30A	119.0 (3)	C55—C50—C51	122.3 (5)
C27—C26—C30B	121.6 (7)	C55—C50—Cl1	114.6 (4)
C26—C27—H27	120.6	C50—C51—H51	121.4
C28—C27—C26	118.8 (3)	C52—C51—C50	117.2 (5)
C28—C27—H27	120.6	C52—C51—H51	121.4
C27—C28—C29	120.9 (3)	C51—C52—H52	119.5
C27—C28—C31	120.0 (3)	C51—C52—C53	121.0 (5)
C29—C28—C31	119.0 (3)	C51—C52—Cl2	126.3 (4)
C24—C29—C28	120.1 (3)	C53—C52—H52	119.5
C24—C29—H29	119.9	C53—C52—Cl2	111.9 (4)
C28—C29—H29	119.9	C52—C53—H53	120.4
F4A—C31—C28	110.4 (4)	C54—C53—C52	119.2 (5)
F5A—C31—C28	114.4 (4)	C54—C53—H53	120.4
F5A—C31—F4A	108.2 (6)	C53—C54—H54	119.8
F5A—C31—F6A	107.1 (6)	C55—C54—C53	120.4 (5)
F6A—C31—C28	112.5 (4)	C55—C54—H54	119.8
F6A—C31—F4A	103.6 (7)	C50—C55—C54	119.9 (5)
F4B—C31—C28	111.8 (5)	C50—C55—H55	120.1
F4B—C31—F6B	109.3 (10)	C54—C55—H55	120.1
F5B—C31—C28	114.8 (5)	C57—C56—Cl3	120.9 (13)
F5B—C31—F4B	102.3 (8)	C57—C56—C58	122.1 (13)
F5B—C31—F6B	103.3 (8)	C58—C56—Cl3	116.9 (13)
F6B—C31—C28	114.3 (4)	C56—C57—H57	123.9
C33—C32—C10	118.5 (3)	C58^ii^—C57—C56	112.0 (15)
C37—C32—C10	121.2 (3)	C58^ii^—C57—H57	124.1
C37—C32—C33	120.3 (3)	C56—C58—H58	115.9
C32—C33—C38	121.0 (3)	C57^ii^—C58—C56	125.9 (15)
C34—C33—C32	119.4 (3)	C57^ii^—C58—H58	118.2
C34—C33—C38	119.6 (3)	C60—C59—C62^ii^	55.4 (14)
C33—C34—H34	119.4	C59—C60—C61	129.6 (19)
C35—C34—C33	121.2 (3)	C62^ii^—C60—C59	64.9 (15)
C35—C34—H34	119.4	C62^ii^—C60—C61	64.8 (12)
C34—C35—C39	119.8 (3)	C60^ii^—C61—C60	180.0
C36—C35—C34	118.6 (3)	C60^ii^—C61—C62^ii^	125.5 (14)
C36—C35—C39	121.7 (3)	C60^ii^—C61—C62	54.5 (14)
C35—C36—H36	119.1	C60—C61—C62^ii^	54.5 (14)
C35—C36—C37	121.8 (3)	C60—C61—C62	125.5 (14)
C37—C36—H36	119.1	C62^ii^—C61—C62	180.0
C32—C37—C36	118.7 (3)	C59^ii^—C62—C61	120.4 (18)
C32—C37—C40	121.1 (3)	C60^ii^—C62—C61	60.8 (10)
			
N1—C24—C25—C26	177.7 (3)	C27—C28—C29—C24	−0.5 (4)
N1—C24—C29—C28	−177.4 (2)	C27—C28—C31—F4A	106.7 (9)
N2—C8—C9—C10	1.1 (4)	C27—C28—C31—F5A	−131.0 (6)
N3—C19—C20—C21	−0.2 (4)	C27—C28—C31—F6A	−8.5 (8)
C1—N1—C24—C25	−44.8 (3)	C27—C28—C31—F4B	−59.8 (12)
C1—N1—C24—C29	134.4 (2)	C27—C28—C31—F5B	56.2 (8)
C2—C3—C4—C5	0.6 (5)	C27—C28—C31—F6B	175.3 (11)
C2—C7—C8—N2	−7.7 (3)	C29—C24—C25—C26	−1.5 (4)
C2—C7—C8—C9	165.6 (3)	C29—C28—C31—F4A	−72.2 (9)
C3—C2—C7—C6	4.6 (4)	C29—C28—C31—F5A	50.1 (7)
C3—C2—C7—C8	−172.7 (2)	C29—C28—C31—F6A	172.6 (8)
C3—C4—C5—C6	1.9 (5)	C29—C28—C31—F4B	121.3 (12)
C4—C5—C6—C7	−1.0 (5)	C29—C28—C31—F5B	−122.7 (7)
C5—C6—C7—C2	−2.2 (5)	C29—C28—C31—F6B	−3.6 (11)
C5—C6—C7—C8	174.8 (3)	C31—C28—C29—C24	178.4 (3)
C6—C7—C8—N2	175.1 (3)	C32—C10—C11—C12	−170.2 (3)
C6—C7—C8—C9	−11.6 (5)	C32—C33—C34—C35	0.7 (5)
C7—C2—C3—C4	−3.7 (4)	C33—C32—C37—C36	1.2 (5)
C7—C8—C9—C10	−171.8 (3)	C33—C32—C37—C40	−175.9 (3)
C8—N2—C12—C11	−4.1 (4)	C33—C34—C35—C36	−0.1 (5)
C8—C9—C10—C11	−4.9 (4)	C33—C34—C35—C39	−179.9 (3)
C8—C9—C10—C32	169.6 (3)	C34—C35—C36—C37	0.0 (5)
C9—C10—C11—C12	4.3 (4)	C35—C36—C37—C32	−0.6 (5)
C9—C10—C32—C33	−92.3 (4)	C35—C36—C37—C40	176.5 (3)
C9—C10—C32—C37	88.4 (4)	C37—C32—C33—C34	−1.2 (4)
C10—C11—C12—N2	0.1 (5)	C37—C32—C33—C38	179.5 (3)
C10—C32—C33—C34	179.5 (3)	C38—C33—C34—C35	179.9 (3)
C10—C32—C33—C38	0.2 (4)	C39—C35—C36—C37	179.9 (3)
C10—C32—C37—C36	−179.6 (3)	C41—C21—C22—C23	177.2 (3)
C10—C32—C37—C40	3.3 (5)	C41—C42—C43—C44	−1.1 (5)
C11—C10—C32—C33	82.0 (4)	C42—C41—C46—C45	1.0 (5)
C11—C10—C32—C37	−97.2 (4)	C42—C41—C46—C49	−179.8 (3)
C12—N2—C8—C7	177.3 (2)	C42—C43—C44—C45	0.7 (6)
C12—N2—C8—C9	3.5 (4)	C42—C43—C44—C48	180.0 (4)
C13—C14—C15—C16	−0.1 (5)	C43—C44—C45—C46	0.5 (6)
C13—C18—C19—N3	2.4 (3)	C44—C45—C46—C41	−1.4 (5)
C13—C18—C19—C20	−177.6 (3)	C44—C45—C46—C49	179.4 (4)
C14—C13—C18—C17	2.6 (4)	C46—C41—C42—C43	0.2 (5)
C14—C13—C18—C19	−175.8 (2)	C46—C41—C42—C47	178.6 (3)
C14—C15—C16—C17	1.4 (5)	C47—C42—C43—C44	−179.5 (4)
C15—C16—C17—C18	−0.7 (5)	C48—C44—C45—C46	−178.7 (4)
C16—C17—C18—C13	−1.3 (5)	Ir1—O1—C1—N1	171.2 (2)
C16—C17—C18—C19	176.9 (3)	Ir1—O1—C1—C1^i^	−11.4 (3)
C17—C18—C19—N3	−175.9 (3)	Ir1^i^—N1—C1—O1	169.4 (2)
C17—C18—C19—C20	4.1 (5)	Ir1^i^—N1—C1—C1^i^	−8.1 (3)
C18—C13—C14—C15	−1.9 (4)	Ir1^i^—N1—C24—C25	142.3 (2)
C18—C19—C20—C21	179.8 (3)	Ir1^i^—N1—C24—C29	−38.4 (3)
C19—N3—C23—C22	2.0 (4)	Ir1—N2—C8—C7	5.2 (3)
C19—C20—C21—C22	1.9 (4)	Ir1—N2—C8—C9	−168.6 (2)
C19—C20—C21—C41	−177.0 (3)	Ir1—N2—C12—C11	167.4 (2)
C20—C21—C22—C23	−1.7 (4)	Ir1—N3—C19—C18	−9.9 (3)
C20—C21—C41—C42	97.9 (4)	Ir1—N3—C19—C20	170.1 (2)
C20—C21—C41—C46	−81.9 (4)	Ir1—N3—C23—C22	−168.9 (2)
C21—C22—C23—N3	−0.3 (4)	Ir1—C2—C3—C4	176.9 (2)
C21—C41—C42—C43	−179.7 (3)	Ir1—C2—C7—C6	−175.9 (2)
C21—C41—C42—C47	−1.3 (5)	Ir1—C2—C7—C8	6.8 (3)
C21—C41—C46—C45	−179.1 (3)	Ir1—C13—C14—C15	175.7 (2)
C21—C41—C46—C49	0.1 (5)	Ir1—C13—C18—C17	−175.3 (2)
C22—C21—C41—C42	−81.0 (4)	Ir1—C13—C18—C19	6.3 (3)
C22—C21—C41—C46	99.2 (4)	C30A—C26—C27—C28	176.6 (3)
C23—N3—C19—C18	178.2 (2)	C30B—C26—C27—C28	−169.8 (8)
C23—N3—C19—C20	−1.8 (4)	C50—C51—C52—C53	2.9 (8)
C24—N1—C1—O1	−4.4 (4)	C50—C51—C52—Cl2	−166.3 (5)
C24—N1—C1—C1^i^	178.1 (2)	C51—C50—C55—C54	−2.1 (8)
C24—C25—C26—C27	−0.2 (5)	C51—C52—C53—C54	−2.4 (8)
C24—C25—C26—C30A	−175.2 (3)	C52—C53—C54—C55	−0.5 (8)
C24—C25—C26—C30B	171.6 (7)	C53—C54—C55—C50	2.7 (8)
C25—C24—C29—C28	1.9 (4)	C55—C50—C51—C52	−0.7 (8)
C25—C26—C27—C28	1.6 (5)	Cl2—C52—C53—C54	168.3 (4)
C25—C26—C30A—F1A	−40.4 (7)	Cl1—C50—C51—C52	−179.4 (4)
C25—C26—C30A—F2A	82.5 (7)	Cl1—C50—C55—C54	176.7 (4)
C25—C26—C30A—F3A	−158.8 (5)	Cl3—C56—C57—C58^ii^	177.4 (15)
C25—C26—C30B—F1B	138 (2)	Cl3—C56—C58—C57^ii^	−177.5 (16)
C25—C26—C30B—F2B	−74 (2)	C57—C56—C58—C57^ii^	0 (3)
C25—C26—C30B—F3B	42.8 (17)	C58—C56—C57—C58^ii^	0 (3)
C26—C27—C28—C29	−1.2 (5)	C59—C60—C61—C62	−177 (2)
C26—C27—C28—C31	179.9 (3)	C59—C60—C61—C62^ii^	3 (2)
C27—C26—C30A—F1A	144.5 (6)	C60—C61—C62—C59^ii^	−177.3 (18)
C27—C26—C30A—F2A	−92.7 (7)	C60^ii^—C61—C62—C59^ii^	2.7 (18)
C27—C26—C30A—F3A	26.0 (6)	C60—C61—C62—C60^ii^	180.0
C27—C26—C30B—F1B	−50 (2)	C62^ii^—C59—C60—C61	−3 (2)
C27—C26—C30B—F2B	98.0 (19)	C62^ii^—C60—C61—C62	180.0
C27—C26—C30B—F3B	−145.4 (15)		

Symmetry codes: (i) −*x*+1, −*y*+1, −*z*+1; (ii) −*x*, −*y*, −*z*.
